# A revision of the family Ameroseiidae (Acari, Mesostigmata), with some data on Slovak fauna

**DOI:** 10.3897/zookeys.704.13304

**Published:** 2017-09-29

**Authors:** Peter Mašán

**Affiliations:** 1 Institute of Zoology, Slovak Academy of Sciences, Dúbravská cesta 9, 845 06 Bratislava, Slovakia

**Keywords:** Mites, systematics, revision, identification keys, new taxa, catalogue, morphology, taxonomy, Slovak fauna

## Abstract

The family Ameroseiidae Evans, 1961 (Acari: Mesostigmata) includes a total of 12 valid and adequately described genera, namely *Afrocypholaelaps* Elsen, 1972, *Ameroseiella* Bregetova, 1977, *Ameroseius* Berlese, 1904, *Asperolaelaps* Womersley, 1956, *Brontispalaelaps* Womersley, 1956, *Epicriopsis* Berlese, 1916, *Hattena* Domrow, 1963, *Kleemannia* Oudemans, 1930, *Neocypholaelaps* Vitzthum, 1942, *Pseudoameroseius* **gen. n.**, *Sertitympanum* Elsen & Whitaker, 1985 and *Sinoseius* Bai & Gu, 1995. One of these genera includes subgenera, namely Kleemannia (Primoseius) Womersley, 1956.

All genera are reviewed and re-diagnosed, and a dichotomous key is provided for their identification. *Ameroseius* (50 species), *Kleemannia* (28 species) and *Neocypholaelaps* (22 species) are the largest genera in the family. *Ameroseiella*, *Kleemannia*, Kleemannia (Primoseius) and *Sinoseius* are considered to be valid taxa and, in presented systematic classification, they are removed from synonymy with *Ameroseius*. The genus *Pseudoameroseius* **gen. n.**, with type species *Ameroseius michaelangeli* Moraza, 2006 (from Canary Islands), is newly erected to further refine broad primary concept of *Ameroseius* as understood by some former authors (Karg, Bregetova). *Asperolaelaps* is removed from synonymy with *Neocypholaelaps*. Three new species are here described, namely *Ameroseius renatae* **sp. n.** (based on specimens from Slovakia), *Kleemannia dolichochaeta* **sp. n.** (from Spain) and *Kleemannia miranda* **sp. n.** (from U.S.A.).

The following new junior synonymies are proposed: *Ameroseius apodius* Karg, 1971 = *Ameroseiella macrochelae* (Westerboer, 1963); *Ameroseius bregetovae* Livshits & Mitrofanov, 1975 = *Neocypholaelaps favus* Ishikawa, 1968; *Ameroseius chinensis* Khalili-Moghadam & Saboori, 2016 = *Ameroseius guyimingi* Ma, 1997; *Ameroseius crassisetosus* Ye & Ma, 1993, *Ameroseius qinghaiensis* Li & Yang, 2000 and *Ameroseius norvegicus* Narita, Abduch & Moraes, 2015 = *Ameroseius corbiculus* (Sowerby, 1806); *Ameroseius dubitatus* Berlese, 1918 = *Kleemannia plumosa* (Oudemans, 1902); *Ameroseius eumorphus* Bregetova, 1977 and *Kleemannia potchefstroomensis* Kruger & Loots, 1980 = *Kleemannia pseudoplumosa* (Rack, 1972); *Ameroseius gilarovi* Petrova, 1986 = *Kleemannia plumigera* Oudemans, 1930; *Ameroseius imparsetosus* Westerboer, 1963 = *Ameroseius georgei* (Turk, 1943); *Ameroseius lanatus* Solomon, 1969 and *Ameroseius fimetorum* Karg, 1971 = *Kleemannia tenella* (Berlese, 1916); *Ameroseius lanceosetis* Livshits & Mitrofanov, 1975 = *Kleemannia pavida* (C. L. Koch, 1839); *Ameroseius marginalis* Fan & Li, 1993 and *Ameroseius sichuanensis* Fan & Li, 1993 = *Kleemannia insignis* (Bernhard, 1963); *Ameroseius pseudofurcatus* Livshits & Mitrofanov, 1975 = *Ameroseius furcatus* Karg, 1971; *Ameroseius stramenis* Karg, 1976 and Lasioseius (Lasioseius) gracilis Halbert, 1923 = *Kleemannia delicata* (Berlese, 1918); *Epicriopsis langei* Livshits & Mitrofanov, 1975 and *Epicriopsis baloghi* Kandil, 1978 = *Epicriopsis palustris* Karg, 1971; *Epicriopsis rivus* Karg, 1971 = *Epicriopsis mirabilis* Willmann, 1956; *Neocypholaelaps ewae* Haitlinger, 1987 = *Neocypholaelaps indicus* Evans, 1963; *Neocypholaelaps lindquisti* Prasad, 1968, *Afrocypholaelaps ranomafanaensis* Haitlinger, 1987 and *Afrocypholaelaps analicullus* Ho, Ma, Wang & Severinghaus, 2010 = *Afrocypholaelaps africanus* (Evans, 1963); *Sinoseius pinnatus* Huhta & Karg, 2010 = *Sinoseius lobatus* Bai, Gu & Fang, 1995.

*Ameroseius womersleyi* Mašán, is a replacement name proposed for *Ameroseius ornatus* Womersley, 1956, a junior secondary homonym of *Cornubia ornata* Turk, 1943 [= *Ameroseius corbiculus* (Sowerby, 1806)]. *Cornubia georgei* Turk, 1943 is removed from synonymy with *Ameroseius corbiculus* (Sowerby, 1806).

An annotated catalogue of the world species of Ameroseiidae is provided, partly based on type (in more than 60 species) and non-type specimens from various museum deposits and personal collections, including new or revised material from Slovakia. It contains 206 named species (138 valid species, 37 synonyms, nine unrecognizable species, 15 species previously excluded from Ameroseiidae, and seven “nomina nuda”), with details of their authorship, synonyms, nomenclatural and bibliographic details, generic placement, and morphology. Altogether 23 new combinations are proposed.

The genus *Sertitympanum* with *Sertitympanum nodosum* (Sheals, 1962) and two further species, namely *Kleemannia kosi* El-Badry, Nasr & Hafez, 1979 and *Kleemannia parplumosa* Nasr & Abou-Awad, 1986, are reported from Europe for the first time. New keys are given for identification of 37 species belonging to eight genera which have been found in Europe to date (*Ameroseiella*, *Ameroseius*, *Epicriopsis*, *Kleemannia*, *Neocypholaelaps*, *Pseudoameroseius* **gen. n.**, *Sertitympanum* and *Sinoseius*). All of these genera except *Pseudoameroseius* gen. nov. and *Sertitympanum* occur in Slovakia. So, the fauna of Slovakia includes six genera and 27 species, including ten first reports for the country.

## Introduction

Mites of the family Ameroseiidae Evans, 1961 are commonly found in greatly heterogeneous substrates and habitats. A large and diverse ameroseiid fauna is saproxylic and mycophagous (fungivorous), thereby depending upon dead and decaying wood and wood-inhabiting fungi (including ambrosia fungi), or the presence of fresh fruiting bodies (sporocarps) of wood-destroying bracket fungi or other saproxylic animals such as bark and wood-boring beetles. It uses snags, stumps, logs, subcortical area, insect galleries, stems and other forms of woody material for food, foraging, shelter, development, reproduction and other life activities (Westerboer and Bernhard 1963, De Leon 1964, Flechtmann 1985, Mašán 1998). Some ameroseiid species in tropical areas are nectar- and pollen-feeders, associated with various flowers and flower-visiting animals, namely bees and wasps (Hymenoptera, Apoidea), butterflies (Lepidoptera), and rarely birds (Evans 1963a; Elsen 1972a, 1972b, 1973; Klimov et al. 2016; Allred 1970). Along with saproxylic and plant-inhabiting ameroseiids, there is a large group of aerial and potentially cosmopolitan species that scavenge for fungal spores and hyphae in various synanthropic habitats such as inside of human houses (especially new buildings and those under construction), office blocks, cellars, farms, granaries, food and product storages, haystacks, and arable lands (Rack 1963, 1972; Hughes 1976). A rather small group of ameroseiid mites contains edaphic species associated with humid soils with high content of raw humus. Phoretic associations with insects and other animals have been established for many different species of the family, but they are not common among the species of the European fauna. It is assumed that many ameroseiids are non-phoretic species widely distributed by wind and air currents.

The Ameroseiidae, in the concept adopted in this paper, currently comprises the following 12 recognisable genera: *Afrocypholaelaps*, *Ameroseiella*, *Ameroseius*, *Asperolaelaps*, *Brontispalaelaps*, *Epicriopsis*, *Hattena*, *Kleemannia*, *Neocypholaelaps*, *Pseudoameroseius* gen. nov., *Sertitympanum* and *Sinoseius*. Ameroseiid mites can be found over all climatic zones of the world. Only four of these genera, namely *Afrocypholaelaps*, *Asperolaelaps*, *Brontispalaelaps* and *Hattena*, exclusive members of tropical fauna, are not reported from Europe up to now. The family is relatively poorly known in comparison with other groups of Mesostigmata having medical or economic importance. It appears to be morphologically heterogeneous with an unstable genus-level classification, but this has largely been caused by synonymization of some well founded genera by some of the renowned authors. Karg (1993) distinguished only three European genera here regarded to be Ameroseiidae, also including *Melichares* Hering, 1838, *Proctolaelaps* Berlese, 1923, and *Garmaniella* Westerboer & Bernhard, 1963 in the family, but these genera are now placed in Melicharidae, following the modern concept of contemporary authors (Moraes et al. 2016). For example, Halliday (1997) and Hallan (2005) have listed eight and nine valid genera respectively.

The genus *Ameroseius* has been reviewed by Westerboer and Bernhard (1963), partly in collaboration with Hirschmann, as a member of the family Phytoseiidae. Evans (1963a) provided basic information for subsequent work on Ameroseiidae. He discussed the external morphology of the family and elaborated a key to five genera then included in this family (*Ameroseius*, *Brontispalaelaps*, *Epicriopsis*, *Kleemannia* and *Neocypholaelaps*). Evans (1963a) retained the generic status of *Kleemannia* Oudemans, 1930 by adding specific diagnostic characters well distinct from those in *Ameroseius* Berlese, 1904. Some other authors considered *Kleemannia* was probably a synonym of *Ameroseius* (Westerboer and Bernhard 1963; Bregetova 1977; Karg 1993, 2005; Halliday 1997; Karg and Schorlemmer 2009; Narita et al. 2013b; a.o.). In this paper, *Kleemannia* is considered a valid genus (for specific differential characters of the genus see “Remarks” under *Kleemannia* in the catalogue below).

My objectives in this study are (1) to review and newly diagnose the ameroseiid genera known up to now in the taxonomic literature, (2) to take nomenclatural actions to correct problems with systematic status of some of the existing species (names), (3) to resolve some of the classificatory problems, (4) to consolidate previously available information on the inadequately or inconsistently described taxa, (5) to provide dichotomous keys for the identification of specific and supraspecific taxa found in Europe, (6) to provide a catalogue of all the species, with their respective synonyms if any, and references to original description, and (7) to summarise known and new faunistic data from Slovakia.

## Materials and methods

The essential part of this paper consists of a revision and catalogue of supraspecific and specific taxa arranged in alphabetical order with basic bibliographical references to taxonomic nomenclature and previously published records on each species. For many species short taxonomic notes are given. I conducted a comprehensive search for data on Ameroseiidae in the world literature, up to April 2017, using a range of electronic and print resources. In addition, I examined type specimens of more than 60 species treated herein (see “Comparative material” in individual species), including published or unpublished non-type material from various European countries and Iran, and borrowed from the existing slide collections in a number of zoological and museum institutions (see abbreviations below). I tried to base my conclusion on the direct examination of type specimens wherever possible. In some cases (especially in China), I was unable to contact the relevant curators, and in other cases the type material has been destroyed or lost. The available information on some species and genera is still incomplete, and many of the existing species cannot be properly identified. However, the revision presented here should provide a basis for further study of the family in the future.

Altogether almost 1,250 specimens found in 130 samples are newly reported from Slovakia. If accessible, older material, published by Slovak authors in the past, was re-examined and revised, particularly for rare species. Names of incorrectly identified species are also given, within the survey of revised data. A variety of quantitative methods (mainly a core sampler or sifting) and individual collecting were used. Quantitative collection methods include extraction of heterogeneous samples and organic substrates including grassland soils, soils from wet alluvial or littoral habitats, the rhizosphere of herbs, fresh or old dung, compost, and decaying plant or animal remains, etc. Sifting was used to obtain living mites from leaf and needle litter, soil detritus, raw humus, alluvial litter deposits, moss and rotting wood. Mites were extracted from soil samples and sifted detritus by means of a modified Berlese-Tullgren funnel provided with a 40-watt bulb. The extraction lasted 48–72 hours. Individual collecting was especially used to obtain mites living on bracket fungi and decaying wood, and under bark of dying trees. The mites were transferred to tubes with 70% ethanol using fine forceps. The type material of the new species is deposited at the Institute of Zoology, Slovak Academy of Sciences, Bratislava.

For identification, specimens were mounted on permanent microscope slides using Swan’s medium (gum arabic/chloral hydrate). Illustrations were made using photographed specimens. A Leica DM 1000 light microscope equipped with a stage-calibrated ocular micrometer and a Leica EC3 digital camera was used to obtain measurements and photos. Measurements were made from slide-mounted specimens. Some multiple images were combined using the CombineZP software program (Hadley, 2010). Lengths of shields were measured along their midlines, and widths at their widest point (if not otherwise specified in the description). Dorsal setae were measured from the bases of their insertions to their tips. Measurements are mostly presented as ranges (minimum to maximum).

**Figure 1.**
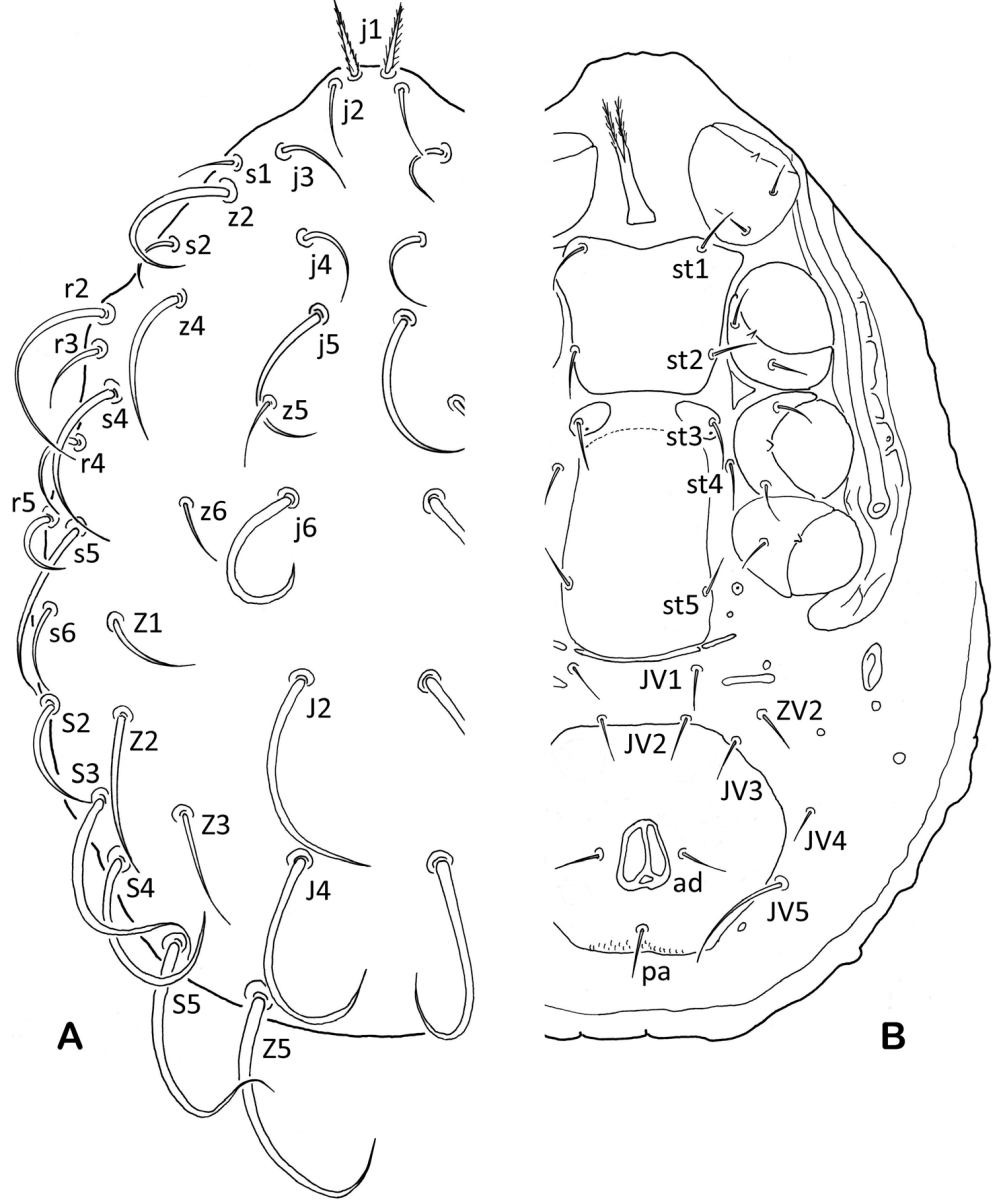
Chaetotactic notation of idiosomal setae on dorsal (**A**) and ventral (**B**) surface of idiosoma.

The nomenclature used for the dorsal idiosomal chaetotaxy is based on that of Lindquist and Evans (1965); leg chaetotaxy is that of Evans (1963b), and other anatomical structures follow Evans and Till (1979). The chaetotaxy symbols used here are illustrated in Fig. 1. Some dorsal shield setae are difficult to recognise, and I have identified them on the basis of their relative positions, not as first appears during ontogenetic development in the developmental stages. In some cases I have amended specific names to comply with the International Code of Zoological Nomenclature.

### Abbreviations

**BMNH** Natural History Museum, London, United Kingdom. Formerly British Museum (Natural History).

**CJH** Collection Jalil Hajizadeh, Rasht, Iran.

**CKI** Collection Kazuo Ishikawa, Kuwabara, Matsuyama, Japan.

**ESALQ** Luiz de Queiroz College of Agriculture, Department of Entomology and Acarology, University of Sao Paulo (Escola Superior de Agricultura "Luiz de Queiroz", Departamento de Entomologia e Acarologia, Universidade de São Paulo), Sao Paulo, Brazil.

**HNHM** Hungarian Natural History Museum (Magyar Természettudományi Múzeum), Budapest, Hungary.

**ISZA** Agriculture Research Council – Research Centre for Agrobiology and Pedology (Consiglio per la ricerca e la sperimentazione in agricoltura – Centro di ricerca per l‘agrobiologia e la pedologia), Florence, Italy. Formerly Istituto Sperimentale per la Zoologia Agraria.

**IZSAV** Institute of Zoology, Slovak Academy of Sciences (Ústav zoológie, Slovenská akadémia vied), Bratislava, Slovakia.

**LACM** Natural History Museum of Los Angeles County, Entomology Section, Los Angeles, USA. Formerly Los Angeles County Museum.

**MPUW** Władysław Rydzewski Museum of Natural History, Wrocław University (Muzeum Przyrodnicze im. Prof. Władysława Rydzewskiego, Uniwersytet Wrocławski), Wrocław, Poland.

**MRAC** The Royal Museum for Central Africa (Musée Royal de l’Afrique Centrale), Tervuren, Belgium.

**MZNA** Museum of Zoology, University of Navarra (Museo de Zoología, Universidad de Navarra), Pamplona, Spain.

**NMINH** National Museum of Ireland, Natural History Division (Ard-Mhúsaem na hÉireann, Stair an Dúlra), Dublin, Ireland.

**RMNH** Naturalis Biodiversity Center (Nederlands Centrum voor Biodiversiteit Naturalis), Leiden, The Netherlands. Formerly Rijksmuseum van Natuurlijke Historie.

**SAMA** South Australian Museum, Adelaide, Australia.

**SHKC** Collection Shahrooz Kazemi, Kerman, Iran.

**ZMB** Natural History Museum, Leibniz Institute for Evolutionary and Biodiversity Research at the Humboldt University (Museum für Naturkunde, Leibniz-Institut für Evolutions- und Biodiversitätsforschung an der Humboldt-Universität), Berlin, Germany. Formerly Zoologisches Museum Berlin.

**ZMH** Grindel Biocentre and Zoological Museum, University of Hamburg (Biozentrum Grindel und Zoologisches Museum, Universität Hamburg), Hamburg, Germany. Formerly Zoologisches Museum Hamburg.

**ZMT** Zoological Museum, University of Turku (Turun yliopiston eläinmuseo), Turku, Finland.

**ZSM** Bavarian State Collection of Zoology (Zoologische Staatssammlung München), Munich, Germany.

## On leg chaetotaxy in the Ameroseiidae

I have examined the leg chaetotaxy patterns in all ameroseiid species found in Slovakia (27 species), and 11 further species from outside Slovakia, and the results are summarised in Tables 1–3. The examination was motivated by the existence of many dissonant and inconsistent data on leg setation available in world literature on Ameroseiidae. In addition, chaetotaxy of genu III has been checked in all species available for this study.

Some specific and supraspecific differences of setation in individual leg segments found in Ameroseiidae can provide useful morphological criteria for their systematic classification. Evans (1963a) presented an identification key to five genera of Ameroseiidae drawing also on specific characters of leg chaetotaxy. As he summarised in a table, chaetotaxy of genua and tibiae of legs III and IV proved to be useful basis for distinguishing some ameroseiid genera such as *Brontispalaelaps* (genu III with no ventral setae), *Kleemannia* (genua III and IV and tibia IV each with two posterolateral setae), and *Epicriopsis* (genu and tibia of leg IV with no posterolateral seta). His results agree with those given for *Brontispalaelaps* by Halliday (1997) and Silva et al. (2014). Two posterolateral setae on genua III and IV and tibia IV, as stated for *Kleemannia* by Evans, can be found also in two other ameroseiid genera, namely *Ameroseiella* and *Sertitympanum* (Table 1). The above mentioned absence of posterolateral seta (pl) on genu IV and tibia IV in *Epicriopsis* introduced by Evans (1963a) appears to be an error, and different from what was observed for this genus in the present study (Table 2), and from what had been reported in the original descriptions of *Epicriopsis atuberculatus* and *Epicriopsis walteri* by Narita and Moraes (2016) and Halliday (1997), respectively.

The chaetotaxy of individual leg segments is a valuable source of differential characters not only for the genus-level classification (Table 1) but also for species-level classification within some genera, such as *Epicriopsis* (Tables 2 and 3). Unspecialised edaphic species of the genus *Epicriopsis* exhibit considerably more interspecific variation than those in other ameroseiid genera. Evans (1963a) reported the chaetotaxy of leg I to be constant throughout the family but I found the anteroventral seta (av2) on tibia I and genu I is absent in adults of *Epicriopsis mirabilis* (Table 2). In this study, I could not find interspecific variation in 12 species of *Ameroseius* and 11 species of *Kleemannia* (enumerated in Table 1), the most speciose ameroseiid genera. In addition, genu III had constant and specific number of nine or ten setae respectively in all of *Ameroseius* and *Kleemannia* species available for this study (34 species of *Ameroseius* and 16 species of *Kleemannia*). Although the chaetotaxy of individual ameroseiid genera is variable, two widely distributed patterns characteristic of a large group of genera in Ameroseiidae can be identified, namely (1) a group with *Ameroseius*, *Neocypholaelaps* and *Pseudoameroseius* gen. nov., having common chaetotactic pattern that can form the basis for comparison with the other genera; and (2) a group with *Kleemannia*, *Sertitympanum* and *Ameroseiella*, having an additional posterolateral seta on three leg segments (genua III and IV, tibia IV). On the basis of the single posterolateral seta on all segments of legs III and IV, *Epicriopsis* has a greater affinity with the species associated with the genus *Ameroseius* than *Kleemannia*. The genus *Sinoseius* has an intermediate position between the two groups. It is characterised by having genu III and tibia III with only one posterolateral seta (shared with *Ameroseius*), tibia IV with two posterolateral setae (shared with *Kleemannia*), and genu IV with two ventral setae, including a posteroventral seta (rarely expressed in Ameroseiidae).

**Table 1.** Leg chaetotaxy in adults of examined species of Ameroseiidae. Figures in bold face represent infrequent numbers of setae, and non-standard expression of leg setation.

*Afrocypholaelaps (africanus)*								
Femur I–IV	2-3/1, 2/2-2	(12)	2-2/1, 2/**1**-1	(**9**)	1-2/1, 1/0-1	(6)	1-2/1, 1/0-1	(6)
Genu I–IV	2-3/2, 2/1-2	(12)	**1**-3/1, 2/1-**1**	(**9**)	**1**-2/1, 2/1-1	(**8**)	2-2/1, 3/0-1	(9)
Tibia I–IV	2-3/2, 2/1-2	(12)	**1**-2/1, 2/1-**1**	(**8**)	**1**-1/1, 2/1-1	(**7**)	2-2/1, 2/1-1	(9)
***Ameroseiella*** (*macrochelae*) ***Kleemannia*** (*delicata*, *dolichochaeta* sp. n., *insignis*, *mineiro*, *parplumosa*, *pavida*, *plumea*, *plumigera*, *plumosa*, *pseudoplumosa*, *tenella*) ***Sertitympanum*** (*aegyptiacum*, *nodosum*)								
Femur I–IV	2-3/1, 2/2-2	(12)	2-2/1, 2/2-1	(10)	1-2/1, 1/0-1	(6)	1-2/1, 1/0-1	(6)
Genu I–IV	2-3/2, 2/1-2	(12)	2-3/1, 2/1-2	(11)	2-2/1, 2/1-**2**	(**10**)	2-2/1, 3/0-1	(9)
Tibia I–IV	2-3/2, 2/1-2	(12)	2-2/1, 2/1-2	(10)	2-1/1, 2/1-**2**	(**9**)	2-2/1, 2/1-**2**	(**10**)
***Asperolaelaps*** (*rotundus*, *sextuberculi*)								
Femur I–IV	2-3/1, 2/2-2	(12)	2-2/1, 2/2-1	(10)	1-2/1, 1/0-1	(6)	1-2/1, 1/0-1	(6)
Genu I–IV	2-3/2, 2/1-2	(12)	2-3/1, 2/1-2	(11)	2-2/1, 2/1-1	(9)	2-2/1, 3/0-1	(9)
Tibia I–IV	2-3/2, 2/1-2	(12)	2-2/1, 2/1-2	(10)	2-1/1, 2/1-1–**2**	(8–**9**)	2-2/1, 2/1-**2**	(**10**)
***Ameroseius*** (*callosus*, *cavernosus*, *corbiculus*, *corniculus*, *fungicola*, *furcatus*, *georgei*, *lidiae*, *longitrichus*, *renatae* sp. n., *sculptilis*, *ulmi*) ***Neocypholaelaps*** (*favus*, *indicus*) ***Pseudoameroseius*** gen. n. (*michaelangeli*)								
Femur I–IV	2-3/1, 2/2-2	(12)	2-2/1, 2/2-1	(10)	1-2/1, 1/0-1	(6)	1-2/1, 1/0-1	(6)
Genu I–IV	2-3/2, 2/1-2	(12)	2-3/1, 2/1-2	(11)	2-2/1, 2/1-1	(9)	2-2/1, 3/0-1	(9)
Tibia I–IV	2-3/2, 2/1-2	(12)	2-2/1, 2/1-2	(10)	2-1/1, 2/1-1	(8)	2-2/1, 2/1-1	(9)
***Epicriopsis*** (*horridus*, *hungaricus*, *mirabilis*, *palustris*, *suedus*)								
Femur I–IV	2-3/1, 2/2-2	(12)	2-2/1, 2/2-1	(10)	1-2/1, 1/0-1	(6)	1-2/1, 1/0-1	(6)
Genu I–IV	2-3/**1**–2, 2/1-2	(**11**–12)	2-3/1, 2/**0**–1-2	(**10**–11)	2-2/1, 2/**0**–1-1	(**8**–9)	2-2/1, **2**–3/0-1	(**8**–9)
Tibia I–IV	2-3/**1**–2, 2/1-2	(**11**–12)	2-2/1, 2/1-2	(10)	2-1/1, 2/1-1	(8)	2-2/1, 2/1-1	(9)
***Sinoseius*** (*lobatus*)								
Femur I–IV	2-3/1, 2/2-2	(12)	2-2/1, 2/2-1	(10)	1-2/1, 1/0-1	(6)	1-2/1, 1/0-1	(6)
Genu I–IV	2-3/2, 2/1-2	(12)	2-3/1, 2/1-2	(11)	2-2/1, 2/1-1	(9)	2-2/1, 3/**1**-1	(**10**)
Tibia I–IV	2-3/2, 2/1-2	(12)	2-2/1, 2/1-2	(10)	2-1/1, 2/1-1	(8)	2-2/1, 2/1-**2**	(**10**)

**Table 2.** Leg chaetotaxy in adults of *Epicriopsis* with known leg chaetotaxy. Figures in bold face represent infrequent numbers of setae (* – autapomorphic expression of leg setation).

*Epicriopsis atuberculatus* (after Narita & Moraes, 2016)								
Femur I–IV	2-3/1, 2/2-2	(12)	2-2/1, 2/2-1	(10)	1-2/1, 1/0-1	(6)	1-2/1, 1/0-1	(6)
Genu I–IV	2-3/2, 2/1-2	(12)	2-3/1, 2/**0**-2	(**10**)	2-2/1, **3/0**-1	(**9**)	2-2/1, 3/0-1	(9)
Tibia I–IV	2-3/2, 2/1-2	(12)	2-2/1, 2/1-2	(10)	2-1/1, 2/1-1	(8)	2-2/1, 2/1-1	(9)
***Epicriopsis horridus***								
Femur I–IV	2-3/1, 2/2-2	(12)	2-2/1, 2/2-1	(10)	1-2/1, 1/0-1	(6)	1-2/1, 1/0-1	(6)
Genu I–IV	2-3/2, 2/1-2	(12)	2-3/1, 2/**0**-2	(**10**)	2-2/1, 2/**0**-1	(**8**)	2-2/1, **2**/0-1	(**8**)*
Tibia I–IV	2-3/2, 2/1-2	(12)	2-2/1, 2/1-2	(10)	2-1/1, 2/1-1	(8)	2-2/1, 2/1-1	(9)
***Epicriopsis hungaricus***								
Femur I–IV	2-3/1, 2/2-2	(12)	2-2/1, 2/2-1	(10)	1-2/1, 1/0-1	(6)	1-2/1, 1/0-1	(6)
Genu I–IV	2-3/2, 2/1-2	(12)	2-3/1, 2/**0**-2	(**10**)	2-2/1, 2/**0**-1	(**8**)	2-2/1, 3/0-1	(9)
Tibia I–IV	2-3/2, 2/1-2	(12)	2-2/1, 2/1-2	(10)	2-1/1, 2/1-1	(8)	2-2/1, 2/1-1	(9)
***Epicriopsis mirabilis***								
Femur I–IV	2-3/1, 2/2-2	(12)	2-2/1, 2/2-1	(10)	1-2/1, 1/0-1	(6)	1-2/1, 1/0-1	(6)
Genu I–IV	2-3/**1**, 2/1-2	(**11**)*	2-3/1, 2/1-2	(11)	2-2/1, 2/1-1	(9)	2-2/1, 3/0-1	(9)
Tibia I–IV	2-3/**1**, 2/1-2	(**11**)*	2-2/1, 2/1-2	(10)	2-1/1, 2/1-1	(8)	2-2/1, 2/1-1	(9)
***Epicriopsis palustris***								
Femur I–IV	2-3/1, 2/2-2	(12)	2-2/1, 2/2-1	(10)	1-2/1, 1/0-1	(6)	1-2/1, 1/0-1	(6)
Genu I–IV	2-3/2, 2/1-2	(12)	2-3/1, 2/1-2	(11)	2-2/1, 2/**0**-1	(**8**)	2-2/1, 3/0-1	(9)
Tibia I–IV	2-3/2, 2/1-2	(12)	2-2/1, 2/1-2	(10)	2-1/1, 2/1-1	(8)	2-2/1, 2/1-1	(9)
***Epicriopsis suedusEpicriopsis walteri*** (after Halliday, 1997)								
Femur I–IV	2-3/1, 2/2-2	(12)	2-2/1, 2/2-1	(10)	1-2/1, 1/0-1	(6)	1-2/1, 1/0-1	(6)
Genu I–IV	2-3/2, 2/1-2	(12)	2-3/1, 2/1-2	(11)	2-2/1, 2/1-1	(9)	2-2/1, 3/0-1	(9)
Tibia I–IV	2-3/2, 2/1-2	(12)	2-2/1, 2/1-2	(10)	2-1/1, 2/1-1	(8)	2-2/1, 2/1-1	(9)

**Table 3.** Differences in leg chaetotaxy in species of *Epicriopsis* with known leg chaetotaxy [based on Halliday (1997)*, Narita and Moraes (2016)**, and own observations].

Species / Leg segment	Tibia I	Genu I	Genu II	Genu III	Genu IV
E. atuberculatus**	av2 present	av2 present	pv1 absent	pv1 absent pd3 present	pd3 present
*E. horridus*	av2 present	av2 present	pv1 absent	pv1 absent pd3 absent	pd3 absent
*E. hungaricus*	av2 present	av2 present	pv1 absent	pv1 absent pd3 absent	pd3 present
*E. mirabilis*	av2 absent	av2 absent	pv1 present	pv1 present pd3 absent	pd3 present
*E. palustris*	av2 present	av2 present	pv1 present	pv1 absent pd3 absent	pd3 present
*E. suedus* E. walteri*	av2 present	av2 present	pv1 present	pv1 present pd3 absent	pd3 present

## On ecology and habitat preference of Slovak Ameroseiidae

According to specific microhabitat requirements, the Slovak species of Ameroseiidae can be classified into the following ecological groups:

(1) Edaphic species (eight species, 29.6% of the total). This group includes all representatives of *Epicriopsis*, and several species of *Ameroseius*: *Ameroseius cavernosus*, *Ameroseius corbiculus*, *Ameroseius lidiae* and *Ameroseius sculptilis*. With the exception of *A. cavernosus*, *A. corbiculus* and *Epicriopsis horridus*, all of these representatives are apparently hygrophilous, inhabiting permanently moist places of wetlands or periodically flooded areas like littoral reed stands of shores, bogs, flood plains, riverine inundation zones and swamps (they can apparently survive in soaked substrates). Edaphic ameroseiids are bound to various kinds of soil substrates, such as highly humic soils, raw humus, decomposing plant substances, leaf and needle litter, moss, etc. *Epicriopsis horridus* is the only Slovak *Epicriopsis* species that can be considered as mesohygrophilous. It colonises also relatively drier but often umbriferous woodland stands. *Epicriopsis* species occur mostly in lowlands, only *Epicriopsis mirabilis* colonises cold mountaineous areas. *Ameroseius corbiculus* is the most common and most widely distributed ameroseiid species in Slovakia, although not in cold stands in montane zone. It occurs in various microhabitats but it prefers substrates with high content of raw humus and decaying plant parts, mainly in lowlands and low highlands, at altitudes with an optimum up to 600 m. It can also penetrate into cultivated landscape habitats (orchards, gardens and degraded stands). *A. cavernosus* seems to be the only xerotolerant species exclusively found in relatively warmer and drier localities of the southern part of Slovakia.

(2) Saproxilic species (eight species, 29.6% of the total). They are adapted to living in rotting wood with growth of wood-inhabiting fungi, including sporocarps of wood-destroying fungi, snags, stumps, stems, subcortical area of dying trees, and galleries of xylophagous insects. This group includes several *Ameroseius* species, namely *Ameroseius callosus*, *Ameroseius corniculus*, *Ameroseius fungicola*, *Ameroseius furcatus*, *Ameroseius georgei*, *Ameroseius longitrichus*, *Ameroseius renatae* sp. n. and *Ameroseius ulmi*. Interesting cases of saproxilic detriticoles appear to be the strictly specialised mycetobionts *A. callosus* and *A. fungicola* exclusively associated with the fruiting bodies of polypore shelf fungi (Holobasidiomycetidae, Polyporales), permanently dwelling fungal sporocarps.

(3) Saprophilous species (two species, 7.4% of the total). They include species showing affinity to strongly decaying organic matter in dunghills, excrements and compost heaps. They facultatively occur in other substrates consisting of a portion of excrements or decomposing plant and animal substances, such as manured arable soils, bird nests, heterogeneous organic refuses, etc. This ecological group includes only *Ameroseiella macrochelae* and *Kleemannia tenella*.

(4) Aerial species (eight species, 29.6% of the total). Especially in species with foliately expanded dorsal setae, spreading by air currents (passive air-borne dispersal) seems to be a very important part of the life history strategy of these mites (real evidence for this phenomenon has not been confirmed up to now), and ensures the continued existence of species that occur in ephemeral and scattered habitats such as mould growths living on diverse organic substrates. A relatively larger group of species that colonise manifold natural and synanthropic microhabitats such as interior of human buildings, cellars, farms, stored food, granaries, dumping grounds, haystacks, vertebrate nests and agrocoenoses. I incorporate here several species as follows: *Kleemannia delicata*, *Kleemannia insignis*, *Kleemannia pavida*, *Kleemannia plumea*, *Kleemannia plumigera*, *Kleemannia plumosa*, *Kleemannia pseudoplumosa* and *Sinoseius lobatus*. Occasionally, some aerial species can be found in caves. For example, *Kleemannia plumigera* and *Sinoseius lobatus* were found in the guano, baited straw or soil detritus in Slovakian caves.

(5) Insecticolous species (one species, 3.7% of the total). This ecological group includes species with well-developed phoretic activity and that spend all or part of their life cycle on a host insect or in its nest. *Neocypholaelaps favus*, as the only representative of this group in Slovak fauna, lives in association with European honey bee *Apis mellifera* Linnaeus, 1758 and its nests (hives), and feeds probably on pollen of flowers visited by their bees carriers.

## Catalogue of world species of Ameroseiidae

I present below an alphabetic list of all genera and lower taxa of Ameroseiidae that I am aware of, together with original identification keys to world genera and the species occurring in Europe. For each species (except three newly described species), the following data are provided: (1) current generic combination of the species, author(s) and year of the original description, (2) name of the species in its original combination, with reference to the author, year and the page on which the original description begins, (3) references to subsequent literature on the species, including different names used in diverse systematic concepts, (4) synonyms if any, each followed by its author, year and page number on which the description of the synonymised species begins, and the reference in which the synonymy is established, (5) extra information on synonymy and other nomenclatural problems where necessary, (6) type depository, name of the institution where the type material is deposited, (7) type locality and habitat from which the name-bearing type specimens are collected, (8) comparative material, type or non-type specimens which were available for comparative purpose of this study, (9) material from Slovakia, references to the older faunistic records, together with complete collection data on revised and newly reported specimens if found in Slovakia, (10) occasional remarks are provided to explain some complicated taxonomic or morphological problems. There are also similar data provided for each genus (see previous points 2, 4, 5 and 10), including newly elaborated morphological diagnosis for each genus.

Some species are not included in the genus classification because they are not described in enough detail, or unavailable for examination, and these are listed separately, after the presentation of the valid species, as (1) unrecognizable species (*species inquirendae*), by the lack sufficient information about important characters needful for their specific identification, (2) species excluded from Ameroseiidae that have been placed in *Ameroseius* in the past, but are now placed in the Ascidae, Blattisociidae, Phytoseiidae and Ologamasidae, (3) *nomina nuda*, names of the species which were apparently never described in any of published papers, and unavailable according to the International Code of Zoological Nomenclature, and (4) other unavailable names.

### Family Ameroseiidae

Taxon classificationAnimaliaMesostigmataAmeroseiidae

Evans, 1961

Ameroseiidae Evans (in Hughes, 1961: 244). Type genus: Ameroseius Berlese, 1904, by inference.

#### Diagnosis

**(adults)**. Idiosoma oblong and dorsoventrally flattened. Dorsal shield entire, holotrichous, having at most 30 pairs of setae; setae J1 and J5 absent. Sternal region with separate sternal and epigynal shields in female, sternitogenital shield in male, and five pairs of sternal setae in both sexes (st1–st5). Sternal shield reduced in size, but entire, with 1–3 pairs of sternal setae (normally two pairs); presternal platelets absent. Metasternal setae (st4) on soft integument or endopodal platelets. Epigynal shield with a pair of genital setae (st5); genital opening of male with presternal position. Peritremes well developed, long. Posterior ventral surface of adults with anal or ventrianal shield, and with at least four pairs of opisthogastric setae. Three circum-anal setae present. Cheliceral digits short; arthrodial membrane at base of movable digit smooth, without brush-like or filamentous processes. Palptarsal apotele with two or three tines. Hypostome with internal malae usually unmodified and lightly fringed. Tibia I with five dorsal setae; legs II of males without spur-like structures; tarsus II in female usually without enlarged spine-like distal setae; genu IV with normally nine setae, including one ventral (but aberrations exist). Mites with podospermal insemination, spermatodactyl of male present and placed on movable digit of chelicera. Female monogynaspid.

#### Key to world genera of Ameroseiidae (females)

**Table:** 

1	Ventrianal shield with three pairs of opisthogastric setae (JV1, JV2, ZV2) and three circum-anal setae; endopodal platelets II-III and III-IV connected, bearing metasternal setae (st4); genua II and III without ventral setae	***Brontispalaelaps* Womersley, 1956**
–	Ventrianal shield absent or with at most two pairs of opisthogastric setae (JV2, ZV2) and three circum-anal setae; endopodal platelets II-III and III-IV separate or absent, setae st4 on soft integument; genua II and III each with at least one ventral seta	**2**
2	Dorsal setae simple, short, mostly smooth and pointed, needle-shaped; peritrematal shield reduced, not reaching the anterior tip of peritreme; peritrematal shields or peritremes not connected to dorsal shield; ambulacra of legs usually without claws	**3**
–	At least some of dorsal setae modified, expanded, thickened or flattened, often elongate and pilose, with acute or obtuse apex, lance-shaped or leaf-shaped; peritrematal shield developed along whole length of peritreme; anterior section of peritrematal shield wide and completely fused to dorsal shield; ambulacral claws in legs II-IV present or absent	**4**
3	Dorsal shield with 29 pairs of setae, dorsolateral soft integument besides the shield without setae; insemination ducts fused and entering sacculus foeminus by a common neck-like process of the sacculus before entering spermatheca	***Afrocypholaelaps* Elsen, 1972**
–	Dorsal shield with 18–25 pairs of setae, dorsolateral soft integument besides the shield with 3–20 pairs of setae; insemination ducts unfused and entering sacculus foeminus separately	***Hattena* Domrow, 1963**
4	Genu III with nine setae, including one posterolateral seta (2-2/1, 2/1-1)	**5**
–	Genu III with ten setae, including two posterolateral setae (2-2/1, 2/1-2)	**10**
5	Posterior venter with four pairs of setae on soft integument (JV1, JV5) and on ventrianal shield (JV2, ZV2), JV3 and JV4 absent; dorsal shield with 21 pairs of setae	***Pseudoameroseius* gen. n.**
–	Posterior venter with five or six pairs of setae (JV1–JV5, ZV2; JV4 sometimes absent); ventrianal shield (if present) with at most one pair of opisthogastric setae (JV3); dorsal shield with 22–30 pairs of setae	**6**
6	Dorsal shield ornamented with tubercles, or spines arranged in rows, bearing 22–28 pairs of setae; some dorsal shield setae (6–9 pairs) conspicuously thickened and greatly lengthened	***Epicriopsis* Berlese, 1916**
–	Dorsal shield otherwise sculptured, never with tubercles or rows of spines in medial surface, bearing 28–30 pairs of setae; dorsal shield setae usually subequal or less differing in length	**7**
7	Sternal shield with three pairs of setae (st1–st3); genu IV with ten setae, including two ventral setae (2-2/1, 3/1-1); dorsal setae pinnate	***Sinoseius* Bai & Gu, 1995**
–	Sternal shield with two pairs of setae (st1, st2); genu IV with at most nine setae, including one ventral seta (2-2/1, 3/0-1); dorsal setae usually otherwise formed	**8**
8	Some dorsal shield setae (6–7 pairs) inserted on distinct protuberances, these setae longer, thicker and more heavily pilose than those on flat surface; tibia IV with ten setae, including two posterolateral setae (2-2/1, 2/1-2); fixed digit of chelicera slender, straight, with three sharp teeth and widened bilobed tooth close to terminal hook (Plate 77F)	***Asperolaelaps* Womersley, 1956**
–	Dorsal shield setae not inserted on protuberances; tibia IV with nine setae, including one posterolateral seta (2-2/1, 2/1-1); fixed digit of chelicera more robust, at least slightly curved, without subterminal bilobed tooth	**9**
9	Cheliceral digits slender and terminally hooked: fixed digit edentate or with one weak tooth, having hyaline lobed appendage; corniculi slender and convergent, with undivided and pointed apex	***Neocypholaelaps* Vitzthum, 1942**
–	Cheliceral digits robust and straight: fixed digit well dentate, normally with three teeth on proximal masticatory area (rarely with one robust tooth or two denticles), never with hyaline lobed appendage; corniculi stouter and parallel, with bi- or trifid apex	***Ameroseius* Berlese, 1904**
10	Tarsus I without ambulacrum and claws, bearing greatly lengthened sensory setae; posterior venter with anal shield bearing only three circum-anal setae	***Ameroseiella* Bregetova, 1977**
–	Tarsus I with well developed ambulacrum, claws present or absent; posterior venter with ventrianal shield bearing normally two pairs of opisthogastric setae (JV2, JV3) and three circum-anal setae	**11**
11	Fixed digit of chelicera with three proximal denticles; ambulacral claws on legs reduced or absent; epistome with bifurcate apex; dorsal setae relatively short, leaf-shaped, oblanceolate, spatulate or obovate, with rounded apex; anterior hypostomal setae (h1) thickened, curved and attenuate medially, subfalcate; anterior margin of coxa II produced into spiniform process	***Sertitympanum* Elsen & Whitaker, 1985**
–	Fixed digit of chelicera with four proximal denticles; ambulacral claws on legs present; epistome with undivided apex; dorsal setae relatively longer, tubiform or leaf-shaped, with pointed apex; setae h1 thin or thickened, almost straight and regularly tapered; anterior margin of coxa II smooth	***Kleemannia* Oudemans, 1930**

### Genus Afrocypholaelaps

Taxon classificationAnimaliaMesostigmataAmeroseiidae

Elsen, 1972

Afrocypholaelaps Elsen, 1972b: 159. Type species: Neocypholaelaps africana Evans, 1963a, by original designation.

#### Diagnosis (female).

Dorsal shield weakly sclerotised, smooth medially and reticulate laterally, oblong, with 29 pairs of subequal setae. Dorsal setae including j1 short and needle-like, mostly smooth, sometimes delicately pilose to serrate. Setae st1 and st2 on sternal shield, st3 on small pseudo-metasternal platelets and st4 on soft integument. Sternal and epigynal shield relatively long and narrow, smooth and unornamented on surface. Epigynal shield with anterior hyaline portion produced into a prominent cusp reaching the level of st2; genital poroids on soft integument, outside the shield. Metapodal platelets absent. Anal shield relatively small, subcircular, bearing three circum-anal setae. Peritrematal shields or peritremes with anterior ends free, not fused to dorsal shield. Opisthogastric soft integument with six pairs of setae (JV1–JV5, ZV2). Soft striate integument smooth, not incrusted with sclerotic denticles or tubercles. Corniculi slender and convergent, surrounded by hyaline membranes, with undivided and pointed apex. Fixed digit of chelicera unidentate, having hyaline lobed appendage; movable digit edentate, well hooked distally. Epistome with curved and denticulate anterior margin. Palptarsal apotele two-tined. Femur II with two ventral setae, genua II–III and tibiae II–III each with one anterolateral and one posterolateral seta. Tarsi I–IV each with well developed empodium but reduced claws (the claws normal in males and developmental stages). Insemination apparatus with spermathecal ducts entering *sacculus foemineus* through a common neck-like process of the *sacculus*.

#### Remark.

The genus *Afrocypholaelaps* is distributed in the tropical and subtropical areas of the Old World and Oceania (Australia, Papua New Guinea, Hawaii Islands, Japan, Taiwan, Saint Helena Island, Angola and Madagascar). Mites of this monobasic genus live on flowers of various plants where they probably feed on pollen and nectar. Phoresy of females is reported from European honey bee (*Apis mellifera*), bees and wasps of various apoid genera (*Meliponula*, *Ceratina*, *Hylaeus* and *Chlorion*), and other flower-visiting insects (Lepidoptera).

### Afrocypholaelaps africanus

Taxon classificationAnimaliaMesostigmataAmeroseiidae

(Evans, 1963)

Hypoaspis sp. — Simmonds 1949: 42. By Halliday 1997.
Neocypholaelaps sp. — Brimblecombe and Roff 1960: 447. By Halliday 1997.
Neocypholaelaps africana Evans, 1963a: 224.
Neocypholaelaps lindquisti Prasad, 1968: 130. **Syn. n.**
Afrocypholaelaps africana . — Elsen, 1972b: 159; Seeman and Walter 1995: 45; Halliday 1997: 181; Ho et al. 2010: 90.
Afrocypholaelaps lindquisti . — Haitlinger 1987a: 366; Halliday 1997: 181.
Neocypholaelaps africanus . — Domrow 1979: 105.
Neocypholaelaps africana . — Ishikawa 1979: 115; Baker and Delfinado-Baker 1985: 232; Delfinado-Baker et al. 1989: 610; Karg 1993: 233.
Neocypholaelaps lindquisti . — Treat 1975: 118; Baker and Delfinado-Baker 1985: 232.
Afrocypholaelaps ranomafanaensis Haitlinger, 1987b: 531. **Syn. n.**
Afrocypholaelaps ranomafanaensis . — Halliday 1997: 181; Ho et al. 2010: 90; Narita et al. 2013a: 13.
Afrocypholaelaps lindqusti (sic). — Ho et al. 2010: 91.
Afrocypholaelaps analicullus Ho, Ma, Wang & Severinghaus, 2010: 88. **Syn. n.**

#### Type depository.

Of *Neocypholaelaps africana* – British Museum (Natural History), London, United Kingdom; of *Neocypholaelaps lindquisti* – Bernice Pauahi Bishop Museum (Hawaii State Museum of Natural and Cultural History), Honolulu, Hawaii, USA; British Museum (Natural History), London, United Kingdom; Canadian National Collection of Insects, Arachnids and Nematodes, Ottawa, Ontario, Canada; Institute of Acarology, Wooster, Ohio, USA; United States National Museum, Washington, D.C., USA; Entomology Department, University of Hawaii, Honolulu, Hawaii, USA; Zoological Survey of India, Calcutta, India; of *Afrocypholaelaps ranomafanaensis* – Museum of Natural History, Wrocław University, Poland; of *Afrocypholaelaps analicullus* – National Museum of Natural Science, Taichung, Taiwan.

#### Type locality and habitat.

Of *Neocypholaelaps africana* – Angola, Luanda, on an African stingless bee, *Meliponula bocandei* (as *Trigona tomentosa*) (Hymenoptera); of *Neocypholaelaps lindquisti* – Hawaii, Manoa, Oahu, on a noctuid moth, *Achaea janata* (Lepidoptera); of *Afrocypholaelaps ranomafanaensis* – Madagascar, Ranomafana, on unidentified butterfly (Lepidoptera); of *Afrocypholaelaps analicullus* – Taiwan, Chiayi, Fenchihu, on European honey bee, *Apis mellifera* (Hymenoptera).

#### Comparative material.

Madagascar: 1 ♀ (MPUV: MP-1291, holotype by monotypy) – 19. 3. 1986, Ranomafana, Lepidoptera (labelled *Afrocypholaelaps ranomafanae*, holotype).

#### Remarks.

**Figure 2.**
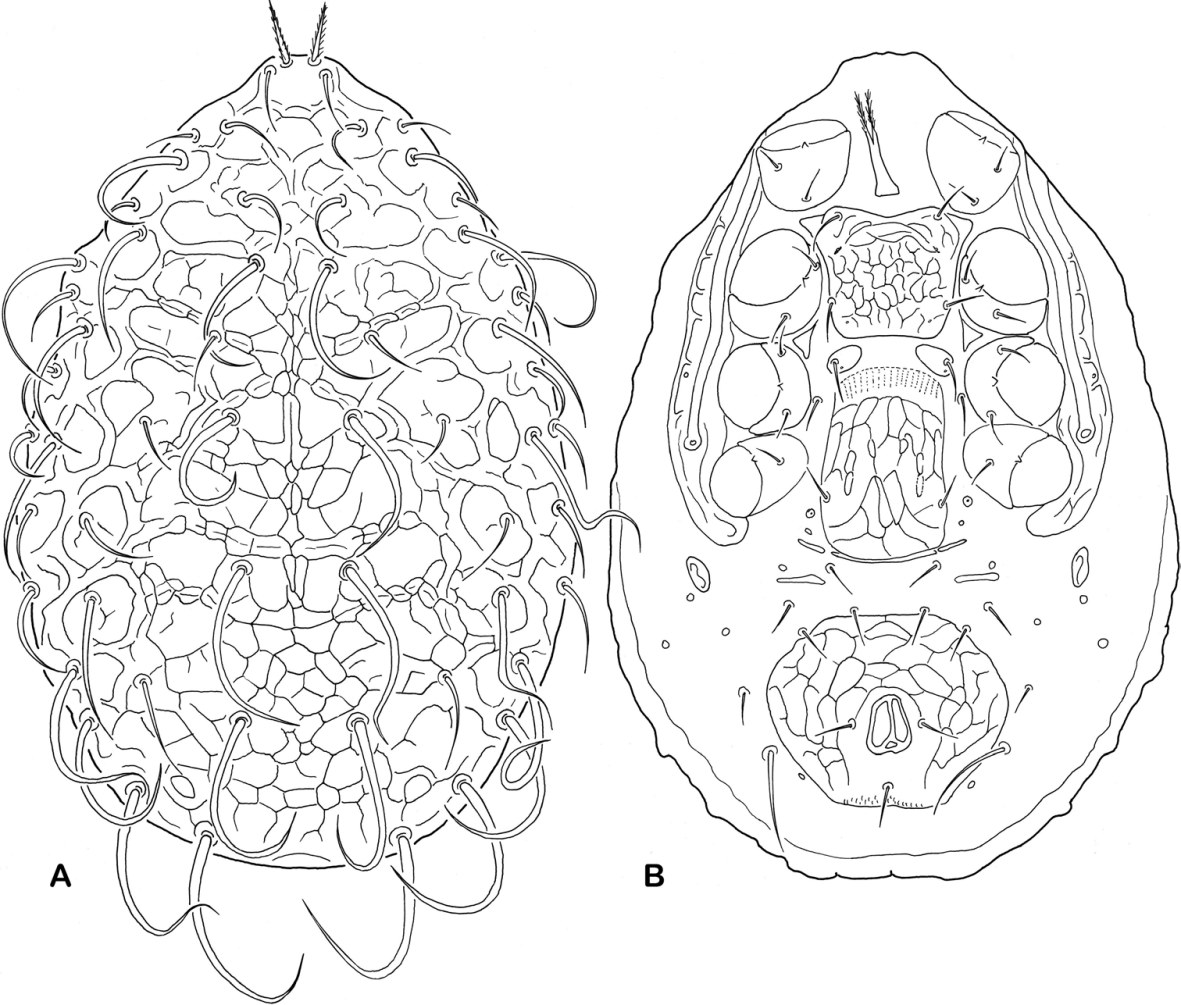
*Ameroseius renatae* sp. n., female. **A** Dorsal idiosoma **B** Ventral idiosoma. Not scaled.

**Figure 3.**
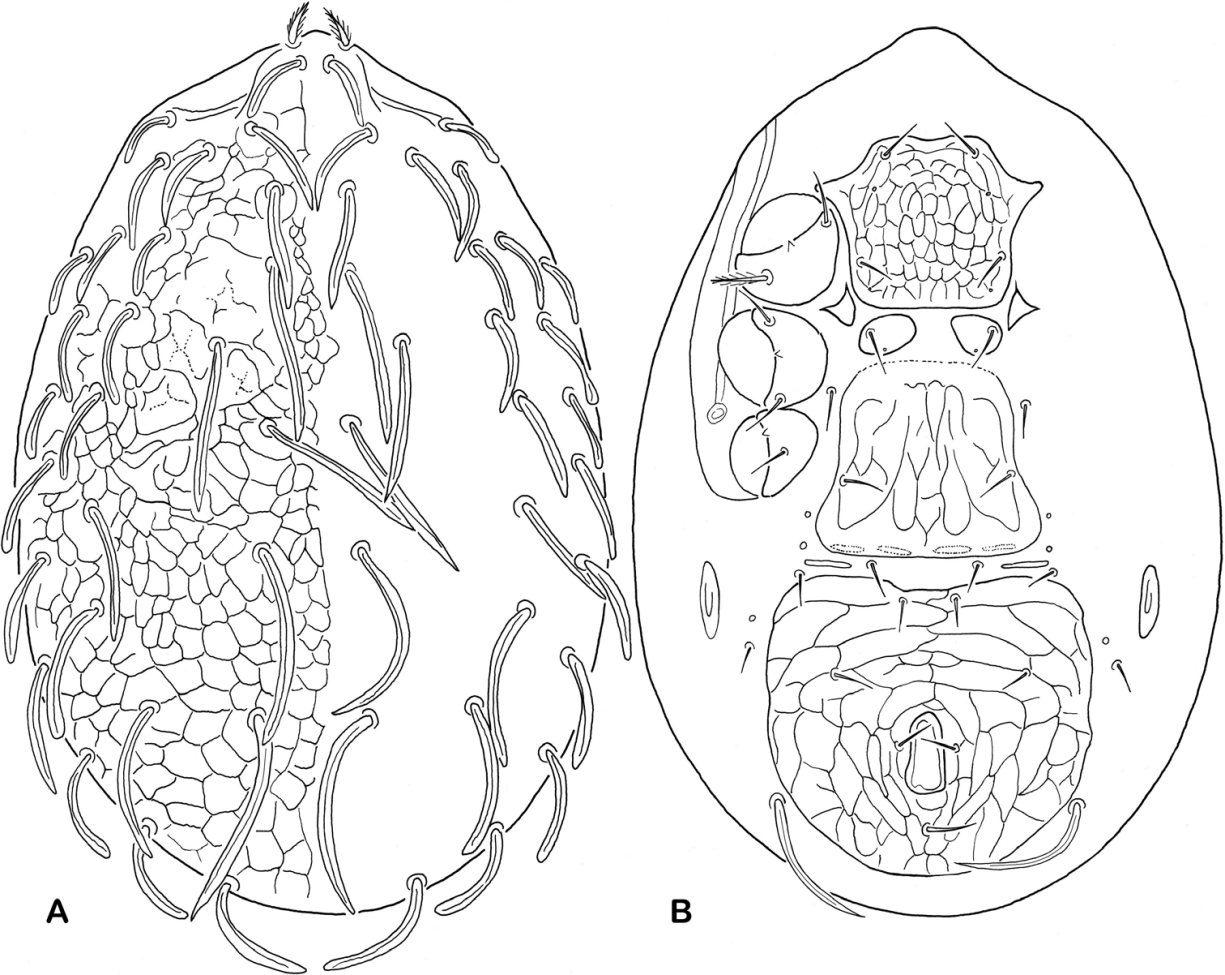
*Kleemannia dolichochaeta* sp. n., female. **A** Dorsal idiosoma **B** Ventral idiosoma. Not scaled.

**Figure 4.**
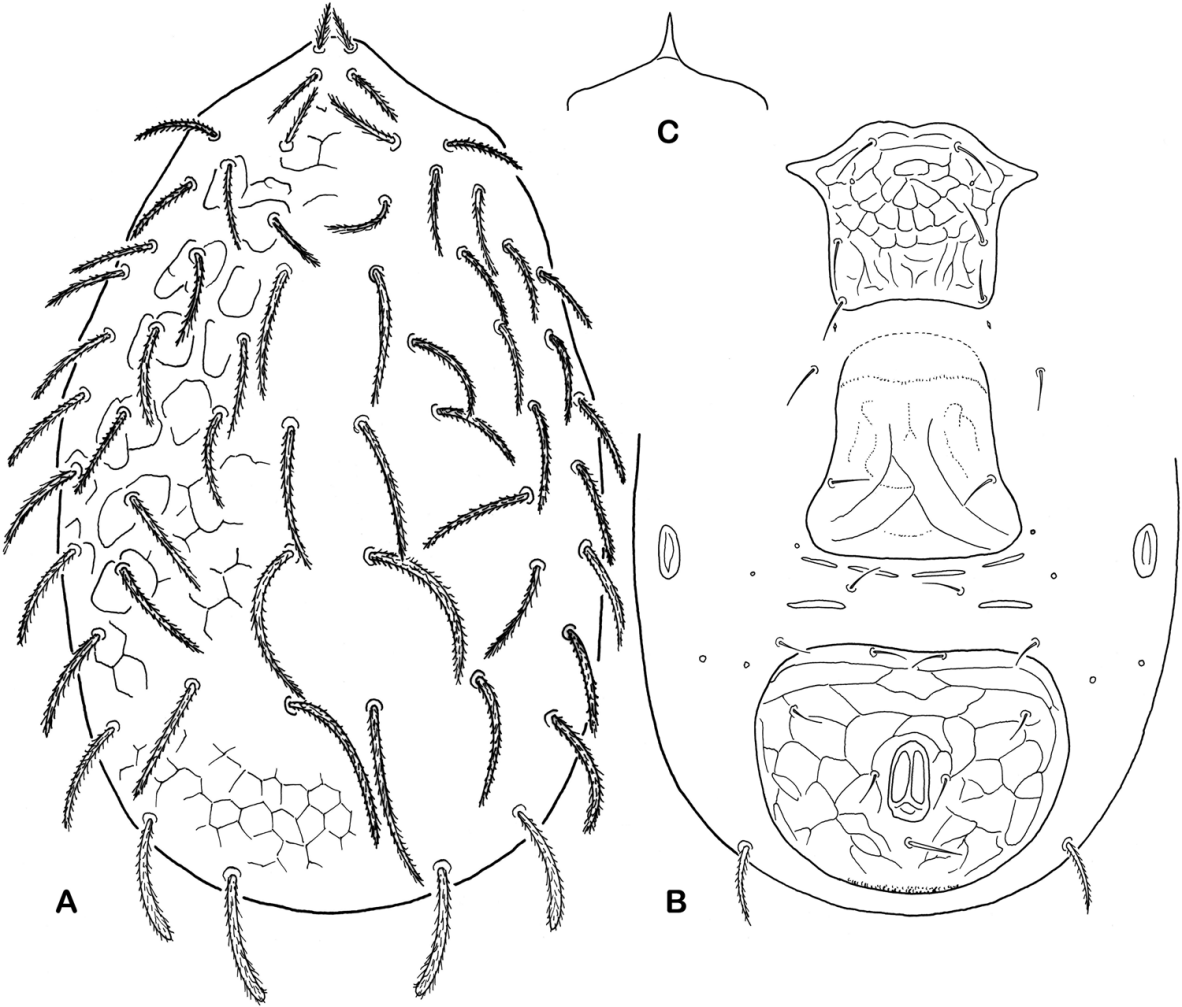
*Kleemannia miranda* sp. n., female. **A** Dorsal idiosoma **B** Ventral idiosoma. Not scaled.

There are some contradictory statements on the leg chaetotaxy of this species in some available papers on the genus *Afrocypholaelaps*. The leg chaetotaxy for *Afrocypholaelaps africanus* in Evans (1963a) is incomplete. His figures (1–4, page 211) show the chaetotaxy of *Neocypholaelaps*, with three ventral setae on femur II, and two anterolateral setae on genu II, tibia II, genu III and tibia III. He then mentioned that *A. africanus* has only one anterolateral seta on genu III and tibia III (page 213, and 225). He did not mention the deficiency of a ventral seta on femur II, and one anterolateral seta on genu II and tibia II. Prasad (1968) misunderstood this. He thought *A. africanus* had three ventral setae on femur II and two anterolateral setae on genu II and tibia II. That is why he thought *A. africanus* had more leg setae than the species he then described as *Afrocypholaelaps lindquisti*. These two species actually have the same chaetotaxy, and they are here considered to be conspecific. Later, in his original generic diagnosis of *Afrocypholaelaps*, Elsen (1972b) re-examined the type species *A. africanus*, stating the correct number of two ventral setae on femur II, and one anterolateral and one posterolateral seta on genu II and tibia II. This chaetotactic pattern was confirmed also by Halliday (personal communication), who additionally checked his Australian specimens of *A. africanus*, although his previous chaetotactic data (Halliday 1997) showed the same misinterpretation of Prasad (1968) and Ho et al. (2010).

*Afrocypholaelaps ranomafanaensis* was quite briefly and inadequately described on the basis of the single female specimen by Haitlinger (1987b), and his description needs various amendments because it contains several morphological features inconsistent with *Afrocypholaelaps* (dorsal shield with 28 instead of 29 pairs of setae, soft integument with four instead of six pairs of opisthogastric setae, st3 on soft integument instead of on small shields). The only further differences to the other congeners of *Afrocypholaelaps* that he noted were the relatively larger dorsal shield (488 × 280 vs 350–436 × 216–279 μm respectively) and the shields situated on ventral surface (sternal shield 120 × 90 vs 68–84 × 68–78 μm, epigynal shield 134 × 86 vs 100–173 × 53–60 μm, and anal shield 84 × 92 vs 52–69 × 62–78 μm) in his own specimen from Madagascar. I examined the holotype specimens of *A. ranomafanaensis* and found no important morphological differences with specimens described by various authors under the name *Afrocypholaelaps* (Evans 1963a, Prasad 1968, Elsen 1972b, Domrow 1979, Ishikawa 1979, Seeman and Walter 1995, Halliday 1997, and Ho et al. 2010). Despite the fact that the holotype is not in perfect condition for observation, I could detect full complement of 29 pairs of dorsal shield setae typical of *Afrocypholaelaps*. Setae s1 and s2 are asymmetrically expressed in right side of the dorsal shield, as their bases are markedly adjacent each other. Dorsal setae are quite short and similar in length (anterior and marginal dorsal setae 12–14 μm, posteriormost dorsal setae 10–12 μm, Z5 11 μm), with otherwise position and relative length as in original illustration. Posteroventral region possesses the complete set of four pairs of slightly asymmetrically situated opisthogastric setae (JV1–JV3, ZV2) of which left JV2 displaced somewhat posteriorly and left JV3 abnormally placed on anal shield. Remaining two pairs of opisthogastric setae can be found on dorsal marginal surface, but I have detected only two of them (JV4, JV5) on the left side of the holotype. Ventrally placed setae clearly longer than those on dorsum (JV1 27–29 μm; JV2, JV3 and ZV2 16–23 μm). Setae st3 inserted on very small suboval pseudo-metasternal platelets. Anal shield not regularly rounded, but with posterior portion slightly expanded. I have checked the size of the all above mentioned shields, and can report holotype specimen with lower values for these measurements: dorsal shield 488 × 280 vs 428 × 248 μm respectively, sternal shield 120 × 90 vs 74 × 77,5 μm (at the level of st2), width of epigynal shield 86 vs 80 or 69 (at the level of genital setae), and anal shield 84 × 92 vs 78 × 89 μm. I believe that slight differences in size of scutal structures and setal length, found in different world populations of *Afrocypholaelaps* and documented by various above cited authors can be interpreted as variation within a widespread species. Seeman and Walter (1995) examined the holotype and paratype of *Afrocypholaelaps africanus* and a rich material of the specimens from Australia, finding no morphological differences between them. I believe that also *A. ranomafanaensis* and *A. africanus* cannot be distinguished morphologically, and are considered here to be conspecific.

*Afrocypholaelaps analicullus* was characterised by the features of hypostome, anal shield and adjacent soft integument (Ho et al. 2010), and introduced as a species closely related with *Afrocypholaelaps lindquisti*. The most important diagnostic characters of *A. analicullus* were mentioned to be (1) setae h2 and h3 similar in length; (2) ventral hypostome with five short rows of denticles on each side; (3) anal shield expanded posteriorly, with V-shaped cribrum; (4) setae JV4 and JV5 inserted close to the anal shield; in *A. lindquisti*, these characteristics were mentioned as (1) setae h2 longer than h3; (2) ventral hypostome with one short and two long rows of denticles; (3) anal shield rounded, with U-shaped cribrum; (4) setae JV4 and JV5 distant from the anal shield. Undoubtedly, Ho’s interpretation of *A. lindquisti* was based on the original descriptions and illustrations, and not on study of the type material. The above enumerated distinguishing characters do not provide a useful basis for establishment of a new species, so I relegate *A. analicullus* into synonymy with *Afrocypholaelaps africanus*. For example, the position of JV4 and JV5, and their distance from the anal shield, depends on an expansion of soft integument. The soft integument is well striated in *Afrocypholaelaps*, and it could conceivably increase its surface considerably in gravid females, as it is originally depicted in *A. lindquisti* by Prasad (1968). Also anal shield may vary in size and shape, as documented in *A. africanus* by illustrations of Evans (1963a) and Ishikawa (1979). Except for the leg chaetotaxy, the only reliable difference between *A. africanus* and other two species, *A. lindquisti* and *A. analicullus*, appeared the number of serrated setae on the posteriormost surface of the dorsal shield. Evans (1963a) stated three pairs of serrate setae in *A. africanus*, whereas Prasad (1968) and Ho et al. (2010) reported six pairs of such setae for *A. lindquisti* and *A. analicullus*, respectively. But according to Elsen (1972b), the former species actually has the same number of serrate posterior setae as the two latter ones.

### Genus Ameroseiella

Taxon classificationAnimaliaMesostigmataAmeroseiidae

Bregetova, 1977

Ameroseiella Bregetova, 1977: 167. Type species: Ameroseius apodius Karg, 1971 (= Ameroseius macrochelae Westerboer, 1963; new synonymy), by original designation.

#### Diagnosis (adults).

Dorsal shield well sclerotised and coarsely ornamented, with 29 pairs of setae, including z6, similar in length and form, thickened, often flattened, with longitudinal vein and smooth or sparsely serrate lateral margins; setae j1 differently formed, leaf-like to fan-shaped, with distinctive longitudinal midrib and regularly denticulate anterolateral margin. In female, sternal setae on sternal shield (st1, st2), soft integument (st3, st4) and epigynal shield (st5); opisthogastric region with six pairs of setae (JV1–JV5, ZV2), all on soft integument (anal shield with only three circum-anal setae). In male, ventrianal shield with 3–4 pairs of opisthogastric setae (JV1 on or off the shield, JV5 always off the shield); setae JV4 absent. Opisthogastric setae mostly smooth, short and needle-like; setae JV4 pilose; setae JV5 modified and similar to those on dorsal shield. Corniculi normally sclerotised, relatively slender, with splitted apex; setae h1 markedly thickened. Cheliceral digits relatively large, fixed digit of chelicera with three well developed teeth on proximal masticatory area (two proximal teeth somewhat adjacent). Epistome with anterior margin produced into long and narrow central projection. Palptarsal apotele two-tined. Genu III, and tibiae III–IV with two anterolateral and two posterolateral setae. Tarsi of legs I with empodium and claws not developed, terminating with four conspicuously lengthened sensory setae and some shorter ones; tarsi II–IV each with normal empodium and claws.

#### Remarks.

The genus *Ameroseiella* Bregetova, 1977 was originally diagnosed from other ameroseiid genera by the absence of pretarsal empodium and claws on legs I. This genus was considered as a synonym of *Ameroseius* Berlese, 1904 (Karg 1993, 2005). In the meantime, Evans and Till (1979) and Halliday (1997) accepted *Ameroseiella* as a distinct genus, and their concept is adopted in the present paper because the genus exhibits some character states presented by *Kleemannia* and others by *Ameroseius*. For example, the presence of anal shield and three teeth on fixed cheliceral digit is not consistent with *Kleemannia*, whereas the presence of two posterolateral setae on genu III and tibiae III–IV, pointed epistome, thickened h1 are the features not consistent with those found in *Ameroseius*.

The concept of *Ameroseiella* is based especially on the following combination of characters: (1) leg I without ambulacrum and terminating in lengthened setae; (2) dorsal shield with 29 pairs of setae; (3) setae j1 fan-shaped and with longitudinal midrib; (4) in female, st3 on soft integument; (5) in female, anal shield only with three circum-anal setae; (6) setae JV5 similar to those on dorsum; (7) cheliceral digits robust, fixed digit with three large teeth; (8) epistome with pointed medial process; (9) setae h1 thickened; (10) palptarsal apotele two-tined.

*Ameroseiella* is distributed exclusively throughout the Palaearctic region, and it currently comprises two species reported from Europe and Asia. They occur in various decomposing organic materials like compost and dung, and in the nests of mammals and birds (Westerboer and Bernhard 1963, Karg 1971a, Bregetova 1977).

#### Key to species of *Ameroseiella* occurring in Europe (adults)

**Table:** 

1	Dorsocentral setae relatively narrow, with lateral margins parallel and sparsely serrate; postanal seta thickened, densely pilose, almost brush-shaped	***Ameroseiella macrochelae* (Westerboer, 1963)** (Plates 1, 2)
–	Dorsocentral setae flattened, foliately expanded in medial portion, with smooth lateral margins; postanal seta thin, smooth and needle-shaped	***Ameroseiella stepposa* Bregetova, 1977**

### Ameroseiella macrochelae

Taxon classificationAnimaliaMesostigmataAmeroseiidae

(Westerboer, 1963) comb. n.

Plates 1 , 2

Ameroseius macrochelae Westerboer (in Westerboer & Bernhard, 1963: 501).
Ameroseius apodius Karg, 1971a: 226. **Syn. n.**
Ameroseius macrochelae . — Karg 1971a: 230; Bregetova 1977: 161; Karg 1993: 228.
Ameroseiella apodius . — Bregetova 1977: 167.
Ameroseius apodus . — Evans and Till 1979: 195.
Ameroseiella apoda . — Evans and Till 1979: 230.
Ameroseius apodius . — Karg 1993: 226.

#### Type depository.

Of *Ameroseius macrochelae* – Zoologischen Staatssammlung München, Germany (originally not stated; holotype not designated); of *Ameroseius apodius* – Museum für Naturkunde, Berlin, Germany.

#### Type locality and habitat.

Of *Ameroseius macrochelae* – France, Nimes, in compost; of *Ameroseius apodius* – Germany, Frankfurt/Oder, Manschnow (holticulture), in compost soil.

#### Comparative material.

Germany: 1 ♀ (ZMB: 39868, holotype) – Sept. 1962, Manschnow/Oderbruch, 2981 (labelled *Ameroseius apodius*); 3 ♀♀ (ZMB: 42520) – Kleinmachnow, 1034 (labelled *Ameroseius macrochelae*). United Kingdom: 1 ♀ (BMNH) – 6. 3. 1963, Sutton Bonington, Nottingham County, oak-beech forest, „Scotland plantation“, leg. D. J. L. Harding.

#### Published material from Slovakia.

Borská Nížina Lowland: Rohožník Village (Ambros et al. 1992, cited as *Ameroseiella apodius*). Bukovské Vrchy Hills: Nová Sedlica Village, Zakasárenský Potok Brook (Fenďa et al. 1998, Fenďa and Mašán 2003; cited as *Ameroseius apodius*). Cerová Vrchovina Highland: Šurice Village (Fenďa and Mašán 2009, cited as *Ameroseius apodius*). Laborecká Vrchovina Highland: Stakčín Village, Starina Dam, Gazdoráň Forest (Fenďa et al. 1998, Fenďa and Mašán 2003; cited as *Ameroseius apodius*). Malá Fatra Mts.: Turčianske Kľačany Village, Kľačianska Magura Mt. (Kalúz and Žuffa 1988, cited as *Ameroseiella apodius*). Podunajská Rovina Flatland: Dobrohošť Village, Dunajské Kriviny Forest (Fenďa et al. 1998, cited as *Ameroseius apodius*). Gabčíkovo Town, Ercséd Forest; Gabčíkovo Town, Ostrov Orliaka Morského Forest; Vojka Nad Dunajom Village, Hajóšok Forest (Fenďa and Lengyel 2007, cited as *Ameroseius apodius*). Svätý Jur Town, Šúr Forest (Fenďa et al. 2011, cited as *Ameroseius apodius*).

#### New material from Slovakia.

borská Nížina Lowland: 1 ♀ – 2. 8. 1993, Bratislava Capital, Devínska Nová Ves Settlement, Devínske Jazero Lake, willow-poplar flood-plain forest (*Salici-Populetum*), nest of *Falco tinnunculus* (Aves), altitude 140 m, leg. J. Krištofík and A. Darolová. Malé Karpaty Mts.: 1 ♀ – 5. 5. 2004, Bratislava Capital, Patrónka Settlement, ruderal with hornbeams (*Carpinus betulus*), strongly decaying seedy sunflowers, altitude 180 m, leg. P. Mašán. Podunajská Rovina Flatland: 8 ♀♀ – 5. 4. 1989, Kráľovičove Kračany Village, nest of *Corvus frugilegus* (Aves), altitude 120 m, leg. J. Krištofík and A. Darolová; 14 ♀♀ – 26. 7. 1991, Dunajská Streda Town, nest of *Accipiter gentilis* (Aves), altitude 110 m, leg. J. Krištofík and A. Darolová; 2 ♀♀ – 12. 5. 1998, Medveďov Village, shore reed stand (*Phragmition*), strongly decaying compost (maize), altitude 120 m, leg. P. Mašán; 1 ♀ – 1. 5. 2008, Číčov Village, willow-poplar flood-plain forest (*Salici-Populetum*), nest of *Haliaeetus albicilla* (Aves), altitude 115 m, leg. J. Krištofík and A. Darolová. Trnavská Pahorkatina Highland: 2 ♀♀ – 1. 7. 2001, Horná Streda Village, garden, accumulation of dung and plant refuse, decaying organic detritus, altitude 170 m, leg. P. Mašán; 4 ♀♀ – 28. 4. 2002, Horná Streda Village, garden, accumulation of dung and plant refuse, decaying organic detritus, altitude 170 m, leg. P. Mašán; 12 ♀♀, 5 ♂♂ – 26. 5. 2005, Šenkvice Village, Šenkvický Háj Forest, oak forest (*Quercetum*), decaying straw and soil detritus, altitude 210 m, leg. P. Mašán.

#### Remarks.

*Ameroseius macrochelae* had been originally described based on specimens from France. There are three females on two slides in the Hirschmann/Willmann Collection in Munich labelled „*Ameros*. *macrochelae* n. sp.” and „*Ameroseius macrochelae* n. sp.”, all in relatively good condition. Unfortunately, none of available specimens (slides) bears a type designation and specific collection data, but without doubt the two slides belong to the original series of Westerboer because the original illustrations of the dorsum and ventrum given by Westerboer and Bernhard (1963) match perfectly one of the three available mounted specimens. All of these females lack ambulacral apparatus on legs I, being conspecific with examined holotype female described by Karg (1971a) as *Ameroseius apodius* Karg, 1971 (see comparative material above).

### Ameroseiella stepposa

Taxon classificationAnimaliaMesostigmataAmeroseiidae

Bregetova, 1977

Ameroseiella stepposa Bregetova, 1977: 167.

#### Type depository.

Zoological Institute, Russian Academy of Sciences, Saint Petersburg, Russia.

#### Type locality and habitat.

Kazakhstan, Yanvartsevo, in nest of common vole, *Microtus arvalis* (Mammalia, Rodentia).

### Genus Ameroseius

Taxon classificationAnimaliaMesostigmataAmeroseiidae

Berlese, 1904

Ameroseius Berlese, 1904: 258. Type species: Seius echinatus C. L. Koch, 1839 (= Acarus corbicula Sowerby, 1806), by original designation. Synonymy by Vitzthum (1942).
Cornubia Turk, 1943: 858. Type species: Cornubia ornata Turk, 1943 (= Acarus corbicula Sowerby, 1806), by original designation. Synonymy by Turk (1953).
Ameroseius . — Evans 1963a: 230.

#### Diagnosis (adults).

Dorsal shield strongly sclerotised and coarsely sculptured, rugose, callous or ornamented with a series of depressions or interconnecting ridges, and normally with 29 pairs of setae (in *magnisetosus* group, the shield delicately reticulated and with only 26–27 pairs of setae). Dorsal setae similar or differently formed, variously modified, thickened and lengthened, lanceolate or oblanceolate to slightly claviform (plumose, pilose, serrate or spinate on surface), rarely short, smooth and needle-like, and not sexually dimorphic (except *fungicola* group). In female, st1 and st2 on sternal shield, st3 on small pseudo-metasternal platelets or soft integument and st4 on soft integument; ventral shields usually well reticulate on surface; genital poroids outside the epigynal shield. Anal shield having three circum-anal setae in female, only rarely bearing an extra pair of opisthogastric setae (JV3) close to its anterolateral edges; male with expanded ventrianal shield having usually three pairs of opisthogastric setae: JV2, JV3, ZV2 (in *Ameroseius dendrovagans*, the shield less expanded to capture only two pairs of opisthogastric setae: JV2, JV3). Peritrematal shields with anterior ends connected to dorsal shield. Opisthogastric soft integument with five or six pairs of setae in female (JV4 sometimes absent); male never with JV4 developed, with five pairs of opisthogastric setae (JV1–JV3, JV5, ZV2). Corniculi normally horn-like, relatively broad and parallel, with splitted and pointed apex (in *fungicola* group, corniculi membranous, hyaline, undivided and directed laterally). In female, fixed digit of chelicera normally tridentate on proximal masticatory area (bidentate in *fungicola* group); movable digit edentate, at most with subapical denticle, and provided with short spermatodactyl in male. Epistome usually subtriangular, with curved apex, with smooth or denticulate anterior margin. Palptarsal apotele usually three-tined. Genu III and tibiae III–IV with two anterolateral and one posterolateral setae. Tarsi I–IV each with well developed empodium and claws. Insemination apparatus with barely discernible structures.

#### Remarks.

*Ameroseius* is the most speciose genus of Ameroseiidae. In this paper, it comprises 50 valid species having their type specimens reported from almost all continents: 13 species each from Africa and Asia, 12 species from Europe, five species each from North and South America, and two species from Australia. In Slovakia, this genus is represented by 13 recorded species. Mites of this genus are apparently fungivorous living in a wide variety of habitats such as wood substrates, wood-destroying fungi, decomposing plant material and humic soils. Most of them belong to highly specialised species, each adapted to a particular environment. There are species associated with bark beetle galleries, or wood-boring beetle galleries, in subcorticolous habitats, and feeding on a specific diet of ambrosia fungi, and phoretically active on xylophagous insects (mostly Cerambycidae). Some African species are phoretically associated with wasps and bees. In Slovakia, most species can be easily found on bracket fungi, especially on a lower fertile surface of the sporocarp (fruiting body).

Some authors (Bregetova 1977, Evans and Till 1979) attempted to clarify the general concept of *Ameroseius* by removing some species that obviously belong to other genera (*Ameroseiella*, *Kleemannia*). In this paper I refine the concept of the genus further, by establishing a new genus based on *Ameroseius michaelangeli* Moraza, 2006, removing all species that belong to *Kleemannia* and *Asperolaelaps* and that were previously placed in *Ameroseius*, and introducing two species groups based on *Ameroseius magnisetosus* and *Ameroseius fungicola* (see below). The process of clarifying the genus should continue, especially with regard to some characters inconsistent with *Ameroseius* in the species from Africa described by Elsen (1973). His African species show some atypical characters for *Ameroseius*, for example: (1) cheliceral digits distally curved, fixed digit with five proximal denticles in *Amerosieus megatritosternum*, or with only one very robust medial tooth in *Ameroseius bembix*, *Ameroseius gabonensis* and *Ameroseius leclercqi*; (2) peritremes and peritrematal shields densely spinate, especially the outer posterior margin of peritrematal shields with large spines; (3) dorsal shield setae relatively short and stout, brush-shaped; (4) anterior margin of epistome deeply dentate; (5) setae JV5 similar to those on dorsal shield, brush-shaped; (6) strong sclerotic incrustation of soft integument on opisthogastric surface. Generally, the border between *Ameroseius* and *Neocypholaelaps* is weak, based on a few diagnostic features of gnathosoma. The species of Elsen (1973) appear to have an intermediate position between these two genera because, in some respects, they exhibit certain similarities with *Neocypholaelaps*, whose members are also associated with bees and wasps (form of cheliceral digits and dorsal setae, and additional sclerotization of soft integument).

There are three Asian *Ameroseius* species (*denticulatus*, *magnisetosus* and *submagnisetosus*) representing a specific group of closely related (if not identical) congeners characterised especially by a combination of the following character states: (1) dorsum with deficient chaetotaxy, having only 26–27 pairs of setae; (2) dorsal shield lacking coarse sculpture, with only delicate reticulate pattern; (3) in female, st3 on soft integument due to the absence of pseudo-metasternal platelets; (4) in male, st4 on soft integument, outside the sternogenital shield (5) five pairs of opisthogastric setae present (JV4 absent), of which JV3 on (ventri)anal shield, and JV5 similar to other setae on ventral surface. The above enumerated species are here referred to as the *Ameroseius magnisetosus* group. Unfortunately, I have examined no representative of this peculiar group to confirm reduced number of dorsal setae and other features as stated above.

There is a combination of diagnostic characters to recognise the newly designated *Ameroseius fungicola* species group, namely (1) fixed digit of chelicera bidentate, the two teeth small and similar in size; (2) corniculi unsclerotised, membranous, hyaline, medially curved, with tapered and undivided apex directed anterolaterally; (3) conspicuous dimorphism of dorsal chaetotaxy: in males, centrally situated setae strongly reduced in length when compared with those in females; (4) male with anal shield bearing only three circum-anal setae; (5) setae z5 minute; (6) absence of postgenital slit-like sclerites; (7) anus close to anterior margin of anal shield; (8) cheliceral digits relatively small; (9) legs I relatively short and thick (especially tarsi); (10) in male, legs I and palptrochanters with some setae thicker and spiniform when compared with those in female; and (11) empodium and claws of tarsi I–IV well developed, relativelly large; (12) in males, tarsal claws of legs II apparently larger than in other legs. This group contains only two described species (*fungicola*, *callosus*), and it can be characterised by several peculiar characters (see points 1–4, 12), unique or rarely expressed in Ameroseiidae. From a phylogenetic point of view, presence of these characters might support the idea of the justified existence of a separate ameroseiid taxon based on *A. fungicola* and *A. callosus*.

Narita et al. (2015) constituted a separate species group for 21 species of *Ameroseius*, referred to as the *sculptilis* group, with the following character states: (1) dorsal shield with ridges and pit-like depressions combined with a reticulate pattern of simple lines; (2) dorsal shield with 29 pairs of mostly stout and serrate setae; (3) posterior ventral surface with five or six pairs of opisthogastric setae; (4) ventrianal shield with 0–2 pairs of setae (in addition to three circum-anal setae). However, all these character-state arguments are weak, based on greatly variant and vague features, and not suitable for correct separation of the species, even at least at the level of *Ameroseius*/*Kleemannia* species as understood in this paper. So, I here did not follow Narita et al. (2015) in their concept of *sculptilis* as a separate and reasonably derived species group of *Ameroseius*.

#### Key to species of *Ameroseius* occurring in Europe (females)

European species of *Ameroseius* can be identified using keys from Westerboer and Bernhard (1963), Karg (1971a, 1993) and Bregetova (1977). In their keys, these authors included six, eight or nine species, respectively. A smaller part of the world species of *Ameroseius* (17 species) can be identified using keys of Narita et al. (2015), but better portion of congeners should be exclusively identified using the primary species descriptions. A new key presented here contains 13 species considered to be of the European origin, including recently described species (*Ameroseius callosus*, *Ameroseius fungicola* and *Ameroseius lehtineni*), and a new species described in this study.

**Table:** 

1	Setae JV3 on (ventri)anal shield close to its anterolateral margins; soft integument between pseudo-metasternal platelets with a pair of rounded platelets; lateral margins of epistome produced into elongate, narrow, rounded and deeply dentate apex; movable digit of chelicera terminally with short and rounded hyaline appendage	***Ameroseius furcatus* Karg, 1971** (Plates 14–16)
–	All opisthogastric setae on soft integument, anal shield with only three circum-anal setae; a pair of rounded platelets between pseudo-metasternal platelets absent; epistome relatively flat, with anterior margin smooth or delicately serrate; cheliceral digits terminally with no hyaline appendages	**2**
2	Dorsal setae relatively shorter and subequal: j6 with their tips reaching between bases of j6 and J2, J2 reaching between bases of J2 and J4, and J4 clearly not reaching posterior margin of dorsal shield	**3**
–	Dorsal setae relatively longer, well differing in length: j6 and J2 reaching or overlapping bases of following setae, and J4 reaching or overlapping posterior margin of dorsal shield	**8**
3	Five pairs of opisthogastric setae present (JV4 absent); setae j1 conspicuously expanded, apically curved, apparently otherwise formed as other dorsal setae; vertex with a pair of horn-shaped structures between bases of j1	***Ameroseius corniculus* Karg, 1971** (Plates 9, 10)
–	Six pairs of opisthogastric setae present, including JV4; setae j1 progressively narrowed towards their tips, at most moderately thickened, similar to other dorsal setae; vertex without a pair of horn-shaped structures between j1 (at most an unpaired process present)	**4**
4	Setae z5 strongly reduced in length, minute, several times shorter than z6; fixed digit of chelicera with proximal masticatory area bidentate; corniculi hyaline, curved and directed laterally	**5**
–	Setae z5 and z6 subequal in length; fixed digit of chelicera with proximal masticatory area tridentate; corniculi well sclerotised, straight and directed forward	**6**
5	Dorsal setae relatively shorter: j6 with tips reaching between bases of j6 and J2; posterior dorsal surface between J2 and J4 rugose	***Ameroseius fungicola* Mašán, 1998** (Plates 11–13)
–	Dorsal setae relatively longer: j6 almost reaching bases of following setae J2; posterior dorsal surface between J2 and J4 coarsely reticulate	***Ameroseius callosus* Mašán, 1998** (Plate 3)
6	Medial dorsal surface between j6 and J2 with subtriangular sculptural pattern; anal shield subpentagonal, coarsely reticulate on surface; metapodal platelets enlarged and rounded	***Ameroseius sculptilis* Berlese, 1916** (Plates 26, 27)
–	Medial dorsal surface between j6 and J2 otherwise sculptured; anal shield suboval, with delicate reticulation on surface; metapodal platelets small, elongate and narrow	**7**
7	Setae Z5 and S5 progressively narrowed towards their tips, lanceolate; vertex with finely serrate horn-like process on each side and between bases of j1; setae j5 thinner and shorter than j6; anal shield with smaller suboval anus situated in posterior portion of the shield	***Ameroseius lidiae* Bregetova, 1977** (Plates 20, 21)
–	Setae Z5 and S5 progressively broadened towards their tips, oblanceolate; vertex densely denticulate; setae j5 and j6 similar in length and form; anal shield with larger elongate anus having central position on the shield	***Ameroseius ulmi* Hirschmann, 1963** (Plate 28)
8	Coarse dorsal shield sculpture additionally ornamented with plentiful tubercles arranged in rows and clusters; setae j5 shorter, with tips not reaching bases of j6; three pairs of setae (s2, r3, r4) on anteromarginal dorsal surface markedly reduced in length, minute	***Ameroseius lehtineni* Huhta & Karg, 2010** (Plate 19)
–	Coarse dorsal shield lacking tubercles on it surface; setae j5 longer, reaching or overlapping bases of j6; minute setae on dorsal surface absent, or their number and arrangement otherwise	**9**
9	Five pairs of opisthogastric setae present (JV4 absent); setae j1 conspicuously expanded and denate, apparently otherwise formed as other dorsal setae; dorsal setae notably robust	***Ameroseius corbiculus* (Sowerby, 1806)** (Plates 6–8)
–	Six pairs of opisthogastric setae present, including JV4; setae j1 progressively narrowed towards the tip, lanceolate, almost similar to other dorsal setae; dorsal setae normal in thickness	**10**
10	Dorsal setae extremely differing in length; setae j4, z5, z6, Z1–Z3 strongly reduced in length, subequal, smooth and minute; setae Z2 and Z3 similar in length; most dorsal setae greatly lengthened: J2 with tips reaching beyond posterior margin of dorsal shield	***Ameroseius georgei* (Turk, 1943)** (Plates 17, 18)
–	Dorsal setae less obviously differing in length; setae j4 reaching or overlapping bases of j5; setae Z2 and Z3 never similar in length; setae J2 never reaching beyond posterior margin of dorsal shield	**11**
11.	Setae z5 and z6 longer: z5 with tips reaching or overlapping bases of j6, z6 reaching or overlapping bases of Z1; setae JV5 less lengthened, with tips reaching between bases of JV5 and anus	***Ameroseius cavernosus* Westerboer, 1963** (Plates 4, 5)
–	Setae z5 and z6 shorter: z5 reaching between bases of z5 and j6, z6 reaching between bases of z6 and Z1; setae JV5 more lengthened, with tips reaching to or beyond anus	**12**
12.	Setae z5, z6, Z1 and Z2 similar in length, short; setae Z2 apparently shorter than Z3, with tips reaching between bases of Z2 and Z3	***Ameroseius longitrichus* Hirschmann, 1963** (Plates 22, 23)
–	Setae z5, z6, Z1 and Z2 becoming progressively longer posteriorly; setae Z2 lengthened, markedly longer than Z3, reaching far beyond bases of Z3	***Ameroseius renatae* sp. n.** (Figure 2, Plates 24, 25, 76G)

### Ameroseius aegypticus

Taxon classificationAnimaliaMesostigmataAmeroseiidae

El-Badry, Nasr & Hafez, 1979

Ameroseius aegypticus El-Badry, Nasr & Hafez, 1979: 2.
Ameroseius aegypticus . — Zaher 1986: 401; Narita et al. 2015: 395.

#### Type depository.

Not stated.

#### Type locality and habitat.

Egypt, Giza Governorate, Dokki, Ministry of Agriculture, decayed debris under wild-sage, *Lantana camara* (Verbenaceae).

#### Remarks.

A species closely related with *Ameroseius lidiae* Bregetova, 1977, if not identical. I have seen a series of photos displaying a specimen of this species from Egypt (kindly sent by R. Abo-Shnaf). Some differential characters for separating *Ameroseius aegypticus* and *A. lidiae* could be based on dorsal chaetotaxy (in *A. aegypticus*, dorsal setae slightly longer and more slender when compared with *A. lidiae*, the distance between left and right base in setal pairs j5, J2 and J4 relatively shorter; setae Z2 only slightly longer than Z1 and Z3).

### Ameroseius asper

Taxon classificationAnimaliaMesostigmataAmeroseiidae

Karg, 1994

Ameroseius asper Karg, 1994a: 117.
Ameroseius asper . — Narita et al. 2015: 395.

#### Type depository.

Museum für Naturkunde, Berlin, Germany.

#### Type locality and habitat.

Ecuador, Galápagos Islands, Floreana, littoral zone of lagoon, humid and rotting leaf litter.

#### Comparative material.

Ecuador: 2 ♀♀ (ZMB: 45134, holotype; ZMB: 45135, paratype) – 6. 4. 1985, Galapagos I., Floreana, Uferzone, 85-335, 6612-6613.

#### Remarks.

In the holotype, fixed digit of chelicerae with normal set of three denticles on proximal masticatory area (originally stated five to six teeth on fixed digit).

### Ameroseius avium

Taxon classificationAnimaliaMesostigmataAmeroseiidae

Karg, 1976

Ameroseius avium Karg, 1976: 541.

#### Type depository.

Museum für Naturkunde, Berlin, Germany; Hungarian Natural History Museum, Budapest, Hungary.

#### Type locality and habitat.

Chile, Tarapaca Region, Azapa, edge of marsh habitat, under rocks.

#### Comparative material.

Chile: 1 ♀ (HNHM: Meso-636, holotype) – 23. 11. 1965, Azapa (Prov. Tarapaca), unter Steinen, am Bach und am völlig ausgetrockneten Bachbett; 2 ♀♀ (ZMB: 39921, 39922, paratypes) – 23. 11. 1965, Azapa (Prov. Tarapaca), unter Steinen, am Bach und am völlig ausgetrockneten Bachbett, 2998, 2999; 1 ♀ (ZMB: 39923, paratype) – 30. 10. 1965, El Manzano (Prov. Santiago), unter blühenden Sträuchern im Tal, 3000.

#### Remarks.

I examined the holotype (♀) and three paratype females of the original series of Karg (1976) who has not specified the number of type specimens. All specimens examined are in poor condition for study, and only dorsal shield in those specimens can be examined with some difficulty. I was unable to check most of the characters on the venter of the idiosoma satisfactorily, especially the posterior margin of the sternal shield and the placement of st3. This species is very similar to those in the genus *Sinoseius* in that it has pinnate dorsal setae, curved epistome, oblong and anteriorly bilobed sternal shield, five pairs of opisthogastric setae, enlarged cheliceral digits and subcircular anal shield. Therefore, the systematic placement of *Ameroseius avium* in *Ameroseius* should be considered provisional and questionable. Karg (1976) did not state the number of dorsal shield setae; 26 pairs of setae were depicted in the original illustration, but I observed the normal complement of 29 pairs of the setae in the holotype. Karg overlooked some marginally inserted setae in medial part of the dorsal shield, and also overestimated the length of some very short setae inserted in the marginal region.

### Ameroseius bembix

Taxon classificationAnimaliaMesostigmataAmeroseiidae

Elsen, 1973

Ameroseius bembix Elsen, 1973: 750.
Ameroseius bembix subspec. ealensis Elsen, 1973: 752.
Ameroseius bembix . — Narita et al. 2015: 396.

#### Type depository.

Of *Ameroseius bembix* – Musée Royal de l’Afrique Centrale, Tervuren Belgium; of Ameroseius bembix subspec. ealensis – Musée Royal de l’Afrique Centrale, Tervuren Belgium.

#### Type locality and habitat.

Of *Ameroseius bembix* – Democratic Republic of Congo (as Zaire), l’Equateur Province, Bokuma, on sand wasp, *Bembix braunsii* (Hymenoptera); of Ameroseius bembix subspec. ealensis – Democratic Republic of Congo (as Zaire), Eala, on carpenter bee, *Xylocopa varipes* (as *Mesotrichia varipes atritarsis*) (Hymenoptera).

#### Comparative material.

Democratic Republic of Congo: 1 ♀ (MRAC: 142500, holotype) – Dec. 1935, Eala, Mesotrichia varipes var. atritarsis, leg. J. Ghesquière; 1 ♀ (MRAC: 142509, paratype) – 14. 11. 1931, Eala, *Sphex tuberculatus*, leg. J. Brédo; 1 ♀ (MRAC: 170345, paratype) – March 1935, Eala, Sphex haemorrhoidalis var. volubis, leg. A. Corbisier; 1 ♀ (MRAC: 142510, paratype; published as 142501) – July 1952, Equateur, Bokuma, Sphex haemorrhoidalis var. pulchripennis, leg. R. P. Lootens (all type specimens labelled *Ameroseius bembix ealensis*).

#### Remarks.

I consider both subspecies to be identical although no type material of *Ameroseius bembix bembix* was examined for the purpose of this study. Given that the characteristics of the type specimens of *Ameroseius bembix ealensis* are within the range of variability found in *Ameroseius leclercqi* Elsen, 1973, I suspect *A. bembix ealensis* to be a synonym of *A. leclercqi*.

### Ameroseius benoiti

Taxon classificationAnimaliaMesostigmataAmeroseiidae

Elsen, 1973

Ameroseius benoiti Elsen, 1973: 735.
Ameroseius benoiti . — Narita et al. 2015: 396.

#### Type depository.

Musée Royal de l'Afrique Centrale, Tervuren Belgium.

#### Type locality and habitat.

Democratic Republic of Congo (as Zaire), Katompi, Katanga, on carpenter bee, *Xylocopa lepeletieri* (as *Mesotrichia lepeletieri*) (Hymenoptera).

#### Comparative material.

Democratic Republic of Congo: 1 ♀ (MRAC: 136509, holotype) – Oct. 1920, Katanga, Katompi, *Mesotrichia lepeletieri*, leg. Ch. Seydel; 1 ♀ (MRAC: 142506, paratype) – Kasaï, Kondue, Chlorion ciliatum var. maxillae, leg. E. Luja; 1 ♀ (MRAC: 170379, paratype; published as 142507) – Dec. 1932, Kapanga, *Chlorion incomptum*, leg. G. F. Overlaet; 1 ♀ (MRAC: 142507, paratype) – Nov. 1932, Lulua, Kapanga, Chlorion ciliatum var. instabile, leg. G. F. Overlaet.

#### Remarks.

No fundamental differences could be found between the type specimens of the four species described by Elsen (1973), namely *Ameroseius benoiti*, *Ameroseius gillardinae* and *Ameroseius longitarsis*. These species were erected in my opinion on untenable characters. For example, I could not detect any of the illustrated structures of sperm induction system as they were presented in original figures 1–18 given by Elsen (1973).

### Ameroseius californicus

Taxon classificationAnimaliaMesostigmataAmeroseiidae

Garman & McGregor, 1956

Ameroseius californicus Garman & McGregor, 1956: 13.

#### Type depository.

Los Angeles County Museum, California, USA.

#### Type locality and habitat.

USA, California, Stanton, on citrus tree (Rutaceae).

#### Comparative material.

U.S.A.: 1 ♀ (LACM: ENT 160784, holotype) – 17. 3. 1955, near Stanton, California, citrus, No. 3-31-55.

#### Remarks.

Garman and McGregor (1956) did not comment on the number of setae on the dorsal shield in their description of *Ameroseius californicus*, although their illustration shows 26 pairs of setae. I detected the usual complement of 29 pairs in the holotype.

### Ameroseius callosus

Taxon classificationAnimaliaMesostigmataAmeroseiidae

Mašán, 1998

Plate 3

Ameroseius callosus Mašán, 1998: 648.

#### Type depository.

Institute of Zoology, Slovak Academy of Sciences, Bratislava, Slovakia.

#### Type locality and habitat.

Slovakia, Podunajská Nížina Lowland, Brunovce, in floodplain forest, on fruiting body of willow bracket fungus, *Phellinus igniarius* (Basidiomycota, Hymenochaetaceae).

#### Published material from Slovakia.

Trnavská Pahorkatina Wold: Brunovce Village (Mašán 1998).

#### New material from Slovakia.

Ipeľská Kotlina Basin: 1 ♀ – 23. 6. 1997, Ipeľské Predmostie Village, Ryžovisko Forest, willow-poplar flood-plain forest (*Salici-Populetum*), wood-destroying fungus *Trametes* sp., altitude 130 m, leg. P. Mašán. Podunajská Rovina Flatland: 4 ♀♀ – 5. 10. 2004, Bratislava Capital, Mlynské Nivy Settlement, park with willows and poplars, individual collecting on fresh wood-destroying fungi, altitude 135 m, leg. P. Mašán; 3 ♀♀ – 14. 6. 2015, Bratislava Capital, Čunovo Settlement, wood-destroying fungus (*Phellinus* sp.) growing on old walnut tree (*Juglans regia*), altitude 130 m, leg. P. Mašán. Považský Inovec Mts.: 1 ♀ – 17. 5. 1997, Hrádok Village, Hrádocká Dolina Valley, broad-leaved deciduous forest, individual collecting on wood-destroying fungi, altitude 450 m, leg. P. Mašán. Trnavská Pahorkatina Wold: 1 ♀ – 18. 5. 1997, Brunovce Village, Váh River (alluvium), degraded willow-poplar flood-plain forest (*Salici-Populetum*), individual collecting on wood-destroying fungus *Trametes* sp., altitude 170 m, leg. P. Mašán.

#### Remarks.

*Ameroseius callosus* shows a close resemblance to *Ameroseius fungicola*, except for some characters of the dorsal shield (sculpture and relative length of setae). Although *A. callosus* may be easy and reliably separated from *A. fungicola*, several pieces of evidence show that this species could be only a polymorphic form and synonym of *A. fungicola*. This hypothesis should be confirmed by further comparative studies, based also on laboratory rearing and molecular analyses, to establish their correct systematic status. Polymorphism has been mentioned in a few mesostigmatic mites of the families Ascidae, Laelapidae, Macrochelidae and Parasitidae.

### Ameroseius cavernosus

Taxon classificationAnimaliaMesostigmataAmeroseiidae

Westerboer, 1963

Plates 4 , 5

Ameroseius cavernosus Westerboer (in Westerboer & Bernhard, 1963: 526).
Ameroseius cavernosus . — Karg 1971a: 232; Bregetova 1977: 161; Karg 1993: 229.

#### Type depository.

Not stated.

#### Type locality and habitat.

Spain, Monte Montera Mt. (altitude 850 m), mixed forest predominated by beech and growing on clay slate, in humid leaf litter and humus.

#### New material from Slovakia.

Hronská Pahorkatina Wold: 2 ♀♀ – 12. 11. 2015, Vieska Nad Žitavou Village, Mlyňany Arboretum, deciduous forest, soil with raw humus and wood detritus, altitude 210 m, leg. P. Mašán. Ipeľská Pahorkatina Wold: 1 ♀ – 13. 11. 2015, Horša Village, Horšianska Dolina Valley, hornbeam forest (*Carpinion betuli*) with oak (*Quercus* sp.) and elm (*Ulmus* sp.), leaf litter and soil detritus, altitude 200 m, leg. P. Mašán. Zemplínske Vrchy Hills: 2 ♀♀ – 10. 11. 2006, Ladmovce Village, Kašvár Mt., steppe with small groups of acacia-trees (*Robinia pseudoacacia*) and oaks (*Quercus* sp.), leaf litter and soil detritus, altitude 160 m, leg. P. Mašán.

#### Remarks.

The illustration of the original description shows only 26–27 pairs of dorsal shield setae. I detected the usual complement of 29 pairs in the specimens from Slovakia.

### Ameroseius corbiculus

Taxon classificationAnimaliaMesostigmataAmeroseiidae

(Sowerby, 1806)

Plates 6 , 7 , 8

Acarus corbicula Sowerby, 1806: Tab. 66/14.
Seius hirsutus C. L. Koch, 1839: Fasc. 24/12. Synonymy by Vitzthum (1942).
Seius echinatus C. L. Koch, 1839: Fasc. 24/13. Synonymy by Vitzthum (1942).
Seius echinatus . — Canestrini and Fanzago 1876: 130; Kramer 1882: 427; Berlese 1892: 38.
Gamasus echinatus . — Kramer 1881: 433.
Laelaps echinatus . — Berlese 1882: 638.
Seuis (sic) hirsutus. — Berlese 1887b: 41/2.
Seius hirsutus . — Berlese 1892: 38.
Seiulus hirsutus . — Halbert 1915: 77.
Ameroseius hirsutus . — Berlese 1916a: 33; Castagnoli and Pegazzano 1985: 181.
Ameroseius corbicula . — Oudemans 1929: 400; Turk 1943: 859; Westerboer and Bernhard 1963: 518; Bregetova 1977: 161; Lapiņa 1988: 38.
Cornubia ornata Turk, 1943: 859. Synonymy by Turk (1953).
Ameroseius ornata . — Turk 1953: 12.
Ameroseius echinatus . — Schweizer 1961: 85; Evans and Till 1979: 230.
Ameroseius corbiculus . — Karg 1971a: 233; Vasilyeva et al. 1976: 26; Karg 1993: 230; Kontschán 2007: 100; Kazemi and Rajaei 2013: 64; Khalili-Moghadam and Saboori 2014: 674; Kontschán et al. 2016: 135.
Ameroseius crassisetosus Ye & Ma, 1993: 86. **Syn. n.**
Ameroseius qinghaiensis Li & Yang, 2000: 65. **Syn. n.** Synonymy with Ameroseius crassisetosus Ye & Ma, 1993 by Ma, 2006.
Ameroseius (Ameroseius) corbiculus . — Hajizadeh et al. 2013a: 150.
Ameroseius corbicus (sic). — Nemati et al. 2013: 19.
Ameroseius curbicula (sic). — Khalili-Moghadam and Saboori, 2014: 680.
Ameroseius norvegicus Narita, Abduch & Moraes (in Narita et al., 2015: 391). **Syn. n.**
Ameroseius crassisetosus . — Khalili-Moghadam and Saboori 2016: 547.
Ameroseius qinghaiensis . — Khalili-Moghadam and Saboori 2016: 547. not Ameroseius qinghaiensis Ma, 2008: 748.

#### Type depository.

Of *Acarus corbicula* – not stated; of *Seius echinatus* – not stated; of *Seius hirsutus* – not stated; of *Cornubia ornata* – not stated; of *Ameroseius crassisetosus* – Xinjiang Institute for Endemic Disease Control and Research, Urumqi, China; of *Ameroseius qinghaiensis* – Qinghai Institute for Endemic Disease Prevention and Control, Qinghai, China; of *Ameroseius norvegicus* – Departamento de Entomologia e Acarologia, Escola Superior de Agricultura Luiz de Queiroz, Universidade de São Paulo, Piracicaba, São Paulo, Brazil.

#### Type locality and habitat.

Of *Acarus corbicula* – British Isles, in mollusc ctenidium moss, *Ctenidium molluscum* (as *Hypnum molluscum*) (Bryophyta); of *Seius echinatus* – Germany, in unspecified substrate (orchard); of *Seius hirsutus* – Germany, in unspecified substrate (shore zone of a pond, in humid soil); of *Cornubia ornata* – United Kingdom, England, Camborne, garden of Rosewarne Farm, in dead leaves; of *Ameroseius crassisetosus* – China, Xinjiang Region, Nilka County, on wood mouse, *Apodemus sylvaticus* (Mammalia, Rodentia); of *Ameroseius qinghaiensis* – China, Qinghai Province, Huangzhong County, Zongzhai Area, in humus; of *Ameroseius norvegicus* – Norway, Buskerud County, Sylling, strawberry field, in litter.

#### Comparative material.

Belgium: 1 ♂ (MRAC: 170354) – 6. 10. 1967, Anvers, zoological garden, litière de cobaye (*Cavia porcellus*), leg. P. Elsen (labelled Ameroseiidae). Germany: 1 ♂, 1 ♂ (ZMB: 40082, 40083) – July 1957, Brandenburg, Potsdam-Mittelmark, Stahnsdorf bei Berlin, 2969, 2970; 1 ♂ (ZMB: 40084) – 31. 7. 1958, Brandenburg, Potsdam-Mittelmark, Stahnsdorf bei Berlin, Bodenfall, 2971. Iran: 1 ♀ (CJH) – Guilan Province, olive garden, soil sample, leg. and det. J. Hajizadeh. Italy: 1 ♀, 1 DN (ISZA: 3/31) – Cison di Valmarino, Treviso, musco; 3 ♀♀ (ISZA: 27/8, 169/13) – Castions di Strada, Udine, foglie marce (all labelled *Ameroseius hirsutus*). Netherlands: 1 ♀ (RMNH: ACA.P.4055) – 28. 1. 1923, Arnhem, in de steelgroeve van een appel, leg. Oudemans; 1 ♀ (RMNH: ACA.P.4056) – 7. 5. 1916, Valkeveen (Gooi), rotte bladeren, leg. Mac Gillavry. Norway: 2 ♀♀ (ESALQ: T-MZLQ 3342 C=7766, holotype and paratype), 1 ♀ (ESALQ: T-MZLQ 3344 C=7766, paratype) – 27. 4. 2011, Silling, strawberry field (tunel), Ex: litter, leg. I. Kling (labelled *Ameroseius norvegicus*). United Kingdom: 1 ♀ (BMNH) – 12. 4. 1934, Rednal, nest, leg. T. Warwick, det. G. O. Evans.

#### Published material from Slovakia.

Borská Nížina Lowland: Bratislava Capital, Devín Settlement, Devínske Jazero Lake (Kalúz 2006). Jakubov Village, Jakubovské Rybníky Fishponds (Fenďa 2005, Fenďa and Schniererová 2005). Malacky Town, Vinohrádok Area (Krištofík et al. 2005). Závod Village, Abrod Meadow (Kalúz and Čarnogurský 2000, Kalúz 2003). Bukovské Vrchy Hills: Nová Sedlica Village, Vrch Hrbu Mt.; Ulič Village (Fenďa and Mašán 2003). Cerová Vrchovina Highland: Chrámec Village, Vinohrady Area; Gemerský Jablonec Village, Petrovce Reservoir; Šurice Village; Šurice Village, Soví Hrad Mt.; Tachty Village (Fenďa and Mašán 2009). Ipeľská Pahorkatina Wold: Žemberovce Village (Kalúz 1994a). Košická Kotlina Basin: Haniska Village, Grajciar Area (Várfalvyová et al. 2010). Kechnec Village (Mašán and Stanko 2005). Laborecká Vrchovina Highland: Stakčín Village, Starina Dam, Kremenica Forest (Fenďa and Mašán 2003). Malé Karpaty Mts.: Borinka Village (Mašán et al. 1994). Bratislava Capital, Devín Settlement, Devínska Kobyla Mt.; Bratislava Capital, Devín Settlement, Devínska Lesostep Forest (Kalúz 2005, cited as *Ameroseius corbicula*). Podunajská Rovina Flatland: Bodíky Village, Bodícka Brána Forest (Kalúz 1997). Dobrohošť Village, Dunajské Kriviny Forest; Veľké Blahovo Village (Krištofík et al. 1993). Gabčíkovo Town, Istragov Forest (Kalúz 1995b). Ivanka Pri Dunaji Village (Mašán and Krištofík 1993). Svätý Jur Town, Šúr Forest, Šúrsky Rybník Pond (Fenďa et al. 1998). Slovenský Kras Karst: Silica Village, Pod Fabiankou Forest (Kalúz 1992). Silica Village, Jašteričie Jazierko Lake (Kalúz 1995a, cited as *Ameroseius corbicula*). Trnavská Pahorkatina Wold: Pezinok Town, Gánok Area (Kalúz 1994b). Východoslovenská Pahorkatina Wold: Egreš Village, Zadné Hony Area (Kováč et al. 1999). Východoslovenská Rovina Flatland: Čičarovce Village (Kováč et al. 1999). Svätuše Village (Várfalvyová et al. 2010).

#### New material from Slovakia.

Borská Nížina Lowland: 1 ♀ – 24. 7. 1998, Stupava Town, littoral reed stand (*Phragmition*), leaf litter and soil detritus, altitude 180 m, leg. P. Mašán; 1 ♀, 1 ♂ – 25. 4. 2000, Suchohrad Village, reed stand (*Phragmition*), leaf litter and soil detritus, altitude 150 m, leg. P. Mašán; 1 ♀, 1 ♂ – 27. 6. 2002, Malé Leváre Village, grassy dike of Morava River nearly wet willow-poplar flood-plain forest (*Salici-Populetum*), nest of unidentified small mammal, altitude 150 m, leg. P. Mašán; 1 ♀ – 8. 1. 2006, Borský Svätý Jur Village, village residence, subterraneous night-cellar, decaying potatoes, altitude 170 m, leg. P. Mašán. Burda Hills: 1 ♀ – 9. 11. 1997, Kamenica Nad Hronom Village, Kováčovské Kopce-juh Forest, oak forest (*Quercetum cerris*), leaf litter and soil detritus, altitude 330 m, leg. P. Mašán. Horehronské Podolie Basin: 1 ♀ – 22. 5. 2002, Zlatno Village, Zlatnianske Skalky Forest, pine forest (*Pinus* sp.), soil and detritus from ant-hill of *Formica* sp. (Hymenoptera, Formicidae), altitude 755 m, leg. P. Mašán. Ipeľská Kotlina Basin: 4 ♀♀, 5 ♂♂, 2 DNs – 23. 6. 1997, Ipeľské Predmostie Village, Ryžovisko Forest, willow-poplar flood-plain forest (*Salici-Populetum*), growth of *Carex* sp., leaf litter and soil detritus, altitude 130 m, leg. P. Mašán; 1 ♀ – 23. 6. 1997, Ipeľské Predmostie Village, Ryžovisko Forest, willow-poplar flood-plain forest (*Salici-Populetum*), wood-destroying fungus *Trametes* sp., altitude 130 m, leg. P. Mašán. Malé Karpaty Mts.: 1 ♀ – 25. 6. 1990, Bratislava Capital, Železná Studienka Forest, broad-leaved deciduous forest, nest of *Motacilla cinerea* (Aves), altitude 250 m, leg. J. Krištofík and A. Darolová; 2 ♀♀ – 30. 5. 1991, Bratislava Capital, Železná Studienka Forest, broad-leaved deciduous forest, individual collecting under bark, stones and pieces of wood, altitude 350 m, leg. P. Mašán; 3 ♀♀ – 3. 5. 1998, Bratislava Capital, zoological garden, oak-hornbeam forest (*Querco-Carpinetum*), individual collecting under bark, stones and pieces of wood, altitude 180 m, leg. P. Mašán. Podunajská Rovina Flatland: 2 ♀♀, 1 ♂ – 28. 6. 1989, Veľké Blahovo Village, fish-ponds, shore reed stand (*Phragmition*), nest of *Circus aeruginosus* (Aves), altitude 120 m, leg. J. Krištofík and A. Darolová; 2 ♀♀ – 5. 6. 1990, Dobrohošť Village, willow-poplar flood-plain forest (*Salici-Populetum*), nest of *Sylvia atricapila* (Aves), altitude 120 m, leg. J. Krištofík and A. Darolová; 3 ♀♀ – 24. 7. 1990, Veľké Blahovo Village, fish-ponds, shore reed stand (*Phragmition*), nest of *Circus aeruginosus* (Aves), altitude 120 m, leg. J. Krištofík and A. Darolová; 3 ♀♀, 1 ♂ – 25. 6. 1991, Veľké Blahovo Village, fish-ponds, shore reed stand (*Phragmition*), nest of *Aythya ferina* (Aves), altitude 120 m, leg. J. Krištofík and A. Darolová; 1 ♀ – 18. 6. 1994, Dolný Štál, shore reed stand (*Phragmition*), nest of *Emberiza schoeniclus* (Aves), altitude 120 m, leg. J. Krištofík and A. Darolová; 4 ♀♀, 1 ♂ – 28. 6. 1996, Gabčíkovo Town, Istragov Forest, willow-poplar flood-plain forest (*Salici-Populetum*), leaf litter and soil detritus, altitude 120 m, leg. P. Mašán; 1 ♀ – 21. 4. 1998, Veľký Meder Town, ruderal, nest of *Clethrionomys glareolus* (Mammalia), altitude 110 m, leg. P. Mašán; 1 ♀ – 8. 9. 1998, Veľké Kosihy Village, littoral reed stand (*Phragmition*), leaf litter and soil detritus, altitude 120 m, leg. P. Mašán; 1 ♀ – 28. 4. 1999, Medveďov Village, reed stand (*Phragmition*), leaf litter and soil detritus, altitude 120 m, leg. P. Mašán; 3 ♀♀ – 3. 5. 2006, Bratislava Capital, air-port, meadow, burrow entry of *Spermophilus citellus* (Mammalia), rotting leaves and vegetation rests, altitude 150 m, leg. P. Mašán. Považský Inovec Mts.: 1 ♂ – 23. 8. 1992, Hrádok Village, Hrádocká Dolina Valley, pasture, dunghill substrate, altitude 350 m, leg. P. Mašán; 8 ♀♀ – 10. 5. 1998, Lúka Village, steppe, straw and miscellaneous organic detritus, altitude 250 m, leg. P. Mašán; 2 ♀♀ – 7. 6. 1998, Lúka Village, field, sample of old and drier dung, altitude 300 m, leg. P. Mašán. Turčianska Kotlina Basin: 11 ♀♀, 4 ♂♂ – 11. 6. 2000, Turčianske Teplice Town, Diviaky Settlement, nest of *Motacilla flava* (Aves), altitude 490 m, leg. M. Dobrotka. Žilinská Kotlina Basin: 4 ♀♀ – 14. 9. 1994, Stráňavy Village, ruderal with willow (*Salix* sp.), leaf litter and soil detritus, altitude 350 m, leg. I. Országh.

#### Remarks.

Berlese (1892, 1916a) considered *Seius echinatus* C. L. Koch, 1839 to be identical to *Seius hirsutus* C. L. Koch, 1839. Later, Vitzthum (1942) stated that these two species are synonymous with *Acarus corbicula* Sowerby, 1806. Moreover, Vitzthum (1942) added another species under the synonymy with *A. corbicula*, namely *Seius muricatus* C. L. Koch, 1839, but his interpretation is not accepted in this paper because *S. muricatus* is the type species of *Aceoseius* Sellnick, 1941 (now relegated into synonymy with *Lasioseius* Berlese, 1916).

The dorsal shield of *Ameroseius corbiculus* was illustrated by Westerboer and Bernhard (1963) as having 30 pairs of quite slim setae instead of 29 pairs of apparently stout and robust setae. In addition, Karg (1971a) erroneously illustrated a male specimen of this species with six instead of five pairs of opisthogastric setae (see his page 15). These facts could lead to misinterpretation of the morphological characters resulting in an incorrect identification of this easily recognizable species.

I have examined three type specimens of *Ameroseius norvegicus*, a species decribed by Narita et al. (2015) from Norway. However, the slide labelled „Holotype“ contains two individuals. They are both females, and are clearly conspecific, in a perfect agreement with the primary description and original illustrations. I here designate as lectotype the specimen nearer to the left-hand edge of the slide when the labels are in the upright position. I have ringed this specimen with black ink and labelled the slide with the words „Lectotype ringed“. There is no doubt that these type specimens from Norway are conspecific with those widely distributed in Slovakia and other European countries (including those from Asia, Iran), and known under the name *Ameroseius corbiculus*. Narita et al. (2015) added complete leg chaetotaxy to the description of *A. norvegicus*. According to their original data, genu I and femur IV would have respectively 11 and 5 setae, but in the type specimens I found typical 12 and 6 setae instead (including two anterolateral setae on genu I, and a ventral seta on femur IV).

I have not examined type specimens of *Ameroseius corbiculus* (no type material exists for this species), *Ameroseius crassisetosus* and *Ameroseius qinghaiensis*, but the distinctions made in the original descriptions are based on characteristics that vary considerably, and I do not hesitate to propose the above synonymy.

### Ameroseius corniculus

Taxon classificationAnimaliaMesostigmataAmeroseiidae

Karg, 1971

Plates 9 , 10

Ameroseius corniculus Karg, 1971a: 233.
Ameroseius corniculus . — Vasilyeva et al. 1976: 25; Bregetova 1977: 163; Karg 1993: 231; Kontschán 2007: 101; Kazemi and Rajaei 2013: 65; Nemati et al. 2013: 19.
Ameroseius (Ameroseius) corniculus . — Hajizadeh et al. 2013a: 150.

#### Type depository.

Museum für Naturkunde, Berlin, Germany.

#### Type locality and habitat.

Germany, Magdeburg Area, Holtemme (labelled: Mahndorf) at Halberstadt, river bank (labelled: meadow), in humus.

#### Comparative material.

Germany: 1 ♀ (ZMB: 40091, holotype) – 5. 9. 1963, Mahndorf b. Halberstadt, Wiese a. d. Holt., 2983.

#### Published material from Slovakia.

Cerová Vrchovina Highland: Šiatorská Bukovinka Village, Dolina Bukovinského Potoka Valley (Fenďa and Mašán 2009).

#### New material from Slovakia.

Borská Nížina Lowland: 1 ♀ – 26. 8. 1997, Plavecký Štvrtok Village, pine forest (*Pinus sylvestris*), individual collecting under bark, stones and pieces of wood, altitude 170 m, leg. P. Mašán. Burda Hills: 1 ♀ – 9. 11. 1997, Kamenica Nad Hronom Village, Kováčovské Kopce-juh Forest, oak forest (*Quercetum cerris*), leaf litter and soil detritus, altitude 330 m, leg. P. Mašán. Hronská Pahorkatina Wold: 3 ♀♀ – 12. 11. 2015, Vieska Nad Žitavou Village, Mlyňany Arboretum, deciduous forest, soil with wood detritus, altitude 210 m, leg. P. Mašán. Ipeľská Pahorkatina Wold: 1 ♀ – 25. 5. 2004, Horša Village, Horšianska Dolina Valley, hornbeam forest (*Carpinion betuli*) with oak (*Quercus* sp.) and elm (*Ulmus* sp.), leaf litter and decaying plant remnants, altitude 200 m, leg. P. Mašán; 9 ♀♀ – 13. 11. 2015, Santovka Village, park, growth of horse-chestnut (*Aesculus hippocastanum*), on unidentified wood-destroying fungi, altitude 155 m, leg. P. Mašán. Malé Karpaty Mts.: 1 ♀ – 30. 5. 1991, Bratislava Capital, Železná Studienka Forest, broad-leaved deciduous forest, individual collecting under bark, stones and pieces of wood, altitude 350 m, leg. P. Mašán. Podunajská Rovina Flatland: 1 ♀ – 8. 5. 2004, Bratislava Capital, Čuňovo Settlement, Ostrovné Lúčky Forest, forest steppe, moss and wood detritus, altitude 135 m, leg. P. Mašán; 10 ♀♀, 5 ♂♂ – 6. 10. 2012, Svätý Jur Town, Šúr Forest, forest steppe with oak (*Quercus* sp.), unidentified decaying wood-destroying fungi on oak stem in soil detritus, altitude 130 m, leg. P. Mašán. Považský Inovec Mts.: 6 ♀♀ – 13. 10. 2012, Hrádok Village, Hrádocká Dolina Valley, edge of broad-leaved deciduous forest, decaying wood-destroying fungi, altitude 290 m, leg. P. Mašán. Trnavská Pahorkatina Highland: 5 ♀♀, 1 ♂ – 21. 7. 2002, Častkovce Village, park with lime (*Tilia* sp.), plane *Platanus orientalis*, poplar (*Populus* sp.) and maple (*Acer* sp.), leaf litter and soil detritus, altitude 180 m, leg. P. Mašán. Veľká Fatra Mts.: 4 ♀♀ – 21. 7. 2004, Liptovské Revúce Village, Veľká Rakytová Dolina Valley, deciduous forest, individual collecting under bark and on wood-destroying fungi, altitude 780 m, leg. P. Mašán; 1 ♂ – 21. 7. 2004, Liptovské Revúce Village, Veľká Rakytová Dolina Valley, beech forest (*Fagion sylvaticae*), rocky canyon, moss, altitude 780 m, leg. P. Mašán. Vihorlatské Vrchy Hills: 1 ♂ – 7. 9. 2005, Humenné Town, Humenský Sokol Forest, Červená Skala Mt., oak-hornbeam forest (*Querco-Carpinetum*) with cherry (*Cerasus avium*), leaf litter and soil detritus, altitude 400 m, leg. P. Mašán. Vtáčnik Mts.: 1 ♀ – 4. 11. 2003, Ostrý Grúň Village, Hlboká Dolina Valley, Pokuty, alluvium of brook with *Petasites* sp., mixed forest (*Ulmus* sp., *Fagus sylvatica* and *Abies alba*), moist soil detritus and moss, altitude 650 m, leg. P. Mašán.

### Ameroseius coronarius

Taxon classificationAnimaliaMesostigmataAmeroseiidae

De Leon, 1964

Ameroseius coronarius De Leon, 1964: 213.

#### Type depository.

„Author’s collection“.

#### Type locality and habitat.

USA, Tennessee, Erwin, under bark of dead oak limb.

#### Remarks.

A species closely related with *Ameroseius ulmi* Hirschmann, 1963, if not identical. I have seen several specimens of this species from Ohio, the United States (from J. C. Moser Mite Collection), labelled *Ameroseius ulmi*. A Moser’s specimen from Utah, labelled as *A. ulmi*, belonged to an undescribed species.

### Ameroseius cuiqishengi

Taxon classificationAnimaliaMesostigmataAmeroseiidae

Ma, 1995

Ameroseius cuiqishengi Ma, 1995: 92.
Ameroseius cuiqisheng (sic). — Ho et al. 2010: 92.

#### Type depository.

National Base of Plague and Brucellosis Control, Baicheng, China.

#### Type locality and habitat.

China, Jilin Province, Baicheng, under decomposed bark of poplar tree.

### Ameroseius decemsetosus

Taxon classificationAnimaliaMesostigmataAmeroseiidae

Micherdziński, 1965

Ameroseius decemsetosus Micherdziński, 1965: 26.

#### Type depository.

Zoological Museum, Jagiellonian University, Krakow, Poland.

#### Type locality and habitat.

Vietnam, Cha-Pa, SW from Lao-Kay (at Geophysical Station), in mould under wood trunk.

### Ameroseius dendrovagans

Taxon classificationAnimaliaMesostigmataAmeroseiidae

Flechtmann & Flechtmann, 1985

Ameroseius dendrovagans Flechtmann & Flechtmann, 1985: 393.
Ameroseius dendrovagans . — Flechtmann 1985: 397; Narita et al. 2015: 395.

#### Type depository.

Setor de Zoologia, Departamento de Entomologia e Acarologia (cited as Departamento de Zoologia), Escola Superior de Agricultura Luiz de Queiroz, Universidade de São Paulo, Piracicaba, São Paulo, Brazil.

#### Type locality and habitat.

Brazil, Minas Gerais State, Sacramento, in galleries of unidentified bark beetles (Coleoptera, Curculionidae, Scolytinae), in *Pinus* sp. (Pinaceae).

#### Remarks.

Although Flechtmann and Flechtmann (1985) stated 28 pairs of setae in the original description of this species, 29 pairs of asymmetrically situated setae can be seen in their illustration.

### Ameroseius denticulatus

Taxon classificationAnimaliaMesostigmataAmeroseiidae

Gu & Guo, 1997

Ameroseius denticulatus Gu & Guo, 1997: 137.

#### Type depository.

Department of Parasitology, Medical School, Nanjing University, Nanjing, China.

#### Type locality and habitat.

China, Yunnan Province, Xiaguan, on Yunnan red-backed vole, *Eothenomys miletus* (Mammalia, Rodentia, Cricetidae).

### Ameroseius elegantissimus

Taxon classificationAnimaliaMesostigmataAmeroseiidae

Ishikawa, 1984

Ameroseius elegantissimus Ishikawa, 1984: 93.

#### Type depository.

Biological Laboratory, Matsuyama Shinonome Junior College, Matsuyama, Japan.

#### Type locality and habitat.

Japan, Ehime Prefecture, Kamiukena-gun, Oda-chô, on yellow-spotted longicorn beetle, *Psacothea hilaris* (Coleoptera, Cerambycidae).

### Ameroseius fungicola

Taxon classificationAnimaliaMesostigmataAmeroseiidae

Mašán, 1998

Plates 11 , 12 , 13

Ameroseius fungicolis Mašán, 1998: 645.
Ameroseius fungicolis . — Kazemi and Rajaei 2013: 65.
Ameroseius (Ameroseius) fungicolis . — Hajizadeh et al. 2013a: 150.

#### Type depository.

Institute of Zoology, Slovak Academy of Sciences, Bratislava, Slovakia.

#### Type locality and habitat.

Slovakia, Podunajská Nížina Lowland, Brunovce, in floodplain forest, on fruiting body of willow bracket fungus, *Phellinus igniarius* (Basidiomycota, Hymenochaetaceae).

#### Published material from Slovakia.

Cerová Vrchovina Highland: Teplý Vrch Village, Hikóriový Porast Forest (Fenďa and Mašán 2009). Malé Karpaty Mts.: Bratislava Capital, Železná Studienka Forest (Mašán 1998). Trnavská Pahorkatina Wold: Brunovce Village; Horná Streda Village (Mašán 1998).

#### New material from Slovakia.

Ipeľská Kotlina Basin: 141 ♀♀ – 23. 6. 1997, Ipeľské Predmostie Village, Ryžovisko Forest, willow-poplar flood-plain forest (*Salici-Populetum*), wood-destroying fungus *Trametes* sp., altitude 130 m, leg. P. Mašán; 5 ♀♀ – 24. 6. 1997, Ipeľské Predmostie Village, pasture in degraded willow-poplar flood-plain forest (*Salici-Populetum*), wood-destroying fungus *Trametes* sp., altitude 130 m, leg. P. Mašán; 17 ♀♀ – 13. 11. 2015, Santovka Village, park, growth of horse-chestnut (*Aesculus hippocastanum*), on unidentified wood-destroying fungi, altitude 155 m, leg. P. Mašán. Malé Karpaty Mts.: 36 ♀♀ – 7. 4. 1995, Bratislava Capital, Železná Studienka Forest, broad-leaved deciduous forest, individual collecting on wood-destroying fungus (*Trametes* sp.), altitude 350 m, leg. P. Mašán. Považský Inovec Mts.: 20 ♀♀ – 17. 5. 1997, Hrádok Village, Hrádocká Dolina Valley, broad-leaved deciduous forest, individual collecting on wood-destroying fungi, altitude 450 m, leg. P. Mašán; 32 ♀♀, 11 ♂♂ – 26. 5. 2013, Lúka Village, Srnia Dolina Valley, alluvium of brook, wood-destroying fungus (*Phellinus* sp.) growing on old walnut tree (*Juglans regia*), altitude 175 m, leg. P. Mašán; 136 ♀♀, 41 ♂♂ – 1. 11. 2013, Lúka Village, garden, wood-destroying fungus (*Phellinus* sp.) growing on old walnut tree (*Juglans regia*), altitude 175 m, leg. P. Mašán. Trnavská Pahorkatina Wold: 49 ♀♀ – 15. 4. 1995, Horná Streda Village, Váh River (alluvium), degraded willow-poplar flood-plain forest (*Salici-Populetum*), individual collecting on wood-destroying fungus *Trametes* sp., altitude 170 m, leg. P. Mašán; 7 ♀♀ – 18. 5. 1997, Brunovce Village, Váh River (alluvium), degraded willow-poplar flood-plain forest (*Salici-Populetum*), individual collecting on wood-destroying fungus *Trametes* sp., altitude 170 m, leg. P. Mašán.

#### Remarks.

*Ameroseius fungicola* appears to be a specialised mycetobiont primarily colonising the fruiting bodies of the willow bracket fungus, *Phellinus igniarius*, where it occurs on the active hymenophore. This polypore fungus is common mostly throughout the temperate Northern Hemisphere, where it grows mainly on living willow, poplar and aspen. *A. fungicola* is a very common and abundant species, often with several hundred mites in a single fungus, occasionally including all post-embryonic stages.

### Ameroseius furcatus

Taxon classificationAnimaliaMesostigmataAmeroseiidae

Karg, 1971

Plates 14 , 15 , 16

Ameroseius furcatus Karg, 1971a: 231.
Ameroseius pseudofurcatus Livshits & Mitrofanov, 1975: 462. **Syn. n.**
Ameroseius furcatus . — Bregetova 1977: 158; Karg 1993: 228; Hajizadeh et al. 2013b:68.

#### Type depository.

Of *Ameroseius furcatus* – Museum für Naturkunde, Berlin, Germany; of *Ameroseius pseudofurcatus* – Nikita Botanical Gardens, National Scientific Center, Yalta, Crimea, Russia (the type specimens not found and probably lost, based on personal communication from Alex Khaustov).

#### Type locality and habitat.

Of *Ameroseius furcatus* – Germany, Naturschutzgebiet Kalktuffmiedermoor at Oechsen (Rhön), in tussocks of grass with roots; of *Ameroseius pseudofurcatus* – Russia, Crimea, Nikita Botanical Gardens, in leaf litter under common hazel (*Corylus avellana*) from a park.

#### Comparative material.

Germany: 2 ♀♀ (ZMB: 40199, holotype; ZMB: 40200, paratype) – 17. 8. 1967, Kalktuffniedermoor, südw. von Oechsen/Rhön, Gras m. Wurzeln am Westhang, 2979, 2980. Iran: 2 ♀♀ (CJH) – Guilan Province, soil sample, leg. J. Hajizadeh. Russia: 10 ♀♀, 4 ♂♂ (IZSAV) – 9. 1. 1973, Crimea, Nikita Botanical Gardens, litter under *Quercus pubescens* (labelled *Ameroseius pseudofurcatus*).

#### Published material from Slovakia.

Cerová Vrchovina Highland: Šiatorská Bukovinka Village, Šomoška Castle (Fenďa and Mašán 2009).

#### Revised material from Slovakia:

Podunajská Rovina Flatland: 1 ♀ – 30. 11. 1994, Bodíky Village (labelled as *Ameroseius pseudoplumosus* – leg. et det. S. Kalúz, unpublished but registered by the ‘Databank of Slovak Fauna’ at former http://zoology.fns.uniba.sk/dfs/system300.htm).

#### New material from Slovakia.

Horehronské Podolie Basin: 2 ♀♀, 1 ♂ – 20. 6. 2006, Zlatno Village, Zlatnianske Skalky Forest, pine forest (*Pinus sylvestris*), soil and detritus from ant-hill of *Formica* sp. (Hymenoptera, Formicidae), altitude 755 m, leg. P. Mašán. Malé Karpaty Mts.: 2 ♀♀ – 1. 5. 2005, Bratislava Capital, Karlova Ves Settlement, Mokrý Jarok Valley, oak forest (*Quercetum*) with ash (*Fraxinus* sp.), hornbeam (*Carpinus betulus*) and maple (*Acer* sp.), moss and tinder from decaying ash stump, altitude 230 m, leg. P. Mašán; 85 ♀♀, 9 ♂♂ – 8. 6. 2013, Plavecký Mikuláš Village, Deravá Skala Cave, broad-leaved deciduous forest, rocky shelter (crepuscular cave), soil detritus, altitude 380 m, leg. P. Mašán. Nitrianska Pahorkatina Wold: 1 ♀, 1 ♂ – 28. 6. 2004, Nemečky Village, Kulháň Forest, oak-beech forest (*Querco-Fagetum*), leaf litter and soil detritus, altitude 320 m, leg. J. Čarnogurský. Volovské Vrchy Hills: 2 ♀♀ – 7. 8. 2016, Kojšov Village, Turniská Forest, beech forest (*Fagion sylvaticae*), rocky shelter (crepuscular cave), leaf litter and soil detritus deposited in rocky cervices, altitude 720 m, leg. P. Mašán.

#### Remarks.

*Ameroseius furcatus* was originally described from Germany, where it was collected in a tussock of grass with roots (Karg 1971a). Karg included this species to an identification key, without adequate morphological description. The species was only very briefly diagnosed by him, and based only on two accompanying illustrations, the epistome and dorsal shield. These illustrations were partly confusing because Karg drew the epistome as a flat structure having smooth anterior margin, and the dorsal shield bearing only 24 pairs of setae. The examination of the type material of *A. furcatus* showed that the epistome is elongate and marginally serrate, and the dorsal shield possesses the normal 29 pairs of setae. Distinctive features of *A. furcatus* females include the peculiar presence of a pair of small rounded platelets between pseudo-metasternal platelets, short and rounded hyaline appendage on distal apex of movable cheliceral digit, and the insertion of JV3 on (ventri)anal shield, but they were neglected by Karg (1971a).

I believe that the inconsistent original description of *Ameroseius furcatus*, especially the absence of comments on specific ventral features and a misinterpretation of the dorsal chaetotaxy (Karg 1971a), led to description of the same species under the name *Ameroseius pseudofurcatus*. The latter has been thoroughly described and illustrated by Livshits and Mitrofanov (1975) from specimens from the Nikita Botanical Gardens, Crimea, from leaf litter under common hazel. I have not examined type specimens of that species, but I have a slide with numerous specimens (ten females, four males) from a mite collection of the Nikita Botanical Gardens, National Scientific Center, Yalta, Crimea (received from Alex Khaustov), collected in the type locality, and labelled as follows: *Ameroseius pseudofurcatus*, дуб пушистый, подстилка, гнбс (госудaрсенный никитский ботанический сaд), 9. 1. 1973 (leaf litter under downy oak, *Quercus pubescens*, State Nikita Botanical Gardens). From previously published descriptions (Karg 1971a, Livshits and Mitrofanov 1975), observations of the primary type of *A. furcatus*, and examination of conspecific material from Germany, Crimea and Slovakia I have concluded that the both above mentioned species are identical and should be considered as synonymous.

### Ameroseius gabonensis

Taxon classificationAnimaliaMesostigmataAmeroseiidae

Elsen, 1973

Ameroseius gabonensis Elsen, 1973: 753.
Ameroseius gabonensis . — Narita et al. 2015: 396.

#### Type depository.

Musée Royal de l'Afrique Centrale, Tervuren Belgium.

#### Type locality and habitat.

Democratic Republic of Congo (as Zaire), Lemba, Mayumbe, on longhorn beetle, *Ancylonotus tribulus* (Coleoptera, Cerambycidae). Cameroon and Gabon (paratypes).

#### Comparative material.

Democratic Republic of Congo: 2 ♀♀ (MRAC: 142495, holotype; MRAC: 170349, paratype) – 5. 5. 1970, Lemba, *Ancylonotus tribulus*, leg. P. Elsen. Gabon: 2 ♀♀ (MRAC: 142504, 170348, paratypes) – Oct. 1913, Libreville, *Crocisa meripes*, leg. G. Babault.

#### Remarks.

Given that the characteristics of the type specimens of *Ameroseius gabonensis* are within the range of variability found in *Ameroseius leclercqi* Elsen, 1973, I suspect *A. gabonensis* to be a synonym of *A. leclercqi*.

### Ameroseius georgei

Taxon classificationAnimaliaMesostigmataAmeroseiidae

(Turk, 1943)

Plates 17 , 18

Epicrius canestrenii (sic). — George 1906: 264.
Epicrius canistrinii (sic). — George 1906: 266.
Epicrius canestrinii . — Turk 1943: 859.
Cornubia georgii (sic) Turk, 1943: 859 (nom. n. pro Epicrius canestrinii Haller, 1881 sensu George, 1906). Incorrect synonymy with Ameroseius corbiculus (Sowerby, 1806) by Turk 1953.
Ameroseius georgei . — Turk 1953: 12.
Ameroseius imparsetosus Westerboer (in Westerboer & Bernhard, 1963: 514). **Syn. n.**
Ameroseius imparsetosus . — Karg 1971a: 231; Bregetova 1977: 150; Karg 1993: 229; Kontschán 2007: 101.

#### Type depository.

Of *Cornubia georgei* – not stated; of *Ameroseius imparsetosus* – not stated.

#### Type locality and habitat.

Of *Cornubia georgei* – United Kingdom, England, Cornwall, habitat not stated; of *Ameroseius imparsetosus* – Spain, Sierra de Ancares Mountains, moss and bark of fallen and standing old oaks.

#### Comparative material.

United Kingdom: 2 ♀♀ (BMNH: E010147155, E010147156) – 1882, Ranmore, A. D. Michael Coll., R.z. 106–107, 1930.8.25.2204–2205 (originally labelled Acarina, Gamasidae, *Epicrius*; secondary added: *Cornubia georgii*).

#### Published material from Slovakia.

Malé Karpaty Mts.: Častá Village, Červený Kameň Castle (Mašán 2001b, cited as *Ameroseius imparsetosus*).

#### New material from Slovakia.

Malé Karpaty Mts.: 2 ♀♀ – 10. 6. 2002, Bratislava Capital, zoological garden, oak-hornbeam forest (*Querco-Carpinetum*), individual collecting on wood-destroying fungus *Trametes* sp., altitude 180 m, leg. P. Mašán; 1 ♀ – 22. 5. 2013, Bratislava Capital, Železná Studienka Forest, broad-leaved deciduous forest, individual collecting on unidentified wood-decaying fungi, altitude 270 m, leg. P. Mašán; 10 ♀♀ – 20. 9. 2014, Borinka Village, broad-leaved deciduous forest, individual collecting on unidentified wood-decaying fungi, altitude 420 m, leg. P. Mašán. Podunajská Rovina Flatland: 1 ♀ – 21. 5. 1996, Bodíky Village, Kráľovská Lúka Forest, willow-poplar flood-plain forest (*Salici-Populetum*), leaf litter and soil detritus, altitude 120 m, leg. P. Mašán; 123 ♀♀, 48 ♂♂ – 5. 5. 2013, Svätý Jur Town, Panónsky Háj Forest, oak forest (*Quercus* spp.), rotting wood-destroying fungi *Meripilus giganteus* growing at base of old oak, altitude 130 m, leg. P. Mašán.

#### Remarks.

In his paper on the genus *Epicrius* from Lincolnshire in England, George (1906) re-described and illustrated three species of which two are now regarded to be members of Ameroseiidae. According to Evans (1955a), the species George believed to be *Epicrius mollis* (Kramer, 1876) is actually *Epicriopsis horridus* and what George identified as *Epicrius canestrinii* Haller, 1881 has formerly been given the new name *Cornubia georgei* by Turk (1943). Moreover, Turk (1943) proposed the new genus *Cornubia* with his new species *Cornubia ornata* as type species. Later, Turk (1953) regarded *Cornubia* as being synonymous with *Ameroseius*. At the same time, he synonymised the two apparently different species, originally included in *Cornubia* (*C. ornata* and *C. georgei*), with *Ameroseius corbiculus*. This interpretation is accepted here only in part because *C. georgei* is considered to be a distinct and reliably distinguishable species. Therefore, it is removed from a synonymy with *A. corbiculus*. George‘s original illustration in his paper (1906) is sufficiently detailed for the recognition of *C. georgei*. This species can be characterised as having dorsal setae extremely long (for example, j6 almost reaching posterior margin of idiosoma, and J2 reaching apparently beyond posterior margin of idiosoma). *Ameroseius imparsetosus*, based on a female from Spain and described by Westerboer (in Westerboer and Bernhard 1963), is in perfect agreement with what is reported by George (1906). *A. georgei* resembles also *Ameroseius elegantissimus* by Ishikawa (1984) from Japan. These two species can be easily distinguished by the number of elongate setae (11 pairs in *A. georgei*, only eight pairs in *A. elegantissimus*).

I examined three slides (BMNH.E.010147154–56) of Michael Collection deposited at the Natural History Museum, London, each bearing a specimen originally labelled *Epicrius* and additionally specified as *Cornubia georgii* (sic). The specimen from Port Garrah (on the slide BMNH.E.010147154) labelled „nymph” is unsuitable for adequate study, and definitely not conspecific with those on the remaining two slides. Other two female specimens from Ranmore are identical to one another, in very good condition to be easily recognised as *Ameroseius imparsetosus*. Unfortunately, none of available slides bears a type designation and more specific collection data, but I believe that one female belongs to the authentic specimen of George (1906) because the original illustration of the dorsum with legs given by George (1906), for his *Epicrius canistrinii* (sic), match perfectly one of the two available mounted specimens (on slide E010147156). Based on the above mentioned circumstances, a new synonymy is established between *C. georgei* and *A. imparsetosus* in present study.

### Ameroseius gillardinae

Taxon classificationAnimaliaMesostigmataAmeroseiidae

Elsen, 1973

Ameroseius gillardinae Elsen, 1973: 741.
Ameroseius gillardinae . — Narita et al. 2015: 396.

#### Type depository.

Musée Royal de l'Afrique Centrale, Tervuren Belgium.

#### Type locality and habitat.

Democratic Republic of Congo (as Zaire), Luluabourg, on digger wasp, *Sphex fumicatus* (as *Sphex umbrosus metallicus*) (Hymenoptera).

#### Comparative material.

Democratic Republic of Congo: 4 ♀♀ (MRAC: 142496, holotype; MRAC: 142508, 170343, paratypes) – 7–18. 3. 1935, Luluabourg, Sphex umbrosus var. metallicus, leg. Gillardin.

#### Remarks.

Given that the characteristics of the type specimens of *Ameroseius gillardinae* are within the range of variability found in *Ameroseius benoiti* Elsen, 1973, I suspect *A. gillardinae* to be a synonym of *A. benoiti*.

### Ameroseius halongicus

Taxon classificationAnimaliaMesostigmataAmeroseiidae

(Haitlinger, 1987)

Kleemannia halongica Haitlinger, 1987a: 361.
Kleemannia halongia (sic). — Halliday 1997: 186.
Ameroseius halongica . — Ho 2010: 172.

#### Type depository.

Museum of Natural History, Wrocław University, Poland.

#### Type locality and habitat.

Vietnam, Hongai, Halong, on unidentified butterfly (Lepidoptera).

#### Comparative material.

Vietnam: 1 ♀ (MPUV: MP-1289, syntype) – 25. 2. 1985, Halong n. Hongai, z ćmy (labelled *Kleemannia halongica*, holotyp).

#### Remarks.

The epistome of *Ameroseius halongicus* illustrated by Haitlinger (1987a) is actually a labrum. The epistome is subtriangular, with wide and obtuse central cusp, ornamented with two rows of denticles on proximal surface. Fixed digit of chelicara tridentate, teeth similar in size (two proximal teeth somewhat separate from the most distal tooth). Palptarsal claw not well observable. Genu III with nine setae (2-2/1, 2/1-1).

### Ameroseius haplocosmus

Taxon classificationAnimaliaMesostigmataAmeroseiidae

Elsen, 1973

Ameroseius haplocosmus Elsen, 1973: 745.

#### Type depository.

Musée Royal de l'Afrique Centrale, Tervuren Belgium.

#### Type locality and habitat.

Democratic Republic of Congo (as Zaire), Kasaï, Kondue, on digger wasp, *Chlorion maxillosum ciliatum* (as *Chlorion ciliatum maxillae*) (Hymenoptera).

#### Comparative material.

Democratic Republic of Congo: 1 ♀ (MRAC: 142494, holotype; published as 142498) – Kasaï, Kondue, Chlorion ciliatum var. maxillae, leg. E. Luja; 1 ♀ (MRAC: 170344, paratype) – 7–18. 3. 1935, Luluabourg, Sphex umbrosus var. metallicus, leg. Gillardin.

### Ameroseius imbellicus

Taxon classificationAnimaliaMesostigmataAmeroseiidae

Karg, 1976

Ameroseius imbellicus Karg, 1976: 538.
Ameroseius imbecillus (sic). — Karg 1994a: 117.

#### Type depository.

Museum für Naturkunde, Berlin, Germany; Hungarian Natural History Museum, Budapest, Hungary.

#### Type locality and habitat.

Chile, Santiago Province, Cuesta La Dormida, at Tiltil, meadow habitat with loamy-sandy soil, on grass roots.

#### Comparative material.

Chile: 1 ♀ (HNHM: Meso-715, holotype) – 5. 11. 1965, Tiltil, Cuesta La Dormida (Prov. Santiago), von lehmiger Erde im Dickicht in einem feucht. Tal; 1 ♀ (ZMB: 40404, paratype) – 5. 11. 1965, Tiltil, Cuesta La Dormida (Prov. Santiago), Nematodenproben von Graswurzeln von einer Wiese nahe Wald, 3001.

#### Remarks.

A species closely related to *Ameroseius lidiae* Bregetova, 1977.

### Ameroseius imitocorbiculus

Taxon classificationAnimaliaMesostigmataAmeroseiidae

Ma & Lin, 2013

Ameroseius imitocorbiculus Ma & Lin, 2013: 82.

#### Type depository.

Institute of Plant Protection, Fujian Academy of Agricultural Science, Fuzhou, China.

#### Type locality and habitat.

China, Hebei Province, Changli Town, from a tree.

### Ameroseius latofolius

Taxon classificationAnimaliaMesostigmataAmeroseiidae

Karg & Schorlemmer, 2009

Ameroseius latofolius Karg & Schorlemmer, 2009: 62.

#### Type depository.

Museum für Naturkunde, Berlin, Germany.

#### Type locality and habitat.

Ecuador, between Pifo and Papallacta, jumble of dicotyledon creeping to 2 m height on a tree (labelled litter).

#### Comparative material.

Ecuador: 1 ♀ (ZMB: 46150, holotype) – 1989, near Pifo-Papallacta, litter, 70152.

#### Remarks.

The dorsal chaetotaxy shown in the original description is partly confused by depicting a non existing setae next to vertex (see depicted s1), and by omitting two pairs of setae on mediolateral and marginal surface (z5, r4). What was shown as s1 is apparently part of the palps, strongly pressed under the vertex. Thus, I could detect the normal set of 29 pairs of the dorsal shield setae for this species.

### Ameroseius leclercqi

Taxon classificationAnimaliaMesostigmataAmeroseiidae

Elsen, 1973

Ameroseius leclercqi Elsen, 1973: 748.
Ameroseius leclercqi . — Narita et al. 2015: 396.

#### Type depository.

Musée Royal des Sciences Naturelles de Belgique, Bruxelles, Belgium; Musée Royal de l'Afrique Centrale, Tervuren Belgium (cited as „author’s collection“).

#### Type locality and habitat.

Philippines, Butuan, Mindanao, on wasp, *Lestica constricta* (Hymenoptera).

#### Comparative material.

Philippines: 1 ♀ (MRAC: 170346, paratype) – Mindanao, Butuan, *Lestica constricta*, leg. J. Leclercq.

### Ameroseius lehtineni

Taxon classificationAnimaliaMesostigmataAmeroseiidae

Huhta & Karg, 2010

Plate 19

Ameroseius lehtineni Huhta & Karg, 2010: 337.

#### Type depository.

Zoological Museum, University of Turku, Finland.

#### Type locality and habitat.

Finland, Kuusisto, Ylitalo, in old pile of sawdust under barn.

#### Comparative material.

Finland: 1 ♀ (ZMT: ACA.MES.FIN.4.151, holotype) – 6. 5. 1983, Kuusisto, Ylitalo, pile of sawdust, leg. P. T. Lehtinen.

### Ameroseius lidiae

Taxon classificationAnimaliaMesostigmataAmeroseiidae

Bregetova, 1977

Plates 20 , 21

Ameroseius lidiae Bregetova, 1977: 161.
Ameroseius lidiae . — Ho et al. 2010: 91; Kazemi and Rajaei 2013: 65; Nemati et al. 2013: 19; Khalili-Moghadam and Saboori 2014: 675; Khalili-Moghadam and Saboori 2016: 548.
Ameroseius (Ameroseius) lidiae . — Hajizadeh et al. 2013a: 150.

#### Type depository.

Zoological Institute, Russian Academy of Sciences, Saint Petersburg, Russia.

#### Type locality and habitat.

Ukraine, estuary of Dnieper River, hollow of willow tree (paratype: Tajikistan).

#### Comparative material.

Iran: 1 ♀ (CJH) – Guilan Province, olive garden, soil sample, leg. and det. J. Hajizadeh.

#### Published material from Slovakia.

Borská Nížina Lowland: Jakubov Village, Jakubovské Rybníky Fishponds (Fenďa 2005, Fenďa and Schniererová 2005). Podunajská Rovina Flatland: Číčov Village, Číčovské Mŕtve Rameno Arm (Fenďa et al. 1998, Fenďa and Schniererová 2010). Dolný Štál Village, Boheľovský Rybník Fishpond (Mašán and Krištofík 1995). Svätý Jur Town, Šúr Forest (Fenďa 2005, Švaňa et al. 2006, Fenďa and Schniererová 2010, Fenďa et al. 2011). Veľké Blahovo Village (Krištofík et al. 1993, Krištofík et al. 2001).

#### New material from Slovakia.

Borská Nížina Lowland: 2 ♀♀ – 23. 7. 1991, Jakubov Village, nest of *Serinus serinus* (Aves), altitude 145 m, leg. J. Krištofík and A. Darolová; 1 ♀ – 4. 6. 1992, Malé Leváre Village, littoral reed stand (*Phragmition*), nest of *Cygnus olor* (Aves), altitude 150 m, leg. J. Krištofík and A. Darolová; 2 ♀♀ – 27. 6. 2002, Moravský Svätý Ján Village, wet reed stand (*Phragmition*), leaf litter, altitude 150 m, leg. P. Mašán. Malé Karpaty Mts.: 1 ♂ – 24. 6. 1991, Bratislava Capital, Železná Studienka Forest, broad-leaved deciduous forest, nest of *Turdus philomelos* (Aves), altitude 250 m, leg. J. Krištofík and A. Darolová. Podunajská Rovina Flatland: 10 ♀♀ – 28. 6. 1989, Veľké Blahovo Village, fish-ponds, shore reed stand (*Phragmition*), nest of *Circus aeruginosus* (Aves), altitude 120 m, leg. J. Krištofík and A. Darolová; 16 ♀♀ – 28. 6. 1989, Veľké Blahovo Village, fish-ponds, shore reed stand (*Phragmition*), nest of *Fulica atra* (Aves), altitude 120 m, leg. J. Krištofík and A. Darolová; 14 ♀♀ – 24. 7. 1990, Veľké Blahovo Village, fish-ponds, shore reed stand (*Phragmition*), nest of *Circus aeruginosus* (Aves), altitude 120 m, leg. J. Krištofík and A. Darolová; 10 ♀♀ – 25. 6. 1991, Veľké Blahovo Village, fish-ponds, shore reed stand (*Phragmition*), nest of *Aythya ferina* (Aves), altitude 120 m, leg. J. Krištofík and A. Darolová; 8 ♀♀ – 25. 6. 1991, Veľké Blahovo Village, fish-ponds, shore reed stand (*Phragmition*), nest of *Fulica atra* (Aves), altitude 120 m, leg. J. Krištofík and A. Darolová; 4 ♀♀ – 16. 7. 1993, Veľké Blahovo Village, fish-ponds, shore reed stand (*Phragmition*), nest of *Circus aeruginosus* (Aves), altitude 120 m, leg. J. Krištofík and A. Darolová; 3 ♀♀ – 15. 8. 2000, Veľké Kosihy Village, littoral reed stand (*Phragmition*), leaf litter and soil detritus, altitude 120 m, leg. P. Mašán.

### Ameroseius longitarsis

Taxon classificationAnimaliaMesostigmataAmeroseiidae

Elsen, 1973

Ameroseius longitarsis Elsen, 1973: 743.
Ameroseius longitarsis . — Narita et al. 2015: 396.

#### Type depository.

Musée Royal de l'Afrique Centrale, Tervuren Belgium.

#### Type locality and habitat.

Democratic Republic of Congo (as Zaire), Ganda Sundi, Mayumbe, on fungus weevil, *Mecocerus rhombeus* (Coleoptera, Anthribidae).

#### Comparative material.

Democratic Republic of Congo: 1 ♀ (MRAC: 142498, holotype) – 11. 2. 1970, Ganda Sundi, *Mecocerus rhombeus*, leg. P. Elsen.

#### Remarks.

Given that the characteristics of the type specimen of *Ameroseius longitarsis* are within the range of variability found in *Ameroseius benoiti* Elsen, 1973, I suspect *A. longitarsis* to be a synonym of *A. benoiti*.

### Ameroseius longitrichus

Taxon classificationAnimaliaMesostigmataAmeroseiidae

Hirschmann, 1963

Plates 22 , 23

Ameroseius longitrichus Hirschmann (in Westerboer & Bernhard, 1963: 530).
Ameroseius longitrichus . — Karg 1971a: 231; Bregetova 1977: 152; Kaczmarek and Michalski 1988: 101; Karg 1993: 229; Ma and Lin 2006: 241; Narita et al. 2015: 395.

#### Type depository.

Not stated (holotype not designated).

#### Type locality and habitat.

Germany (from several localities, in wood detritus of spruces and firs, and in galleries of various scolytine bark beetles).

#### Published material from Slovakia.

Podunajská Rovina Flatland: Šuľany Village (not Šurany Village as originally cited) (Mašán 2001a).

#### New material from Slovakia.

Borská Nížina Lowland: 2 ♀♀ – 30. 3. 2002, Borský Svätý Jur Village, edge of mixed forest (*Pinus sylvestris*, *Robinia pseudoacacia*), sandy soil and wood detritus from colony of *Formica* sp. (Hymenoptera) situated under pine stem, altitude 170 m, leg. P. Mašán; 1 ♀ – 10. 4. 2004, Borský Svätý Jur Village, Šaštínsky Les Forest, mixed forest (*Pinus sylvestris* and *Betula* sp.), individual collecting under bark of fallen and dead birches, altitude 195 m, leg. P. Mašán; 1 ♀ – 3. 4. 2005, Tomky Village, Šaštínsky Les Forest, pine forest, individual collecting under bark of old pines (*Pinus sylvestris*), altitude 180 m, leg. P. Mašán. Burda Hills: 1 ♀ – 9. 11. 1997, Kamenica Nad Hronom Village, Kováčovské Kopce-juh Forest, oak forest (*Quercetum cerris*), leaf litter and soil detritus, altitude 330 m, leg. P. Mašán. Malé Karpaty Mts.: 2 ♂♂ – 18. 6. 1997, Častá Village, Červený Kameň Castle, beech forest (*Fagion sylvaticae*) with oak (*Quercus* sp.), individual collecting under bark, stones and pieces of wood, altitude 360 m, leg. P. Mašán; 1 ♀ – 28. 7. 1997, Bratislava Capital, Železná Studienka Forest, broad-leaved deciduous forest, individual collecting under bark, stones and pieces of wood, altitude 380 m, leg. P. Mašán. Muránska Planina Plateau: 1 ♀ – 23. 6. 2003, Muráň Village, Javorníková Dolina Valley, maple-beech forest (*Aceri-Fagetum*) with ash (*Fraxinus* sp.), rocky canyon, on wood-destroying fungus (*Daedalea quercina*), altitude 500 m, leg. P. Mašán. Podunajská Rovina Flatland: 1 ♀ – 24. 4. 1997, Šuľany Village, willow-poplar flood plain forest (*Salici-Populetum*), individual collecting under bark of old poplar trunks, altitude 120 m, leg. P. Mašán. Trnavská Pahorkatina Wold: 1 ♀ – 18. 5. 1997, Brunovce Village, Váh River (alluvium), degraded willow-poplar flood-plain forest (*Salici-Populetum*), individual collecting on wood-destroying fungus *Trametes* sp., altitude 170 m, leg. P. Mašán. Zvolenská Kotlina Basin: 1 ♀ – 19. 8. 2014, Čačín Village, oak forest with *Quercus cerris*, on wood-destroying fungus (*Daedalea quercina*), altitude 420 m, leg. P. Mašán.

#### Remarks.

The female of *Ameroseius longitrichus* was originally illustrated by Hirschmann (in Westerboer and Bernhard 1963) as having only 28 pairs instead of 29 pairs of dorsal shield setae. My examination of the specimens from various parts of the North America, identified as *A. longitrichus* by Malcolm M. Furniss, Evert E. Lindquist, or John C. Moser, and published in part by Moser and Roton (1971), Furniss et al. (1972) and Moser (1975), has revealed that these specimens represents at least two species different from *A. longitrichus*, including *Ameroseius peniophorae* De Leon, 1964, previously described from Tennessee. These two species seem to be very similar to *A. longitrichus*, but can be distinguished by some characters of dorsal and leg chaetotaxy (in *A. peniophorae*, all legs are notably thickened and shortend, and femur I and genu I bear a robust and smooth seta on medial ventral surface).

### Ameroseius macropilis

Taxon classificationAnimaliaMesostigmataAmeroseiidae

De Leon, 1964

Ameroseius macropilis De Leon, 1964: 215.

#### Type depository.

„Author’s collection“.

#### Type locality and habitat.

USA, Tennessee, Erwin, on dead strawberry plant.

#### Comparative material.

U.S.A.: 2 ♀♀ (LACM: ENT 198896) – Fall-1947, Ohio, Bowling Green, Red clover, Ray Everly & P. E. Telford (additionally labelled *Ameroseius corbicula*).

#### Remarks.

*Ameroseius macropilis* is very similar to *Ameroseius corbiculus* (Sowerby, 1806), but they can be easily distinguished by the number of opisthogastric setae expressed on soft integument (six pairs in *A. macropilis*; five pairs in *A. corbiculus*, having its JV4 absent). The reports of *A. corbiculus* given by Oatman (1971), Freitag and Ryder (1973), and Goh and Lange (1989) seem to be a misidentification of *A. macropilis*, and they should be revised.

### Ameroseius magnisetosus

Taxon classificationAnimaliaMesostigmataAmeroseiidae

(Ishikawa, 1972)

Kleemannia magnisetosa Ishikawa, 1972: 99.
Ameroseius magnisetosa . — Gu and Guo 1997: 139; Ho et al. 2010: 91. not Ameroseius magnisetosus. — Bregetova 1977: 150, 152 (= Ameroseius guyimingi Ma, 1997, misidentification).

#### Type depository.

Biological Laboratory, Matsuyama Shinonome Junior College, Matsuyama, Japan.

#### Type locality and habitat.

Japan, Yamaguchi Prefecture, Hagi, habitat stated.

### Ameroseius mariehigginsae

Taxon classificationAnimaliaMesostigmataAmeroseiidae

De Leon, 1964

Ameroseius mariehigginsae De Leon, 1964: 215.

#### Type depository.

„Author’s collection“.

#### Type locality and habitat.

USA, Washington, Lewis County, White Pass, from duff.

### Ameroseius matsudai

Taxon classificationAnimaliaMesostigmataAmeroseiidae

Ishikawa, 1977

Ameroseius matsudai Ishikawa, 1977: 182.
Ameroseius matsudai . — Ho et al. 2010: 91

#### Type depository.

Biological Laboratory, Matsuyama Shinonome Junior College, Matsuyama, Japan.

#### Type locality and habitat.

Japan, Ehime Prefecture, ôzu, Sugeta, on Japanese pine sawyer, *Monochamus alternatus* (Coleoptera, Cerambycidae).

### Ameroseius megatritosternum

Taxon classificationAnimaliaMesostigmataAmeroseiidae

Elsen, 1973

Ameroseius megatritosternum Elsen, 1973: 756.
Ameroseius megatritosternum . — Narita et al. 2015: 396.

#### Type depository.

Musée Royal de l'Afrique Centrale, Tervuren Belgium.

#### Type locality and habitat.

Democratic Republic of Congo (as Zaire), Stanleyville, on digger wasp, *Isodontia stanleyi* [as Chlorion (Isodontia) stanleyi)] (Hymenoptera).

#### Comparative material.

Democratic Republic of Congo: 1 ♀ (MRAC: 142492, holotype) – April 1915, Stanleyville, Chlorion (Isodontia) stanleyi, leg. J. Bequaert.

### Ameroseius mirus

Taxon classificationAnimaliaMesostigmataAmeroseiidae

Elsen, 1973

Ameroseius mirus Elsen, 1973: 733.
Ameroseius mirus . — Narita et al. 2015: 396.

#### Type depository.

Musée Royal de l’Afrique Centrale, Tervuren Belgium.

#### Type locality and habitat.

Democratic Republic of Congo (as Zaire), Luluabourg, on digger wasp, *Sphex fumicatus* (as *Sphex umbrosus metallicus*) (Hymenoptera).

#### Comparative material.

Democratic Republic of Congo: 4 ♀♀ (MRAC: 142493, holotype; MRAC: 142505, 170342, paratypes) – 7–18. 3. 1935, Luluabourg, Sphex umbrosus var. metallicus, leg. Gillardin.

### Ameroseius octobrevisetae

Taxon classificationAnimaliaMesostigmataAmeroseiidae

Elsen, 1973

Ameroseius octobrevisetae Elsen, 1973: 738.
Ameroseius octobrevisetae . — Narita et al. 2015: 396.

#### Type depository.

Musée Royal de l'Afrique Centrale, Tervuren Belgium.

#### Type locality and habitat.

Democratic Republic of Congo (as Zaire), Kasaï, Kondue, on digger wasp, *Chlorion maxillosum ciliatum* (as *Chlorion ciliatum maxillae*) (Hymenoptera).

#### Comparative material.

Democratic Republic of Congo: 1 ♀ (MRAC: 142497, holotype) – Kasaï, Kondue, Chlorion ciliatum var. maxillae, leg. Léonard.

### Ameroseius peniophorae

Taxon classificationAnimaliaMesostigmataAmeroseiidae

De Leon, 1964

Ameroseius peniphorae (sic) De Leon, 1964: 213.
Ameroseius peniophorae . — De Leon 1964: 213, 215.

#### Type depository.

„Author’s collection“.

#### Type locality and habitat.

USA, Tennessee, Erwin, on hyphae of white-rot fungus, *Phlebiopsis gigantea* (as *Peniophora gigantea*), on bark of log of white pine, *Pinus strobus* (Pinaceae).

#### Comparative material.

U.S.A.: 2 ♀♀ (IZSAV) – 14. 6. 1965, Elizabeth, inner bark, *Dendroctonus frontalis*, J. Moser Collection (No. 4814, 4840).

#### Remarks.

This species has been occasionally misidentified as *Ameroseius longitrichus* Hirschmann, 1963 in North America (Pielou and Verma 1968, Moser and Roton 1971, Furniss et al. 1972, Moser 1975; see also remarks under *A. longitrichus*). There are 29 pairs of setae on dorsal shield of *Ameroseius peniophorae*, not 27 pairs as stated in the original description.

### Ameroseius proteae

Taxon classificationAnimaliaMesostigmataAmeroseiidae

Ryke, 1964

Ameroseius proteae Ryke, 1964: 344.

#### Type depository.

Institute for Zoological Research, Potchefstroom University, Potchefstroom, South Africa.

#### Type locality and habitat.

South Africa, Grabouw, on dry flower of sugarbush, *Protea repens* (as *Protea mellifera*) (Proteaceae).

### Ameroseius renatae sp. n.

Taxon classificationAnimaliaMesostigmataAmeroseiidae

http://zoobank.org/39C89C41-2610-4E1B-90DC-33E58F258E0C

Figure 2 , Plates 24 , 25 , 76G

#### Type material.

Slovakia, Považský Inovec Mts.: 1 ♀ (IZSAV, holotype) – 13. 10. 2012, Hrádok Village, Hrádocká Dolina Valley, edge of broad-leaved deciduous forest, decaying wood-destroying fungi, altitude 290 m, leg. P. Mašán; 4 ♀♀ (IZSAV, paratypes), with the same collection data as in holotype.

#### Diagnosis.

Dorsal shield strongly sculptured, scrobiculate, with a series of interconnecting ridges. Dorsal setae of different length, relatively thin and variously curved; setae J2 long, with tips reaching clearly beyond bases of following setae J4; setae Z2 notably longer than Z1 and Z3. Pseudo-metasternal platelets relatively well developed, subequal or slightly larger than metapodal platelets. Opisthogastric soft integument with six pairs of setae; setae JV5 lengthened, with tips reaching anal aperture.

#### Description.

Female. Idiosoma oval to egg-shaped, narrowed anteriorly, 405–460 μm long and 270–325 μm wide. Dorsal shield strongly sclerotised and coarsely ornamented, scrobiculate, covering whole dorsal surface, and bearing 29 pairs of setae; vertex with anterior margin denticulated. Dorsal setae well differing in length, relatively thin, curved, sparsely covered by very minute spines, inserted in small basal papilla-like tubercles; setae j1–j4, z5, z6, Z1, Z3, s1, s2, S2, S3, and r3–r5 notably shorter than other dorsal shield setae; setae j1 lanceolate and conspicuosly spinate; posterior dorsocentral setae long, J2 and J4 with tips reaching beyond bases of J4 and Z5, respectively. The length of some selected dorsal setae as follows: j5 83–92 μm, j6 92–102 μm, J2 105–118 μm, z5 25–30 μm, z6 29–40 μm, Z1 38–46 μm, Z2 78–92 μm, Z3 45–55 μm. Sternal shield subquadrate, 62–70 μm in length and width (at level of st2), reticulate on surface, with two pairs of setae (st1, st2). Pseudo-metasternal platelets relatively large, rounded, bearing a pair of sternal setae (st3). Metasternal setae (st4) on soft integument. Epigynal shield 70 μm wide, oblong, with reticulate pattern and a pair of genital setae (st5). Postgenital sclerites present, partly fused behind posterior margin of epigynal shield. Metapodal platelets small, suboval. Peritremes and peritrematal shields normally developed. Ventrianal shield 125–140 μm wide and 100–115 μm long, almost hexagonal, well reticulate, having three circum-anal setae and anus in its posteromedial portion. Ventrally situated setae smooth, needle-like, about 25 μm long, except JV5, markedly elongated, 60–67 μm in length. Epistome subtriangular, with rounded apex. Fixed digit of chelicera with three small teeth on proximal masticatory area. Other gnathosomal structures and leg chaetotaxy typical of genus.

#### Etymology.

The new species is named in honour of my dear older sister.

### Ameroseius sculptilis

Taxon classificationAnimaliaMesostigmataAmeroseiidae

Berlese, 1916

Plates 26 , 27

Ameroseius sculptilis Berlese, 1916a: 47.
Ameroseius pulcher Westerboer (in Westerboer & Bernhard, 1963: 505). Synonymy by Bregetova (1977).
Ameroseius pulcher . — Karg 1971a: 234; Ishikawa 1972: 102; Karg 1993: 232.
Ameroseius sculptilis . — Bregetova 1977: 152; Castagnoli and Pegazzano 1985: 372; Narita et al. 2015: 395.
Kleemannia sculptilis . — Haitlinger 1991: 273.
Ameroseius (Ameroseius) sculptilis . — Hajizadeh et al. 2013a: 150.

#### Type depository.

Of *Ameroseius sculptilis* – Istituto Sperimentale per la Zoologia Agraria, Firenze, Italy; of *Ameroseius pulcher* – Zoologischen Staatssammlung München, Germany (originally not stated; holotype not designated).

#### Type locality and habitat.

Of *Ameroseius sculptilis* – Italy, Vallombrosa, in moss; of *Ameroseius pulcher* – Germany, Erlangen, in rotting grass.

#### Comparative material.

Germany: 3 ♀♀ (ZMB: 41124–41126) – Jan. 1986, Einsendung, LPG(P), Frühgemüse-zentrum Dresden, Gutkenpflanz. (Strohballen Kult.), 2990–2991, 2993 (labelled *Ameroseius pulcher*). Italy: 1 ex. (ISZA: 169/3, holotype) – Vallombrosa, musco; 1 ♀ (ISZA: 190/19) – Firenze, Giardino R. Stazione.

#### Published material from Slovakia.

Borská Nížina Lowland: Láb Village (Ambros 1989). Hronská Pahorkatina Wold: Malá Mužla Village (Várfalvyová et al. 2010).

#### New material from Slovakia.

Podunajská Rovina Flatland: 1 ♀ – 24. 7. 1990, Veľké Blahovo Village, fish-ponds, shore reed stand (*Phragmition*), nest of *Circus aeruginosus* (Aves), altitude 120 m, leg. J. Krištofík and A. Darolová.

#### Remarks.

There are two slides of this species in the Berlese Collection in Florence, only one of them (169/3) is labelled „tipico”. Unfortunately, the holotype specimen is wholly unsuitable for study, and practically the same can be stated for the female mounted onto the second slide (190/19), reported from Florence. Bregetova (1977) considered *Ameroseius sculptilis* to be identical to *Ameroseius pulcher* Westerboer, 1963, and her interpretation is accepted in this paper. I examined one quite well preserved female on a slide in the Hirschmann/Willmann Collection in Munich labelled „*Ameroseius pulcher* n. sp.”. Unfortunately, this available specimen (slide) bears no type designation and specific collection data, but without doubt it belongs to the original series of Westerboer (see remarks under *Ameroseiella macrochelae*).

### Ameroseius sternalis

Taxon classificationAnimaliaMesostigmataAmeroseiidae

Bhattacharyya & Kheto, 2015

Ameroseius sternalis Bhattacharyya & Kheto, 2015: 298.

#### Type depository.

Department of Zoology, Y. S. Palpara Mahavidyalaya, Palpara, Midnapore, West Bengal, India; National Zoological Collection, Zoological Survey of India, Calcutta, India.

#### Type locality and habitat.

India, West Bengal, Sitala, nest of lesser banded hornet, *Vespa affinis* (Hymenoptera).

### Ameroseius stultus

Taxon classificationAnimaliaMesostigmataAmeroseiidae

Karg, 1996

Ameroseius stultus Karg, 1996: 153.

#### Type depository.

Museum für Naturkunde, Berlin, Germany.

#### Type locality and habitat.

Pacific Ocean Region, New Caledonia, Isle of Pines, habitat and substrate unspecified.

#### Comparative material.

New Caledonia: 1 ♀ (ZMB: 45236, holotype) – 24. 2. 1977, Ile de Pins, 6696, leg. J. Balogh.

#### Remarks.

Karg (1996) illustrated this species as having only 28 pairs of dorsal setae on the dorsal shield. I could detect 29 setal pairs (including r4, missing in the illustration of the original description), leg chaetotaxy typical of the genus, a prominent poroid structure on each peritrematal shield, and slightly smaller sternal shield than depicted in the original description.

### Ameroseius submagnisetosus

Taxon classificationAnimaliaMesostigmataAmeroseiidae

Ma & Lin, 2005

Ameroseius submagnisetosus Ma & Lin, 2005: 77.

#### Type depository.

Institute of Plant Protection, Fujian Academy of Agricultural Science, Fuzhou, China.

#### Type locality and habitat.

China, Henan Province, Luanchuan County, Longgu Bend, under fallen leaves.

### Ameroseius taoerhensis

Taxon classificationAnimaliaMesostigmataAmeroseiidae

Ma, 1995

Ameroseius taoerhensis Ma, 1995: 93.
Ameroseius taoerhensis . — Ma 2002: 309.

#### Type depository.

National Base of Plague and Brucellosis Control, Baicheng, China.

#### Type locality and habitat.

China, Jilin Province, Baicheng, under decomposed bark of pine tree.

#### Remarks.

A species closely related with *Ameroseius longitrichus* Hirschmann, 1963, if not identical.

### Ameroseius ulmi

Taxon classificationAnimaliaMesostigmataAmeroseiidae

Hirschmann, 1963

Plate 28

Ameroseius ulmi Hirschmann (in Westerboer & Bernhard, 1963: 498).
Ameroseius ulmi . — Karg 1971a: 234; Bregetova 1977: 161; Karg 1993: 231; Nemati et al. 2013: 20; Narita et al. 2015: 395; Ma and Lin 2016: 15.

#### Type depository.

Not stated.

#### Type locality and habitat.

Germany, München, Englischer Garten, in gallery of bark beetle, *Scolytus scolytus* (Coleoptera, Curculionidae), on elm tree (*Ulmus* sp.).

#### New material from Slovakia.

Malé Karpaty Mts.: 1 ♀ – 7. 4. 1995, Bratislava Capital, Železná Studienka Forest, broad-leaved deciduous forest, individual collecting on wood-destroying fungus (*Trametes* sp.), altitude 350 m, leg. P. Mašán. Podunajská Rovina Flatland: 1 ♀ – 6. 10. 2012, Svätý Jur Town, Šúr Forest, forest steppe with oak (*Quercus* sp.), unidentified decaying wood-destroying fungi on oak stem in soil detritus, altitude 130 m, leg. P. Mašán. Považský Inovec Mts.: 1 ♀ – 12. 7. 1997, Hrádok Village, Hrádocká Dolina Valley, oak forest (*Quercetum*) with beech (*Fagus sylvatica*), individual collecting under bark, stones and pieces of wood, altitude 370 m, leg. P. Mašán; 3 ♀♀ – 13. 10. 2012, Hrádok Village, Hrádocká Dolina Valley, edge of broad-leaved deciduous forest, decaying wood-destroying fungi, altitude 290 m, leg. P. Mašán.

#### Remarks.

Hirschmann (in Westerboer and Bernhard, 1963) described and illustrated the opisthogastric surface of this species as having only five instead of six pairs of setae (JV4 were erroneously omitted).

### Ameroseius variolarius

Taxon classificationAnimaliaMesostigmataAmeroseiidae

Ishikawa, 1972

Ameroseius variolarius Ishikawa, 1972: 101.

#### Type depository.

Biological Laboratory, Matsuyama Shinonome Junior College, Matsuyama, Japan.

#### Type locality and habitat.

Japan, Tokushima Prefecture, Ishii, habitat stated.

#### Comparative material.

Japan: 1 ♀ (CKI, paratype) – 12. 4. 1969, Tokushima, leg. M. Sakai.

### Ameroseius vietnamensis

Taxon classificationAnimaliaMesostigmataAmeroseiidae

Micherdziński, 1965

Amesoseius (sic) vietnamensis Micherdziński, 1965: 17.
Amersoseius (sic) vietnamensis. — Micherdziński 1965: 21.
Ameroseius vietnamensis . — Ho et al. 2010: 91; Ma and Lin 2013: 84.

#### Type depository.

Zoological Museum, Jagiellonian University, Krakow, Poland; Zoological Department, University of Hanoi, Vietnam.

#### Type locality and habitat.

Vietnam, Cha-Pa, SW from Lao-Kay (at Geophysical Station), habitat not specified (mould in rocky cavity, leaf litter, dry and moist moss).

### Ameroseius womersleyi

Taxon classificationAnimaliaMesostigmataAmeroseiidae

, new name Ameroseius ornatus Womersley, 1956a: 547. Junior secondary homonym.

Ameroseius ornatus . — Halliday 1997: 182; Kazemi and Rajaei 2013: 65; Nemati et al. 2013: 19. not Cornubia ornata Turk, 1943: 859. Synonymy with Ameroseius corbiculus (Sowerby, 1806) by Turk (1953).

#### Type depository.

South Australian Museum, Adelaide, Australia.

#### Type locality and habitat.

Australia, Tasmania, from strawberry plants.

#### Comparative material.

Australia: 1 ♀ (SAMA: ARA73140, holotype) – June 1952, Burnley, Victoria, on strawberry leaves ex Tasmania, det. H. Womersley.

#### Remarks.

*Ameroseius ornatus* Womersley, 1956 is a junior secondary homonym of *Cornubia ornata* Turk, 1943, *Cornubia* being a junior synonym of *Ameroseius*. Turk (1953) synonymised *Cornubia ornata* Turk, 1943, under *Ameroseius corbiculus* (Sowerby, 1806). Since *C. ornata* is the type species of *Cornubia* Turk, 1943, and *A. corbiculus* is the type species of *Ameroseius* Berlese, 1904, the above arrangement naturally follows. I hereby rename *Ameroseius ornatus* Womersley, 1956 *Ameroseius womersleyi* nom. n. in recognition of Herbert Womersley and his work on the taxonomy of the mites, including Ameroseiidae.

I examined the holotype female of this species and could detect the normal complement of 29 pairs of setae on dorsal shield (not stated in original description), including j1 which are missing in the holotype (together with a small part of vertex). In *Ameroseius womersleyi*, st3 are on soft integument, opisthogastric soft integument presumably has six pairs of setae (JV4 present), and fixed digit of chelicera possesses three well observable teeth on proximal masticatory area of which two proximal are slightly smaller and somewhat separate from the most distal tooth. The recent diagnosis given by Halliday (1997) does not require additional amendment.

### Genus Asperolaelaps

Taxon classificationAnimaliaMesostigmataAmeroseiidae

Womersley, 1956

Asperolaelaps Womersley, 1956a: 534. Type species: Asperolaelaps rotundus Womersley, 1956, by original designation.

#### Diagnosis (adults).

Soft striate integument delicately incrusted with sclerotic tubercles. Dorsal shield widely oval, not completely covering dorsal surface, reticulate or striate, with 6–7 pairs of distinct protuberances (each bearing a seta), and 29 pairs of setae differing in length and form; setae mounted on protuberances (z4, z5, s5, Z1, Z3, S5, and sometimes z2) markedly longer, thicker, and more heavily pilose than the other setae on the shield. Dorsal shield setae needle-like to lanceolate, smooth, pilose or serrate, the thicker setae densely plumose; sexual dimorphism of dorsal chaetotaxy not developed. In female, sternal setae on sternal shield (st1, st2), soft integument (st3, st4) and epigynal shield (st5). Female with anal shield bearing only three circum-anal setae, male with expanded ventrianal shield capturing some opisthogastric setae. Peritrematal shields and peritremes well developed: anterior end of peritremes reaching or overlapping bases of j1; in male, peritrematal shields well expanded beyond coxae IV and abutting the ventrianal shield. Opisthogastric soft integument with six pairs of setae in female. Corniculi stout, weakly sclerotised, well separate and parallel, with undivided apex having one or two denticles. In female, cheliceral shafts relatively elongate, slender; fixed digit with three prominent sharp teeth (two proximal teeth slightly separated from the other, medial tooth), and a special bilobed tooth close to terminal hook (Plate 76F); movable digit only with tiny subapical denticle; cheliceral digits almost straight, not conspicuously hooked distally, without hyaline petal-like appendages. Male spermatodactyl relatively short, directed forward. Palptarsal apotele three-tined. Tibia IV with two posterolateral setae. Tarsi I–IV each with well developed empodium and claws. Insemination apparatus with spermathecal ducts separated.

#### Remarks.

The genus *Asperolaelaps* was proposed by Womersley (1956a), with *Asperolaelaps rotundus* as its type species, but it has been considered a synonym of *Neocypholaelaps* Vitzthum, 1942, for example by Domrow (1979), Halliday (1997), and Moraes and Narita (2010). Womersley (1956) originally placed it in the family Neoparasitidae but Domrow (1979) re-examined the type species observing the normal complement of 29 pairs of dorsal shield setae instead of the reduced number illustrated by Domrow, and some further features typical of ameroseiid species.

In this paper, the genus is removed from synonymy with *Neocypholaelaps*, re-diagnosed, and ressurected to accommodate one further species from Australasian Region, namely *Asperolaelaps sextuberculi* (Karg, 1996). None of these species have been found in association with flowers and their pollinators, as opposed to species of *Neocypholaelaps*, the closest genus. Separate systematic position of *Asperolaelaps* species can better clarify the concept of *Neocypholaelaps*. *Asperolaelaps rotundus* and *A. sextuberculi* are considered not to belong to *Neocypholaelaps* because their diagnostic character states are based on the features inconsistent with *Neocypholaelaps*: (1) fixed digit of chelicera with three sharp teeth in proximal-medial part and a wide bilobed subapical tooth (edentate in *Neocypholaelaps*); (2) chelicera without hyaline appendages (appendages developed in *Neocypholaelaps*); (3) corniculi well separate and parallel to each other (adjacent and convergent in *Neocypholaelaps*); (4) palptarsal claw three-tined (two-tined in *Neocypholaelaps*); (5) some dorsal setae mounted on protuberances, and some with unusual position on the dorsal shield: e.g., Z-series setae with insertions well separate from those in central J-rows and more adjacent to those in marginal S-rows (dorsal shield smooth, with dorsal setae otherwise situated in *Neocypholaelaps*); (6) sexual dimorphism of dorsal setation absent (present in *Neocypholaelaps*); (7) tibia IV with two posterolateral setae (only one posterolateral seta in *Neocypholaelaps*). The dentation of cheliceral digits (together with other characters of gnathosoma) shows important generic difference between *Asperolaelaps* and *Neocypholaelaps*, probably due to their specific mode of life and different feeding habits. On the contrary, except for items 1, 5 and 7, the above mentioned character states are well consistent with the genus *Ameroseius*. *Asperolaelaps* represents a group of species with *Neocypholaelaps*-like venter, *Ameroseius*-like gnathosoma, and specific dorsal chaetotaxy, cheliceral dentation and tibial setation of legs IV.

Examination of the type specimens of *Asperolaelaps rotundus* and *Asperolaelaps sextuberculi* confirmed their validity and specific features. They are quite similar species originally reported from the same biogeographic realm, namely from Australia and New Caledonia, respectively. Female of *A. rotundus* differs from *A. sextuberculi* by having three setae on femur of leg I conspicuously thickened and shortened, conical (pl2, pv1 and pv2; pl2 unusually displaced close to pv2), and by the peritremes with anterior tips touching each other. In *A. sextuberculi*, femur I is relatively narrower and shorter than in previous species, having only normally formed setae, and anterior ends of peritremes separated by insertions of j1.

### Asperolaelaps rotundus

Taxon classificationAnimaliaMesostigmataAmeroseiidae

Womersley, 1956

Plate 76F

Asperolaelaps rotundus Womersley, 1956a: 534.
Neocypholaelaps rotundus . — Domrow 1979: 104; Halliday 1997: 197; Moraes and Narita 2010: 42; Hajizadeh et al. 2013a: 150; Nemati et al. 2013: 21.
Sertitympanum rotundus . — Kazemi and Rajaei 2013: 67.

#### Type depository.

South Australian Museum, Adelaide, Australia; Queensland Institute of Medical Research, Brisbane, Australia.

#### Type locality and habitat.

Australia, Queensland, Brisbane, Brookfield, on cards.

#### Comparative material.

Australia: 1 ♀ (SAMA: ARA73140, holotype) – 19. 1. 1951, Brookfield, Brisbane, coll. on card, leg. E. H. Derrick.

#### Remarks.

Genu and tibia of legs III bear one posterolateral seta, but tibia IV has two posterolateral setae.

### Asperolaelaps sextuberculi

Taxon classificationAnimaliaMesostigmataAmeroseiidae

(Karg, 1996) comb. n.

Ameroseius sextuberculi Karg, 1996: 154.

#### Type depository.

Museum für Naturkunde, Berlin, Germany.

#### Type locality and habitat.

Pacific Ocean Region, New Caledonia, Koumac, in a cave.

#### Comparative material.

New Caledonia: 4 ♀♀, 1 ♂ (ZMB: 45259, female holotype; ZMB: 45260, paratype; ZMB: 45261, allotype; ZMB: 45267, paratypes) – 15. 2. 1977, Koumac, höhle, 6731–6733, 2653, leg. J. Balogh.

#### Remarks.

In the original description, Karg (1996) did not comment the number of the dorsal shield setae. There are 29 pairs of the dorsal shield setae on his quite detailed illustration of the dorsum. Examination of the type specimens showed that the dorsal chaetotaxy is partly confused by depicting a non existing pair between r3 and r4, and by omitting one setal pair on medial surface (z6). In addition, I could find six pairs of opisthogastric setae (only five pairs shown in the original illustration). Genu III with one posterolateral seta (as in *Ameroseius*), but tibiae III and IV with two posterolateral setae (as in *Kleemannia*).

### Genus Brontispalaelaps

Taxon classificationAnimaliaMesostigmataAmeroseiidae

Womersley, 1956

Brontispalaelaps Womersley, 1956a: 533. Type species: Brontispalaelaps leveri Womersley, 1956, by original designation.
Brontispalaelaps . — Evans 1963a: 229.

#### Diagnosis (adults).

Dorsal shield well sclerotised and ornamented, with 27–29 pairs of setae. Dorsal setae not conspicuously thickened or otherwise modified. In female, sternal setae on sternal shield (st1, st2, st3), complex metasternal-endopodal platelet (st4), and epigynal shield (st5). Epigynal shield relatively large, well oblong, and with posterior margin widely abutting ventrianal shield. In both sexes, posteroventral region with reduced number of four pairs of opisthogastric setae on ventrianal shield (JV2, JV3, ZV2) or on soft integument (JV5); setae JV1 and JV4 absent. Ventrally inserted setae including JV5 short, smooth and needle-like. Corniculi apically bifid. Fixed digit of chelicera with eight subequal denticles on masticatory area; spermatodactyl relatively long, apparently longer than movable digit. Epistome subtriangular, with one or two sharply pointed cusps. Palptarsal apotele three-tined. Genua II–III without ventral setae, genu III with two anterolateral and one posterolateral setae, tibia IV with two anterolateral and two posterolateral setae. Tarsi I–IV each with normal empodium and claws.

#### Remarks.

The genus comprises only two described species. They are reported from the Solomon Islands, Papua New Guinea, Australia (Queensland) and Thailand (Womersley 1956a, Halliday 1997, Silva et al. 2014), as phoretic associates of phytophagous insects (see below).

### Brontispalaelaps leveri

Taxon classificationAnimaliaMesostigmataAmeroseiidae

Womersley 1956

Brontispalaelaps leveri Womersley, 1956a: 533.
Brontispalaelaps leveri . — Silva et al. 2014: 710.

#### Type depository.

South Australian Museum, Adelaide, Australia.

#### Type locality and habitat.

Pacific Ocean Region, Solomon Islands, Guadalcanal, Tenaru, on coconut leaf beetle, *Brontispa longissima* (as *Brontispa froggatti*) (Coleoptera, Chrysomelidae), found in coconut leaves.

#### Remarks.

Some important amendments to the original description of *Brontispalaelaps leveri* were carried out by Halliday (1997) and Silva et al. (2014). This species was described from specimens associated with the chrysomelid beetle *Brontispa froggatti* Sharp, 1904 [= *Brontispa longissima* (Gestro, 1885)] from the Solomon Islands and Papua New Guinea. It is remarkable that the type specimens of *Ameroseius crassipes* were collected from the same host beetle in Australia. For that reason there may be some speculation about conspecificity of *A. crassipes* and *B. leveri*, the type species of the genus *Brontispalaelaps*. The conspecificity of both species should be carefully checked by further study once more, despite of the fact that Lindquist’s examination of the type specimens has shown that *A. crassipes* belongs to an unspecified genus of Ologamasidae (Halliday 1997).

### Brontispalaelaps marianneae

Taxon classificationAnimaliaMesostigmataAmeroseiidae

Halliday, 1997

Brontispalaelaps marianneae Halliday, 1997: 184.
Brontispalaelaps mariannae (sic). — Halliday 1997: 186.

#### Type depository.

Australian National Insect Collection, CSIRO, Canberra, Australia.

#### Type locality and habitat.

Australia, Queensland, McIlwraith Range, Golden Nugget Creek, on wings of undescribed species of phycitine pyralid moth (Lepidoptera).

### Genus Epicriopsis

Taxon classificationAnimaliaMesostigmataAmeroseiidae

Berlese, 1916

Epicriopsis Berlese, 1916a: 34. Type species: Gamasus horridus Kramer, 1876, by original designation.
Epicriopsis . — Evans 1963a: 229; Karg 1971a: 223; Bregetova 1977: 167; Karg 1993: 220.

#### Diagnosis (adults).

Dorsal shield heavily sclerotised and ornamented, with a pattern of conspicuous tubercles; these tubercles star-like, polygonal, sometimes reduced in size (in *Epicriopsis walteri*) or modified into small spines arranged into a rows (in *Epicriopsis atuberculatus*). Some dorsal shield setae (6–9 pairs) conspicuous, thickened, extremely long, pilose, and much longer than some of the shortest setae; dorsal shield with 22–28 pairs of setae. In female, sternal setae on sternal shield (st1, st2), soft integument (st3, st4), and epigynal shield (st5); setae st3 rarely on small and rounded pseudo-metasternal platelets (in *E. atuberculatus*). Female with anal shield, male with ventrianal shield bearing 3–4 pairs of opisthogastric setae (JV1 on or off the shield). Opisthogastric soft integument with 5–6 pairs of setae in female (JV1–JV3, JV5, ZV2; JV4 present or absent), and five pairs of setae in male (JV4 always absent). Corniculi unsclerotised, undivided, stout, with distal extension and obtuse apex; the apex sometimes with denticles. Fixed digit of chelicera with 3–5 teeth on proximal masticatory area (normally with three teeth, but with 4–5 teeth in species with sharply pointed epistome); at least one of cheliceral digits (fixed digit) with membranous structures. Palptarsal apotele usually three-tined. Genu III, and tibiae III–IV with two anterolateral and one posterolateral setae. Tarsi I–IV each with empodium and claws.

#### Remarks.

The genus *Epicriopsis* was previously known from 14 named species (*atuberculatus*, *baloghi*, *berlesei*, *horridus*, *hungaricus*, *jilinensis*, *langei*, *linzhiensis*, *mirabilis*, *palustris*, *rivus*, *stellata*, *suedus* and *walteri*), occurring mostly in leaf litter, soil detritus, raw humus, fungi and moss, in humid habitats in Europe (Karg 1971a, 1971b, 1993; Kandil 1978), Asia (Ishikawa 1972, Iavorschi 1995, Ma 2002, Hajizadeh et al. 2013b, Ma and Lin 2016), North America (Farrier and Hennessey 1993), South America (Marticorena and Berrío 2014, Narita and Moraes 2016) and Australia (Halliday 1997). The genus often comprises strongly hygrophilous species found in swamp areas, inundation zones, river beds and floodplain forests.

In the checklist below, ten species of this genus are recognised as valid. Among them, now I recognise five species occurring in Europe (*horridus* = *berlesei*, *hungaricus*, *mirabilis* = *rivus* syn. n., *palustris* = *baloghi* syn. n. and *langei* syn. n., and *suedus*), with the new synonymies proposed after my examination of the type specimens and specimens collected in Slovakia. For specific remarks to the individual *Epicriopsis* species and their synonyms see the checklist below.

The dorsal chaetotaxy of individual species occurring in Europe is not adequately described up to now. The two species (*rivus* and *suedus*) described by Karg (1971a) are known only on the base of illustrations and very short description as a part of an identification key. The dorsal shield bears an uncomplete set of setae in the illustrations of *Epicriopsis horridus*, *Epicriopsis rivus* and *Epicriopsis suedus* by Karg (1971a). Therefore, I carefully checked the number of dorsal shield setae in available *Epicriopsis* species. *Epicriopsis hungaricus*, *Epicriopsis palustris* and *Epicriopsis mirabilis* (= *E. rivus*) possess 24 pairs of setae (j1–j6, J2, J4, z2, z4, z5, Z1, Z3, Z5, s1, s2, s4–s6, S3–S5), *E. horridus* 23 pairs of setae (j6 absent in comparison with previous three species), and *E. suedus* 28 pairs of setae (s6 absent in comparison with normal complement of dorsal setae found in most of Ameroseiidae).

#### Key to species of *Epicriopsis* occurring in Europe (adults)

Partial keys to species of *Epicriopsis* may be found in Karg (1971a, 1971b, 1993), Bregetova (1977), and Kandil (1978). The most complete key is that of Narita and Moraes (2016), based on adult females of the world species (not including *Epicriopsis mirabilis*). A new key to the genus is needed to introduce new differential characters and include only valid species and those exclusively reported from Europe. I found that the setation of several leg segments is unstable and relatively highly variable in studied species from Europe, namely *Epicriopsis horridus*, *Epicriopsis hungaricus*, *E. mirabilis*, *Epicriopsis palustris* and *Epicriopsis suedus*. All these mentioned species can be reliably separated only with the help of leg chaetotaxy (see Table 1 and 2).

**Table:** 

1	Dorsal shield with 28 pairs of setae (s6 absent); setae j5 relatively short, not conspicuously thickened and lengthened as some other dorsal setae, with tips hardly reaching to bases of J2; genu I with three and genu III with two ventral setae	***Epicriopsis suedus* Karg, 1971** (Plates 34, 76C)
–	Dorsal shield with 23–24 pairs of setae (r2, r3, z6, Z2, S2, and sometimes j6 absent); setae j5 conspicuously thickened and lengthened, reaching apparently beyond bases of J2; genu I or genu III with different ventral setation (av2 of genu I or pv1 of genu III absent)	**2**
2	Area between lengthened j5 and J2 with one pair of setae (z5 present, j6 absent); genu IV with eight setae of which four dorsal (pd3 absent)	***Epicriopsis horridus* (Kramer, 1876)** (Plate 29)
–	Area between lengthened j5 and J2 with two pairs of setae (z5 and j6 present); genu IV with nine setae of which five dorsal (pd3 present)	**3**
3	Setae z5 and j6 relatively longer: z5 with tips reaching beyond bases of j6, j6 reaching beyond bases of J2; genu I and tibia I each with 11 setae of which two ventral (av2 absent); genu III with nine setae of which two ventral (pv1 present); epistome pointed; fixed digit of chelicera with four teeth, except terminal hook	***Epicriopsis mirabilis* Willmann, 1956** (Plates 31, 32)
–	Setae z5 and j6 relatively shorter: z5 and j6 with tips never reaching insertions of j6 and J2, respectively; genu I and tibia I each with 12 setae of which three ventral (av2 present); genu III with eight setae of which one ventral (pv1 absent); epistome rounded; fixed digit of chelicera with three teeth, except terminal hook	**4**
4	Setae j6 more slender, needle-shaped; genu II with 11 setae of which two ventral (pv1 present)	***Epicriopsis palustris* Karg, 1971** (Plate 33)
–	Setae j6 shortened and thickened, spine-shaped; genu II with ten setae of which one ventral (pv1 absent)	***Epicriopsis hungaricus* Kandil, 1978** (Plate 30)

### Epicriopsis atuberculatus

Taxon classificationAnimaliaMesostigmataAmeroseiidae

Narita & Moraes, 2016

Epicriopsis atuberculatus Narita & Moraes, 2016: 478.

#### Type depository.

Departamento de Entomologia e Acarologia, Escola Superior de Agricultura “Luiz de Queiroz”, Universidade de São Paulo, Piracicaba, São Paulo, Brazil.

#### Type locality and habitat.

Brazil, Minas Gerais State, Congonhas Town – leaves of macaúba palm, *Acrocomia aculeata* (Arecaceae), from a patch of Atlantic Forest.

#### Comparative material.

Brazil: 2 ♀♀ (ESALQ: T-MZLQ 3305 C=7735, holotype; ESALQ: T-MZLQ 3306 C=7735, paratype) – 11. 8. 2014, Congonhas – MS, Ex: *Acrocomia aculeata* (macaúba), leg. R. V. Veloso.

#### Remarks.

The number of the dorsal setae is different in both adult stages: 25 pairs in females, and 27 pairs in males (Narita and Moraes 2016). This fact is unusual in Ameroseiidae and it should be carefully checked again in newly collected specimens.

### Epicriopsis horridus

Taxon classificationAnimaliaMesostigmataAmeroseiidae

(Kramer, 1876)

Plate 29

Gamasus horridus Kramer, 1876: 82.
Epicrius mollis . — Haller 1881: 190 (misidentification by Berlese 1916a); Berlese 1887a: 40/9 (misidentification by Berlese 1916a); Oudemans 1903: 87 (misidentification by Oudemans 1939a); George 1906: 265 (misidentification by Evans 1955a); Turk 1943: 856 (misidentification by Evans 1955a).
Hypoaspis mollis . — Oudemans 1903: 87, and Oudemans 1904: 84 (both misidentification by Oudemans 1939a); Turk 1943: 856 (misidentification by Evans 1955a).
Epicriopsis horrida . — Berlese 1916a: 48.
Epicriopsis berlesei Oudemans, 1939a: 198 (new name for Epicrius mollis sensu Berlese, 1887). Synonymy by Karg (1971a, 1971b).
Epicriopsis horridus . — Karg 1971a: 225; 1971b: VI/7; Bregetova 1977: 169; Kandil 1978: 170; Lapiņa 1988: 38, 176; Karg 1993: 221; Hajizadeh et al. 2013a: 150; Kazemi and Rajaei 2013: 66; Nemati et al. 2013: 20; Khalili-Moghadam and Saboori 2014: 674; Narita and Moraes 2016: 482. not Epicriopsis horridus. — Evans and Till 1979: 230 (misidentification of Epicriopsis palustris); Iavorschi 1995: 61 (misidentification of Epicriopsis palustris); Kalúz 2005: Pl. 2, and Kalúz 2007: fig. 6 (plagiarism of original figure of Epicriopsis langei, a species considered here to be conspecific with Epicriopsis palustris).

#### Type depository.

Of *Gamasus horridus* – not stated; of *Epicriopsis berlesei* – National Museum of Natural History, Naturalis Biodiversity Center, Leiden, Netherlands.

#### Type locality and habitat.

Of *Gamasus horridus* – Germany, Thuringia, Scheusingen, in litter; of *Epicriopsis berlesei* – locality not specified (Germany, Borkum Island; Italy, San Remo; France, Sucy-en-Brie), in rotting leaves.

#### Comparative material.

Germany: 1 ♀ (RMNH: ACA.P.4063) – Aug. 1900, Eil. Borkum, leg. Prof. Oskar Schneider (labelled Nph II, *Epicriopsis berlesei*); 1 ♀ (IZSAV) – 25. 4. 2007, Bavarian Prealps Mts., Flintsbach am Inn, altitude 800 m, spruce forest, wet soil detritus, leg. P. Mašán.

#### Published material from Slovakia.

Borská Nížina Lowland: Brodské Village (Kalúz and Majzlan 2009). Jakubov Village, Jakubovské Rybníky Fishponds (Fenďa 2005, Fenďa and Schniererová 2005). Bukovské Vrchy Hills: Nová Sedlica Village, Dolina Zbojského Potoka Valley; Ruský Potok Village, Veľký Bukovec Mt., Borsučina Forest (Fenďa and Mašán 2003). Cerová Vrchovina Highland: Chrámec Village, Teplá Dolina Valley; Chrámec Village, Vinohrady Area; Gemerský Jablonec Village, Petrovce Reservoir; Janice Village, Hadia Stráň Meadow; Petrovce Village, Fenek Forest; Šiatorská Bukovinka Village, Dolina Bukovinského Potoka Valley; Šiatorská Bukovinka Village, Rybník Pond; Šurice Village (Fenďa and Mašán 2009). Podunajská Rovina Flatland: Bratislava Capital, Podunajské Biskupice Settlement, Ostrov Kopáč Steppe (Kalúz 2007). Svätý Jur Town, Šúr Forest, Šúrsky Rybník Pond (Fenďa et al. 1998, Fenďa 2005). Trstená Na Ostrove Village, Kráľovská Lúka Forest (Kalúz 1994c). Jurová Village, Jurovský Les Forest (Čarnogurský et al. 1994). Slovenský Kras Karst: Silica Village, Silická Ľadnica Abyss (Kalúz 1993). Silica Village, Pod Fabiankou Forest (Kalúz 1992).

#### Dubious published material from Slovakia.

Malé Karpaty Mts.: Bratislava Capital, Devín Settlement, Devínska Lesostep Forest (Kalúz 2005). [Notes: *Epicriopsis horridus* belongs to the common species in Slovakia but the published specimens from Malé Karpaty Mountains are excluded from the list of valid findings of *Epicrius horridus* because accompanying „original“ figure available in Kalúz (2005) is obviously not belonging to this species, and it is only a true copy of illustration of *Epicriopsis langei* given to the original description of the new species from Central Asia by Livshitz and Mitrofanov (1975). Moreover, in a previous paper on mites collected in the Abrod Meadows by Kalúz (2003), the same illustration was used although no record of any *Epicriopsis* species from that locality is reported by the author.

#### New material from Slovakia.

Biele Karpaty Mts.: 1 ♀ – 3. 10. 1999, Nová Bošáca Village, broad-leaved deciduous forest (*Fagus sylvatica*, *Acer* sp., *Tilia* sp., *Carpinus betulus*, *Ulmus* sp.), leaf litter and soil detritus with nest of *Clethrionomys glareolus* (Mammalia), 450 m, leg. P. Mašán. Borská Nížina Lowland: 1 ♀ – 18. 5. 1992, Vysoká Pri Morave Village, littoral reed stand (*Phragmition*), nest of *Anas platyrhynchos* (Aves), altitude 145 m, leg. J. Krištofík and A. Darolová; 1 ♀ – 8. 4. 1998, Suchohrad Village, broad-leaved deciduous forest (mostly with *Ulmus* sp., *Carpinus betulus* and *Quercus* sp.), leaf litter and soil detritus, altitude 150 m, leg. P. Mašán; 1 ♀ – 24. 7. 1998, Stupava Town, littoral reed stand (*Phragmition*), leaf litter and soil detritus, altitude 180 m, leg. P. Mašán; 1 ♀ – 27. 6. 2002, Malé Leváre Village, Morava River (alluvium), wet willow-poplar flood plain forest (*Salici-Populetum*), leaf litter and soil detritus, altitude 160 m, leg. P. Mašán; 19 ♀♀ – 10. 4. 2004, Tomky Village, Dolná Studená Voda Reservoir, littoral alder forest (*Alnus* sp.), wet leaf litter and soil detritus, altitude 175 m, leg. P. Mašán; 1 ♀ – 29. 10. 2005, Borský Svätý Jur Village, agrocoenose, heap of decaying herbaceous vegetation, altitude 170 m, leg. P. Mašán. Burda Hills: 5 ♀♀ – Kamenica Nad Hronom Village, Kováčovské Kopce-juh Forest, oak forest (*Quercetum cerris*), leaf litter and soil detritus, altitude 330 m, leg. P. Mašán. Horehronské Podolie Basin: 1 ♂ – 20. 6. 2006, Zlatno Village, Zlatnianske Skalky Forest, pine forest (*Pinus sylvestris*), soil and detritus from ant-hill of *Formica* sp. (Hymenoptera, Formicidae), altitude 755 m, leg. P. Mašán. Ipeľská Kotlina Basin: 1 DN – 23. 6. 1997, Ipeľské Predmostie Village, Ryžovisko Forest, willow-poplar flood-plain forest (*Salici-Populetum*), growth of *Carex* sp., leaf litter and soil detritus, altitude 130 m, leg. P. Mašán. Kozie Chrbty Mts.: 1 ♀, 1 ♂ – 9. 7. 2003, Svit Town, Lopušná Dolina Valley, Tabličky Saddleback, glade in spruce forest (*Piceetum abietinum*) with solitary beeches (*Fagus sylvatica*), leaf litter and soil detritus, altitude 1,080 m, leg. P. Mašán. Malé Karpaty Mts.: 2 ♀♀ – 25. 7. 1990, Bratislava Capital, Železná Studienka Forest, broad-leaved deciduous forest, nest of *Erithacus rubecula* (Aves), altitude 250 m, leg. J. Krištofík and A. Darolová; 3 ♀♀ – 17. 4. 2006, Bratislava Capital, Devín Settlement, Devínska Kobyla Hill, forest steppe, moss, altitude 370 m, leg. P. Mašán. Muránska Planina Plateau: 1 ♀ – 8. 10. 2002, Závadka Nad Hronom Village, Veľká Stožka Mt., Birčiareň Forest, meadow, hay-loft, heterogeneous organic detritus under haystack, altitude 950 m, leg. P. Mašán. Podunajská Rovina Flatland: 1 ♀ – 6. 6. 1989, Veľké Blahovo Village, fish-ponds, shore reed stand (*Phragmition*), nest of *Fulica atra* (Aves), altitude 120 m, leg. J. Krištofík and A. Darolová; 1 ♀ – 26. 9. 2000, Veľký Meder Town, littoral reed stand (*Phragmition*), leaf litter and soil detritus, altitude 110 m, leg. P. Mašán; 1 ♀ – 9. 6. 2002, Bratislava Capital, Petržalka Settlement, Starý Háj Wood, hard-wood flood-plain forest (*Fraxino-Ulmetum carpinetosum*) with oak (*Quercus* sp.), leaf litter and soil detritus, altitude 135 m, leg. P. Mašán; 1 ♀ – 14. 9. 2002, Svätý Jur Town, Šúr Forest, wet alder forest (*Alnion glutinosae*), leaf litter and soil detritus, altitude 130 m, leg. P. Mašán; 1 ♀ – 19. 5. 2004, Bratislava Capital, Rusovce Settlement, park, growth of plane (*Platanus orientalis*) and lime (*Tilia* sp.), leaf litter and soil detritus, altitude 135 m, leg. P. Mašán. Pohronský Inovec Mts.: 1 ♀ – 2. 7. 2002, Stará Huta Village, Drozdovo Forest, beech forest (*Fagion sylvaticae*), leaf litter and soil detritus, altitude 650 m, leg. P. Mašán. Považský Inovec Mts.: 1 ♀ – 1. 4. 2000, Lúka Village, forest steppe with juniper (*Juniperus communis*), individual collecting under bark, stones and pieces of wood, altitude 250 m, leg. P. Mašán; 1 ♀ – 5. 7. 2004, Lúka Village, Tematínska Lesostep Forest, xerothermic oak forest (*Quercetum*) with pine (*Pinus nigra*), leaf litter and soil detritus, altitude 340 m, leg. P. Mašán; 1 ♀ – 9. 7. 2006, Lúka Village, Srnia Dolina Valley, xero-thermophilous edge of oak forest (*Quercetum*) with juniper (*Juniperus communis*), moss and soil detritus, altitude 250 m, leg. P. Mašán; 4 ♀♀ – 1. 11. 2013, Hrádok Village, Hrádocká Dolina Valley, meadow in broad-leaved deciduous forest, soil detritus with rotting plant remnants, altitude 280 m, leg. P. Mašán. Veľká Fatra Mts.: 2 ♀♀ – 29. 8. 2003, Necpaly Village, Necpalská Dolina Valley, Kýšky Mt., beech forest (*Fagion sylvaticae*), leaf litter and soil detritus, altitude 1,360 m, leg. P. Mašán; 16 ♀♀, 3 ♂♂ – 21. 7. 2004, Liptovské Revúce Village, Veľká Rakytová Dolina Valley, beech forest (*Fagion sylvaticae*), rocky canyon, moss, altitude 780 m, leg. P. Mašán. Veporské Vrchy Hills: 1 ♀ – 8. 10. 2002, Závadka Nad Hronom Village, Hronec Valley, Pod Pätinou, alluvium of brook with *Petasites hybridus*, soil detritus, altitude 780 m, leg. P. Mašán. Volovské Vrchy Hills: 4 ♀♀ – 24. 7. 2003, Betliar Village, park, park with *Quercus palustris*, *Fagus sylvatica*, *Ulmus laevis*, *Tilia* spp., *Abies alba* and *Picea abies*, leaf litter, soil detritus amd decaying plant remnants, altitude 350 m, leg. P. Mašán. Vtáčnik Mts.: 2 ♀♀ – 4. 11. 2003, Ostrý Grúň Village, Hlboká Dolina Valley, Pokuty, alluvium of brook with *Petasites* sp., mixed forest (*Ulmus* sp., *Fagus sylvatica* and *Abies alba*), moist soil detritus and moss, altitude 650 m, leg. P. Mašán; 2 ♀♀ – 4. 11. 2003, Ostrý Grúň Village, Hlboká Dolina Valley, Pavlova Lúka Meadow, meadow with juniper (*Juniperus communis*) and solitary elms (*Ulmus laevis*), soil detritus and leaf litter, altitude 850 m, leg. P. Mašán. Východoslovenská Rovina Plain: 3 ♀♀, 1 ♂ – 1. 6. 2004, Boťany Village, Latorický Luh I. Forest, hard-wood flood-plain forest (*Fraxino-Ulmetum carpinetosum*) with oak (*Quercus* sp.), wet leaf litter, altitude 100 m, leg. P. Mašán.

#### Remarks.

A new name, *Epicriopsis berlesei*, was proposed by Oudemans (1939a), namely for Italian specimens of *Epicriopsis horridus* first published by Berlese (1887a) under the name *Epicrius mollis*. There are three slides with the apparently original specimens of Oudemans labelled *E. berlesei* in the collection in Leiden (4061–4063), all with no type designation. The revised specimens belong at least to the two different species: (1) a deutonymph from San Remo, labelled 4061, is weakly sclerotised and unidentifiable, (2) a female from Sucy-en-Brie, labelled 4062, is hardly observable and very similar if not identical with *Epicriopsis palustris*, (3) a “deutonymph” from Borkum Island, labelled 4063, is a female of *E. horridus*. Karg (1971a, 1971b) considered *E. berlesei* to be a junior synonym of *E. horridus* giving no supporting information. Here I have adopted his synonymy although I have not examined the Italian specimens of *E. horridus* deposited in Berlese Collection in Firenze.

In addition, there are two further slides of the genus *Epicriopsis* in the Oudemans Collection (4064, 4065), labelled *Epicriopsis horridus*: (1) a “deutonymph” from Delden, labelled P4064, is male of *Epicriopsis rivus*, (2) a “female” from Delden, labelled P4065, was not found on that empty slide.

### Epicriopsis hungaricus

Taxon classificationAnimaliaMesostigmataAmeroseiidae

Kandil, 1978

Plate 30

Epicriopsis hungarica Kandil, 1978: 168.
Epicriopsis hungarica . — Narita and Moraes 2016: 483.

#### Type depository.

Hungarian Natural History Museum, Budapest, Hungary; Faculty of Agricultural Sciences, Moshtohor, Egypt.

#### Type locality and habitat.

Hungary, Bátorliget, habitat stated.

#### Comparative material.

Hungary: 3 ♀♀ (HNHM: Meso-1393, Meso-1393, Meso-1394, types) – 21. 4. 1976, Bátorliget, leg. S. Mahunka, H-1513; 1 ♀ (HNHM: Meso-1350) – May 1972, Balassagyarmat, leg. S. Mahunka, H-1395 (labelled *Epicriopsis baloghi*); 1 ♀ (HNHM: Meso-1392) – Aug. 1969, Dobogókő, leg. S. Mahunka, H-1154.

#### New material from Slovakia.

Borská Nížina Lowland: 1 ♀ – 25. 4. 2000, Stupava Town, littoral reed stand (*Phragmition*), leaf litter and soil detritus, altitude 180 m, leg. P. Mašán; 4 ♀♀ – 27. 6. 2002, Malé Leváre Village, Stará Morava Arm, hard-wood flood-plain forest (*Fraxino-Ulmetum carpinetosum*), leaf litter and wood detritus, altitude 150 m, leg. P. Mašán; 1 ♀ – 10. 4. 2004, Tomky Village, Dolná Studená Voda Reservoir, littoral alder forest (*Alnus* sp.), wet leaf litter and soil detritus, altitude 175 m, leg. P. Mašán. Podunajská Rovina Flatland: 1 ♀ – 17. 5. 1999, Veľké Kosihy Village, littoral reed stand (*Phragmition*), leaf litter and soil detritus, altitude 120 m, leg. P. Mašán; 1 ♀ – 19. 5. 2004 Bratislava Capital, Rusovce Settlement, park, growth of plane (*Platanus orientalis*) and lime (*Tilia* sp.), leaf litter and soil detritus, altitude 135 m, leg. P. Mašán; 1 ♀ – 8. 6. 2006, Bratislava Capital, Rusovce Settlement, park, growth of plane (*Platanus orientalis*), leaf litter and soil detritus, altitude 135 m, leg. P. Mašán. Považský Inovec Mts.: 2 ♀♀ – 22. 6. 2004, Lúka Village, Srnia Dolina Valley, broad-leaved deciduous forest, alluvium of brook, leaf litter and soil detritus, altitude 220 m, leg. P. Mašán.

#### Remarks.

There are seven slides labelled *Epicriopsis hungarica* in the Budapest Museum Collection, none with type designation. Except for one female from Dobogókő (see comparative material above), all of these specimens belong to the original type series. The same is true for the five available slides labelled *Epicriopsis baloghi* Kandil, 1978. One of the slides, labelled Meso-1350, bears a female of *E. hungaricus*, misidentified as *E. baloghi*.

### Epicriopsis jilinensis

Taxon classificationAnimaliaMesostigmataAmeroseiidae

Ma, 2002

Epicriopsis jilinensis Ma, 2002: 308.
Epicriopsis jilinensis . — Narita and Moraes 2016: 483.

#### Type depository.

National Base of Plague and Brucellosis Control, Baicheng, China.

#### Type locality and habitat.

China, Jilin Province, Dunhua County, in forest soil.

#### Remarks.

A species with dorsal chaetotaxy very similar to that of *Epicriopsis suedus*.

### Epicriopsis linzhiensis

Taxon classificationAnimaliaMesostigmataAmeroseiidae

Ma & Lin, 2016

Epicriopsis linzhiensis Ma & Lin, 2016: 14.

#### Type depository.

Institute of Plant Protection, Fujian Academy of Agricultural Science, Fuzhou, China.

#### Type locality and habitat.

China, Xizang Autonomous Region, Linzhi County, bark of tree.

### Epicriopsis mirabilis

Taxon classificationAnimaliaMesostigmataAmeroseiidae

Willmann, 1956

Plates 31 , 32

Epicriopsis mirabilis Willmann, 1956: 218.
Epicriopsis mirabilis . — Karg 1971a: 226; Karg 1971b: VI/7; Bregetova 1977: 169; Kandil 1978: 171; Karg 1993: 225.
Epicriopsis rivus Karg, 1971a: 226. **Syn. n.**
Epicriopsis rivus . — Karg 1971b: VI/7; Bregetova 1977: 169; Kandil 1978: 171; Karg 1993: 224; Narita and Moraes 2016: 483.

#### Type depository.

Of *Epicriopsis mirabilis* – Zoologischen Staatssammlung München, Germany; of *Epicriopsis rivus* – Museum für Naturkunde, Berlin, Germany.

#### Type locality and habitat.

Of *Epicriopsis mirabilis* – Czech Republic, Králický Sněžník Mt., southern slope with growth of European white hellebore (*Veratrum album*), in moss (*Sphagnum* sp., *Hypnum* sp.); of *Epicriopsis rivus* – Germany, Quarmbeck, Suderode/Harz, leaf litter under beech tree.

#### Comparative material.

Czech Republic: 1 ♂ (ZSM: Coll. Hirschm./Willm. 42/8, 43/8, holotype) – W10/11, W10/13, no collection data, det. C. Willmann. Germany: 1 ♀ (ZMB: 41221, holotype) – 10. 10. 1963, Suderode, Harz, Friedr. brunn, Laub, am Bach, 2927; 2 ♀♀ (ZMB: 41223, 41224, paratypes) – 4. 11. 1965, Nähe Hasselfelde/Harz, Kahlschlag, Moos, Humusschicht, Buchenlaub, 2929–2930 (all labelled *Epicriopsis rivus*); 1 ♀ (IZSAV) – 25. 4. 2007, Bavarian Prealps Mts., Flintsbach am Inn, Peterskirchlein, altitude 800 m, spruce forest, wet soil detritus, leg. P. Mašán. Netherlands: 1 ♂ (RMNH: ACA.P.4064) – 9. 4. 1896, Delden, in rottende bladen, leg. Oudemans (labelled Nph II, *Epicriopsis horridus*).

#### Published material from Slovakia.

Malá Fatra Mts.: Terchová Village, Rozsutec Mt., Skalné Mesto Forest (Kalúz 1998, cited as *Epicriopsis rivus*). Oravská Magura Mts.: Oravská Lesná Village, Paráč Forest (Kalúz 1996, cited as *Epicriopsis rivus*).

#### New material from Slovakia.

Kozie Chrbty Mts.: 3 ♀♀ – 9. 7. 2003, Svit Town, Lopušná Dolina Valley, spruce forest (*Piceetum abietinum*) with beech (*Fagus sylvatica*), wet growth of *Petasites* sp. in brook alluvium, moss, altitude 900 m, leg. P. Mašán. Moravsko-Sliezske Beskydy Mts.: 1 ♀ – 30. 6. 1997, Klokočov Village, Malý Polom Forest, peat-bog in spruce forest (*Piceetum abietinum*), moss and soil detritus, altitude 1,000 m, leg. P. Mašán. Vtáčnik Mts.: 1 ♂ – 4. 11. 2003, Ostrý Grúň Village, Hlboká Dolina Valley, Pokuty, alluvium of brook with *Petasites* sp., mixed forest (*Ulmus* sp., *Fagus sylvatica* and *Abies alba*), moist soil detritus and moss, altitude 650 m, leg. P. Mašán.

#### Remarks.

There are two apparently original slides of *Epicriopsis mirabilis* with a dissected male in comparatively good condition in the Hirschmann/Willmann Collection in München (gnathosoma, chelicera and leg I are separately mounted on a slide), labelled No. 42/8 and 43/8. The male may be considered as holotype by monotypy. My examination of this Willmann‘s specimen confirmed the conspecificity of *E. mirabilis* with the species *Epicriopsis rivus* Karg 1971. According to Karg (1971a) and the followers, the only reliable diagnostic feature for distinguishing *E. mirabilis* from *E. rivus* seemed to be the form of the epistome. In *E. mirabilis*, the epistome was misinterpretated by Willmann (1956) and illustrated as a trilobate structure while the epistome of *E. rivus* was correctly understood to be a sharply pointed structure. There is a relatively clearly visible epistome in dissected gnathosoma on slide No. 43/8, with the anterior margin produced into a medial acute process, as in *E. rivus*. The type specimens of both species are also identical in relation to all other morphological features, including the peculiar sculpture composed of polygonal instead of star-shaped tubercles.

### Epicriopsis palustris

Taxon classificationAnimaliaMesostigmataAmeroseiidae

Karg, 1971

Plate 33

Epicriopsis palustris Karg, 1971b: VI/3.
Epicriopsis langei Livshits & Mitrofanov, 1975: 464. **Syn. n.**
Epicriopsis baloghi Kandil, 1978: 165. **Syn. n.**
Epicriopsis palustris . — Karg 1993: 222; Călugăr 2008: 169; Hajizadeh et al. 2013b: 68; Kazemi and Rajaei 2013: 67; Nemati et al. 2013: 20; Narita and Moraes 2016: 483.
Epicriopsis horridus . — Evans and Till 1979: 230; Iavorschi 1995: 61. **Misidentifications.**
Epicriopsis baloghi . — Khalili-Moghadam and Saboori 2016: 546; Narita and Moraes 2016: 483.
Epicriopsis langei . — Narita and Moraes 2016: 483.

#### Type depository.

Of *Epicriopsis palustris* – Museum für Naturkunde, Berlin, Germany; of *Epicriopsis langei* – Nikita Botanical Gardens, National Scientific Center, Yalta, Crimea, Russia (the type specimens not found and probably lost, based on personal communication from Alex Khaustov); of *Epicriopsis baloghi* – Hungarian Natural History Museum, Budapest, Hungary; Faculty of Agricultural Sciences, Moshtohor, Egypt.

#### Type locality and habitat.

Of *Epicriopsis palustris* – Germany, Kalktuffniedermoor Nature Reserve, Oechsen/Rhön; leaf litter under hawthorn, *Crataegus* sp.; of *Epicriopsis langei* – Russia, Crimea, Nikita Botanical Gardens, in leaf litter, in park; of *Epicriopsis baloghi* – Hungary, Balassagyarmat, habitat stated.

#### Comparative material.

Germany: 1 ♀, 1 ♂ (ZMB: 40957, male holotype; ZMB: 40958, female paratype) – 16. 8. 1967, Kalktuffniedermoor/Rhön, Laubstreu unter Weißdorn, 2957, 2956; 1 ♂ (ZMB: 40959, paratype) – 5. 9. 1963, Mahndorf b. Halberstadt, Lehm, 2951. Greece: 3 ♀♀ (IZSAV) – 16. 8. 2005, Chalkidiki Peninsula, Sarti Village, bank of a small river under plane trees (*Platanus* sp.), soil detritus with leaf fall, leg. P. Mašán. Hungary: 2 ♀♀ (HNHM: Meso-1348, Meso-1349, types) – May 1972, Balassagyarmat, leg. S. Mahunka, H-1395 (labelled *Epicriopsis baloghi*); 1 ♀ (HNHM: Meso-1347, type) – May 1972, Balassagyarmat, leg. S. Mahunka, H-1394 (labelled *Epicriopsis baloghi*). Iran: 1 ♀ (CJH) – Talesh, Guilan Province, soil sample, leg. and det. J. Hajizadeh (labelled *Epicriopsis horridus*). United Kingdom: 1 ♀ (BMNH: 1955.6.1.75) – 1951, Gorey, Jersey, grassland, leg. J. G. Sheals, det. G. O. Evans (labelled *Epicriopsis horridus*).

#### Published material from Slovakia.

Košická Kotlina Basin: Valaliky Village (Kováč et al. 1999).

#### New material from Slovakia.

Borská Nížina Lowland: 2 ♀♀ – 10. 4. 2004, Tomky Village, Dolná Studená Voda Reservoir, littoral alder forest (*Alnus* sp.), wet leaf litter and soil detritus, altitude 175 m, leg. P. Mašán. Veľká Fatra Mts.: 4 ♀♀, 2 ♂♂ – 21. 7. 2004, Liptovské Revúce Village, Veľká Rakytová Dolina Valley, beech forest (*Fagion sylvaticae*), rocky canyon, moss, altitude 780 m, leg. P. Mašán.

#### Remarks.

Despite of the fact that *Epicriopsis palustris* was described in 1971 by Karg, it was not included in Karg‘s widely used monograph focused on identification of the European mesostigmatans, published in the same year (Karg 1971a). The original description of this species contains an incorrect statement about the setation of ventrianal shield in female. I have carefully checked the ventrianal shield in the paratype female, and I could detect only three circum-anal setae standard for the genus, but not the two additional opisthogastric setae inserted on the lateral parts of the shield, as illustrated in fig. 7a (page 6) of Karg (1971b). Karg’s confusion is still respected by some authors, and it can be found in the most recent studies on the genus *Epicriopsis* up to now (Narita and Moraes 2016), although *E. palustris* was adequately redescribed by Călugăr (2008), based on females and males from Romania.

*Epicriopsis palustris* was originally described based on the type specimens from Germany; subsequent findings of this species are reported from various countries in central and northern Europe (Hungary, Latvia, Poland, Romania, Slovakia). I have collected this species in the Mediterranean area of Greece (Sarti Village, Chalkidiki Peninsula), and checked relatively rich material from various parts of Iran. Under the name *horridus*, the species is reported from Israel by Iavorschi (1995). Therefore, the species has clearly more expanded distribution area than previously expected. The specimens of *E. palustris* from Ukraine and Hungary were treated as a new species by Livshits and Mitrofanov (1975) and Kandil (1978), namely *Epicriopsis langei* and *Epicriopsis baloghi*. These authors apparently neglected the existence of *E. palustris*; only *Epicriopsis horridus* and *Epicriopsis rivus* were compared with their newly described species in the differential diagnose, and no reference to *E. palustris* can be found there.

Tracking of the dorsal setae in European species of *Epicriopsis*, especially the shortest ones, may be quite tricky task due to heavy sclerotization and coarse sculpture of the dorsal shield. In published papers, the full complement of the dorsal shield setae has been correctly depicted only exceptionally and in limited number of illustrations. For instance, also in *Epicriopsis palustris*, we can see incomplete numbers of setae on the dorsal shield, given by various authors, namely 18 pairs by Karg (1971b) and Livshits and Mitrofanov (1975), 17 pairs by Kandil (1978), 16 pairs by Iavorschi (1995), and 21 pairs by Călugăr (2008). I have found 24 pairs of the dorsal shield setae, and the same number is depicted on a figure of misidentified female published by Evans and Till (1979).

Livshits and Mitrofanov (1975) described *Epicriopsis langei* on the base of a single female reportedly deposited at the Nikita Botanical Gardens, National Scientific Center, Yalta, Crimea. The species seems to be apparently conspecific with *Epicriopsis palustris*, when compared with the types from Germany and my own specimens from Slovakia, except the form of epistome (illustrated as pointed in *E. langei*, rounded in *E. palustris*). I dare to say the epistome of *E. langei* is not correctly illustrated in the original illustrations, as in *Epicriopsis mirabilis*. I tried to obtain the type specimen of *E. langei* for examination, asking Alex Khaustov (at that time an acarologist of the National Scientific Center in Yalta) for help. Despite his great effort, the types were not found and are presumably lost.

### Epicriopsis stellata

Taxon classificationAnimaliaMesostigmataAmeroseiidae

Ishikawa, 1972

Epicriopsis stellata Ishikawa, 1972: 95.
Epicriopsis stellata . — Narita and Moraes 2016: 483.

#### Type depository.

Biological Laboratory, Matsuyama Shinonome Junior College, Matsuyama, Japan.

#### Type locality and habitat.

Japan, Ehime Prefectship, Matsuyama, Kuwabara, in haystack, in persimmon orchard (*Diospyros kaki*).

#### Comparative material.

Japan: 1 ♀ (CKI, paratype) – 28. 1. 1967, Matsuyama, K. Ishikawa.

#### Remarks.

I examined a paratype female of this species. I could detect 24 pairs of dorsal shield setae instead of 23 pairs as stated by Ishikawa (1972). *Epicriopsis stellata* is closely related to *Epicriopsis hungaricus* and *Epicriopsis palustris*, and it can be distinguished by the number of opisthogastric setae inserted on soft integument (six pairs in *E. stellata*, five pairs in *E. palustris* and *E. hungaricus*), and the relative length of S4 and S5 (in *E. stellata*, both similar; in *E. palustris* and *E. hungaricus*, S5 thicker, delicately pilose and at least twice longer than S4).

### Epicriopsis suedus

Taxon classificationAnimaliaMesostigmataAmeroseiidae

Karg, 1971

Plates 34 , 76C

Epicriopsis suedus Karg, 1971a: 226.
Epicriopsis suedus . — Karg 1971b: VI/7; Bregetova 1977: 169; Kandil 1978: 171; Karg 1993: 224; Narita and Moraes 2016: 482.

#### Type depository.

Museum für Naturkunde, Berlin, Germany.

#### Type locality and habitat.

Sweden, Solna Municipality, agricultural land, microhabitat stated.

#### Comparative material.

Sweden: 2 ♀♀ (ZMB: 41443, holotype; ZMB: 41444 paratype) – Schweden, 2966–2967.

#### Remarks.

This species bears almost full complement of dorsal shield setae. I found 28 pairs in the type specimens from Sweden (s6 presumably absent). Karg (1971a) depicted only 22 pairs in his original illustration. I was unable to check the number of opisthogastric setae due to the position of the specimens on the slide.

### Epicriopsis walteri

Taxon classificationAnimaliaMesostigmataAmeroseiidae

Halliday, 1997

Epicriopsis walteri Halliday, 1997: 187.
Epicriopsis walteri . — Narita and Moraes 2016: 482.

#### Type depository.

Australian National Insect Collection, CSIRO, Canberra, Australia.

#### Type locality and habitat.

Australia, Queensland, Conondale National Park, Bundaroo Creek, rainforest leaf litter.

### Genus Hattena

Taxon classificationAnimaliaMesostigmataAmeroseiidae

Domrow, 1963

Hattena Domrow, 1963: 202. Type species: Hattena erosa Domrow, 1963, by monotypy.
Edbarellus Manson, 1974: 115. Type species: Edbarellus tonganus Manson, 1974, by original designation. Synonymy by Halliday (1997).

#### Diagnosis (adults).

In female, dorsal shield lightly sclerotised, weakly reticulated or unornamented, often irregular in outlines (with 1–3 pairs of lateral incisions), narrowed and not covering whole dorsal surface; dorsal setae short, needle-shaped, similar in length, inserted on dorsal shield (18–25 pairs) and soft integument beside the dorsal shield (3–20 pairs); sometimes dorsolateral and ventrolateral soft integument hypertrichous, with up to 35 setal pairs (non-hypertrichous forms usually with standard number of 28–29 pairs of dorsal setae on dorsal shield and soft integument). Male often with some dorsal shield setae (8–10 pairs) conspicuous, thickened, extremely long and much longer than some of the shortest ones; with slightly expanded dorsal shield capturing some additional pairs of setae inserted on soft integument in female. Female with st1 on weakly sclerotised and inconspicuous sternal shield; setae st2 on sternal shield, small sternal shield fragments or soft integument; setae st3 associated with small endopodal platelets II-III or on soft integument; and st4 on soft integument; sternal shield usually markedly reduced; epigynal shield widely separated from anal shield, with parallel lateral margins, and usually with genital poroids on its surface. Female with anal shield, male with anal shield or ventrianal shield bearing 1–3 pairs of opisthogastric setae. Peritrematal shields or peritremes with anterior ends free and not connected to dorsal shield. Opisthogastric soft integument with five to six pairs of setae (JV3 usually absent in male). Corniculi slender and pointed, with undivided apex. Fixed digit of female chelicera usually edentate, with bifid terminal hook, sometimes with one or two medial denticles; movable digit edentate, with strongly developed terminal hook; pilus dentilis usually not modified into a membranous structure; male with well elongated spermatodactyl directed downward. Palptarsal apotele two-tined. Genu III and tibia III with two anterolateral and one posterolateral setae; tibia IV with two anterolateral and two posterolateral setae. Tarsi I–IV each with empodium and slightly to fully reduced claws. Insemination apparatus with spermathecal ducts unfused.

#### Remarks.

Generally, the genus *Hattena* as a whole shows relatively high diversity in external morphology, and it represents a conglomerate of morphologically heterogeneous species. The following character states were used to separate the *Hattena* species into five species groups (see the identification key below): (1) expression of z6 (absent in *cometis* group); (2) expression of J3 (present in *panopla* group); (3) position of Z5 (on soft integument in *panopla* group); (4) setation of soft integument (hypertrichy in *cometis* and *erosa* groups); (5) form of posterior part of dorsal shield (incised in *dalyi* and *panopla* groups); (6) sexual dimorphism of dorsal shield setae (markedly developed in *cometis* group); (7) position of genital poroids (outside the epigynal shield in *erosa* and *senaria* groups); (8) sexual dimorphism of anal shield (absent in *dalyi* group); (9) character of tritosternal laciniae (fused for most of their length in *panopla* group); (10) setation of ventrianal shield in male (with a pair of opisthogastric setae in *panopla* group and three pairs in *cometis* group); (11) setation of palptrochanter (with only one ventral seta in *cometis* and *erosa* groups); (12) legs IV in male (spurred in *cometis* group).

In this genus, the dorsal shield of males is more expanded than in females, thus bearing up to ten pairs of the setae that in females are inserted on the soft integument. *Hattena cometis*, *Hattena erosa* and *Hattena floricola* have polytrichous soft integument on dorso- and ventrolateral idiosomal surface. Polytrichy has been mentioned in the literature only for females; it was not reported in the descriptions of males of *H. cometis* and *H. floricola*. Therefore, the polytrichy of male soft integument may be neglected by Domrow (1979) and Halliday (1997), if not considerably reduced or absent.

It is remarkable that there is an additional setal pair of the J-series (J3) expressed on the dorsal shield in *Hattena panopla* and *Hattena tongana*. This seta is also present in *Neocypholaelaps novaehollandiae*.

*Hattena clemmys* was not included in the species group classification scheme introduced in this paper because there are several evidences that it is incorrectly placed in this genus: (1) dorsal shield divided into six fragments; (2) sternal shield not strongly reduced but without sternal setae and poroids; (3) setae st5 on soft integument, off the epigynal shield; (4) peritremes strongly abbreviated; (5) movable digit of chelicera with elongate and fringed process; (6) palptarsal apotele three-tined; (7) tarsus of legs I–IV with normally developed claws on empodium. It was placed in *Hattena* because of the extent of the dorsal shield and the number of dorsal shield setae, but it probably represents a new genus. Therefore the above mentioned specific characters of *H. clemmys* are not included in the diagnosis of *Hattena*.

The genus *Hattena* now comprises ten species distributed in the tropical areas of the Old and New Worlds (Australia, Ecuador, Kenya, Malaysia, Pacific Ocean Region, Papua New Guinea, Taiwan and Vietnam), in association with flowers and flower-visiting animals (birds, bees and bats). *Hattena dalyi* and *Hattena tonganus* are known to be phoretically associated with the Euroepean honey bee, *Apis mellifera* (from tropics).

Partial keys to the known species of *Hattena* may be found only in Halliday (1997), for four species reported from Australia (*cometis*, *floricola*, *incisa* and *panopla*), and in Faraji and Cornejo (2006), based on six species known up to that time (*cometis*, *erosa*, *floricola*, *incisa*, *panopla* and *rhizophorae*). A new and more complete key to the genus is needed to include other species, namely *Hattena dalyi* (transferred from *Afrocypholaelaps* by Klimov et al. in 2016) and *Hattena senaria* comb. n.

#### Key to species group of *Hattena* (adults)

**Table:** 

1	Female idiosoma hypotrichous or holotrichous, with at most 30 pairs of dorsal and six pairs of opisthogastric setae (in addition to the sternal and circum-anal setae); sexual dimorphism of dorsal shield setae not well developed: female and male (when described) with short, pointed and subequal setae; male with anal shield or small ventrianal shield bearing at most one pair of opisthogastric setae (JV2); palptrochanter with two ventral setae; male femur IV not spurred	**2**
–	Female idiosoma polytrichous, with 34–40 pairs of dorsal and 8–15 pairs of opisthogastric setae; sexual dimorphism of dorsal shield setae well developed: female with short, pointed and subequal setae, male with some dorsal setae (8–10 pairs) conspicuously thickened and lengthened; male with expanded ventrianal shield bearing three pairs of opisthogastric setae (JV1, JV2, ZV2); palptrochanter with one ventral seta; male femur IV with ventral spur	**4**
2	Dorsal shield without posterolateral incisions; genital poroids on soft integument close to posterolateral margins of epigynal shield, the shield with moderate posterior expansion	***Hattena senaria* group** (*senaria*)
–	Dorsal shield with 1–3 pairs of posterolateral incisions; genital poroids on epigynal shield, the shield with parallel lateral margins, unexpanded posteriorly	**3**
3	Dorsocentral J-series with three pairs of setae (J2–J4); in female, Z5 on soft integument behind posterior margin of dorsal shield; male ventrianal shield bearing a pair of opisthogastric setae (in addition to the circum-anal setae) and a wide incision on anterior margin; laciniae of tritosternum longer, free for most of their length	***Hattena panopla* group** (*panopla*, *tongana*)
–	Dorsocentral J-series with two pairs of setae (J2, J4; J3 absent); in female, Z5 on dorsal shield; male anal shield bearing only circum-anal setae, anterior magin of the shield without incision; laciniae of tritosternum shorter, fused for most of their length	***Hattena dalyi* group** (*dalyi*, *incisa*, *rhizophorae*)
4	Setae z6 present; genital poroids on soft integument close to posterolateral margins of epigynal shield	***Hattena erosa* group** (*erosa*)
–	Setae z6 absent; genital poroids on epigynal shield	***Hattena cometis* group** (*cometis*, *floricola*)

### Hattena clemmys

Taxon classificationAnimaliaMesostigmataAmeroseiidae

Domrow, 1981

Hattena clemmys Domrow, 1981: 415.

#### Type depository.

United States National Museum, Washington, D.C., USA; Canada Department of Agriculture, Ottawa, Canada; British Museum (Natural History), London, United Kingdom; Institute for Medical Research, Kuala Lumpur, Malaysia; Queensland Institute of Medical Research, Brisbane, Australia.

#### Type locality and habitat.

Malaysia, Pahang, Gunong Batu Brinchang, on long-tongued nectar bat, *Mocroglossus minimus* (Mammalia, Chiroptera).

### Hattena cometis

Taxon classificationAnimaliaMesostigmataAmeroseiidae

Domrow, 1979

Hattena cometis Domrow, 1979: 109.
Hattena cometis . — Seeman 1996: 193; Halliday 1997: 190; Faraji and Cornejo 2006: 291; Palma et al. 2013a: 404; Palma et al. 2013b: 918; Alberti et al. 2013: 1010.

#### Type depository.

Queensland Museum, Brisbane, Australia; Queensland Institute of Medical Research, Brisbane, Australia.

#### Type locality and habitat.

Australia, Queensland, Bamaga, from beak and nostrils of yellow honeyeater, *Meliphaga flava* (Aves, Passeriformes).

### Hattena dalyi

Taxon classificationAnimaliaMesostigmataAmeroseiidae

(Elsen, 1974)

Afrocypholaelaps dalyi Elsen, 1974: 160.
Hattena dalyi . — Klimov et al. 2016.

#### Type depository.

Musée Royal de l'Afrique Centrale, Tervuren Belgium.

#### Type locality and habitat.

Kenya, Malindi, on apid bee, *Ceratina subquadrata* (Hymenoptera).

### Hattena erosa

Taxon classificationAnimaliaMesostigmataAmeroseiidae

Domrow, 1963

Hattena erosa Domrow, 1963: 203.
Hattena erosa . — Faraji and Cornejo 2006: 291.

#### Type depository.

United States National Museum, Washington, D.C., USA; British Museum (Natural History), London, United Kingdom; Queensland Institute of Medical Research, Brisbane, Australia.

#### Type locality and habitat.

Malaysia, Borneo, Kamborangah, Mt. Kinabalu, on unidentified bird species.

### Hattena floricola

Taxon classificationAnimaliaMesostigmataAmeroseiidae

Halliday, 1997

Hattena floricola Halliday, 1997: 190.
Hattena floricola . — Faraji and Cornejo 2006: 291.

#### Type depository.

Australian National Insect Collection, CSIRO, Canberra, Australia.

#### Type locality and habitat.

Australia, Victoria, Boronia, on flowers of mountain correa, *Correa lawrenceana* (Rutaceae).

### Hattena incisa

Taxon classificationAnimaliaMesostigmataAmeroseiidae

Halliday, 1997

Hattena incisa Halliday, 1997: 193.
Hattena incisa . — Faraji and Cornejo 2006: 291.

#### Type depository.

Australian National Insect Collection, CSIRO, Canberra, Australia.

#### Type locality and habitat.

Australia, Northern Territory, Elizabeth River near Palmerston, on flowers of spotted mangrove, *Rhizophora stylosa* (Rhizophoraceae).

#### Remarks.

*Hattena dalyi* (Elsen, 1974) is exceedingly similar to *Hattena incisa*. The position of st2 appears to be the only important difference between these species; these are on the sternal shield margin in *H. dalyi* and on separate and small shield fragments close to lateral margins of the sternal shield in *H. incisa*. Halliday (1997) may have overlooked the existence of *H. dalyi* because it was described as a species of *Afrocypholaelaps*, until Klimov et al. (2016) transferred it to *Hattena*. In addition, Halliday (1997) did not cite Elsen (1974). Thus, a thorough morphological comparison between these species is necessary.

### Hattena panopla

Taxon classificationAnimaliaMesostigmataAmeroseiidae

Domrow, 1966

Hattena panopla Domrow, 1966: 208.
Hattena panopla . — Fain and Lukoschus 1979: 20; Domrow 1979: 105; Seeman 1996: 193; Halliday 1997: 195.
Hattena panolpa (sic). — Faraji and Cornejo 2006: 291.

#### Type depository.

Australian National Insect Collection, CSIRO, Canberra, Australia.

#### Type locality and habitat.

Australia, Queensland, Sarina, Chelona, from nostrils of brown honeyeater, *Lichmera indistincta* (as *Gliciphila indistincta*) (Aves, Passeriformes).

### Hattena rhizophorae

Taxon classificationAnimaliaMesostigmataAmeroseiidae

Faraji & Cornejo, 2006

Hattena rhizophorae Faraji & Cornejo, 2006: 287.

#### Type depository.

Museo de Zoología de la Pontificia Universidad Católica de Quito, Ecaudor; Australian National Insect Collection, CSIRO, Canberra, Australia; National Museum of Natural History, Naturalis Biodiversity Center, Leiden, Netherlands; British Museum (Natural History), London, United Kingdom; United States National Museum, Washington, D.C., USA.

#### Type locality and habitat.

Ecuador, Manabí Province, Río Chone Estuary, Punta Blanca Mangrove, Bahía de Caraquez, on flowers of red mangrove, *Rhizophora mangle* (Rhizophoraceae).

#### Remarks.

Faraji and Cornejo (2006) reported this species as having only four instead of six pairs of setae on opisthogastric soft integument, taking no account of the relative positions of all these setae on an expanded striate integument and their dimorphic expression common in Ameroseiidae (a pair of opisthogastric setae usually absent in male). For that reason, they interpreted JV4 and JV5 (designated in their illustration as R3 and R4) to belong to a complement of posterior marginal dorsal setae. Later, in their description of male and deutonymph, these setae are not referred to. Their JV5 in female and deutonymph are interpreted here as JV3, and they seem to be absent in male. I suspect JV4 to be omitted in the male illustrations of Faraji and Cornejo (2006), and JV5 not to be homologous in adults of both sexes.

### Hattena senaria

Taxon classificationAnimaliaMesostigmataAmeroseiidae

(Allred, 1970) comb. n.

Ameroseius senarius Allred, 1970: 101.

#### Type depository.

Bernice Pauahi Bishop Museum (Hawaii State Museum of Natural and Cultural History), Honolulu, Hawaii, USA; United States National Museum, Washington, D.C., USA.

#### Type locality and habitat.

Papua New Guinea, May River, on imperial pigeon, *Ducula* sp. (Aves, Columbiformes).

#### Remarks.

I have placed this species in *Hattena* on the basis of the following characters: (1) dorsal shield not covering whole dorsal surface and bearing only 22 pairs of setae; (2) soft integument beside the anterior dorsal shield with seven pairs of setae; (3) idiosomal setae short, smooth and pointed.

### Hattena tongana

Taxon classificationAnimaliaMesostigmataAmeroseiidae

(Manson, 1974)

Edbarellus tonganus Manson, 1974: 116.
Hattena tongana . — Halliday 1997: 188.
Hattena tonganus . — Klimov et al. 2016.

#### Type depository.

Ministry of Agriculture and Fisheries, Levin, New Zealand; United States National Museum, Washington, D.C., USA; Entomology Division, Department of Scientific and Industrial Research, Auckland, New Zealand.

#### Type locality and habitat.

Pacific Ocean Region, Kingdom of Tonga, Tongatapu Island, in a hive of European honey bee, *Apis mellifera* (Hymenoptera).

#### Remarks.

I suspect this species to be a junior synonym of *Hattena panopla*. Halliday (1997) reported many extreme similarities between *Hattena tongana* and *H. panopla*, but he did not consider them to be conspecific. Differences in shape of sternal shields described by Halliday (1997) could be questionable due to the weak sclerotization of the sternal shield and its margins, characterstic of *Hattena*. However, I adopted in this paper the interpretation of Halliday (1997), considering them distinct species.

### Genus Kleemannia

Taxon classificationAnimaliaMesostigmataAmeroseiidae

Oudemans, 1930

Kleemannia Oudemans, 1930: 135. Type species: Zercon pavidus C. L. Koch, 1839, by original designation.
Primoseius Womersley, 1956b: 116. Type species: Zercoseius macauleyi Hughes, 1948 (= Seiulus plumosus Oudemans, 1902), by original designation. Synonymy by Hughes (1961).
Kleemannia . — Evans 1963a: 229. not Zercoseius Berlese, 1916a: 33. Type species: Seius spathuliger Leonardi, 1899, by original designation. Incorrect synonymy by Oudemans (1939b).

#### Diagnosis (adults).

Dorsal shield variously sclerotised and ornamented, normally with 28–29 pairs of setae (z6 present or absent). Dorsal shield setae similar in adults of both sexes, well developed and relatively long, thickened, tubiform or flattened, mostly densely pilose or serrate. In female, st1 and st2 on sternal shield, st3 on small suboval or subcircular pseudo-metasternal platelets or sometimes on soft integument (in *Kleemannia mirabilis* sp. n. on sternal shield), and st4 on soft integument; endopodal platelets II-III present, subtriangular, and relatively small; anterior margin of epigynal shield convex, genital poroids on soft integument. Female with five or six pairs of opisthogastric setae (JV1–JV3, JV5, ZV2; JV4 sometimes absent), two pairs of which (JV2, JV3) on ventrianal shield (in *Kleemannia insignis*, JV2 occasionally on soft integument but contiguous to anterior margin of ventrianal shield), other on soft integument; male with five pairs of opisthogastric setae (JV4 always absent), 3–5 pairs of which on expanded ventrianal shield (JV1 and/or JV5 sometimes on soft integument). In both sexes, JV5 usually similar to those on dorsal shield, other ventrally inserted setae simple, smooth and needle-like. Peritrematal shields anteriorly connected to dorsal shield, and often with enlarged cavity-like poroid structure at level of coxae III. Metapodal platelets well developed, elongate. Corniculi well sclerotised and spaced, usually with bifid or trifid apex; setae h1 thickened, straight or slightly and regularly curved, progressively taperred and pointed. Fixed digit of chelicera with four subequal teeth on proximal masticatory area; male spermatodactyl relatively small, usually as long as movable cheliceral digit (if not shorter). Epistome with anterior margin produced into narrow and pointed central projection or, sometimes, short obtuse cusp; lateral margins mostly smooth. Palptarsal apotele usually two-tined. Coxae I–IV with delicate sculptural ornamentation, striate and reticulate. Genu III and tibiae III–IV with two anterolateral and two posterolateral setae. Tarsi I–IV each with empodium and claws; tarsi I usually well pigmented, brown in colour. Insemination apparatus with papilla-like sperm induction pores (solenostomes) associated with inner margin of coxae III.

#### Remarks.

Oudemans (1930) proposed the new genus *Kleemannia* with *Zercon pavidus* C. L. Koch, 1839 as type species, and added three further species in the genus (*plumosa*, *plumea* and *plumigera*). Since then, this taxon has been variously treated as a genus (Vitzthum 1942; Hughes 1961, 1976; Evans 1963a; Ishikawa 1972; Domrow 1974, 1979; Evans and Till 1979), a subgenus of *Ameroseius* (Athias-Henriot 1959, Hajizadeh et al. 2013a), or a junior synonym of *Ameroseius* (Westerboer and Bernhard 1963; Bregetova 1977; Karg 1993, 2005; Halliday 1997; Karg and Schorlemmer 2009; Narita et al. 2013b), although it may be easily and reliably separated from *Ameroseius* by the presence of two posterolateral setae on genu III and tiabiae III–IV (one in *Ameroseius*), four proximal denticles on fixed digit of chelicerae (at most three denticles in *Ameroseius*), two pairs of opisthogastric setae on ventrianal shield (at most one pair in *Ameroseius*, but never JV2), two-tined claw on palptarsus (three-tined in *Ameroseius*), thickened h1 (similar if compared to other hypostomal setae in *Ameroseius*), epistome usually with sharply or obtusely pointed apex (never produced into a single pointed process in *Ameroseius*). Therefore, the genus *Kleemannia* is considered to be a valid genus, and is removed from synonymy with *Ameroseius*.

The related genus *Primoseius* was proposed and very briefly characterised by Womersley (1956b). He included two species in the genus, the type species, mentioned as Lasioseius (Zercoseius) macauleyi Hughes, 1948 (sic, should be *Zercoseius macauleyi*), and Lasioseius (Zercoseius) gracei Hughes, 1948 (sic, should be *Zercoseius gracei*), which were subsequently synonymised with *Kleemannia plumosa* and *Kleemannia plumigera*, respectively (Evans 1954). Later, the separate taxonomic status of this genus was rejected by Hughes (1961), Evans and Till (1979), Karg (1971a, 1993, 2005), Bregetova (1977), and many others, because the original concept of *Primoseius* was defined with the help of the same characters as they can be found in the most relative genus *Ameroseius*, or *Kleemannia* s. l. Womersley (1956b) based the diagnosis of his new genus especially on the possession of two pairs of setae (st1, st2) on sternal shield, a pair of pseudo-metasternal platelets each bearing a seta (st3), and metasternal setae (st4) on soft integument. Despite these facts, Primoseius is included as a subgenus of Kleemannia in the list of valid taxa of Ameroseiidae in this paper. It may be reliably distinguished from *Kleemannia* s. str. mainly by the absence of z6, and presence of specifically modified dorsal setae (conspicuously flattened, leaf- to feather-shaped). *Primoseius* currently comprises 12 described species (*bisetae*, *dipankari*, *dubitatus*, *eumorphus*, *macauleyi*, *mineiro*, *parplumosa*, *plumosoides*, *plumosa*, *potchefstroomensis*, *pseudoplumosa* and *wahabi*), of which only eight species are considered here to be valid (*bisetae*, *dipankari*, *mineiro*, *parplumosa*, *plumosoides*, *plumosa*, *pseudoplumosa* and *wahabi*). Narita et al. (2013b) classified eight species of „*Ameroseius*“ into a species group named *plumosus*-group (*dipankari*, *eumorphus*, *mineiro*, *parplumosa*, *plumosa*, *potchefstroomensis*, *reticulatus* and *wahabi*). They defined the group by the following character states, of which some are vague and applicable also for other groups inside the genus: corniculus bifid or trifid; dorsal shield reticulate and without pit-like depressions; 26 or 28 pairs of dorsal shield setae, most of which are lanceolate to leaf-shaped; and five or six pairs of opisthogastric setae, two pairs of which are on ventrianal shield. The *plumosus*-group of Narita et al. (2013b) could be considered to be identical with *Primoseius*, and it omits three species described before 2013, namely *Kleemannia bisetae*, *Kleemannia plumosoides* and *Kleemannia pseudoplumosa*, and inexplicably includes *Kleemannia reticulata*, which has z6 clearly developed and present on the dorsal shield.

*Kleemannia* currently comprises 28 valid species described mostly from the Palaearctic, and only rarely from Neotropics (four species), Nearctic (one species), Afrotropics (one species) and Oriental Region (two species). Specimens reported as *plumosa* and *plumigera* have been reported from many countries around the world, from various natural and synanthropic habitats (stored grain and food, litter in sheds and stalls, mould in buildings). In Slovakia, this genus is represented by eight recorded species.

#### Key to species of *Kleemannia* occurring in Europe (females)

Partial keys to species of *Kleemannia* (classified in *Ameroseius*) from Europe and former Soviet Union may be found in Karg (1971a, 1993) and Bregetova (1977), and they include eight and 13 species, respectively. The world species can be identified using keys from Narita et al. (2013b, 2015). A new key to the genus is needed to include a species here firstly reported from Europe (*Kleemannia kosi* and *Kleemannia parplumosa*), the new species described in this study (*Kleemannia dolichochaeta* sp. n.), and a revised Berlese species previously considered to be dubious (*Kleemannia tenella*). The identification key for 12 species presented below is based on direct examination of type specimens or specimens considered to be conspecific with the type material, except for *Kleemannia elegans*.

Several diagnostic features can be useful in recognising of the *Kleemannia* species occurring in Europe, namely (1) presence/absence of z6 (absent in *dolichochaeta*, *parlplumosa*, *plumosa* and *pseudoplumosa*); (2) placement of st3 (on soft integument in *plumea* and *tenella*); (3) number of opisthogastric setae (with reduced number of five pairs in *insignis*, *plumea* and *tenella*); (4) presence/absence of cavity-like poroid structure on peritrematal shield (absent in *plumea*, *plumigera* and *tenella*); (5) placement of JV2 and JV3 on anterior portion of ventrianal shield (both setae on medial surface in *elegans*, *kosi*, *pavida* and *plumigera*; JV2 on anterior margin or surface in *dolichochaeta*, *insignis*, *parplumosa*, *plumosa* and *pseudoplumosa*; both setae on anterior margin or surface in *delicata* and *tenella*; and (6) relative length of Z- and S-setae (Z-setae apparently shorter than S-setae in *tenella*).

**Table:** 

1	Dorsal shield with 28 pairs of setae (z6 absent); dorsal setae flattened, feather- to leaf-shaped (subgenus Primoseius Womersley, 1956)	**2**
–	Dorsal shield with 29 pairs of setae (z6 present); dorsal setae variously formed, mostly tubiform, rod-shaped, lanceolate or oblanceolate (subgenus Kleemannia s. str.)	**5**
2	Sternal shield with inverted U-shaped sculptural structure on anterior surface, the structure with anterior margin scalloped and heavily sclerotised	***Kleemannia plumosa* (Oudemans, 1902)** (Plates 53–56)
–	Sternal shield with simple sculpture on anterior surface, smooth or reticulate	**3**
3	Epigynal shield with inverted U-shaped sculptural structure on anterior surface, the structure smooth and well sclerotised	***Kleemannia parplumosa* Nasr & Abou-Awad, 1986** (Plate 45)
–	Epigynal shield with simple sculpture on anterior surface, smooth or reticulate	**4**
4	Setae j5 apparently shorter than J2 and J4, with tips reaching between bases of j5 and j6	***Kleemannia pseudoplumosa* (Rack, 1972)** (Plates 57–60)
–	Setae j5 notably lengthened, similar in length to J2 and J4, with tips reaching beyond bases of j6	***Kleemannia dolichochaeta* sp. n.** (Figure 3, Plate 38)
5	Epigynal shield with inverted U-shaped sculptural structure on anterior surface, the structure smooth and well sclerotised	***Kleemannia plumea* Oudemans, 1930** (Plates 48, 49)
–	Epigynal shield with simple sculpture on anterior surface, smooth or reticulate	**6**
6	Ventrianal shield with at least one pair of opisthogastric setae (JV2, sometimes also JV3) on or very close to its anterior edge	**7**
–	Ventrianal shield with both pairs of opisthogastric setae inserted well inside the anterior portion of the shield	**9**
7	Dorsal shield with j-setae apparently shorter than J-setae; setae j6 with tips reaching between bases of j6 and J2; dorsal shield densely granulated; ventrianal shield wider than long, rugose	***Kleemannia insignis* (Bernhard, 1963)** (Plates 39, 40)
–	Setae j1–j6, J2 and J4 similar in length; setae j6 reaching beyond bases of J2; idiosomal shields not granulated or rugose; ventrianal shield about as wide as long	**8**
8	Setae z5, z6, and Z1–Z3 apparently shorter than adjacent setae in central and latero-marginal rows; setae st3 on soft integument; five pairs of opisthogastric setae present (JV4 absent); peritrematal shields without conspicuous poroid structures	***Kleemannia tenella* (Berlese, 1916)** (Plates 61–63)
–	Setae z5, z6, and Z1–Z3 relatively long, almost subequal to those in central and latero-marginal rows; setae st3 on small suboval pseudo-metasternal platelets; six pairs of opisthogastric setae present (JV4 developed); peritrematal shields each with a cavity-like poroid structure	***Kleemannia delicata* (Berlese, 1918)** (Plates 35–37A, 37C, 76A, 76H, 77A)
9	Pseudo-metasternal platelets partly fused to sternal shield; basal part of sperm induction pores conspicuously thickened and heavily sclerotised	***Kleemannia plumigera* Oudemans, 1930** (Plates 50–52, 76B, 76D, 77C, 77D)
–	Pseudo-metasternal platelets free from sternal shield; basal part of sperm induction pores weakly sclerotised	**10**
10	Ventrianal shield slightly constricted laterally (posteriad of setae JV3); transverse row of setae j6, z6, s5 and r5 with j6 longest; setae J4 relatively longer, with tips reaching to or beyond posterior margin of dorsal shield; dorsal shield unevenly reticulate	***Kleemannia kosi* El-Badry, Nasr & Hafez, 1979** (Plates 41, 42)
–	Ventrianal shield regularly curved laterally; transverse row of setae j6, z6, s5 and r5 with subequal setae (z6 slightly shorter); setae J4 relatively shorter, reaching between their bases and posterior margin of dorsal shield; dorsal shield entirely reticulate	**11**
11	Medial dorsal setae rather lanceolate	***Kleemannia elegans* (Bernhard, 1963)**
–	Medial dorsal setae rather oblanceolate	***Kleemannia pavida* (C. L. Koch, 1839)** (Plates 37B, 46, 47)

### Kleemannia bella

Taxon classificationAnimaliaMesostigmataAmeroseiidae

(Barilo, 1987) comb. n.

Ameroseius bellus Barilo, 1987: 1267.

#### Type depository.

Zoological Institute, Russian Academy of Sciences, Saint Petersburg, Russia; Department of Invertebrate Zoology, Faculty of Biology, Samarkand University, Uzbekistan.

#### Type locality and habitat.

Uzbekistan, Karakalpakstan, Nukus Botanical Gardens, bank of furrow channel, soil substrate.

### Kleemannia bengalensis

Taxon classificationAnimaliaMesostigmataAmeroseiidae

Bhattacharyya, 1972

Kleemannia bengalensis Bhattacharyya, 1972: 131.

#### Type depository.

Zoological Survey of India, Calcutta, India.

#### Type locality and habitat.

India, West Bengal, Calcutta, Ballygunge, campus of the Calcutta University College of Science, edge of a pond, soil litter under decomposed water hyacinth, *Eichhornia crassipes* (Pontederiaceae).

#### Comparative material.

India: 1 ♂, 1 ♀ (BMNH: 65/633/20, male holotype, female allotype) – 14. 1. 1964, Calcutta, University, Ballygunge, soil litter under decomposed *Eichhornia crassipes*, sides of a pond.

### Kleemannia bisetae

Taxon classificationAnimaliaMesostigmataAmeroseiidae

(Karg, 1994) comb. n.

Ameroseius bisetae Karg, 1994b: 197.

#### Type depository.

Museum für Naturkunde, Berlin, Germany.

#### Type locality and habitat.

Ecuador, Galápagos Islands, Bartolomé Island, Pinnacle Rock, mangroves of coastal zone, in rotten leaves and wood detritus.

#### Comparative material.

Ecuador: 1 ♀ (ZMB: 44922, holotype) – 12. 2. 1985, Galapagos I., Bartolomé, Uferzone, 85-139, 6653.

#### Remarks.

The original description given by Karg (1994b) requires the following amendment: (1) dorsal shield with 28 pairs of setae, z6 absent; (2) sternal and epigynal shields without conspicuous specific sculpture, unevenly and weakly reticulate; (3) peritrematal shields each with a cavity-like poroid structure at the level of coxae III; (4) genu III, and tibiae III–IV each with two anterolateral and two posterolateral seta; (5) left JV2 on anterior margin of ventrianal shield (in the original description, this seta is stated to be on soft integument and outside the ventrianal shield).

### Kleemannia curvata

Taxon classificationAnimaliaMesostigmataAmeroseiidae

(Gu, Wang & Bai, 1989) comb. n.

Ameroseius curvatus Gu, Wang & Bai, 1989: 48.

#### Type depository.

Institute of Endemic Disease Control, Ningxia Autonomous Region, Yinchuan, China.

#### Type locality and habitat.

China, Ningxia Autonomous Region, Zhongning County, on midday gerbil, *Meriones meridianus* (Mammalia, Rodentia).

#### Remarks.

A species closely related with *Kleemannia delicata* (Berlese, 1918), if not identical.

### Kleemannia delicata

Taxon classificationAnimaliaMesostigmataAmeroseiidae

(Berlese, 1918) comb. n.

Plates 35 , 36 , 37A , 37C , 76A , 76H , 77A

Ameroseius delicatus Berlese, 1918: 144.
Lasioseius (Lasioseius) gracilis Halbert, 1923: 369. **Syn. n.**
Ameroseius (Kleemania) (sic) gracilis. — Athias-Henriot 1959: 192.
Ameroseius delicatus . — Westerboer and Bernhard 1963: 474; Karg 1971a: 230; Bregetova 1977: 158; Castagnoli and Pegazzano 1985: 109; Karg 1993: 227.
Ameroseius gracilis . — Westerboer and Bernhard 1963: 535; Karg 1971a: 229; Bregetova 1977: 156; Karg 1993: 227; Christian and Karg 2006: 238.
Ameroseius stramenis Karg, 1976: 538. **Syn. n.**
Kleemania (sic) gracilis. — Luxton 1998: 21.

#### Type depository.

Of *Ameroseius delicatus* – Istituto Sperimentale per la Zoologia Agraria, Firenze, Italy (holotype not designated); of *Ameroseius gracilis* – National Museum of Ireland, (cited as Irish National Museum), Dublin, Ireland; of *Ameroseius stramenis* – Museum für Naturkunde, Berlin, Germany; Hungarian Natural History Museum, Budapest, Hungary.

#### Type locality and habitat.

Of *Ameroseius delicatus* – locality not specified (Firenze, Padova, Italy), in hay; of *Ameroseius gracilis* – Ireland, Dublin, Albert Model Farm, on sprouting potatoes; of *Ameroseius stramenis* – Chile, Santiago Province, Santiago (slide label: El Arrayán), humid leaf litter.

#### Comparative material.

Chile: 1 ♀ (ZMB: 41662, holotype) – 9. 10. 1965, El Arrayan (Prov. Santiago), aus dicker Laubschicht, 2997 (labelled *Ameroseius stramenis*). Germany: 1 ♀ (ZMB: 41009) – Jun. 1958, Müncheberg, Luzerne (labelled *Ameroseius plumigera*); 1 ♀ (ZSM: Coll. Hirschm./Willm. 1) – Waging am See, Neubau, leg. O. Brandt, M. Postner, det. W. Hirschmann; 78 ♀♀ (ZSM: Coll. Hirschm./Willm. 2) – 26. 4. 1963, München, Neubau, leg. M. Postner, det. W. Hirschmann; 19 ♀♀ (ZMB: 41008) – 17. 10. 1963, Kiel, Neubau, leg. F. Bolle, det. G. Rack, redet. G. O. Evans in 1964, 2987, A19/63 (labelled *Ameroseius plumigera*); 4 ♀♀ (ZSM: Coll. Popp P483/1) – Sep. 1968, Pei Benberg, Neubau, leg. Ludwig, det. Popp; 10 ♀♀ (ZMH: A26/69) – 14. 7. 1969, Bomlitz, Einfamilienhaus-neubau, leg. H. Wasser, det. G. Rack in 1969 (labelled *Kleemannia plumigera*); 1 ♀ (ZMH: A41/73) – May 1973, Kassel, Neubau, leg. H. Kühne, det. G. Rack in 1973 (labelled *Ameroseius plumigerus*); 9 ♀♀ (ZMH: A12/74) – Oct. 1973, Bez. Hannover, Neubau, leg. E. Gersdorf, det. G. Rack in 1973 (labelled *Ameroseius plumigerus*); 2 ♀♀ (ZMH: A11/74) – 25. 10. 1973, Meinhard-Grebendorf/Niedersachsen, Neubauwohnung, leg. H. Wenderoth, det. G. Rack in 1973 (labelled *Ameroseius plumigerus*); 14 ♀♀ (ZMH: A53/73) – 5. 11. 1973, Uetze (Niedersachsen), massenhaft in den Räumen einer Firma, leg. U. Zellentin, det. G. Rack in 1973 (labelled *Ameroseius plumigerus*); 38 ♀♀ (ZMH: A78/89) – 25. 10. 1988, Bergisch-Gladbach, massenhaft in einer Wohnung nach Neuverlegung des Parketts, leg. U. Lambert, det. G. Rack in 1988 (labelled *Ameroseius plumigerus*). Ireland: 1 ♀ (NMINH: 2013.3.1, holotype) – no collection data (labelled *Lasioseius gracilis*). Italy: 4 ♀♀, 1 ♂ (ISZA: 190/15, 190/16, 194/18, syntypes) – Firenze, fiorume stalle; 2 ♀♀ (ISZA: 190/17, syntypes) – Firenze, fenili; 4 ♀♀ (ISZA: 210/37, 210/38) – Padova, fiorume.

#### New material from Slovakia.

Trnavská Pahorkatina Wold: 2 ♀♀, 4 ♂♂ – 18. 11. 2005, Horná Streda Village, cellar of a single-family house, decaying potatoes with soil-sand substrate, altitude 170 m, leg. P. Mašán; 9 ♀♀ – 13. 10. 2012, Horná Streda Village, cellar of a single-family house, decaying potatoes with soil-sand substrate, altitude 170 m, leg. P. Mašán.

#### Remarks.

There are six slides of this species in the Berlese Acaroteca (190/15–17, 194/18, 210/37–38). These slides contain at least 12 specimens, including those of *Ameroseius molliculus* (nomen nudum) and *Ameroseius dubitatus*, all collected in Italy (Firenze and Padova), and not labelled as „tipico”. Without doubt, those from Firenze belong to the original type specimens of Berlese. They are mostly in bad condition to be examined. However, an examination of the specimens showed specific features also detected in well preserved specimens available for study from Slovakia and other regions of Europe.

Based on an examination of the type specimens, *Ameroseius stramenis* Karg, 1976 and *Lasioseius gracilis* Halbert, 1923 are considered new junior synonyms of *Kleemannia delicata* (Berlese, 1918). The two former species were briefly described in their original descriptions. Halbert (1923) did not inform the number of setae on the dorsum, but his illustration shows 26–27 setae, lacking z6. Karg (1971a, 1993) slightly modified Halbert’s illustration to show 27 setal pairs. I could detect the full complement of 29 pairs of setae in type series of both, *delicatus* and *gracilis*. The re-description and illustrations given by Westerboer and Bernhard (1963) for *delicatus* do not require serious amendment, except for the shape of ventrianal shield. This shield is usually not so regularly subcircular as illustrated, but almost truncate and slightly concave anteriorly. In *A. stramenis*, Karg’s (1976) interpretation of curved epistome seems to be apparently atypical for the genus, but I could not confirm it in the single available type specimen loaned from Berlin Collection (the epistome is obscure and not observable). In addition, the female of *A. stramenis* was illustrated by Karg as lacking metapodal platelets and one pair of opisthogastric setae (JV4), although both structures were reliably detected in an axamination of the holotype. Examination of numerous specimens from various mueum collections showed that *Kleemannia delicata* is fairly common in buildings in Europe and has often been misidentified, mainly as *Kleemannia plumigera*.

### Kleemannia dipankari

Taxon classificationAnimaliaMesostigmataAmeroseiidae

(Bhattacharyya, 2004) comb. n.

Ameroseius dipankari Bhattacharyya, 2004: 2.
Ameroseius dispankari (sic). — Moraza 2006: 165.
Ameroseius dipankari . — Narita et al. 2013b: 2324.

#### Type depository.

Zoological Survey of India, Jhalamand, Jodhpur, Rajasthan, India.

#### Type locality and habitat.

India, Rajasthan, semi-dried drainage system, decomposed grass litter.

#### Remarks.

Based on the illustrations given by Nasr and Abou-Awad (1986) for *Kleemannia parplumosa* and Bhattacharyya (2004) for *Kleemannia dipankari*, both species are remarkably similar in having nearly identical inverted U-shaped sculptural structure on the anterior portion of the epigynal shield. Adult females of only a few *Kleemannia* species (namely *dipankari*, *plumea* and *parplumosa*) have been described as having this specific ornamentation structure. Other similarities include an elongate idiosoma; form and relative length of dorsal setae; relative size, shape and arrangement of ventral shields; and form of epistome, with lateral margins straight. Therefore, I suspect these two species to be conspecific. However, the illustration of Bhattacharyya (2004) shows the dorsal and ventral chaetotaxy to be quite dissimilar to those of *K. parplumosa* in lacking two pairs of dorsal shield setae (s1 or z2, and s4), and a pair of opisthogastric setae (JV4), usually placed on soft integument close to lateral margins of ventrianal shield. However, I believe the complement of dorsal and opisthogastric setae to be the same as in *K. parplumosa*, some setae being inadvertently omitted in its original description.

### Kleemannia dolichochaeta sp. n.

Taxon classificationAnimaliaMesostigmataAmeroseiidae

http://zoobank.org/9BEE9397-2906-41A7-AA1E-BBFF69F24BCB

Figure 3 , Plate 38

#### Type material.

Spain: 1 ♀ (IZSAV, holotype) – 18. 9. 2005, Balearic Islands, Mallorca, Serra de Tramuntana, Coll de Sa Bataia, side of Puig de Massanella Mount, forest soil detritus, leg. I. Országh.

#### Diagnosis.

Dorsal shield with 28 pairs of setae (z6 absent); setae relatively narrow, sword-shaped. Setae j5 and j6 conspicuously lengthened, subequal to J2 and J4, and with tips reaching beyond bases of j6 and J2, respectively. Ventrianal shield with two pairs of opisthogastric setae (JV2, JV3); setae JV2 close to anterior margin of the shield. Setae JV4 present.

#### Description.

Female. Idiosoma suboval, widened medially and narrowed anteriorly, 385 μm long and 255 μm wide. Dorsal shield normally sclerotised, evenly and entirely reticulate, with several pairs of very shallow and indistinct depressions on anterior surface, covering whole dorsal surface, and bearing 28 pairs of setae. Dorsal setae spatulate, with parallel lateral margins and central longitudinal rib; setal margins smooth or serrate. Setae in central rows longer (up to 100 μm) than those in submarginal (60–70 μm) and marginal rows (50–60 μm). Setae j5 and j6 conspicuously lengthened, subequal to J2 and J4, longer than distance between their bases and bases of j6 and J2, respectively. Setae J2 with tips reaching beyond bases of J4, and setae J4 reaching beyond posterior margin of idiosoma. Setae j1 relatively short, 19–22 μm long, with spines on lateral margins. The length of some selected dorsal setae as follows: j4 50–55 μm, j5 and j6 77–85 μm, J2 90–95 μm, J4 93–100 μm, z5 68–73 μm. Sternal shield subquadrate, with anterolateral corners between coxae I and II, 72 μm in length and width (at level of st2), reticulate, with two pairs of setae (st1, st2). Pseudo-metasternal platelets present, relatively large, subcircular, each bearing a seta (st3) and poroid structure. Metasternal setae (st4) on soft integument. Epigynal shield relatively shorter and wider, 98 μm in width, slightly expanded behind genital setae, almost trapezoidal, with reticulate pattern and a pair of genital setae (st5). Three pairs of narrow and transverse postgenital sclerites present. Metapodal platelets suboval. Peritremes and peritrematal shields normally developed. Six pairs of opisthogastric setae present (JV4 expressed). Ventrianal shield 143 μm wide and 123 μm long, with medial anterior margin moderately concave and posterior margin widely rounded, almost pentagonal, well reticulate, having three circum-anal setae and two pairs of opisthogastric setae. Except for JV5, all ventrally situated setae smooth and needle-like; sternal setae 21–24 μm in length, slightly longer than opisthogastric and adanal setae (16–18 μm), and postanal seta (23 μm). Setae JV5 of similar form and length as those on dorsal shield, 62–66 μm long. Epistome with central spine-like process. Setae h1 thickened. Fixed digit of chelicera with four small teeth on proximal masticatory area. Other gnathosomal structures and leg chaetotaxy typical of genus.

#### Etymology.

The name is derived from the Greek words „*dolichos*“ (long) and „*chaete*“ (bristle), and it expresses one of the most important features of the species—the dorsal shield bears the longest setae among the known members of the subgenus Primoseius, especially in its anterior medial portion.

### Kleemannia elegans

Taxon classificationAnimaliaMesostigmataAmeroseiidae

(Bernhard, 1963) comb. n.

Ameroseius elegans Bernhard (in Westerboer & Bernhard, 1963: 483).
Ameroseius elegans . — Karg 1971a: 233; Bregetova 1977: 158; Karg 1993: 230.

#### Type depository.

Not stated.

#### Type locality and habitat.

Germany, Erlangen, field habitat, in heap of weed plants.

### Kleemannia guyimingi

Taxon classificationAnimaliaMesostigmataAmeroseiidae

(Ma, 1997) comb. n.

Ameroseius guyimingi Ma, 1997: 140.
Ameroseius magnisetosa . — Bregetova 1977: 150, 152. **Misidentification.**
Ameroseius qinghaiensis Ma, 2008: 748. Synonymy by Ho et al. (2010), based on unpublished data of Ma. Junior primary homonym of Ameroseius qinghaiensis Li & Yang, 2000.
Ameroseius guyiming (sic). — Ho et al. 2010: 92.
Ameroseius chinensis Khalili-Moghadam & Saboori, 2016: 546 (nom. n. pro Ameroseius qinghaiensis Ma, 2008). **Syn. n.**
Ameroseius qinghaiensis . — Khalili-Moghadam and Saboori 2016: 547. not Ameroseius qinghaiensis Li & Yang, 2000: 65.

#### Type depository.

Of *Ameroseius guyimingi* – National Base of Plague and Brucellosis Control, Jilin Province, Baicheng; of *Ameroseius qinghaiensis* – Qinghai Institute for Endemic Disease Prevention and Control, Qinghai, China.

#### Type locality and habitat.

Of *Ameroseius guyimingi* – China, Jilin Province, Qianguoerluosi Mongolian Autonomous County, on house mouse, *Mus musculus* (Mammalia, Rodentia); of *Ameroseius qinghaiensis* – China, Qinghai Province, Huangzhong County, on brown rat, *Rattus norvegicus* (Mammalia, Rodentia).

### Kleemannia insignis

Taxon classificationAnimaliaMesostigmataAmeroseiidae

(Bernhard, 1963) comb. n.

Plates 39 , 40

Ameroseius insignis Bernhard (in Westerboer & Bernhard, 1963: 487).
Ameroseius insignis . — Karg 1971a: 230; Vasilyeva et al. 1976: 25; Bregetova 1977: 160; Karg 1993: 227; Kazemi and Rajaei 2013: 65; Nemati et al. 2013: 19; Narita et al. 2015: 395.
Ameroseius marginalis Fan & Li, 1993: 17. **Syn. n.**
Ameroseius sichanensis (sic) Fan & Li, 1993: 18, 20. **Syn. n.**
Ameroseius sichuanensis . — Fan and Li 1993: 17, 19.
Ameroseius (Kleemannia) insignis . — Hajizadeh et al. 2013a: 151.
Ameroseius marginalis . — Narita et al. 2015: 395.

#### Type depository.

Of *Ameroseius insignis* – not stated (holotype not designated); of *Ameroseius marginalis* – Depertment of Plant Protection, Fujian Agricultural College, Fuzhou, China; Depertment of Plant Protection, Southwest Agricultural University, Chongqing, China; of *Ameroseius sichuanensis* – Depertment of Plant Protection, Fujian Agricultural College, Fuzhou, China; Depertment of Plant Protection, Southwest Agricultural University, Chongqing, China.

#### Type locality and habitat.

Of *Ameroseius insignis* – Germany, Erlangen, Nürnberg, from horse dung, rabbit dung, and rotting leaves of northern water hemlock, *Cicuta virosa* (Apiaceae); *Ameroseius marginalis* – China, Sichuan, Chongqing, from bean stick; of *Ameroseius sichuanensis* – China, Sichuan, Chongqing, on jelly ear fungus, *Auricularia auricula-judae* (Basidiomycota, Auriculariaceae).

#### New material from Slovakia.

Podunajská Rovina Flatland: 1 ♀ – 28. 7. 2004, Svätý Jur Town, Šúr Forest, alder forest, nest of *Passer monatnus* (Aves), artificial nest box, straw litter, altitude 130 m, leg. K. Sobeková and P. Puchala.

#### Remarks.

Based on primary description of Bernhard (in Westerboer and Bernhard 1963), and re-description of Vasilyeva et al. (1976), females of *Kleemannia insignis* may be distinguished from those of all other members of the genus by the insertion of JV2 on soft integument; these setae are illustrated as being out but adjacent to the anterior edge of ventrianal shield. In the only Slovak specimen available for study, my examination showed that there can be an alternative position of these setae because they are both clearly on the shield, adjacent to its anterior margin.

Based on the original illustrations of Fan and Li (1993), *Ameroseius marginalis* perfectly fits to *Kleemannia insignis* from Europe (dorsal and ventral chaetotaxy, specific sculptural granulation of dorsal shield, sculpture of metapodal platelets, shape and arrangement of ventral shields, etc.). Unfortunately, differential characters for separating *A. marginalis* (and also *A. sichuanensis*) from *K. insignis* given by Fan and Li (1993) are founded on variable (shape of pseudo-metasternal platelets, relative length of J2) or misinterpreted characters (in the original description of *insignis*, the number of teeth on fixed digit is stated to be five instead of four).

### Kleemannia kosi

Taxon classificationAnimaliaMesostigmataAmeroseiidae

El-Badry, Nasr & Hafez, 1979

Plates 41 , 42

Kleemania (sic) kosi El-Badry, Nasr & Hafez, 1979: 5.
Kleemannia kosi . — Mohamed et al. 1985: 98; Zaher 1986: 402.
Kleemannia Kosi (sic). — Mohamed et al. 1985: 99.

#### Type depository.

Not stated.

#### Type locality and habitat.

Egypt, Kena Governorate, Koa, in debris under orange tree, Citrus × sinensis (as *Citrus sininses*) (Rutaceae).

#### Comparative material.

Germany/India: 2 ♀♀ (ZMB: 40281) – 2. 5. 1969, Zwiebeln 301, Indien, „Meyenburg“, 2989 (labelled *Ameroseius gracilis*); 2 ♀♀ (ZMH: A102/85) – Nov. 1983, Hamburg, eingeschleppt mit gesacktem Pfeffer, Jutesäcke, leg. Hüter (Degesch), det. G. Rack in 1983 (labelled Ameroseius cf. plumigerus).

#### Remarks.

El-Badry et al. (1979) and Zaher (1986) gave only a brief description of *Kleemannia kosi*. So, my identification of this species was initially based on insufficiently detailed illustrations available in their papers, later also on detailed photos of specimens stated to be *kosi* and originated from Egypt (kindly sent by R. Abo-Shnaf).

Together with other ameroseiid species such as *Kleemannia parplumosa* and *Sertitympanum nodosum*, it can represent a group of stored product mites unintentionally introduced to Europe by various human activities (see comparative material above).

### Kleemannia longisetosa

Taxon classificationAnimaliaMesostigmataAmeroseiidae

(Ye & Ma, 1993) comb. n.

Ameroseius longisetosus Ye & Ma, 1993: 87.

#### Type depository.

Xinjiang Institute for Endemic Disease Control and Research, Urumqi, China.

#### Type locality and habitat.

China, Xinjiang Region, Qitai County, Beita Mountain, yellow steppe lemming, *Eolagurus luteus* (as *Lagurus luteus*) (Mammalia, Rodentia).

### Kleemannia mineiro

Taxon classificationAnimaliaMesostigmataAmeroseiidae

(Narita, Bernardi & Moraes, 2013) comb. n.

Ameroseius mineiro Narita, Bernardi & Moraes (in Narita et al. 2013b: 2313).
Ameroseius mineiro . — Khalili-Moghadam and Saboori 2016: 544.

#### Type depository.

Departamento de Entomologia e Acarologia, Escola Superior de Agricultura “Luiz de Queiroz”, Universidade de São Paulo, Piracicaba, São Paulo, Brazil; Coleção de Invertebrados, Setor de Zoologia, Departamento de Biologia, Universidade Federal de Lavras, Lavras, Minas Gerais State, Brazil.

#### Type locality and habitat.

Brazil, Minas Gerais State, Curvelo, Lapa do Mosquito Cave, in bat guano.

#### Comparative material.

Brazil: 2 ♀♀ (ESALQ: T-MZLQ 3350 C=7767, holotype and paratype) – 2004, Lapa do Mosquito, Curvelo – MG, Ex: guano, leg. L. F. O. Bernardi.

#### Remarks.

I have examined a slide labelled “Holotype” of *Kleemannia mineiro*, a species decribed by Narita et al. (2013b) from Brazil. The slide contains two individuals. They are both females, and are clearly conspecific, in a perfect agreement with the original description and illustrations. I here designate as lectotype the specimen nearer to the top edge of the slide when the labels are in the upright position. I have ringed this specimen with black ink and labelled the slide with the words „Lectotype ringed“.

### Kleemannia miranda sp. n.

Taxon classificationAnimaliaMesostigmataAmeroseiidae

http://zoobank.org/1ECA403E-FDB9-4B8C-8944-41795FFD0B24

Figure 4

#### Type material.

U.S.A.: 1 ♀ (BMNH, holotype) – 16. 2. 1962, Kansas, Douglas County, Lawrence, from tree hole, leg. W. W. Moss; 1 ♀ (BMNH, paratype) – 19. 10. 1955, Maryland, Prince George’s County, Laurel, Patuxent Wildlife Refuge, from nest of *Peromyscus* sp. (Rodentia, Cricetidae), leg. R. O. Drummond.

#### Diagnosis.

Dorsal shield with 29 pairs of setae (z6 present). Z-setae not reduced in length, at least as long as those in S-setal rows; setae J2 and J4 longest, j1 shortest. Sternal shield with three pairs of sternal setae (st1–st3). Ventrianal shield with two pairs of setae (JV2, JV3); setae JV2 close to anterior margin of the shield. Postgenital soft integument with three pairs of narrow and transverse sclerites.

#### Description.

Female. Idiosoma suboval, narrowed anteriorly, 350–370 μm long and 220–245 μm wide. Dorsal shield weakly sclerotised, scrobiculate laterally, smooth or delicately reticulate medially and posteromarginally, covering whole dorsal surface, and bearing 29 pairs of setae. Most of dorsal setae similar in length, relatively thin and regularly covered by minute spines; setae z5, z6 and S-setae 40–50 μm long; Z-setae 50–60 μm long; J2 77–81 μm and J4 83–87 μm long; setae j1 shortest, at most 20 μm in length, laterally with prominent thorns. Sternal shield subquadrate, with anterolateral corners well developed between coxae I and II, 68–71 μm in length and width (at level of st2), weakly reticulate on surface, with three pairs of setae (st1–st3). A pair of poroid structures associated with st3 on soft integument close to posterolateral corners of sternal shield. Pseudo-metasternal platelets absent. Metasternal setae (st4) on soft integument. Epigynal shield 86–90 μm wide, oblong, slightly expanded behind genital setae, tongue-shaped, with indistinct reticulate pattern and a pair of genital setae (st5). Three pairs of postgenital sclerites present. Metapodal platelets suboval, relatively small. Peritremes and peritrematal shields normally developed. Ventrianal shield 125–140 μm wide and 100–110 μm long, straight or very slightly concave anteriorly and widely rounded posteriorly, almost dish-shaped, well reticulate on surface, having three circum-anal setae, two pairs of opisthogastric setae and anus in its posteromedial portion. Except for JV5, all ventrally situated setae smooth and needle-like; sternal setae about 25 μm in length, slightly longer than ventral and adanal setae (15–17 μm), and postanal seta (20 μm). Setae JV5 of similar form and length as those on dorsal shield, 46–48 μm long. Epistome with central spine-like process. Setae h1 thickened. Fixed digit of chelicera with four small teeth on proximal masticatory area. Other gnathosomal structures and leg chaetotaxy typical of genus.

#### Taxonomic notes.

Sternal shield chaetotaxy, with st3 present, is unique and quite unlike any other species in the genus, in which these setae are off the shield, either on soft integument or small pseudo-metasternal platelets.

#### Etymology.

Derived from the Latin word „*mirandus*“ (strange)—based on unique sternal shield chaetotaxy described in previous paragraph.

### Kleemannia multus

Taxon classificationAnimaliaMesostigmataAmeroseiidae

(Gu, Wang & Bai, 1989) comb. n.

Ameroseius multus Gu, Wang & Bai, 1989: 49.

#### Type depository.

Institute of Endemic Disease Control, Ningxia Autonomous Region, Yinchuan, China; Department of Parasitology, Guiyang Medical College, Guiyang, China.

#### Type locality and habitat.

China, Ningxia Autonomous Region, Zhongning County, on midday gerbil, *Meriones meridianus* (Mammalia, Rodentia).

#### Remarks.

A species related with *Kleemannia pavida* (C. L. Koch, 1839).

### Kleemannia nova

Taxon classificationAnimaliaMesostigmataAmeroseiidae

Nasr & Abou-Awad, 1986

Plates 43 , 44

Kleemannia nova Nasr & Abou-Awad, 1986: 75.
Ameroseius (Kleemannia) novus . — Hajizadeh et al. 2013a: 150.
Ameroseius nova . — Kazemi and Rajaei 2013: 65.

#### Type depository.

National Research Centre, Dokki, Cairo, Egypt.

#### Type locality and habitat.

Egypt, Kafr El-Sheikh Governorate, Sakha, in manure.

#### Comparative material.

Iran: 1 ♀ (CJH) – Jiroft, Kerman Province, soil sample, leg. J. Hajizadeh.

#### Remarks.

I examined a female from Iran. This species may be reliably distinguished from other congeners by the following character states: (1) setae z6 present, (2) setae j1 fan-shaped and marginally serrate (other dorsal setae flattened, feather-shaped), (3) setae JV2 and JV3 on anteromedial surface of ventrianal shield, (4) pseudo-metasternal platelets enlarged and well developed, (5) prominent poroid structure on peritrematal shield present, (6) setae JV4 absent. Anterior margin of epistome in this species is produced into pointed central projection (originally illustrated as subtriangular, with rounded apex).

### Kleemannia parplumosa

Taxon classificationAnimaliaMesostigmataAmeroseiidae

Nasr & Abou-Awad, 1986

Plate 45

Kleemannia parplumosus Nasr & Abou-Awad, 1986: 76.
Ameroseius (Kleemannia) parplumosus . — Hajizadeh et al. 2013a: 150.
Ameroseius parplumosus . — Narita et al. 2013b: 2325; Kazemi and Rajaei 2013: 66; Nemati et al. 2013: 19; Khalili-Moghadam and Saboori 2016: 544.

#### Type depository.

National Research Centre, Dokki, Cairo, Egypt.

#### Type locality and habitat.

Egypt, Kafr El-Sheikh Governorate, Sakha, in manure.

#### Comparative material.

Germany/India: 2 ♀♀ (ZMB: 40281) – 2. 5. 1969, Zwiebeln 301, Indien, „Meyenburg“, 2989 (labelled *Ameroseius gracilis*). Iran: 1 ♀ (IZSAV) – Guilan Province, rice storage, 11, leg. J. Hajizadeh; 2 ♀♀ (CJH) – Jiroft, Kerman Province, soil sample, leg. and det. J. Hajizadeh.

#### Remarks.

I have examined several female specimens of this species from Iran (received from A. Ahadiyat and J. Hajizadeh). *Kleemannia parplumosa* is easily identifiable because the adult females of only two further congeneric species (*dipankari*, *plumea*) have been described as having their epigynal shield ornamented by a peculiar sculptural structure that is well sclerotised, inverted U-shaped and on anterior surface of the shield.

There is a slide with two females of this species in the Karg Acaroteca in Berlin (ZMB 40281). The slide bears other three specimens of two other species of *Kleemannia*, and a label with the following collection data: *Ameroseius gracilis* Halbert, 1923, Nr. 2989, Zwiebeln, 301, Indien, Meyenburg, 2. 5. 1969. Based on this inconsistent information, the origin of these specimens cannot be established with any confidence. I believe that specimen are collected in Meyenburg (a town in northern Germany), perhaps from a sample of onion imported from India.

### Kleemannia pavida

Taxon classificationAnimaliaMesostigmataAmeroseiidae

(C. L. Koch, 1839)

Plates 37B , 46 , 47

Zercon pavidus C. L. Koch, 1839: Fasc. 23: 15.
Seiulus plumosus . — Oudemans 1902: 17 (in part).
Hypoaspis pavidus . — Oudemans 1902: 19; Berlese 1904: 276.
Kleemannia pavidus . — Oudemans 1930: 137.
Ameroseius pavidus . — Westerboer and Bernhard 1963: 479; Karg 1971a: 233; Bregetova 1977: 155; Karg 1993: 230.
Ameroseius lanceosetis Livshits & Mitrofanov, 1975: 462. **Syn. n.**
Kleemannia pavida . — Evans and Till 1979: 230.
Ameroseius lanceosetis . — Ma 1996: 201; Hajizadeh et al. 2013a: 146.
Ameroseius (Kleemannia) lanceosetis . — Hajizadeh et al. 2013a: 151.
Ameroseius (Kleemannia) pavidus . — Hajizadeh et al. 2013a: 151.

#### Type depository.

Of *Kleemannia pavida* – not stated; of *Ameroseius lanceosetis* – Nikita Botanical Gardens, National Scientific Center, Yalta, Crimea, Russia (the type specimens not found and probably lost, based on personal communication from Alex Khaustov).

#### Type locality and habitat.

Of *Kleemannia pavida* – Germany, in unspecified substrate (in houses, especially stables, in hay and straw); of *Ameroseius lanceosetis* – Russia, Crimea, Alushtinskyi District, Privetnoye (as Privetnyi), in grain storage house.

#### Comparative material.

Germany: 1 ♀ (ZMB: 40282) – 25. 4. 1969, VAR, an Zwiebeln, mit *Tyrophagus putrescentiae* vergesell. (labelled *Ameroseius gracilis*); 11 ♀♀ (ZMH: A41/73) – May 1973, Kassel, Neubau, leg. H. Kühne, det. G. Rack in 1973. Iran: 16 ♀♀ (IZSAV) – Guilan Province, rice storage, No. 1, leg and det. J. Hajizadeh (labelled *Ameroseius lanceosetis*). Netherlands: 1 ♀ (RMNH: ACA.P.4294) – June 1928, Hengelo (O.), in hooi afval, boerderij, leg. Dr. Baudet; 1 ♀ (RMNH: ACA.P.4295) – Jan. 1927, Arnhem, in huis, leg. Oudemans; 1 ♀ (RMNH: ACA.P.4296) – Aug. 1921, Wapenvelde bij Apeldoorn, in hooi afval, Wageningen; 1 ♀ (RMNH: ACA.P.4297) – July 1896, Sneek, *Vespertilio dasycneme*, leg. Oudemans; 1 ♀ (RMNH: ACA.P.4299) – July 1928, Ophovizolder, Franeker, Utrecht, leg. Dr. Baudet; 3 ♀♀ (ZSM: Coll. Vitzthum/Kneissl V597) – July 1928, Franeker/Friesland, in Heuabfällen, vom Heuboden; 3 ♀♀ (ZMH: A74/74) – 1. 10. 1974, NL-Schayk, Kok, *Crocidura russula*, Zool. Lab. Nijmegen, det. G. Rack in 1974. United Kingdom: 1 ♀ (BMNH: 1955.6.1.91) – 27. 8. 1954, London, Zool. Gardens, hay, det. J. G. Sheals (labelled *Kleemania plumosus*).

#### New material from Slovakia.

Podunajská Rovina Flatland: 1 ♀ – 31. 7. 1991, Šuľany Village, willow-poplar flood plain forest (*Salici-Populetum*), fresh cadaver of *Clethrionomys glareolus* (Mammalia), altitude 120 m, leg. P. Mašán and J. Krištofík.

#### Remarks.

The Iranian specimens of this species, including those re-described under the name *lanceosetis* (Hajizadeh et al. 2013a), are in good agreement with the mites widely collected in Europe, and now deposited in a collection of various museums (see comparative material above).

### Kleemannia pennata

Taxon classificationAnimaliaMesostigmataAmeroseiidae

(Fox, 1949) comb. n.

Borinquolaelaps pennatus Fox, 1949: 39.
Ameroseius (Kleemania) (sic) pennatus. — Athias-Henriot 1959: 192.
Ameroseius pennatus . — Farrier and Hennessey 1996: 21.

#### Type depository.

School of Tropical Medicine, San Juan, Puerto Rico.

#### Type locality and habitat.

Puerto Rico, San Juan, Santuree, on brown rat, *Rattus norvegicus* (Mammalia, Rodentia).

#### Remarks.

The original description and illustrations of this species are not detailed enough to allow its recognition. Fox (1949) illustrated the dorsum of this species as having 24 pairs of setae (z6 present, but j1 and some of the anteriormost and marginal setae were not shown).

### Kleemannia pinicola

Taxon classificationAnimaliaMesostigmataAmeroseiidae

Ishikawa, 1972

Kleemannia pinicola Ishikawa, 1972: 97.
Ameroseius pinicola . — Bregetova 1977: 158; Gu et al. 1989: 46.

#### Type depository.

Biological Laboratory, Matsuyama Shinonome Junior College, Matsuyama, Japan.

#### Type locality and habitat.

Japan, Ehime Prefecture, Kashima Island, Hôjô, pine-wood grove, in humus.

### Kleemannia plumea

Taxon classificationAnimaliaMesostigmataAmeroseiidae

Oudemans, 1930

Plates 48 , 49

Seiulus plumosus . — Oudemans 1902: 17 (in part).
Kleemannia plumea Oudemans, 1930: 139.
Ameroseius plumeus . — Westerboer and Bernhard 1963: 535.
Ameroseius plumea . — Shcherbak (in Bregetova 1977: 149); Bregetova 1977: 150; Kazemi and Rajaei 2013: 66; Nemati et al. 2013: 19; Narita et al. 2015: 395.
Ameroseius tauricus Livshits & Mitrofanov, 1975: 462. Synonymy by Bregetova (1977).
Ameroseius (Kleemannia) plumea . — Hajizadeh et al. 2013a: 150. ? Ameroseius plumea. — Ehrnsberger and Błaszak 1999: 124.

#### Type depository.

Of *Kleemannia plumea* – National Museum of Natural History, Naturalis Biodiversity Center, Leiden, Netherlands; of *Ameroseius tauricus* – Nikita Botanical Gardens, National Scientific Center, Yalta, Crimea, Russia (the type specimens not found and probably lost, based on personal communication from Alex Khaustov).

#### Type locality and habitat.

Of *Kleemannia plumea* – Netherlands, Arnhem, on red squirrel, *Sciurus vulgaris* (Mammalia, Rodentia); of *Ameroseius tauricus* – Russia, Crimea, Nikita Botanical Gardens, tree hollow of oak (*Quercus* sp.), wood detritus.

#### Comparative material.

Netherlands: Slide RMNH: ACA.P.4300 (28. 10. 1896, Arnhem, *Sciurus vulgaris*, ♀ vent., leg. Oudemans), received for a study and labelled *Kleemannia plumea*, is bearing no mounted specimens.

#### Published material from Slovakia.

Laborecká Vrchovina Highland: Stakčín Village, Starina Dam, Gazdoráň Forest (Fenďa et al. 1998, Fenďa and Mašán 2003). Podunajská Rovina Flatland: Bratislava Capital, Botanical Gardens (Švaňa et al. 2006). Dobrohošť Village, Dunajské Kriviny Forest (Krumpál et al. 2001). Gabčíkovo Town, Ostrov Orliaka Morského Forest (Fenďa and Lengyel 2007). Svätý Jur Town, Šúr Forest (Fenďa et al. 1998, Švaňa et al. 2006, Fenďa et al. 2011). All records cited as *Ameroseius plumea*.

#### New material from Slovakia.

Burda Hills: 1 ♀ – 9. 11. 1997, Kamenica Nad Hronom Village, Kováčovské Kopce-juh Forest, oak forest (*Quercetum cerris*), leaf litter and soil detritus, altitude 330 m, leg. P. Mašán.

#### Remarks.

This species may be easily distinguished from other species of *Kleemannia* by the presence of inverted U-shaped sclerotised structure on epigynal shield, z6 on dorsal shield and absence of pseudo-metasternal platelets (st3 on soft integument).

### Kleemannia plumigera

Taxon classificationAnimaliaMesostigmataAmeroseiidae

Oudemans, 1930

Plates 50 , 51 , 52 , 76B , 76D , 77C , 77D

Kleemannia plumigera Oudemans, 1930: 140.
Zercoseius Gracei Hughes, 1948: 149. Synonymy by Evans (1954), and Hughes (1961).
Zercoseius gracei . — Turk 1953: 12.
Zercoseius plumigera . — Evans 1954: 798.
Lasioseius (Zercoseius) gracei . — Womersley 1956b: 116.
Kleemannia delicata . — Schweizer 1961: 130. **Misidentification.**
Kleemania (sic) plumigera. — Hughes 1961: 244.
Kleemannia plumigera . — Rack 1963: 408; Hughes 1976: 336.
Ameroseius plumigerus . — Karg 1971a: 229; Karg 1993: 226; Kazemi and Rajaei 2013: 66; Nemati et al. 2013: 19.
Ameroseius plumigera . — Bregetova 1977: 154; Narita et al. 2013b: 2321.
Ameroseius gilarovi Petrova (in Petrova & Koshchanova, 1986: 31). **Syn. n.**
Ameroseius gilarovi sp. n. — Koshanova 1987: 143.
Ameroseius (Kleemannia) plumigerus . — Hajizadeh et al. 2013a: 151.

#### Type depository.

Of *Kleemannia plumigera* – National Museum of Natural History, Naturalis Biodiversity Center, Leiden, Netherlands; of *Zercoseius gracei* – not stated (holotype not designated); of *Ameroseius gilarovi* – not stated.

#### Type locality and habitat.

Of *Kleemannia plumigera* – Netherlands, Helder, from common eelgrass, *Zostera marina* (Zosteraceae); of *Zercoseius gracei* – the United Kingdom, Northern Ireland, Belfast, in damp cracks in stone walls and in siftings of oats; *Ameroseius gilarovi* – Uzbekistan, Karakalpakstan, Nukus District, field with cotton, *Gossypium* sp. (Malvaceae), in soil substrate.

#### Comparative material.

Germany: 1 ♀ (BMNH) – 11. 10. 1963, Kiel-Suchsdorf, Neubau, leg. F. Bolle, Eing. Nr. A19/63 (labelled *Kleemannia* spec. 1); 2 ♀♀ (ZMH: A11/74) – 25. 10. 1973, Meinhard-Grebendorf/Niedersachsen, Neubauwohnung, leg. H. Wenderoth, det. G. Rack in 1973. Ireland: 1 ♀ (BMNH: E 2001-124) – 29. 12. 1946, Clontarf, Dublin, *Mus musculus*, F. A. Turk Collection, O. M. 749 (labelled *Ameroseius tenellus*). Iran: 2 ♀♀ (SHKC) – Feb. 2010, Fars Province, Koohmare-Sorkhi Region, leg. Sh. Yazdanpanah. Netherlands: 1 ♀ (RMNH: ACA.P.4301, syntype) – Sep. 1910, Helder, in zeegras (pakmateriaal), leg. Dr. Redeke.

#### Published material from Slovakia.

Bodvianska Pahorkatina Wold: Janík Village (Kováč et al. 1999, cited as *Ameroseius plumigerus*). Košická Kotlina Basin: Péder Village; Šebastovce Village; Valaliky Village (Kováč et al. 1999, cited as *Ameroseius plumigerus*). Slovenský Kras Karst: Ardovo Village, Ardovská Jaskyňa Cave; Kečovo Village, Jaskyňa Domica Cave (Kováč et al. 2005, cited as *Ameroseius plumigerus*). Silica Village, Majkova Jaskyňa Cave; Silická Brezová Village, Jaskyňa Milada Cave (Papáč et al. 2006, cited as *Ameroseius plumigerus*). Vihorlatské Vrchy Hills: Kaluža Village (Mrciak 1977, cited as *Kleemania plumigera*). Východoslovenská Pahorkatina Wold: Trhovište Village (Kováč et al. 1999, cited as *Ameroseius plumigerus*). Východoslovenská Rovina Flatland: Čičarovce Village; Hraň Village; Parchovany Village (Kováč et al. 1999, cited as *Ameroseius plumigerus*). Hnojné Village (Mrciak 1977, cited as *Kleemania plumigera*). Žitavská Pahorkatina Wold: Veľké Janíkovce Village (Sidor 1986).

#### New material from Slovakia.

Borská Nížina Lowland: 1 ♀ – 12. 3. 2017, Borský Svätý Jur Village, shed, soil detritus with sawdust, altitude 170 m, leg. P. Mašán. Čierna Hora Mts.: 1 ♀, 1 ♂ – 10. 8. 1996, Malá Lodina Village, Veľká Ružínska Jaskyňa Cave, bottom of cave, organic detritus, leg. Ľ. Kováč. Slovenský Kras Karst: 1 ♀ – 29. 4. 1997, Kečovo Village, NM Jaskyňa Domica Cave, Skupina Sôch Dome, alluvial clay substrate, altitude 360 m, leg. Ľ. Kováč; 1 ♀ – 13. 6. 1997, Ardovo Village, NM Ardovská Jaskyňa Cave, oak forest (*Corno-Quercetum*) with maple (*Acer* sp.), moss on rocky walls in cave entrance, altitude 315 m, leg. Ľ. Kováč; 8 ♀♀, 3 ♂♂ – 4. 9. 1997, Ardovo Village, Ardovská Jaskyňa Cave, Hlavná Chodba Dome, soil detritus with humus, altitude 330 m, leg. Ľ. Kováč; 2 ♀♀ – 4. 9. 1997, Ardovo Village, Ardovská Jaskyňa Cave, Vstupná Chodba Dome, fragments of rock with guano and wood detritus, altitude 330 m, leg. Ľ. Kováč; 5 ♀♀ – 4. 9. 1997, Ardovo Village, Ardovská Jaskyňa Cave, Zrútený Dóm Dome, soil detritus with humus under stone, altitude 330 m, leg. Ľ. Kováč.

#### Remarks.

The presence of a pair of pseudo-metasternal platelets anteriorly connected to the posterolateral margins of the sternal shield in female of *Kleemannia plumigera* distinguishes this species from females of all other species of *Kleemannia* and of other taxa of the Ameroseiidae. The female of *K. plumigera* was described by Oudemans (1930) and recently re-described by Narita et al. (2013b), as having pseudo-metasternal platelets separate and free from sternal shield, although this was not found by me in type female (numbered 4301) available in Oudemans Collection. Only Petrova (in Petrova and Koshchanova 1986) noted the partial fusion of pseudo-metasternal platelets with sternal shield in the specimens she described as *Ameroseius gilarovi*; other morphological characters mentioned in her description and detailed illustrations of *A. gilarovi* are in good agreement with those found in the type of *K. plumigera* (except for epistome, that seems to be misinterpreted). Therefore, a new synonymy is established between these two species in the present study. In one of the illustrations of specimens reported as *Kleemannia delicata* from Switzerland (here considered to be a misidentified *K. plumigera*), Schweizer (1961) only indistinctly indicated a connection between pseudo-metasternal platelets and sternal shield, but clearly and correctly depicted a pair of well sclerotised and prominent sperm induction pores associated with coxae III (another specific character of *K. plumigera* which is often overlooked in its desriptions).

Oudemans (1930) described the dorsal chaetotaxy of *Kleemannia plumigera* as having 30 pairs of setae. However, as pointed out by Narita et al. (2013b), this is an error, as the dorsal shield actually has a normal number of 29 pairs of setae. The identity of *K. plumigera* sensu Rack (Rack 1963, 1964, 1971, 1972) cannot be established with any confidence because of the absence of some information about the ventral idiosoma in her re-description of this species (Rack 1963), and incorrect identity of most of the revised specimens available in Hamburg Collection, labelled *K. plumigera* and identified by this author. A re-examination of the specimens described by Hughes (1948) as *Zercoseius gracei*, and later synonymised with *K. plumigera* (Evans 1954, Hughes 1961), would be necessary to confirm the conspecificity with *K. plumigera* or other related species, including *Kleemannia delicata*. Also Hughes (1948, 1961) illustrated the dorsal chaetotaxy of putative *K. plumigera* incorrectly, as having 25 and 28 pairs of setae, respectively.

### Kleemannia plumosoides

Taxon classificationAnimaliaMesostigmataAmeroseiidae

(Gu, Wang & Bai, 1989) comb. n.

Ameroseius plumosoides Gu, Wang & Bai, 1989: 46.
Ameroseius multus (sic). — Gu et al. 1989: 51.

#### Type depository.

Department of Parasitology, Guiyang Medical College, Guiyang, China.

#### Type locality and habitat.

China, Yunnan Province, Menglian County, on rat, *Rattus tanezumi* (as *Rattus flavipectus*) (Mammalia, Rodentia).

### Kleemannia plumosa

Taxon classificationAnimaliaMesostigmataAmeroseiidae

(Oudemans, 1902)

Plates 53 , 54 , 55 , 56

Seiulus plumosus Oudemans, 1902: 17 (in part).
Seiulus plumosus . — Berlese 1904: 276; Moraes et al. 2016: 250.
Lasioseius plumosus . — Hull 1918: 65.
Ameroseius dubitatus Berlese, 1918: 143. **Syn. n.**
Kleemannia plumosus . — Oudemans 1930: 138; Domrow 1974: 16; Hughes 1976: 338.
Zercoseius Macauleyi Hughes, 1948: 146. Synonymy by Evans (1954), and Hughes (1961).
Zercoseius plumosus . — Turk 1953: 12; Evans 1954: 798.
Zercoseius macauleyi . — Turk 1953: 12.
Lasioseius (Zercoseius) macauleyi . — Womersley 1956b: 116.
Kleemania (sic) plumosus. — Hughes 1961: 246.
Lasioseius macauleyi . — Westerboer and Bernhard 1963: 279.
Ameroseius dubiatus (sic). — Westerboer and Bernhard 1963: 534; Solomon 1969: 274.
Ameroseius plumosus . — Westerboer and Bernhard 1963: 491; Karg 1971a: 228; Bregetova 1977: 150; Karg 1993: 226; Narita et al. 2013b: 2317; Kazemi and Rajaei 2013: 66; Nemati et al. 2013: 20; Khalili-Moghadam and Saboori 2014: 674; Khalili-Moghadam and Saboori 2016: 544.
Kleemannia maculeyi (sic). — Bhattacharyya 1972: 133.
Kleemannia plumosa . — Ishikawa 1972: 97; Domrow 1979: 114.
Ameroseius dubitatus . — Castagnoli and Pegazzano 1985: 118.
Ameroseius (Kleemannia) plumosus . — Hajizadeh et al. 2013a: 150.

#### Type depository.

Of *Seiulus plumosus* – National Museum of Natural History, Naturalis Biodiversity Center, Leiden, Netherlands; of *Ameroseius dubitatus* – Istituto Sperimentale per la Zoologia Agraria, Firenze, Italy; of *Zercoseius macauleyi* – not stated (holotype not designated).

#### Type locality and habitat.

Of *Seiulus plumosus* – Netherlands, Sneek, on pond bat, *Myotis dasycneme* (as *Vespertilio dasycneme*) (Mammalia, Chiroptera); of *Ameroseius dubitatus* – Italy, Udine Region, Castions di Strada, in rotting leaves; of *Zercoseius macauleyi* – The United Kingdom (England, Scotland, Northern Ireland), in sifted oats and detritus from warehouse floors.

#### Comparative material.

Germany: 2 ♀♀ (ZMH: A19/63) – 11. 10. 1963, Kiel-Suchsdorf, Neubau, leg. F. Bolle, det. G. Rack in 1970; 1 ♀ (ZMB: 41031) – 1. 11. 1967, Tessin, Roggen H II, 2986, leg. G. Jahr; 22 ♀♀ (ZMH: A26/69) – 14. 7. 1969, Bomlitz, Einfamilienhaus-neubau, leg. H. Wasser, det. G. Rack in 1969; 3 ♀♀ (ZMH: A18/70) – Apr. 1970, Mettlach/Saar, Büroneubau, leg. W. Behrenz, det. G. Rack in 1970; 3 ♀♀ (ZMH: A70/71) – 26. 10. 1971, Bremen-Huchting, Neubau, Wohnung III, leg. and det. G. Rack in 1971; 6 ♀♀ (ZMH: A20/73) – Jan./Feb. 1973, Hannover, mehrstöckige Neubaublocks, leg. W. Rühm, det. G. Rack in 1973. Iran: 1 ♀ (SHKC) – 14. 5. 1991, Kerman Province, Bam County, Deh Bakri Region, under elytrae of *Oryctes nasicornis* (Coleoptera, Scarabaeidae), leg. N. Mehrzad. Italy: 1 ♀, 1 ♂ (ISZA: 12/36, syntypes) – Firenze, stalle; 1 ♀ (ISZA: 12/47, syntype) – Impruneta-Firenze, letamai; 1 ♀ (ISZA: 36/10, syntype) – Firenze, Cascine, legno castagno, bello; 1 ♀ (ISZA: 36/12, syntype) – Impruneta-Firenze, nel fiorume; 1 ♀ (ISZA: 27/21) – Castions di Strada, Udine, foglie marce (labelled *Ameroseius dubitatus*). Netherlands: 1 ♀ (RMNH: ACA.P.4303, syntype) – July 1896, Sneek, *Vespertilio dasycneme*, leg. Oudemans; 2 ♀♀ (ZMH: A24/72) – 19. 9. 1966, Hatert (Nijmegen), host: *Micromys minutus*, leg. and det. Lukoschus, Zool. Lab. Nijmegen. Norway: 3 ♀♀ (ISZA: 18/28) – Norway, leg. Thor (labelled *Ameroseius dubitatus*).

#### Published material from Slovakia.

Hronská Pahorkatina Wold: Mudroňovo Village (Krištofík et al. 1996, cited as *Ameroseius plumosus*). Malé Karpaty Mts.: Bratislava Capital, Devín Settlement, Devínska Lesostep Forest; Bratislava Capital, Devín Settlement, Fialková Dolina Valley (Kalúz 2005, cited as *Ameroseius plumosus*). Ondavská Vrchovina Highland: Ruská Poruba Village (Mrciak 1963, cited as *Kleemania plumosa*). Vihorlatské Vrchy Hills: Kaluža Village (Mrciak 1977, cited as *Kleemania plumosa*). Východoslovenská Pahorkatina Wold: Klokočov Village (Mrciak 1977, cited as *Kleemania plumosa*). Východoslovenská Rovina Flatland: Hnojné Village; Kristy Village; Oborín Village, Malčice Settlement; Tibava Village (Mrciak 1977, cited as *Kleemania plumosa*). Žitavská Pahorkatina Wold: Veľké Janíkovce Village (Sidor 1986, cited as *Kleemannia plumosus*).

#### Dubious published material from Slovakia.

Ondavská Vrchovina Highland: Ruská Poruba Village (Mrciak 1963, cited as *Ameroseius dubiatus*). [Notes: The original description of *A. dubitatus* is based only on a brief enumeration of unclear or vague diagnostic characters. Therefore it was not included in the identification keys of Westerboer and Bernhard (1963), Karg (1971a, 1993) or Bregetova (1977). According to Bregetova (1977), *A. dubitatus* seems to be a species very closely related with *Ameroseius elegans* Bernhard, 1963. Now we know that *A. dubitatus* is a synonym of *Kleemannia plumosus*. Slovak voucher material labelled *Ameroseius dubiatus* (sic) is not available for revision, so it is excluded from the list of valid published records.

#### New material from Slovakia.

Malé Karpaty Mts.: 2 ♀♀ – 9. 5. 2009, Bratislava Capital, zoological garden, dung-hill, manure, altitude 180 m, leg. P. Mašán.

#### Remarks.

**Plate 1.**
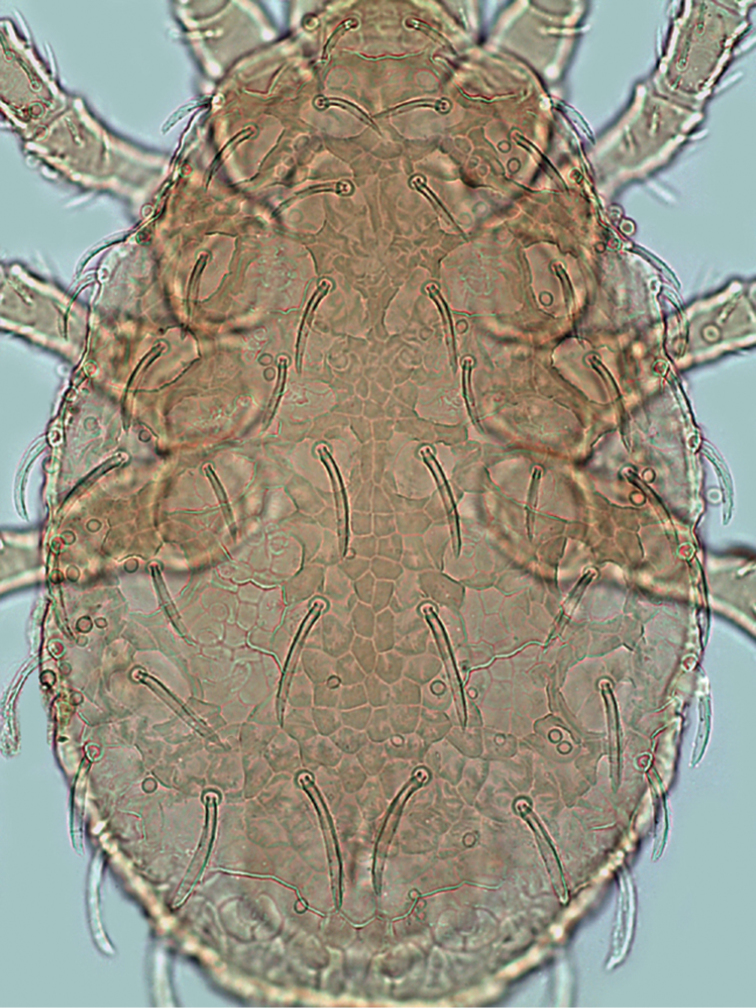
*Ameroseiella macrochelae*, female, dorsal idiosoma.

**Plate 2.**
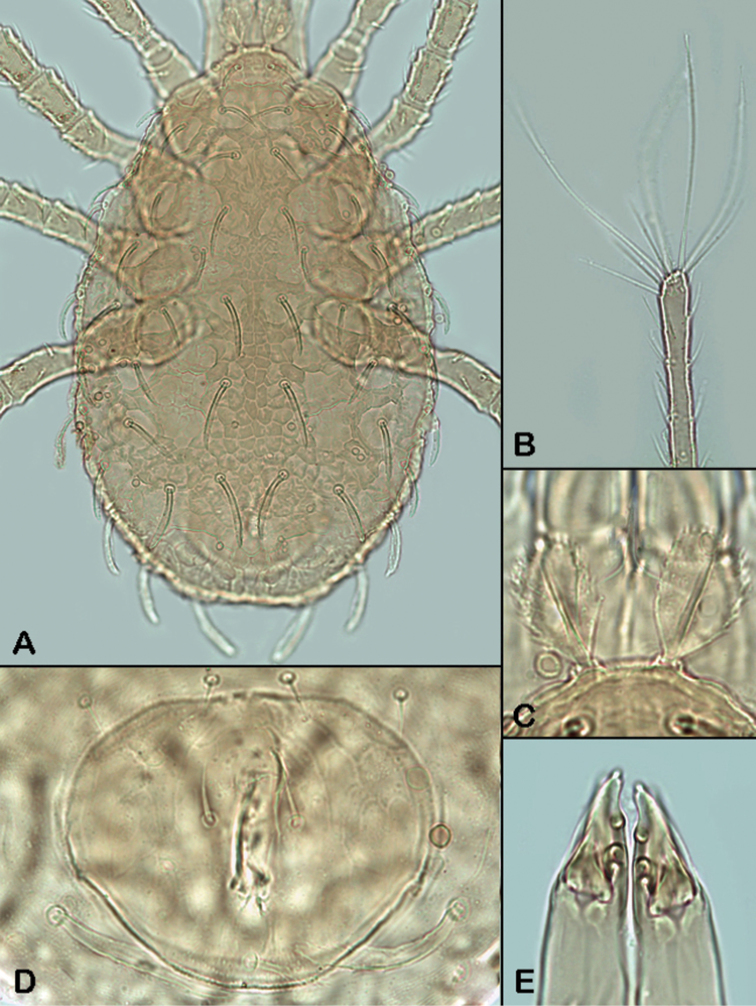
*Ameroseiella macrochelae*, female. **A** Dorsal idiosoma with proximal leg segments **B** Tarsus I, distal part **C** Setae j1 and epistome **D** Anal shield **E** Chelicerae, distal parts, dorsal view.

**Plate 24.**
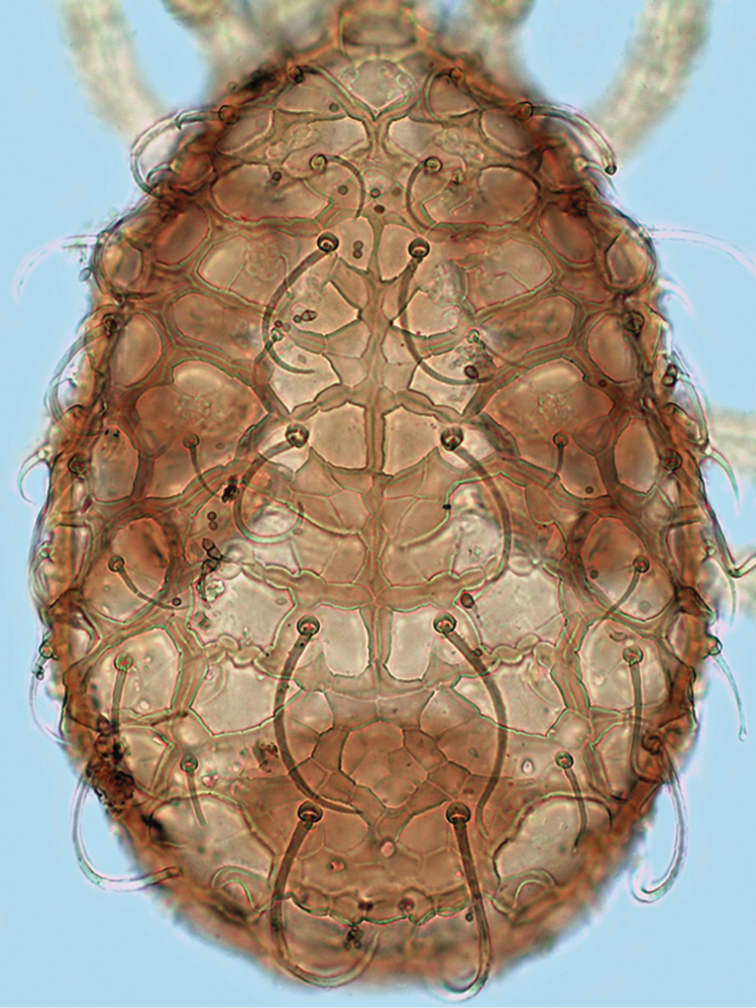
*Ameroseius renatae*, female, dorsal idiosoma.

**Plate 25.**
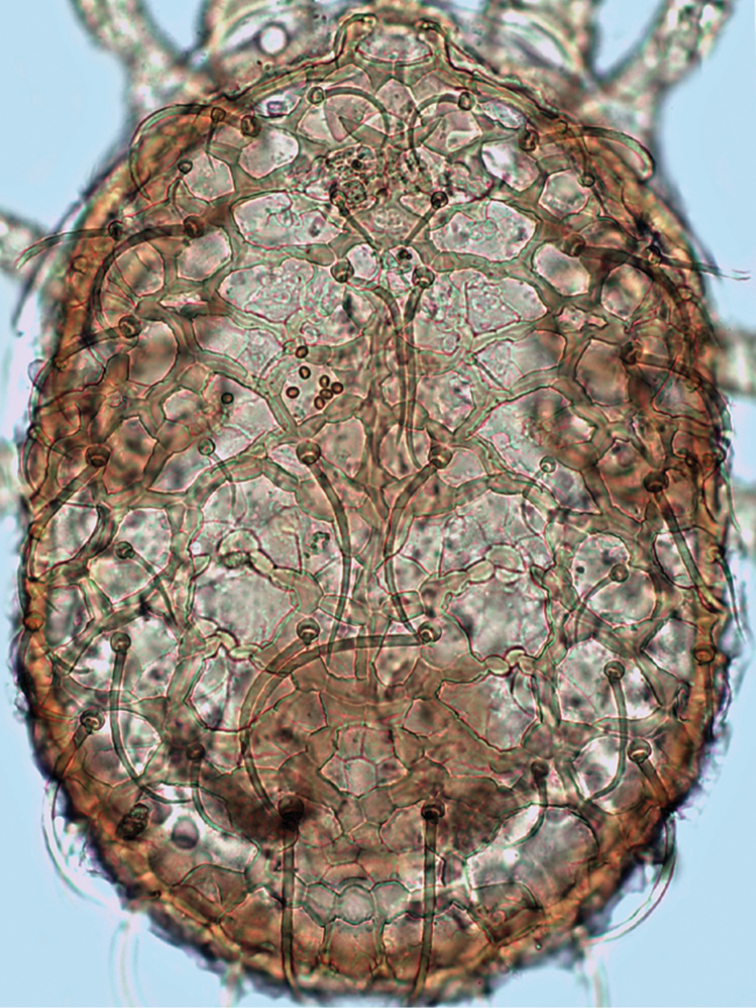
*Ameroseius renatae*, female, dorsal idiosoma (pressed specimen).

**Plate 35.**
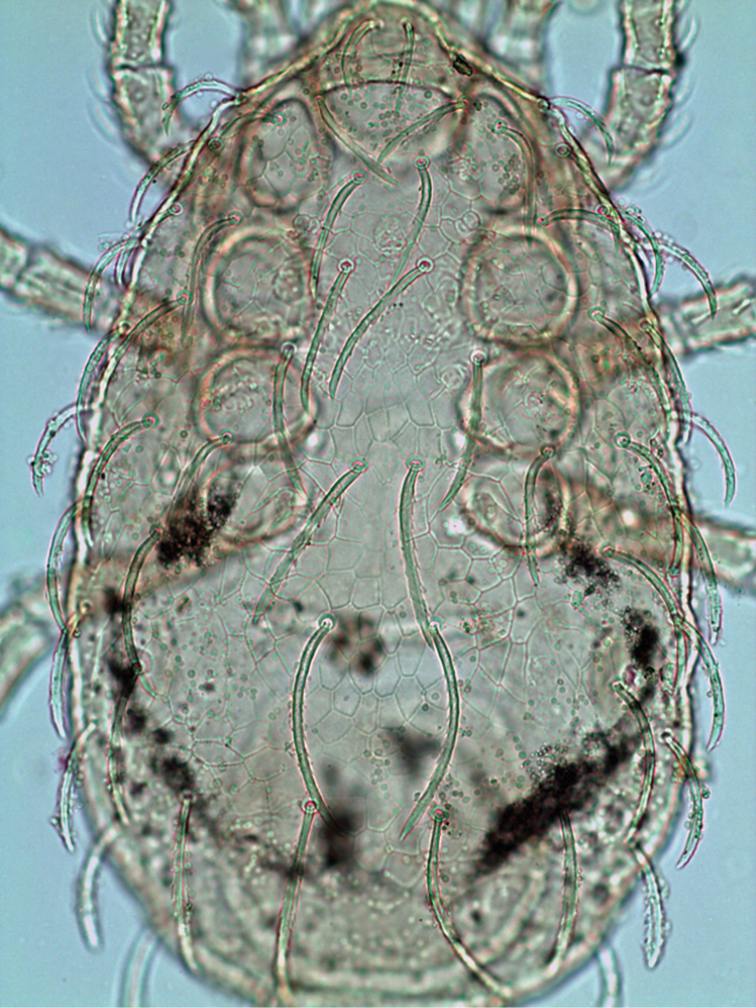
*Kleemannia delicata*, female, dorsal idiosoma.

**Plate 36.**
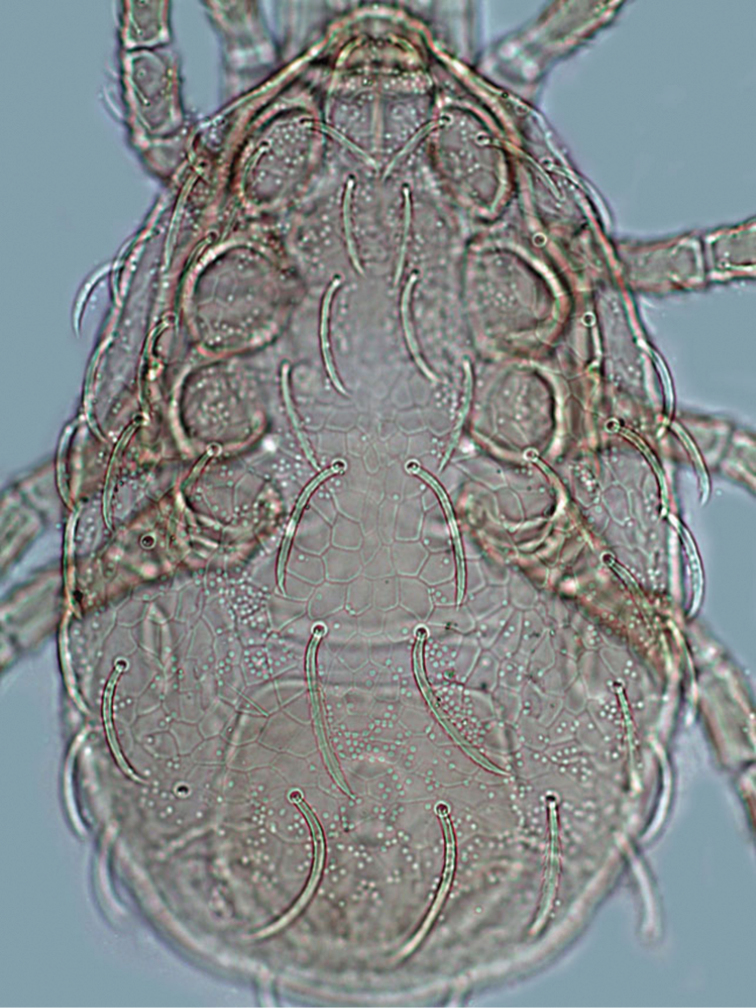
*Kleemannia delicata*, female (from Germany), dorsal idiosoma.

**Plate 37.**
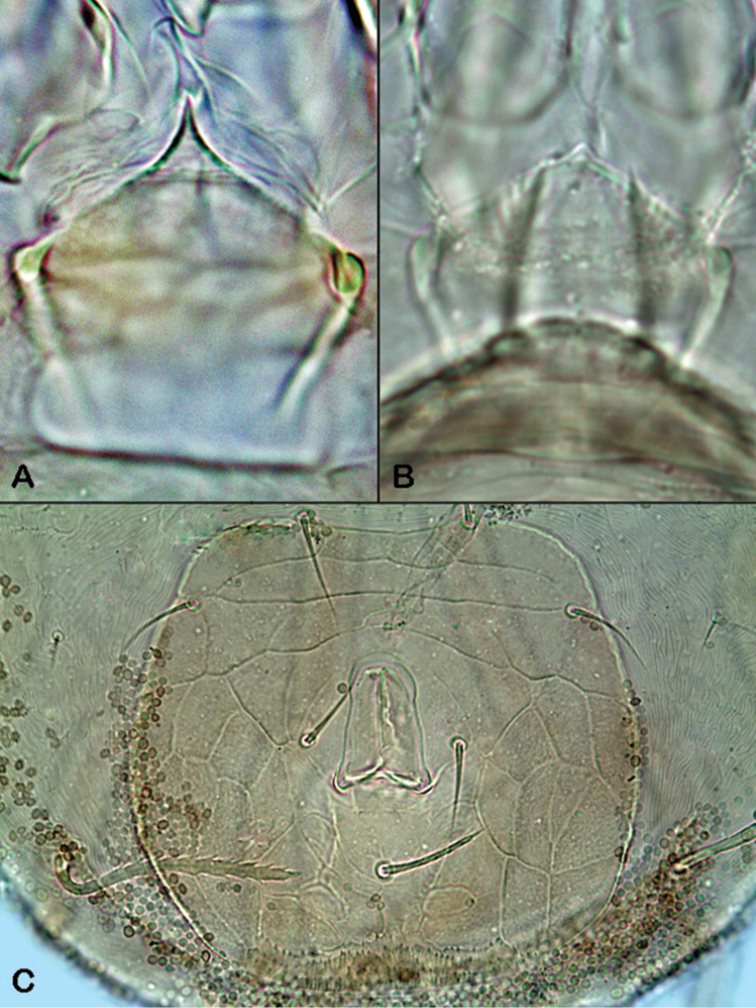
*Kleemannia* spp., female. **A** *Kleemannia delicata*, epistome **B** *Kleemannia pavida*, epistome **C** *Kleemannia delicata*, ventrianal shield.

**Plate 46.**
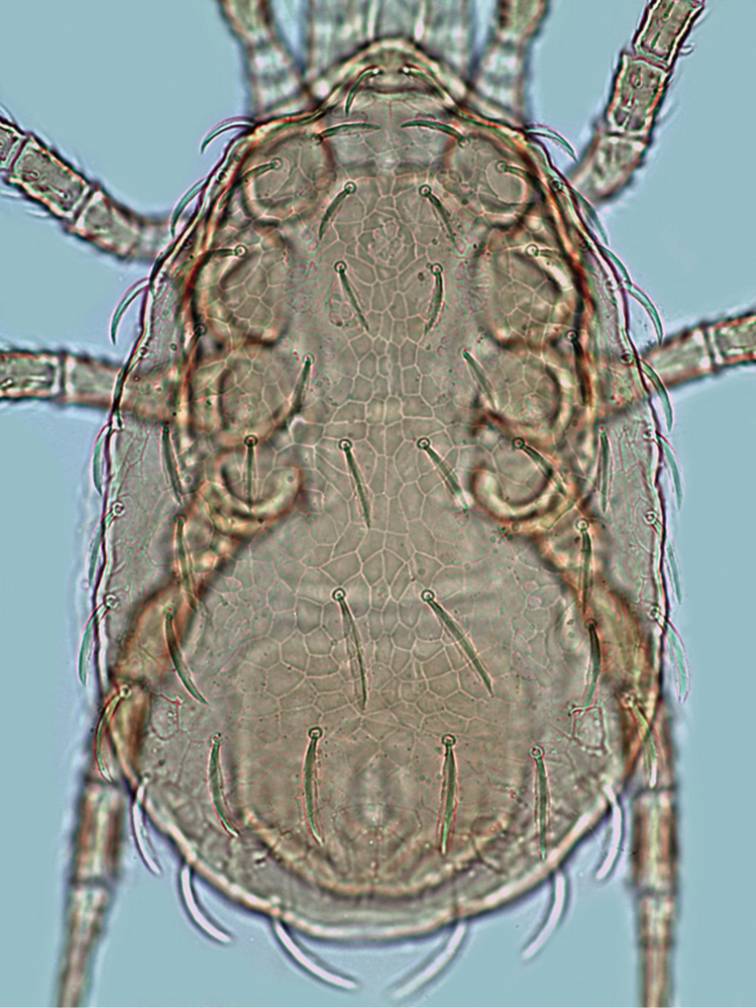
*Kleemannia pavida*, female (from Germany), dorsal idiosoma.

**Plate 47.**
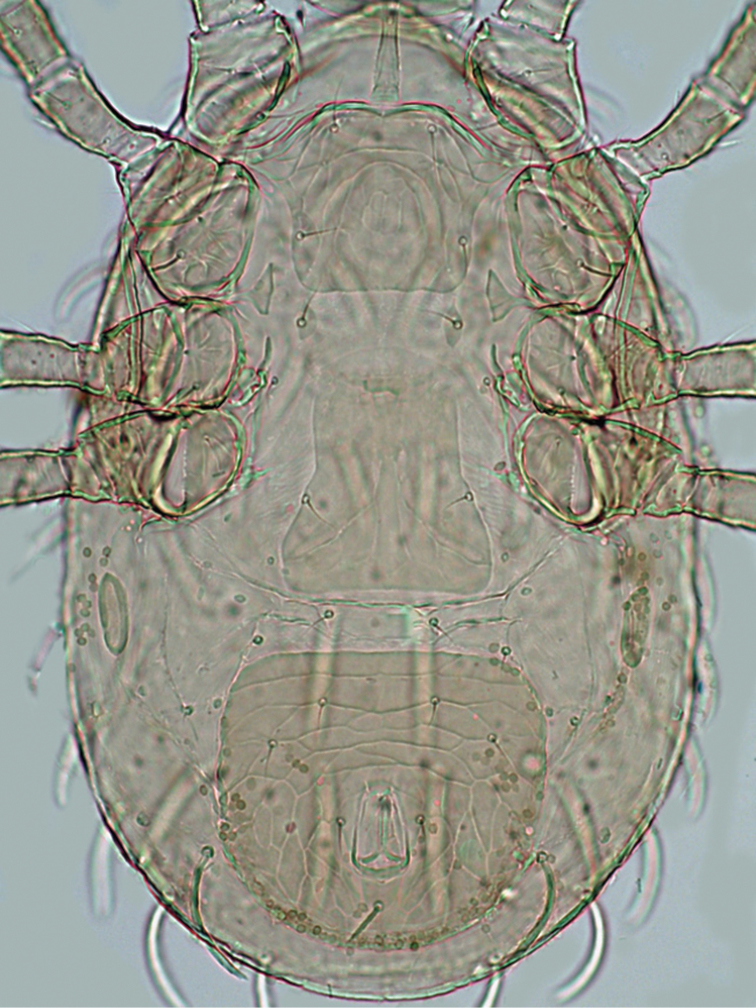
*Kleemannia pavida*, female (from Germany), ventral idiosoma.

**Plate 50.**
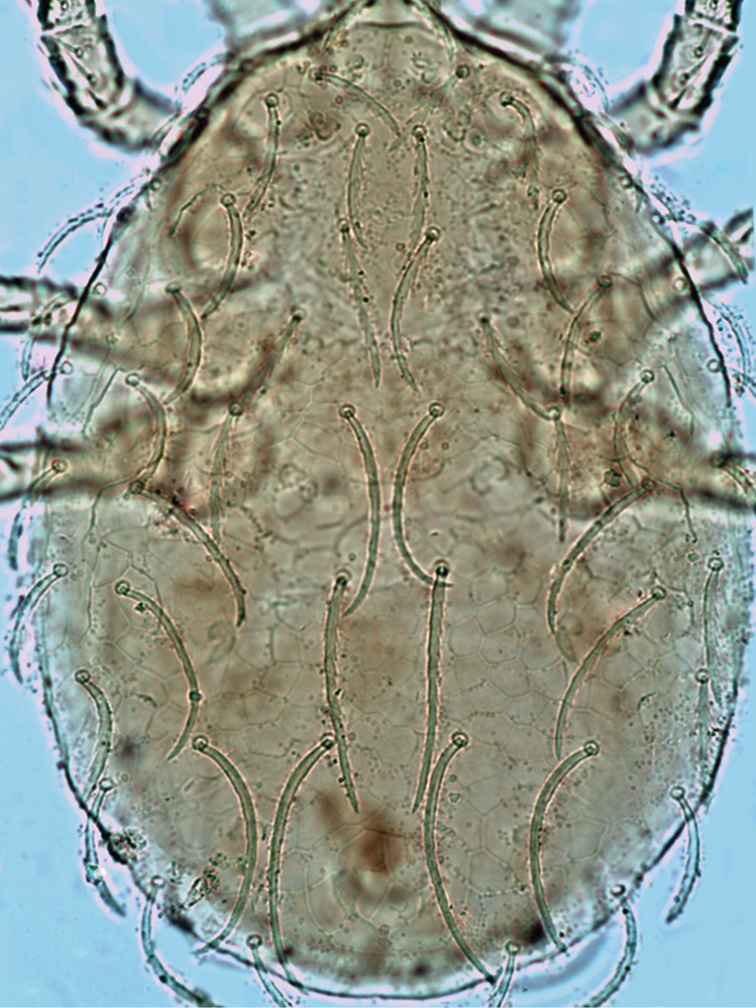
*Kleemannia plumigera*, female, dorsal idiosoma.

**Plate 51.**
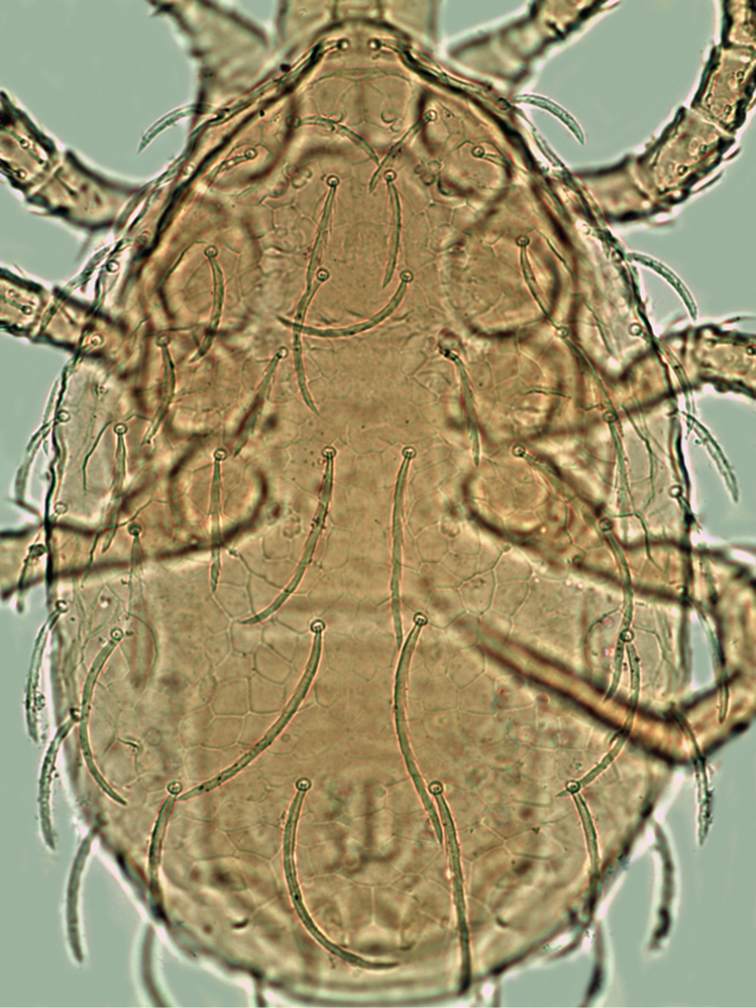
*Kleemannia plumigera*, female (from Germany), dorsal idiosoma.

**Plate 52.**
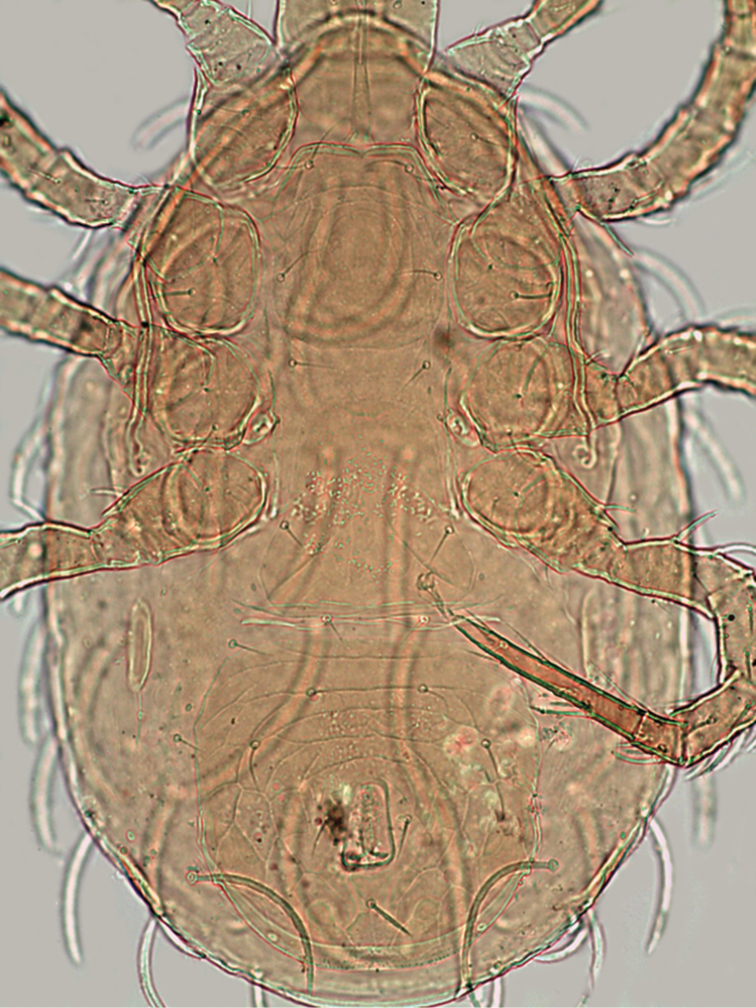
*Kleemannia plumigera*, female (from Germany), ventral idiosoma.

**Plate 53.**
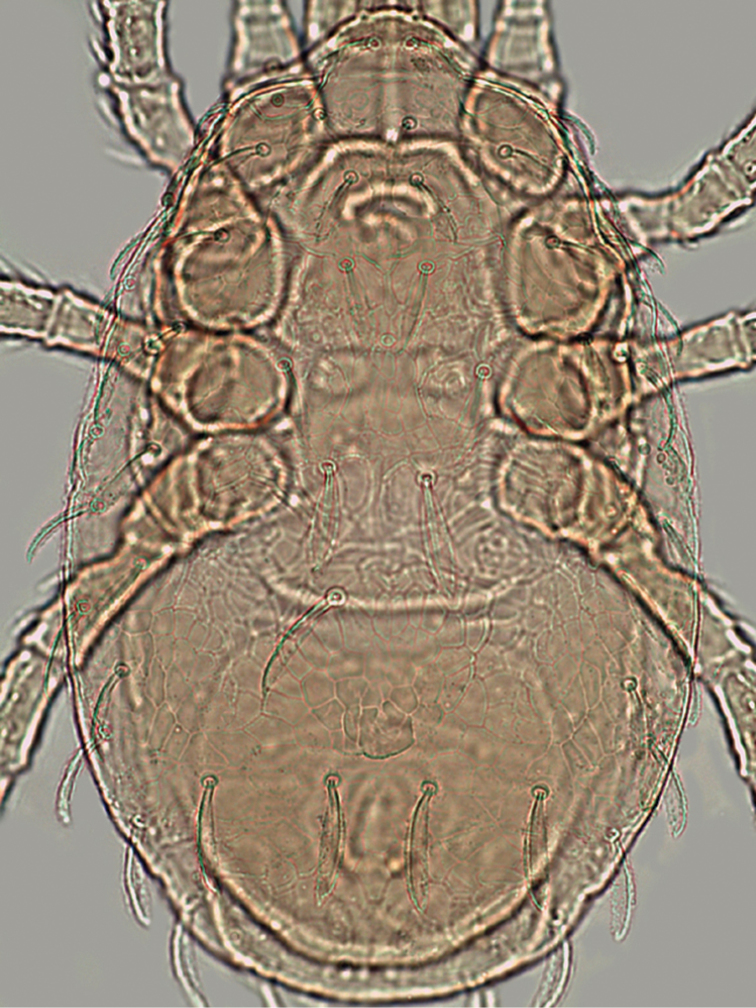
*Kleemannia plumosa*, female, dorsal idiosoma.

**Plate 54.**
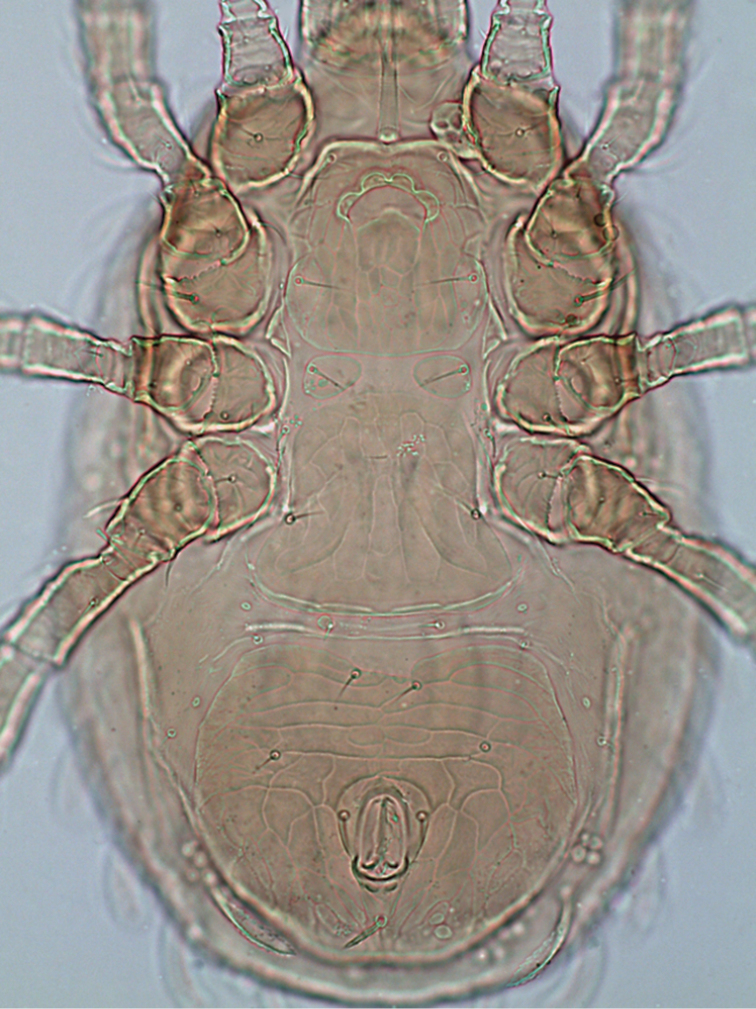
*Kleemannia plumosa*, female, ventral idiosoma.

**Plate 55.**
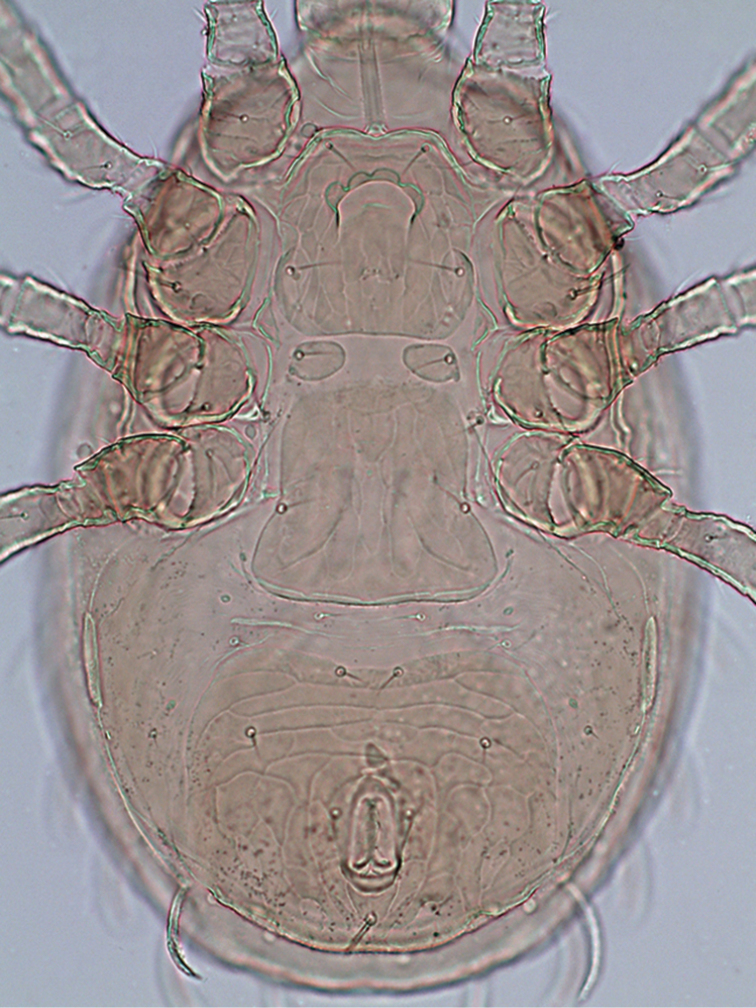
*Kleemannia plumosa*, female, ventral idiosoma.

**Plate 56.**
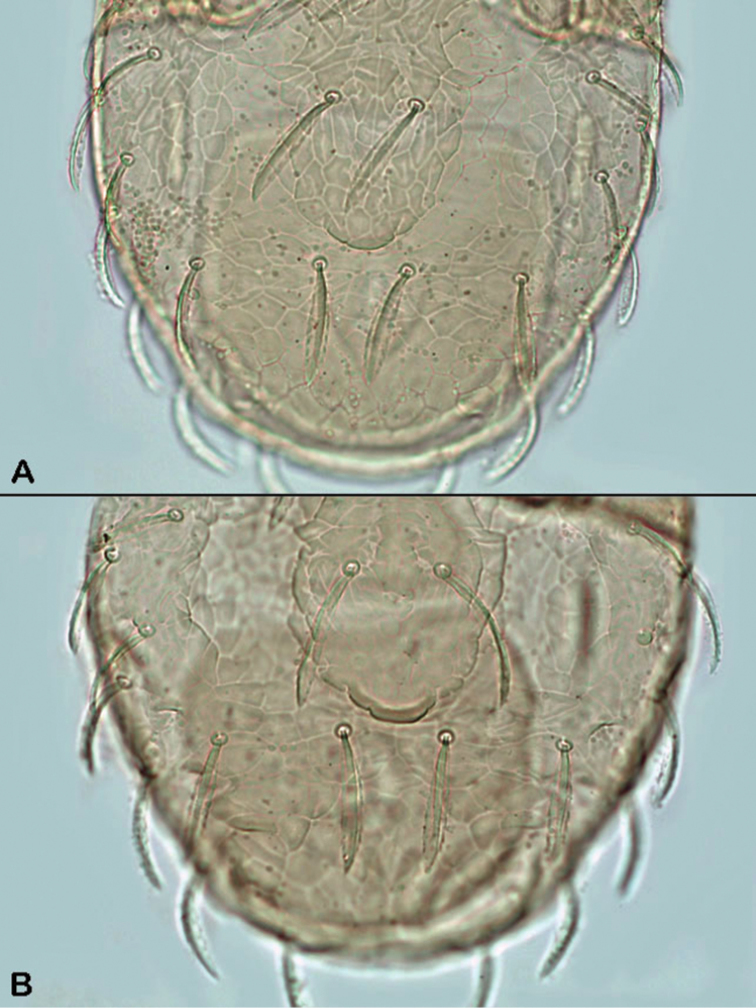
*Kleemannia plumosa*, female, posterior part of dorsal shield. **A** A form with shorter dorsal setae **B** A form with longer dorsal setae.

When Oudemans (1902) described this species, as a member of *Seiulus*, he inadvertently included three different congeneric species to the type specimen series (*pavida* as a protonymph, *plumosa* as a deutonymph, and *plumea* as a female), but did not designate the holotype. Oudemans tried to correct his mistake in 1930, referring the name *plumosus* to a female specimens collected from *Vespertilio dasycneme* (Chiroptera), originally described as a deutonymph and illustrated in figures 18–20 (on plates 1 and 2) of Oudemans (1902). In the re-description provided in that paper, he was ambiguous in relation to the number of dorsal shield setae, stating 28 as well as 29 setal pairs (Oudemans 1930). The specimen shown in figure 19 of that publication apparently has z6, differing from the single type female of *Kleemannia plumosa* available in the Oudemans Collection in Leiden (numbered 4303); thus, the identity of that illustrated specimen is uncertain. Another slide in Oudemans Collection, numbered 4302, and labelled „*Kleemannia plumosus*, ♀ dors., Arnhem, 1919, Oudemans“ bears only part of a sarcoptifom mite, with gnathosoma and the anteriormost part of idiosoma.

*Kleemannia plumosa* is easily recognisable by the specific inverted U-shaped sculptural structure on anterior surface of sternal shield having its anterior margin scalloped and heavily sclerotised, but it has been referred to a variety of names, including some synonyms and misidentifications. A new synonymy is here proposed for *K. plumosa* and *Ameroseius dubitatus*, based on direct examination of their type specimens. There is a relatively large series of slides in the Berlese Collection in Firenze, but none of them bears a type designation. Only specimens from Firenze may belong to the original type material of Berlese. Castagnoli and Pegazzano (1985) considered the female on slide No. 27/21 (from Castions di Strada, Udine Region) to be the holotype specimen but I could not validate their statement here.

### Kleemannia pseudoplumosa

Taxon classificationAnimaliaMesostigmataAmeroseiidae

(Rack, 1972) comb. n.

**Plate 57.**
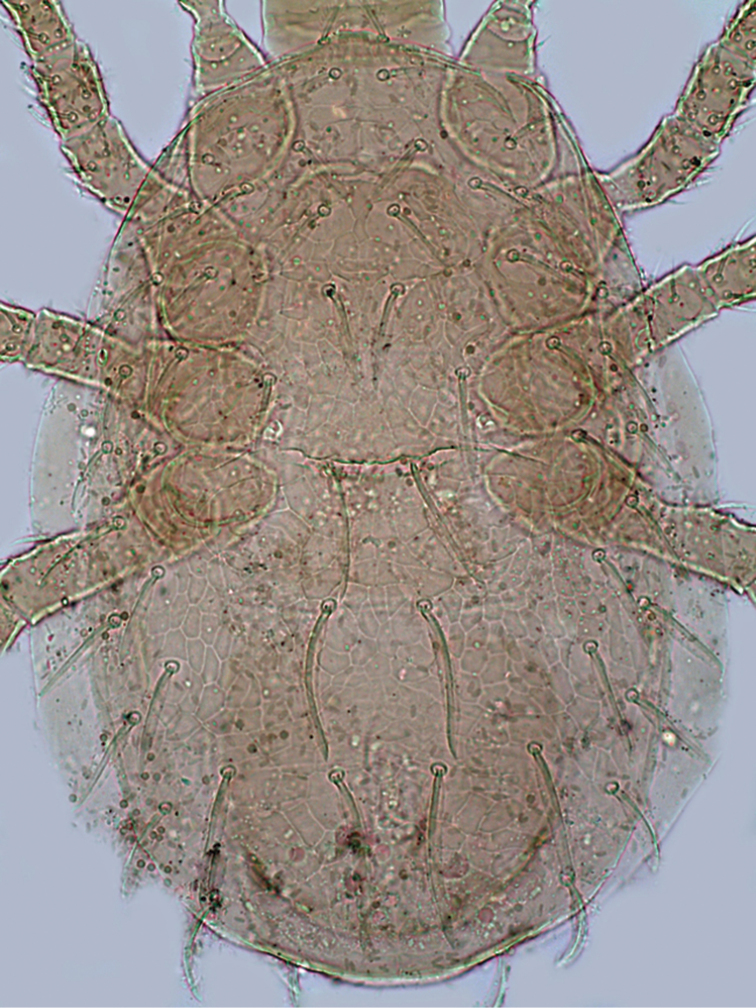
*Kleemannia pseudoplumosa*, female, dorsal idiosoma (pressed specimen).

**Plate 58.**
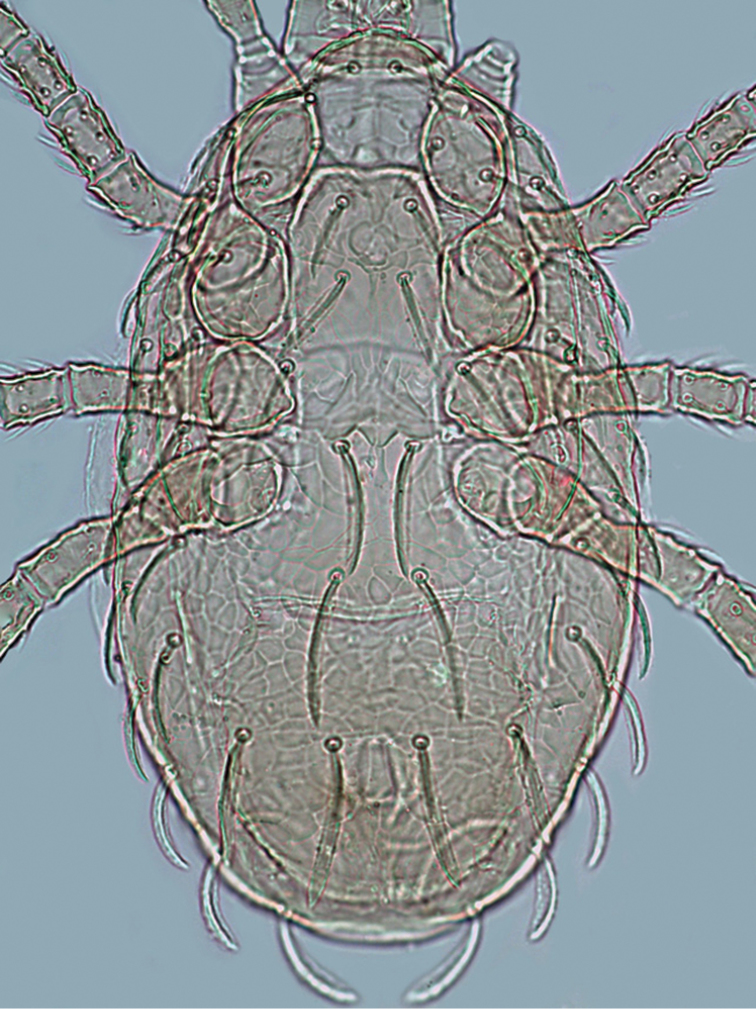
*Kleemannia pseudoplumosa*, female (paratype, from Germany), dorsal idiosoma.

**Plate 59.**
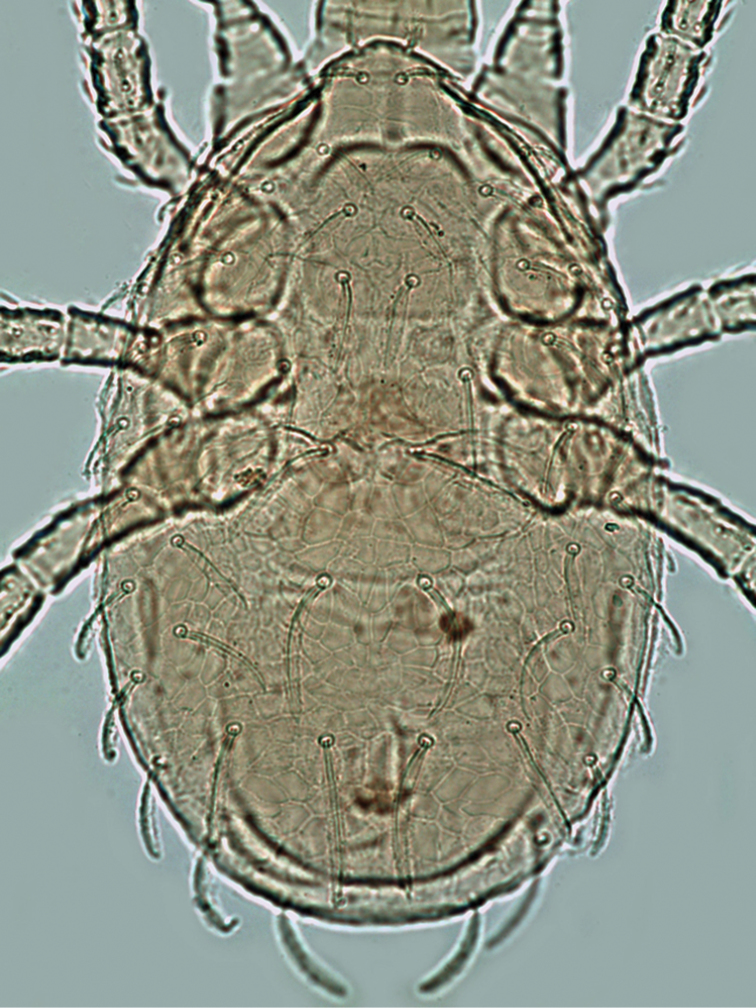
*Kleemannia pseudoplumosa*, female (from Greece), dorsal idiosoma (pressed specimen).

**Plate 60.**
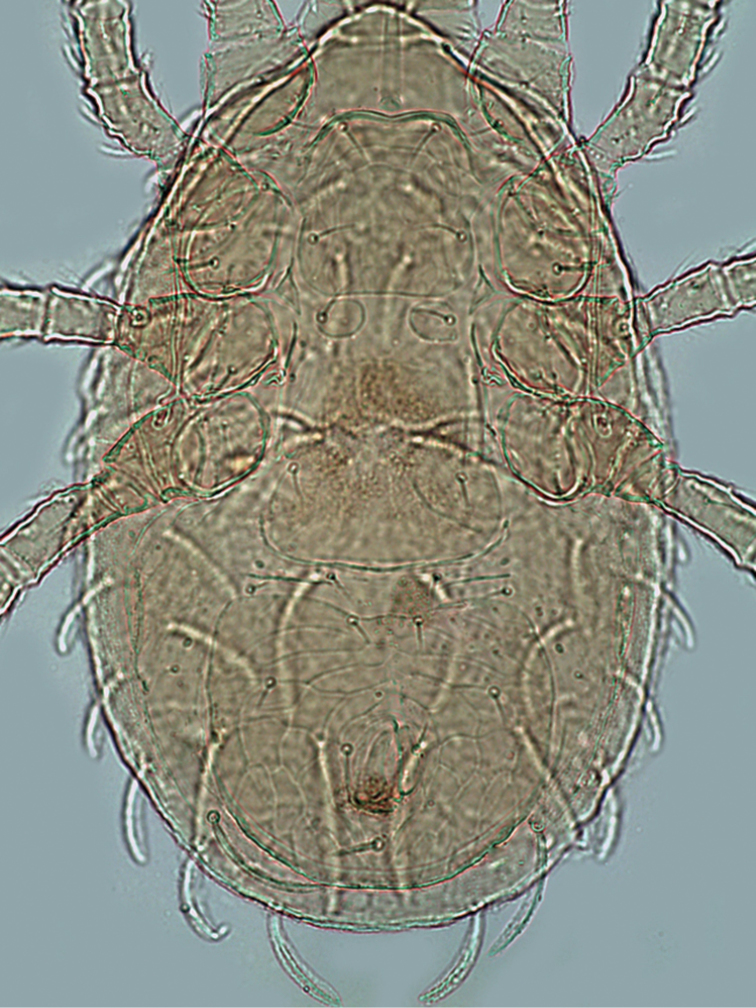
*Kleemannia pseudoplumosa*, female (from Greece), ventral idiosoma (pressed specimen).

Plates 57 , 58 , 59 , 60

Primoseius macauleyi . — Womersley 1956b: 116. **Misidentification.**
Ameroseius pseudoplumosus Rack, 1972: 249.
Ameroseius eumorphus Bregetova, 1977: 153. **Syn. n.**
Kleemannia potchefstroomensis Kruger & Loots, 1980: 1. **Syn. n.**
Kleemannia plumosus . — Zaher 1986: 404. **Misidentification.**
Ameroseius (Kleemannia) eumorphus . — Hajizadeh et al. 2013a: 151.
Ameroseius eumorphus . — Narita et al. 2013b: 2325; Kazemi and Rajaei 2013: 65; Nemati et al. 2013: 19; Khalili-Moghadam and Saboori 2016: 538.
Ameroseius potchefstroomensis . — Narita et al. 2013b: 2325; Khalili-Moghadam and Saboori 2016: 544.
Ameroseius pseudoplumosus . — Kazemi and Rajaei 2013: 66; Nemati et al. 2013: 20; Khalili-Moghadam and Saboori 2016: 544.

#### Type depository.

Of *Ameroseius pseudoplumosus* – Zoologischen Institut und Zoologischen Museum der Universität Hamburg, Germany; of *Ameroseius eumorphus* – Zoological Institute, Russian Academy of Sciences, Saint Petersburg, Russia; of *Kleemannia potchefstroomensis* – Institute for Zoological Research, Potchefstroom University, Potchefstroom, South Africa.

#### Type locality and habitat.

Of *Ameroseius pseudoplumosus* – Germany, Bischofsheim (at Frankfurt am Main), in new building; of *Ameroseius eumorphus* – Russia, Barents Sea, Kharlov Island (Murmansk Region), on rocks under plants (paratypes: Azerbaijan and Uzbekistan); of *Kleemannia potchefstroomensis* – South Africa, Potchefstroom, in compost.

#### Comparative material.

Australia: 2 ♀♀ (SAMA: ARA73/N1951689, ARA73/N1951690) – Jan. 1954, Port Adelaide, in bark scrapings of logs, det. H. Womersley (labelled *Primoseius macauleyi*). Belgium: 1 ♂ (No. 170354) – 6. 10. 1967, Anvers, zoological garden, litière de cobaye (*Cavia porcellus*), leg. P. Elsen (labelled Ameroseiidae). Germany: 1 ♀ (BMNH) – 1957, Langenhorn, Müllplatz, Eing. Nr. A2, 62, leg. H. J. Haas (labelled *Kleemannia plumosus*); 15 ♀♀ (BMNH) – 9. 12. 1963, Flensburg, Neues Rathaus, leg. and det. G. Rack, Eing. Nr. A5/64 (labelled *Kleemannia plumigera*); 4 ♀♀ (ZMH: A53/71, holotype and paratypes) – 4. 11. 1971, Bischofsheim b. Frankfurt a. M., Neubau, leg. F. Lönholdt. Greece: 2 ♀♀ (IZSAV) – 16. 8. 2005, Chalkidiki Peninsula, Sarti Village, bank of a small river under plane trees, soil detritus with leaf fall, leg. P. Mašán. Iran: 1 ♀ (SHKC) – 20. 11. 2001, Tehran Province, Tehran Capital, Lale Park, soil detritus, leg. and det. Sh. Kazemi (labelled *Ameroseius eumorphus*). Israel: 2 ♀♀ (IZSAV) – 27. 11. 1966, Sha‘ar Hagolan, banana plantation, litter, leg. and det. M. Costa (labelled *Kleemania plumosus*); 3 ♀♀ (BMNH) – 5. 10. 1962, Mishmar Haemek, manure heap, ex. 1968.1.26:7, leg. and det. M. Costa [labelled and published as *Kleemania plumosus* by Costa (1966)]. Japan: 1 ♀ (BMNH) – 5. 8. 1959, Japan: at Hawaii, *Arctotis stoechadifolia*, Lot. 60/2461 (4 flowers), leg. H. Woolford (labelled *Kleemania*). Sweden: 1 ♀ (ZMH: A1. 1972.4702) – 28. 9. 1959, Schweden, Västerås, von Birnenblät., M. Sellnick Collection (labelled *Kleemannia pavidus*). United Kingdom: 1 ♀ (BMNH) – 1905, 24. 6. 1925, Lincolnshire, C. F. George Collection, 596 (labelled Acarina, Gamasea, *Sejus*); 1 ♀ (BMNH: E 2001-124) – F. A. Turk Collection, re-det. in Oct. 1973 by R.M.P. (labelled *Kleemania plumosus*).

#### New material from Slovakia.

Oravská Magura Mts.: 2 ♀♀ – 15. 10. 1994, Zázrivá Village, Minčol Forest (labelled as *Ameroseius eumorphus* – leg. et det. S. Kalúz, unpublished but registered by the ‘Databank of Slovak Fauna’ at former http://zoology.fns.uniba.sk/dfs/system300.htm). Podunajská Rovina Flatland: 1 ♀ – 30. 11. 1994, Dobrohošť Village, Dunajské Kriviny Forest (labelled as "ameroseiid" – leg. S. Kalúz, possibly registered by the ‘Databank of Slovak Fauna’ under the name *Ameroseius pseudoplumosus*).

#### Remarks.

It appears likely that the names *plumosa* and *macauleyi* have been widely used for *Kleemannia pseudoplumosa* in the literature about mites associated with synanthropic habitats throughout the world. Based on my examination, the Australian misidentified specimens of Womersley (1956b), originally named *macauleyi* and classified in the newly erected genus *Primoseius*, are in excellent agreement with the type specimens of *K. pseudoplumosa*, collected in new buildings in Germany, and understood here to be conspecific with the specimens from Australia. In the illustration of the specimens identified as *Primoseius macauleyi*, Womersley (1956b) did not comment on the absence of the semi-circular scalloped sculptural pattern on the sternal shield (typical character of the species). Together with Halliday (1997) who revised the Australian fauna of Ameroseiidae, he did not consider this absence to indicate the existence of a separate species.

Further literature records that are consistent with the morphological features of the specimens studied by Womersley (1956b) and Rack (1972) include those from Israel (Costa 1966), Murmansk Region in Russia, Azerbaijan and Uzbekistan (Bregetova 1977), South Africa (Kruger and Loots 1980), Egypt (Zaher 1986), and Iran (Khalili-Moghadam and Saboori 2016). These records are supported by specimens examined here from Slovakia, Greece and Israel, and some photographs of the holotype of *Ameroseius eumorphus* (made by M. Stanyukovich, Russian Academy of Sciences, St. Petersburg, Russia, and obtained from A. Khalili-Moghadam, Iran). Accordingly, *A. eumorphus* Bregetova, 1977 and *Kleemannia potchefstroomensis* Kruger & Loots, 1980 are placed in synonymy with *Kleemannia pseudoplumosa* (Rack 1972).

The description of *Kleemannia pseudoplumosa* by Rack (1972) was apparently overlooked by the followers. Bregetova (1977), Kruger and Loots (1980), Ibrahim and Abdel-Samed (1992) and Karg (1993) have not cited this species in their identification keys and/or differential diagnoses. The same can be stated for the descriptive paper of Narita et al. (2013b), on a new species *Kleemannia mineiro* from Brazil that is closely related with *K. pseudoplumosa*. In their identification key elaborated for a comparative purpose, Narita et al. (2013b) included *mineiro* close to *eumorphus* and *potchefstroomensis*, relying on the detailing the discrepancies in the original drawings. They stated that *eumorphus* differs from *potchefstroomensis* only in the sculpture of the sternal shield. This shield being unevenly ornamented, and with central portion smooth in *eumorphus* (in *potchefstroomensis*, whole surface with even but weak sculptural reticula). This difference is well within the range of variability found in European specimens of *K. pseudoplumosa*, and I regard it to be untenable. Moreover, there are some contradictory data on chaetotaxy of some leg segments as given by Kruger and Loots (1980) for *potchefstroomensis*, Narita et al. (2013b) for *mineiro*, and Khalili-Moghadam and Saboori (2016) for *eumorphus*. I consider them to be controversial, and they should be revised more carefully in the future.

### Kleemannia reticulata

Taxon classificationAnimaliaMesostigmataAmeroseiidae

Kruger & Loots, 1980

Kleemannia reticulatus Kruger & Loots, 1980: 4.
Ameroseius reticulatus . — Narita et al. 2013b: 2324.

#### Type depository.

Institute for Zoological Research, Potchefstroom University, Potchefstroom, South Africa.

#### Type locality and habitat.

Angola, Kassai Province, Luangue, Tchitenga River, in forest soil.

### Kleemannia tenella

Taxon classificationAnimaliaMesostigmataAmeroseiidae

(Berlese, 1916) comb. n.

Plates 61 , 62 , 63

Ameroseius tenellus Berlese, 1916a: 47.
Kleemannia sp. — Piryanik 1958: 130.
Ameroseius tenellus . — Castagnoli and Pegazzano 1985: 410.
Ameroseius lanatus Solomon, 1969: 274. **Syn. n.**
Ameroseius lanatus . — Bregetova 1977: 158.
Ameroseius fimetorum Karg, 1971a: 230. **Syn. n.**
Ameroseius fimetorum . — Bregetova 1977: 160; Karg 1993: 228; Narita et al. 2015: 395.

#### Type depository.

Of *Ameroseius tenellus* – Istituto Sperimentale per la Zoologia Agraria, Firenze, Italy; of *Ameroseius lanatus* – not stated (holotype not designated); of *Ameroseius fimetorum* – Museum für Naturkunde, Berlin, Germany.

#### Type locality and habitat.

Of *Ameroseius tenellus* – Italy, Firenze, in moss; of *Ameroseius lanatus* – Romania, holotype not designated [three females: Luncaviţa at Tulcea, Mogoşoaia at Bucureşti, Podul Iloaie at Iaşi, on small rodents, *Apodemus sylvaticus* and *Microtus arvalis* (Mammalia)]; of *Ameroseius fimetorum* – Germany, Frankfurt/Oder (Oderbruch), Manschnow (holticulture), in compost soil of greenhouse.

#### Comparative material.

Germany: 1 ♀ (ZMB: 40156, holotype) – Febr. 1963, Manschnow/Oderbruch, Gewächshaus, 2985 (labelled *Ameroseius fimetorum*). Italy: 2 ♀♀ (ISZA: 171/12, holotype; ISZA: 171/13, paratype) – Firenze, musco; 1 ♀ (ISZA: 21/8, paratype) – Tiarno, Trentino, fiorume; 2 ♀♀ (ISZA: 168/50, paratypes) – Tiarno, Trentino, nel fieno; 1 ♀ (ISZA: 171/14) – Palermo, musco.

#### New material from Slovakia.

Podunajská Rovina Flatland: 6 ♀♀ – 3. 5. 2006, Bratislava Capital, air-port, meadow, burrow entry of *Spermophilus citellus* (Mammalia), rotting leaves and vegetation rests, altitude 150 m, leg. P. Mašán.

#### Remarks.

This species was only briefly and inadequately described by Berlese (1916a). There are seven slides of this species in the Berlese Acaroteca (21/8, 168/50, 171/12–14, 219/48–49), from moss, hay and litter from Italy (Firenze, Tiarno – Trentino, Palermo). Only one slide bearing a female from Firenze is labelled „tipico” (171/12). I have examined six females mounted onto five slides of the collection (slides 219/48–49 were not available for my study). Despite such a sufficient series of slides, only two of them contains at least partially examinable specimens in which some limited observation of features is possible. Some characters on the dorsum can be examined in the holotype specimen, and some ventral characters in a paratype specimen on slide 168/50. Other examined specimens are in poor condition and barely suitable for thorough observation. As a critical examination of the type specimens has revealed that *Kleemannia tenella* and *Kleemannia fimetorum* (Karg 1971) are identical, the latter must be placed in synonymy with the former. This comparison was extended to specimens of *K. tenella* collected in Slovakia and the agreement was found to be complete. In the original illustrations of *K. fimetorum*, Karg (1971a) asymmetrically depicted the setal complement of 29 pairs of setae on the dorsal shield (30 setae on left portion, and 27 setae on right portion), and mistakenly concluded that only three instead of four teeth were present on proximal masticatory area of the fixed cheliceral digit. On the basis of my examinations of description and illustrations, as the type material of *Ameroseius lanatus* Solomon, 1969 is probably no longer available (Adina Cǎlugǎr made a notable effort to find the type specimens), this species is also placed in synonymy with *K. tenella*. I am not able to detect any consistent morphological differences in the material from Italy, Romania, Germany and Slovakia, and thus all are considered to belong to a single species.

*Kleemannia tenella* and the closely related Chinese species *Kleemannia guyimingi* (Ma, 1997) and *Kleemannia longisetosa* (Ye & Ma, 1993) differ so markedly from the other members of this genus in the length of the dorsal shield setae (J-setae lengthened, Z-setae shortened), and in the arrangement of the ventral shields (pseudo-metasternal platelets absent; setae st3 on soft integument; ventrianal shield reduced and rounded, concave anteriorly, with two pairs of setae very close to its anterior margin), that they might be considered as belonging to a separate species group.

### Kleemannia wahabi

Taxon classificationAnimaliaMesostigmataAmeroseiidae

Ibrahim & Abdel-Samed, 1992

Kleemania (sic) wahabi Ibrahim & Abdel-Samed, 1992: 137.
Ameroseius wahabi . — Narita et al. 2013b: 2325; Khalili-Moghadam and Saboori 2016: 544.

#### Type depository.

Fruit Acarology Division, Plant Protection Research Institute, Agricultural Research Center, Dokki, Egypt.

#### Type locality and habitat.

Egypt, Alexandria Governorate, Sidy Krier District, from debris under fig trees, *Ficus carica* (Moraceae).

#### Remarks.

I suspect this species to be a junior synonym of *Kleemannia pseudoplumosa*.

### Genus Neocypholaelaps

Taxon classificationAnimaliaMesostigmataAmeroseiidae

Vitzthum, 1942

Cypholaelaps Berlese, 1918: 117 (preocc. by Cypholaelaps Berlese, 1916b: 166).
Neocypholaelaps Vitzthum, 1942: 763. Replacement name for Cypholaelaps Berlese, 1918: 117. Type species: Laelaps ampullula Berlese, 1910, by original designation.
Indoseius Evans, 1955b: 107. Type species: Indoseius stridulans Evans, 1955, by original designation. Synonymy by Evans (1963a). Not Indoseius Ghai & Menon, 1969 (= Indoseiulus Ehara, 1982, replacement name for Indoseius sensu Ghai & Menon, 1969).
Neocypholaelaps . — Evans 1963a: 210; Karg 1993: 220; Moraes and Narita 2010: 38.

#### Diagnosis (adults).

Dorsal shield variously sclerotised and ornamented, normally with 29 pairs of setae (28 pairs in *Neocypholaelaps xylocopae*, and 30 pairs in *Neocypholaelaps novaehollandiae*). Dorsal shield setae simple or variously modified, needle-like (smooth, pilose or serrate) to lanceolate or clavate (densely plumose), and sexually dimorphic: females with similar setae; males with at least one seta conspicuously enlarged, or with a mixture of shortened, thickened and lengthened setae). In female, st1 and st2 on sternal shield, st3 on soft integument or small suboval pseudo-metasternal platelets and st4 on soft integument; sternal and epigynal shields usually smooth and unornamented on surface; genital poroids outside the epigynal shield. Female with anal shield bearing three circum-anal setae, male with slightly expanded anal shield or ventrianal shield bearing 1–3 pairs of opisthogastric setae (often JV2 and/or JV3, rarely also ZV2). Peritrematal shields or peritremes with anterior ends connected to dorsal shield. Opisthogastric soft integument with six pairs of setae in female (five pairs in *Neocypholaelaps wilsoni*). Soft striate integument usually densely incrusted with sclerotic denticles or tubercles. Corniculi slender and convergent, surrounded by hyaline membranes, with undivided and pointed apex. In female, fixed digit of chelicera usually edentate on proximal masticatory area, with at most one weak subdistal tooth, bearing hyaline lobed appendage; movable digit edentate, sometimes with subapical denticle, well hooked distally, and provided with spermatodactyl in male. Epistome with rounded and denticulate anterior margin. Palptarsal apotele two-tined. Genu III and tibiae III–IV with two anterolateral and one posterolateral setae. Tarsi I–IV each with well developed empodium and claws. Insemination apparatus with spermathecal ducts fused or separate.

#### Remarks.

This genus now comprises 22 species based from tropical and subtropical areas of Africa (eight species), Asia (ten species), Australia and Oceania (three species) and South America (one species). *Neocypholaelaps favus* and *Neocypholaelaps apicola* seem to be the only two species distributed in temperate zone of the Palaearctic region (see remark under *N. favus*), whereas *Neocypholaelaps novaehollandiae* was originally reported from temperate region of the southern hemisphere, New Zealand. *Neocypholaelaps* species belong to the nectar- and pollen-feeders associated with various flowers and flower-visiting animals (Evans 1963a, Klimov et al. 2016), namely apid bees (Hymenoptera) and butterflies (Lepidoptera). *Neocypholaelaps wilsoni* is reported from nasal cavities of a psittacid bird in Papua New Guinea (Allred 1970).

*Neocypholaelaps favus*, originally known from Japan (Ishikawa 1968), was firstly reported from Europe by Haragsim et al. (1978), based on findings in apiaries in the Czech Republic. There are several other reports of the species of *Neocypholaelaps* from Europe (all in association with *Apis mellifera*), namely from Greece by Emmanouel et al. (1983), from Denmark by Schousboe (1986), from Belgium by Fain and Hosseian (2000), from Slovakia by Fenďa and Lukáš (2014), and from Hungary by Kontschán et al. (2015). The specimens from Belgium and Hungary are reported as *Neocypholaelaps apicola*, remaining as *N. favus*. There is one further finding of *Neocypholaelaps* reported from Europe, a species described as a new *Ameroseius* from Crimean Peninsula by Livshits and Mitrofanov (1975), namely *Ameroseius bregetovae*. It apparently belongs to the genus *Neocypholaelaps* although it was not found in association with flowers or flower-visiting animals.

#### Key to species of *Neocypholaelaps* occurring in Europe (females)

Partial keys to the known species of *Neocypholaelaps* may be found in Evans (1963a), for five species known by that time (*ampullula*, *cocos*, *indicus*, *novaehollandiae* and *stridulans*), in Elsen (1972a), for seven species from Africa (*breviperitrematus*, *capitis*, *crocisae*, *leopoldi*, *novus*, *varipilosus* and *xylocopae*), and Baker and Delfinado-Baker (1985), based on nine world species (*ampullula*, *apicola*, *cocos*, *favus*, *hongkongensis*, *indicus*, *novaehollandiae*, *phooni* and *stridulans*). The most recent and complete key is that of Moraes and Narita (2010), including 18 species here considered to be valid members of the genus.

**Table:** 

1	Setae j4, j6 and z5 of similar form and length as surrounding dorsal setae (except j5), apparently longer than j5	***Neocypholaelaps favus* Ishikawa, 1968** (Plates 64–66)
–	Setae j4, j6 and z5 of similar form and length as j5, apparently shorter than surrounding dorsal setae	***Neocypholaelaps apicola* Delfinado-Baker & Baker, 1983**

### Neocypholaelaps ampullula

Taxon classificationAnimaliaMesostigmataAmeroseiidae

(Berlese, 1910)

Laelaps ampullula Berlese, 1910b: 260.
Cypholaelaps ampullula . — Berlese 1918: 117; Vitzthum 1935: 69; Castagnoli and Pegazzano 1985: 15.
Lagyniphis ampulluta (sic). — Lombardini 1936: 43.
Lagyniphis ampullula . — Evans 1963a: 209; Castagnoli and Pegazzano 1985: 15.
Neocypholaelaps ampullula . — Evans 1963a: 214; Baker and Delfinado-Baker 1985: 232; Moraes and Narita 2010: 43; Narita et al. 2011: 62.

#### Type depository.

Istituto Sperimentale per la Zoologia Agraria, Firenze, Italy.

#### Type locality and habitat.

Indonesia, Java, on Indian honeybee, *Apis cerana indica* (as *Apis indica*) (Hymenoptera).

### Neocypholaelaps apicola

Taxon classificationAnimaliaMesostigmataAmeroseiidae

Delfinado-Baker & Baker, 1983

Neocypholaelaps apicola Delfinado-Baker & Baker, 1983: 2.
Neocypholaelaps apicola . — Baker and Delfinado-Baker 1985: 232; Fain and Hosseinian 2000: 33; Moraes and Narita 2010: 43.

#### Type depository.

National Museum of Natural History, Systematic Entomology Laboratory, USDA-ARS, Beltsville Agricultural Research Centre, Beltsville, Maryland, USA.

#### Type locality and habitat.

Pakistan, Rawalpindi, in brood combs of Indian honeybee, *Apis cerana indica* (Hymenoptera).

### Neocypholaelaps breviperitrematus

Taxon classificationAnimaliaMesostigmataAmeroseiidae

Elsen, 1972

Neocypholaelaps breviperitremata Elsen, 1972a: 28.
Neocypholaelaps breviperitremata . — Moraes and Narita 2010: 43.

#### Type depository.

Musée Royal de l'Afrique Centrale, Tervuren Belgium.

#### Type locality and habitat.

Democratic Republic of Congo (as Zaire), Uélé, Bambesa, on apid bee, *Ctenoplectra bequaerti* (Hymenoptera).

### Neocypholaelaps capitis

Taxon classificationAnimaliaMesostigmataAmeroseiidae

Elsen, 1972

Neocypholaelaps capitis Elsen, 1972a: 21.
Neocypholaelaps capitis . — Moraes and Narita 2010: 43.

#### Type depository.

Musée Royal de l'Afrique Centrale, Tervuren Belgium.

#### Type locality and habitat.

Democratic Republic of Congo (as Zaire), Sankuru, Batempas, on digger wasp, *Sphex tomentosus* (as *Sphex tuberculatus*) (Hymenoptera).

### Neocypholaelaps ceylonicus

Taxon classificationAnimaliaMesostigmataAmeroseiidae

Narita & Moraes, 2011

Neocypholaelaps ceylonicus Narita & Moraes (in Narita et al., 2011: 60).
Neocypholaelaps ceylonicus . — Silva et al. 2014: 714.

#### Type depository.

Departamento de Entomologia e Acarologia, Escola Superior de Agricultura “Luiz de Queiroz”, Universidade de São Paulo, Piracicaba, São Paulo, Brazil.

#### Type locality and habitat.

Sri Lanka, Kalpitiya, on inflorescence of coral tree, *Erythrina* sp. (*Fabaceae*).

#### Remarks.

I suspect this species to be a junior synonym of *Neocypholaelaps stridulans* (Evans, 1963). Differential characters for separating *Neocypholaelaps ceylonicus* from *N. stridulans* given by Narita and Moraes (2011) and Silva et al. (2014) are questionable and not well founded.

### Neocypholaelaps cocos

Taxon classificationAnimaliaMesostigmataAmeroseiidae

Evans, 1963

Neocypholaelaps cocos Evans, 1963a: 221.
Neocypholaelaps cocos . — Baker and Delfinado-Baker 1985: 232; Halliday 1997: 197; Moraes and Narita 2010: 43; Narita et al. 2011: 62.

#### Type depository.

British Museum (Natural History), London, United Kingdom.

#### Type locality and habitat.

Pacific Ocean Region, Solomon Islands, Guadalcanal, Honiara, on inflorescence of coconut palm, *Cocos nucifera* (Arecaceae).

### Neocypholaelaps crocisae

Taxon classificationAnimaliaMesostigmataAmeroseiidae

Elsen, 1972

Neocypholaelaps crocisae Elsen, 1972a: 25.
Neocypholaelaps crocisae . — Moraes and Narita 2010: 43.

#### Type depository.

Musée Royal de l'Afrique Centrale, Tervuren Belgium.

#### Type locality and habitat.

Democratic Republic of Congo (as Zaire), Mombasa, Irumu, on cuckoo bee, *Thyreus bouyssoui* (as *Crocisa bouyssoni*) (Hymenoptera).

### Neocypholaelaps favus

Taxon classificationAnimaliaMesostigmataAmeroseiidae

Ishikawa, 1968

Plates 64 , 65 , 66

Neocypholaelaps favus Ishikawa, 1968: 38.
Neocypholaelaps favus . — Ishikawa 1972: 100; Haragsim et al. 1978: 57; Baker and Delfinado-Baker 1985: 232; Karg 1993: 221; Moraes and Narita 2010: 43; Fenďa and Lukáš 2014: 173; Kontschán et al. 2015: 243.
Ameroseius bregetovae Livshits & Mitrofanov, 1975: 463. **Syn. n.**
Neocypholaelaps apicola . — Kontschán et al. 2015: 238. **Misidentification.**

#### Type depository.

Of *Neocypholaelaps favus* – Biological Laboratory, Matsuyama Shinonome Junior College, Matsuyama, Japan; of *Ameroseius bregetovae* – Nikita Botanical Gardens, National Scientific Center, Yalta, Crimea, Russia (the type specimens not found in the collection and probably lost, based on personal communication from Alex Khaustov).

#### Type locality and habitat.

Of *Neocypholaelaps favus* – Japan, Matsuyama, Shikoku, on European honey bee, *Apis mellifera* (Hymenoptera); of *Ameroseius bregetovae* – Russia, Crimea, Alupka, in litter.

#### Comparative material.

Japan: 1 ♀ (CKI, paratype) – 20. 4. 1968, Matsuyama, K. Ishikawa.

#### Published and verified material from Slovakia.

Ipeľská Kotlina Basin: Veľký Krtíš Town. Malé Karpaty Mts.: Bratislava Capital, Devín Settlement, Fialková Dolina Valley. Podunajská Rovina Flatland: Blatná Na Ostrove Village. All records published by Fenďa and Lukáš (2014).

#### Remarks.

In the original descriptive paper on *Neocypholaelaps apicola* from Pakistan by Delfinado-Baker and Baker (1983), the main character for distinguishing this species from the other congeners was based on the reduced length of some setae in central region of the dorsal shield (j4–j6 and z5 in female, and j5 in male and deutonymph), when they are compared with other dorsal shield setae. Unfortunately, those authors did not provide any metric data for these setae and a comparison with the most related species, *Neocypholaelaps favus*. Later, in their identification key to the species of *Neocypholaelaps*, Baker and Delfinado-Baker (1985) misinterpreted the form of dorsal setae in *N. favus*. They separated *N. favus* from *N. apicola* (see their couplet 3) by the presence of leaf-like dorsal setae in *N. favus*, whereas those setae are regularly thickened in this species.

A relatively large number of *Neocypholaelaps favus* collected from debris on the bottom of bee hives in Slovakia enabled an adequate examination of the morphology of this species. The length and form of some medially inserted setae (except j5) showed a relatively high degree of variability. Setae j4, j6 and z5 are more or less abbreviated and narrowed in almost all Slovakian females of *N. favus*. But mostly in freshly moulted, albescent and weakly sclerotised specimens, these setae are usually better developed, relatively larger and thicker than in the older, brown and strongly sclerotised adult females (rarely j4 and j6 are subequal with j3 and J2). I have compared the Slovakian specimens with a paratype female of *N. favus* (kindly sent me by Kazuo Ishikawa), and I could not find any important differences. In *N. favus*, j5 are the shortest and often subequal in adults of both sexes, and they can be smooth or with fine pilosity.

Based on the chaetotaxy described above, and a series of photos taken from holotype of closely related *Neocypholaelaps apicola* received from Debbie Creel and Ronald Ochoa (USDA ARS, Beltsville Agricultural Research Center, Systematic Entomology Laboratory), I consider *Neocypholaelaps favus* and *N. apicola* as two distinct species, and *Neocypholaelaps bregetovae* n. comb. as a synonym of *N. favus*. In addition, *Neocypholaelaps apicola* sensu Kontschán et al. (2015) is considered here as a misidentification of *N. favus*.

**Plate 66.**
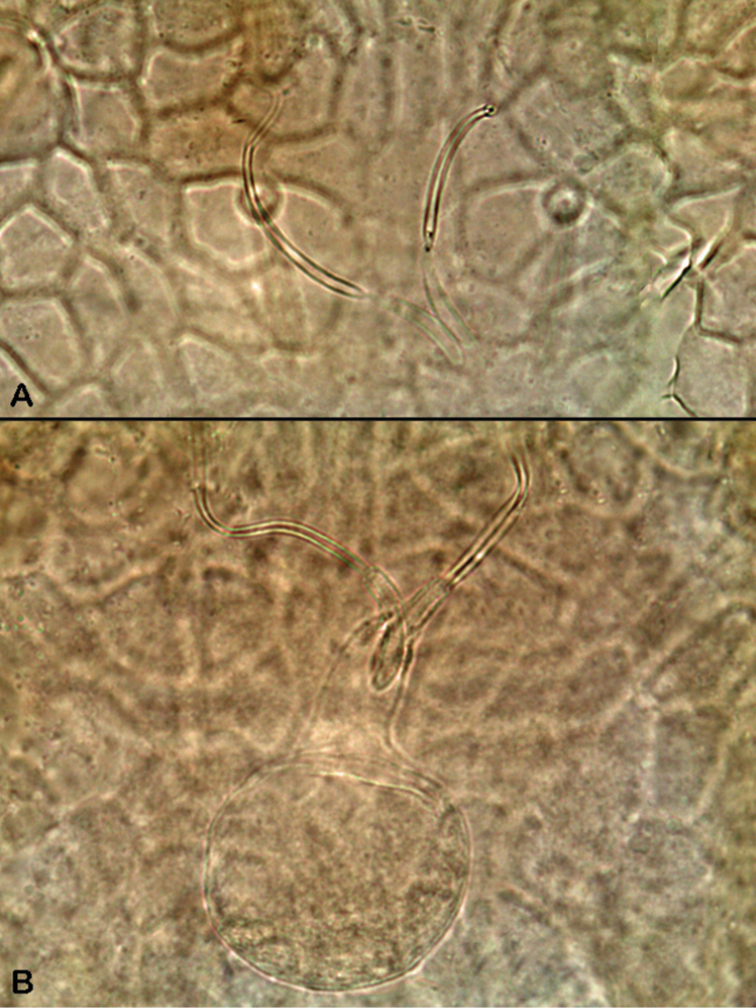
*Neocypholaelaps favus*, sperm induction structures in immature female. **A** spermathecal ducts (*tubuli annulati*) **B** spermathecal ducts associated with *sacculus foemineus*.

The sperm induction structures of the female, especially the structure of *sacculus foemineus* and associated *tubuli annulati*, were considered to be important for separation of some *Neocypholaelaps* species (*ampullula*, *cocos*, *indicus*, *novaehollandiae* and *stridulans*) in taxonomic work of Evans (1963a). Because of the weak sclerotization of these structures, it is not easy to discern the sacculus in *Neocypholaelaps favus*, especially in dark brown mature specimens (Plate 66A). In some freshly moulted and weakly sclerotised immature females of *N. favus* I could detect the *sacculus foemineus* as small, conspicuous and well outlined pear-shaped structure having well developed neck-like process that is connected with *tubuli annulati* (Plate 66B); the *tubuli* are free and enter the narrowed part of the *sacculus* separately. In adult females with egg, or in females after oviposition, the *sacculus foemineus* with neck-like process was not detectable in any of examined specimens (probably due to its conspicuous expansion), and the *tubuli annulati* had their bases well distant from each other, and much more separated when compared with immature females of *N. favus*.

### Neocypholaelaps geonomae

Taxon classificationAnimaliaMesostigmataAmeroseiidae

Moraes & Narita, 2010

Neocypholaelaps geonomae Moraes & Narita, 2010: 38.
Neocypholaelaps geonamae (sic). — Moraes and Narita 2010: 39, 40.

#### Type depository.

Departamento de Entomologia e Acarologia, Escola Superior de Agricultura “Luiz de Queiroz”, Universidade de São Paulo, Piracicaba, São Paulo, Brazil.

#### Type locality and habitat.

Brazil, State of São Paulo, São Pedro, on inflorescence of palm tree, *Geonoma brevispatha* (Arecaceae).

### Neocypholaelaps hongkongensis

Taxon classificationAnimaliaMesostigmataAmeroseiidae

Mo, 1969

Neocypholaelaps hongkongensis Mo, 1969: 96.
Neocypholaelaps hongkongensis . — Treat 1975: 115; Baker and Delfinado-Baker 1985: 232.
Neocypholaelaps kongkongensis (sic). — Ho et al. 2010: 92.
Neocypholaelaps hongkongenis (sic). — Moraes and Narita 2010: 42.

#### Type depository.

Department of Biology, New Asia College, The Chinese University of Hongkong, Hong Kong, China.

#### Type locality and habitat.

China, Hong Kong, Kowloon, Lunggutan, on blue spotted crow, *Euploea midamus* (as *Euploea minamus*) (Lepidoptera).

#### Remarks.

According to Moraes and Narita (2010), this species could be a member of the genus *Afrocypholaelaps* based on smooth dorsal shield and smooth, pointed and subequal dorsal shield setae.

### Neocypholaelaps indicus

Taxon classificationAnimaliaMesostigmataAmeroseiidae

Evans, 1963

Neocypholaelaps indica Evans, 1963a: 217.
Neocypholaelaps indica . — Teng and Pan 1964: 772; Mo 1971: 97; Mo 1972: 15; Treat 1975: 116; Baker and Delfinado-Baker 1985: 232; Haitlinger 1987a: 365; Verma and Singh 1999: 51; Ma and Lin 2006: 241; Moraes and Narita 2010: 43; Fan and Jiang 2014: 248.
Neocypholaelaps ewae Haitlinger, 1987a: 363. **Syn. n.**
Neocypholae lapsindica (sic). — Lin et al. 2007: 128.
Neocypholaelaps ewae . — Ho et. al. 2010: 91; Moraes and Narita 2010: 42.
Hattena ewae . — Narita et al. 2013a: 13; Klimov et al. 2016.
Afrocypholaelaps ewae . — Klimov et al. 2016.

#### Type depository.

Of *Neocypholaelaps indica* – British Museum (Natural History), London, United Kingdom; of *Neocypholaelaps ewae* – Museum of Natural History, Wrocław University, Poland.

#### Type locality and habitat.

Of *Neocypholaelaps indica* – Sri Lanka (as Ceylon), on Indian honeybee, *Apis cerana indica* (as *Apis indica*) (Hymenoptera); of *Neocypholaelaps ewae* – Vietnam, Danang, on unidentified butterfly (Lepidoptera).

#### Comparative material.

Vietnam: 1 ♀, 1 ♂, 1 protonymph (MPUV: MP-1290, syntypes) – 20. 2. 1985, Danang, Lepidoptera (niebieski motyl) (labelled *Neocypholaelaps ewae*, holotyp).

#### Remarks.

The original description and illustrations of *Neocypholaelaps ewae* are not detailed and consistent enough to allow it to be correctly recognised and classified in a genus with any confidence. Therefore, Narita et al. (2013a) and Klimov et al. (2016) transferred this species to *Hattena*, without providing any explanation for the new systematic position. The number of 19 pairs of dorsal shield setae stated for the adults (and developmental stages, protonymph and deutonymph) by Haitlinger (1987a) perfectly fits to the setal patterns of *Hattena*. But many other characters resembling those in *Neocypholaelaps*, especially *Neocypholaelaps indicus*: large subrectangular epigynal shield with slight posterior expansion, dorsal setae smooth (except j1), anal shield with only three circum-anal setae in adults of both sexes, cheliceral spermatodactyl thin and long, J2 of male conspicuously thickened and lengthened, protonymph with S4 thickened and spur-like. My examination of the type specimens of *N. ewae* has shown that the statement on decreased complement of the dorsal setae is misinterpreted and in error. I could found all 29 pairs of setae on the dorsal shield in examined adults of both sexes. Using the key to species of *Neocypholaelaps* by Evans (1963a) and observing the type female from Vietnam, it can be identified as *N. indicus* by the following characteristics: dorsal shield has 29 pairs of setae (entry 1), most of which setiform and at the most weakly serrated (entry 2), all leg ambulacra with two claws and genu and tibia III each with two anterolateral setae (entry 3), Z5 short (about 20 μm long in type female), dorsal setae of tibia I smooth, and anal shield approximately 104 × 135 μm in size in type female (entry 4). Accordingly, a new synonymy is established between *N. ewae* and *N. indicus* in the present study.

### Neocypholaelaps kreiteri

Taxon classificationAnimaliaMesostigmataAmeroseiidae

Narita, Pédelabat & Moraes, 2013

Neocypholaelaps kreiteri Narita, Pédelabat & Moraes, 2013a: 2.

#### Type depository.

Departamento de Entomologia e Acarologia, Escola Superior de Agricultura “Luiz de Queiroz”, Universidade de São Paulo, Piracicaba, São Paulo, Brazil; SupAgro INRA Acarology Collection, Montpellier, France.

#### Type locality and habitat.

Indian Ocean Region, Réunion Island, on inflorescence of coconut palm, *Cocos nucifera* (Arecaceae).

### Neocypholaelaps leopoldi

Taxon classificationAnimaliaMesostigmataAmeroseiidae

Elsen, 1972

Neocypholaelaps leopoldi Elsen, 1972a: 23.
Neocypholaelaps leopoldi . — Moraes and Narita 2010: 43.

#### Type depository.

Musée Royal de l'Afrique Centrale, Tervuren Belgium.

#### Type locality and habitat.

Democratic Republic of Congo (as Zaire), Boma Kandi, on cuckoo bee, *Thyreus interruptus* (as *Crocisa interrupta*) (Hymenoptera).

### Neocypholaelaps malayensis

Taxon classificationAnimaliaMesostigmataAmeroseiidae

Delfinado-Baker, Baker & Phoon, 1989

Neocypholaelaps malayensis Delfinado-Baker, Baker & Phoon, 1989: 612.
Neocypholaelaps malayensis . — Moraes and Narita 2010: 43.

#### Type depository.

National Museum of Natural History, Systematic Entomology Laboratory, USDA-ARS, Beltsville Agricultural Research Centre, Beltsville, Maryland, USA.

#### Type locality and habitat.

Malaysia, Selangor, Puchong, in nest of Indo-Malayan stingless bee, *Heterotrigona itama* (as *Trigona itama*) (Hymenoptera).

### Neocypholaelaps novus

Taxon classificationAnimaliaMesostigmataAmeroseiidae

Elsen, 1972

Neocypholaelaps nova Elsen, 1972a: 20.
Neocypholaelaps nova . — Moraes and Narita 2010: 43.

#### Type depository.

Musée Royal de l'Afrique Centrale, Tervuren Belgium.

#### Type locality and habitat.

Democratic Republic of Congo (as Zaire), Bambesa, on digger bee, *Amegilla acraensis* (as *Anthophora acraensis*) (Hymenoptera).

### Neocypholaelaps novaehollandiae

Taxon classificationAnimaliaMesostigmataAmeroseiidae

Evans, 1963

Neocypholaelaps novaehollandiae Evans, 1963a: 223.
Neocypholaelaps novae-hollandiae (sic). — Baker and Delfinado-Baker 1985: 231.
Neocypholaelaps novaehollandia (sic). — Delfinado-Baker et al. 1989: 610.
Neocypholaelaps novaehollandiae . — Baker and Delfinado-Baker 1985: 232; Moraes and Narita 2010: 42; Kar et al. 2015: 126.

#### Type depository.

British Museum (Natural History), London, United Kingdom.

#### Type locality and habitat.

New Zealand, Auckland, on European honey bee, *Apis mellifera* (Hymenoptera).

### Neocypholaelaps phooni

Taxon classificationAnimaliaMesostigmataAmeroseiidae

Baker & Delfinado-Baker, 1985

Neocypholaelaps phooni Baker & Delfinado-Baker, 1985: 228.
Neocypholaelaps phooni . — Moraes and Narita 2010: 43.

#### Type depository.

National Museum of Natural History, Systematic Entomology Laboratory, USDA-ARS, Beltsville Agricultural Research Centre, Beltsville, Maryland, USA.

#### Type locality and habitat.

Malaysia, Selangor, Serdang, in nest of Indo-Malayan stingless bee, *Geniotrigona thoracica* (as *Trigona thoracica*) (Hymenoptera).

### Neocypholaelaps pradhani

Taxon classificationAnimaliaMesostigmataAmeroseiidae

Gupta, 1969

Neocypholaelaps pradhani Gupta, 1969: 335.
Neocypholaelaps pradhani . — Gupta 1985: 327; Moraes and Narita 2010: 43.

#### Type depository.

Zoological Survey of India, Calcutta, India.

#### Type locality and habitat.

India, Kodaikanal, Madras, on flowers of apple tree, *Malus pumila* (as *Pyrus malus*) (Rosaceae).

#### Remarks.

I suspect this species to be a junior synonym of *Neocypholaelaps indicus* Evans, 1963.

### Neocypholaelaps stridulans

Taxon classificationAnimaliaMesostigmataAmeroseiidae

(Evans, 1955)

Indoseius stridulans Evans, 1955b: 107.
Neocypholaelaps stridulans . — Evans 1963a: 219; Baker and Delfinado-Baker 1985: 232; Gupta 1985: 327; Haq 2001: 361; Moraes and Narita 2010: 43; Narita et al. 2011: 62.
Sertitympanum stridulans . — Hallan 2005.

#### Type depository.

British Museum (Natural History), London, United Kingdom.

#### Type locality and habitat.

India, Upatte, on inflorescence of coconut palm, *Cocos nucifera* (Arecaceae).

### Neocypholaelaps varipilosus

Taxon classificationAnimaliaMesostigmataAmeroseiidae

Elsen, 1972

Neocypholaelaps varipilosa Elsen, 1972a: 22.
Neocypholaelaps varipilosa . — Moraes and Narita 2010: 43.

#### Type depository.

Musée Royal de l'Afrique Centrale, Tervuren Belgium.

#### Type locality and habitat.

Democratic Republic of Congo (as Zaire), Sankuru, Batempas, on digger wasp, *Sphex tomentosus* (as *Sphex tuberculatus*) (Hymenoptera).

### Neocypholaelaps wilsoni

Taxon classificationAnimaliaMesostigmataAmeroseiidae

(Allred, 1970) comb. n.

Ameroseius wilsoni Allred, 1970: 99.

#### Type depository.

Bernice Pauahi Bishop Museum (Hawaii State Museum of Natural and Cultural History), Honolulu, Hawaii, USA; United States National Museum, Washington, D.C., USA; Queensland Institute of Medical Research, Brisbane, Australia; British Museum (Natural History), London, United Kingdom (not stated in the paper).

#### Type locality and habitat.

Papua New Guinea, Wau Creek, from the nasal cavity of rainbow lorry, *Trichoglossus haematodus* (Aves, Psittaciformes).

#### Comparative material.

New Guinea: 1 ♀ (BMNH, paratype) – 1963, Wau Creek, rainbow lorry, leg. P. Shanahan, Bishop Museum, BBM-NG 20425.

#### Remarks.

I examined a well preserved paratype of *Ameroseius wilsoni* deposited at the Natural History Museum in London, concluding that it should be included in *Neocypholaelaps*. The original description and illustrations given by Allred (1970) are quite adequate and do not require amendment, except for the sperm induction apparatus. In the studied specimen, spermathecal ducts (*tubuli annulati*) are well indicated, and they enter the *sacculus foemineus* independently.

### Neocypholaelaps xylocopae

Taxon classificationAnimaliaMesostigmataAmeroseiidae

Elsen, 1972

Neocypholaelaps xylocopae Elsen, 1972a: 26.
Neocypholaelaps xylocopae . — Moraes and Narita 2010: 42.

#### Type depository.

Musée Royal de l'Afrique Centrale, Tervuren Belgium.

#### Type locality and habitat.

Democratic Republic of Congo (as Zaire), Bambesa, on carpenter bee, *Xylocopa hottentotta* (as *Xylocopa carinata*) (Hymenoptera).

### Genus Pseudoameroseius gen. n.

Taxon classificationAnimaliaMesostigmataAmeroseiidae

http://zoobank.org/D3FBB56C-0CFA-4020-A719-7BDBDA1286DE

#### Type species.

*Ameroseius michaelangeli* Moraza, 2006.

#### Diagnosis (female).

Dorsal shield heavily sclerotised and coarsely ornamented, with reduced number of 21 pairs of setae (j1–j6, J2, J4, z4, z5, Z2, Z5, s1, s4, s5, S2–S5, r3 and r4 present; z2, z6, Z1, Z3, s2, s6, r2 and r5 absent). Dorsal setae including j1 similar in length and form, thickened, with serrate edges. Sternal setae on sternal shield (st1, st2), soft integument (st3, st4), and epigynal shield (st5). Opisthogastric region with reduced number of four pairs of setae (JV1, JV2, JV5 and ZV2 present; JV3 and JV4 absent), two of which on ventrianal shield (JV2, ZV2). Setae JV1 and JV5 on soft integument. Ventrally inserted setae smooth and needle-like, except JV5, longer and slightly spine-shaped. Gnathosoma relatively small. Corniculi weakly sclerotised distally, membranous, with undivided apex. Cheliceral digits relatively small, fixed digit with two teeth on medial masticatory area. Epistome subtriangular, with irregularly and obtusely curved apex. Palptarsal apotele three-tined. Genu III, and tibiae III–IV with two anterolateral and one posterolateral setae. Tarsi I–IV each with empodium and claws, apotele of tarsi I sessile.

#### Remarks.

*Pseudoameroseius* gen. n. seems to be monotypic, with *Pseudolaelaps michaelangeli* described from soil and leaf litter in Tenerife Island, Canary Islands (Moraza 2006).

### Pseudoameroseius michaelangeli

Taxon classificationAnimaliaMesostigmataAmeroseiidae

(Moraza, 2006) comb. n.

Plate 67

Ameroseius michaelangeli Moraza, 2006: 163.

#### Type depository.

Museum of Zoology, University of Navarra, Pamplona, Spain; Acarology Collection, Ohio State University, Columbus, Ohio, USA.

#### Type locality and habitat.

Spain, Canary Islands, Tenerife Island, Punta del Diablo (Teno), from soil and litter of *Cheirolophus burchardii* (as Cheirolophus canariensis subsp. subexpinnatus) (Asteraceae) and *Euphorbia aphylla* (Euphorbiaceae).

#### Comparative material.

Spain: 2 ♀♀ (MZNA: 443694, holotype; MZNA: 443695, paratype) – 8. 12. 1995, Islas Canarias, Tenerife, Punta del Diablo (Teno), Altitud 200 m, UTM: 28RCS 141 384.

#### Remarks.

Moraza (2006) added complete leg chaetotaxy to the description of *michaelangeli*. According to her original data, genu and tibia of legs IV would have one anterolateral seta and two posterolaterals, but in the type specimens I found the typical two anterolaterals and one posterolateral seta instead (including some other slight differences in leg chaetotaxy, as detected by a comparison with data presented in Table 1 and in the original description).

### Genus Sertitympanum

Taxon classificationAnimaliaMesostigmataAmeroseiidae

Elsen & Whitaker, 1985

Sertitympanum Elsen & Whitaker, 1985: 119. Type species: Sertitympanum separationis Elsen & Whitaker, 1985, by original designation.

#### Diagnosis (adults).

Dorsal shield variously sclerotised and ornamented, with 28–29 pairs of setae, including z6; setae Z2 absent in some species. Dorsal shield setae similar in adults of both sexes, relatively short (with tips never reaching bases of the following setae of the same longitudinal rows), mostly smooth and spatulate, sometimes crenelated, paddle-shaped or spoon-shaped, with a broad flat end tapering to the base; setae j1 otherwise modified, smooth or serrate, usually with pointed apex, and often with ventral position on vertex. In female, st1 and st2 on sternal shield, st3 on small suboval or subtriangular pseudo-metasternal platelets, and st4 on soft integument; endopodal platelets II-III well developed, relatively large, subtriangular and close to posterolateral angles of sternal shield; anterior margin of epigynal shield usually deeply concave to form two cusps, genital poroids on soft integument. Opisthogastric region with five pairs of setae in females and males (JV1–JV3, JV5, ZV2; JV4 absent); setae JV5 usually similar to those on dorsal shield, other ventrally inserted setae simple, smooth and needle-like; females with three pairs of opisthogastric setae (JV1, JV5, ZV2) on soft integument and two pairs (JV2, JV3) on ventrianal shield; male with more expanded ventrianal shield bearing all five pairs of opisthogastric setae. Peritrematal shields anteriorly fused to dorsal shield, with enlarged cavity-like poroid structure at the level of coxa III and well developed parapodal portion. Metapodal platelets relatively large, longitudinally elongate. Soft striate integument simple, not with additional sclerotic granulation. Corniculi normally sclerotised, well spaced and trifid; setae h1 markedly thickened proximally, curved and abruptly tapered medially, attenuate distally, and sharply pointed apically. Fixed digit of chelicera with three subequal teeth on proximal masticatory area; male spermatodactyl small, shorter than movable cheliceral digit. Epistome with anterior margin produced into narrow and bifid central projection; lateral margins with several minute spines. Palptarsal apotele two-tined. Coxae I–IV with rows of minute spines, coxae II with spine-like process on anterior surface; coxae I–IV and femora I–IV with lamellar and/or papular sclerotic structures. Genu III, and tibiae III–IV with two anterolateral and two posterolateral setae. Tarsi I–IV each with empodium and rudimentary claws, or claws absent. Insemination apparatus with papilla-like sperm induction pores (solenostomes) associated with inner margin of coxae III; spermathecal ducts free and elongated.

#### Remarks.

Elsen and Whitaker (1985) proposed the genus *Sertitympanum* to accommodate three species based on females and found in North America, the type species *Sertitympanum separationis*, as well as *Sertitympanum exarmatum* and *Sertitympanum contiguum*. They characterised this genus by the presence of leg excrescences, peculiar cog-wheel-like sculpture on sternal shield and presence of a pair of large ventrolateral lenticular metapodal platelets (and by many other characters). These characters were considered by them to be the most important and distinct for their newly established genus, although they can be detected also in the other ameroseiid species, especially in the genus *Kleemannia* (for example, rounded and well scalloped circular structure on the sternal shield can be found in *Kleemannia plumosa*).

The two further species of the genus, described from the North Africa (Egypt) by Nasr and Abou-Awad (1986), are lacking the cog-wheel-like sculptural pattern on the sternal shield, but despite this and slightly different appearance they are congeneric with the species from the American continent in many character states as follows: (1) form of dorsal shield setae; (2) dorsal chaetotaxy (z6 present and Z2 absent, as in *Sertitympanum separationis*); (3) general arrangement of ventral shields; (4) bicuspid anterior margin of epigynal shield; (5) chaetotaxy of opisthogastric soft integument and ventrianal shield; (6) presence of cavity-like poroid structure on peritrematal shield; (7) presence of papilla-like sperm induction pores close to coxae III; (8) sclerotic sculpture and chaetotaxy of legs; (9) reduction of tarsal claws in legs I–IV (not stated in an original description of the genus, but found in *S. separationis* in which the claws are fully reduced); (10) special form of h1; (11) dentation of chelicera; (12) trifid form of corniculi; (13) bifurcate form of epistome (not stated in an original description of the genus but found in *S. separationis*); and (14) two-tined form of palptarsal apotele.

*Sertitympanum* is similar in some respects to *Kleemannia*. Both genera have the same leg chaetotaxy, two pairs of opisthogastric setae on ventrianal shield, and arrangement and shape of some ventral shields. As a result of this similarity and the absence of a cog-wheel-like sculpture on sternal shield in some species, three *Sertitympanum* species were initally described in *Kleemannia* (*bassolsae*, *nodosum* and *zaheri*) and classified in this genus up to now. *Sertitympanum* can be distinguished from *Kleemannia* by the following characters: (1) dentation of fixed cheliceral digit (three teeth in *Sertitympanum*, four in *Kleemannia*); (2) structure of ambulacral apparatus (claws rudimentary or absent in *Sertitympanum*, claws normally developed in *Kleemannia*); (3) form of central process of epistome (furcate in *Sertitympanum*, undivided in *Kleemannia*); (4) form of h1 (thickened proximally, curved and attenuate medially, subfalcate in *Sertitympanum*; thin or thickened, always straight and regularly tapered in *Kleemannia*); (5) dorsal setation in forms with 28 pairs of dorsal shield setae (z6 present and Z2 absent in *Sertitympanum*, z6 absent and Z2 present in *Kleemannia*); (6) length and form of dorsal setae (shorter, with their apices and bases well separated, leaf-shaped, oblanceolate, spatulate or obovate, with rounded apex in *Sertitympanum*; longer, with their apices and bases adjacent or overlapping, tubiform or feather-shaped, with pointed apex in *Kleemannia*).

There were five very similar species described to this time (*aegyptiacum*, *bassolsae*, *nodosum*, *palmatum* and *zaheri*), with only simple reticulation on the sternal shield, although some curved sculptural lines, and perhaps also shallow depression, are present and better defined. *Sertitympanum nodosum* was introduced first (Sheals, 1962), based only on a single specimen, a weakly sclerotised and medially distorted female, found in Argentina. Unfortunately, the followers overlooked the existence of *S. nodosum* in a differential diagnosis to their new species (El-Badry et al. 1979, Nasr and Abou-Awad 1986, Vargas and Polaco 2001).

*Sertitympanum* needs a thorough revision, and it comprises eight species considered here to be valid, most of which are widespread in nests of small mammals (species from North America), soil detritus and litter (species from Egypt). In Iran, *Sertitympanum aegyptiacum* is reported from honey bee hives, stored rice, rice dust and debris, and soil of vineyards (Kazemi and Rajaei 2013). The genus *Sertitympanum* is here firstly recorded from Europe (see remarks in *Sertitympanum nodosum*).

### Sertitympanum aegyptiacum

Taxon classificationAnimaliaMesostigmataAmeroseiidae

Nasr & Abou-Awad, 1986

Plate 68

Sertitympanum aegyptiacus Nasr & Abou-Awad, 1986: 77.
Sertitmypanum (sic) aegyptiacus. — Nasr and Abou-Awad 1986: 81.
Sertitympanum aegyptiacus . — Hajizadeh et al. 2013a: 150; Kazemi and Rajaei 2013: 67; Nemati et al. 2013: 21; Khalili-Moghadam and Saboori 2014: 674.

#### Type depository.

National Research Centre, Dokki, Cairo, Egypt.

#### Type locality and habitat.

Egypt, Sohag Region, Girga, in litter.

#### Comparative material.

Iran: 1 ♀ (IZSAV) – Guilan Province, rice storage, No. 11, leg. J. Hajizadeh.

#### Remarks.

I regard *Sertitympanum aegyptiacum* as valid and recognisable species (I examined two specimens collected in a rice storage, Guilan Province, Iran). It may be distinguished from the very similar *Sertitympanum nodosum* especially by the length of the dorsal shield setae (apparently longer in *S. aegyptiacum*).

### Sertitympanum contiguum

Taxon classificationAnimaliaMesostigmataAmeroseiidae

Elsen & Whitaker, 1985

Plate 69

Kleemania (sic) sp. — Allred and Beck 1966: 16.
Sertitympanum contiguum Elsen & Whitaker, 1985: 122.
Sertitympanum contiguum . — Bassols et al. 1993: 4.

#### Type depository.

United States National Museum, Washington, D.C., USA.

#### Type locality and habitat.

USA, Oregon, Crowley Guard Center, on Ord‘s kangaroo rat, *Dipodomys ordii* (Mammalia, Rodentia).

#### Comparative material.

U.S.A.: 1 ♀ (BMNH) – 12. 4. 1955, Co. Utah, Camelback Mt., Dugway, Tooele, host: *Peromyscus maniculatus*, AH124:005, det. H. Hurlbutt (labelled *Kleemannia plumosus*); 1 ♀ (BMNH) – 12. 5. 1955, Co. Utah, Stansbury Mts., Dugway, Tooele, host: *Dipodomys ordii*, AH154:008, det. H. Hurlbutt (labelled *Kleemannia plumosus*).

#### Remarks.

I have seen two females of this species from the British Museum Collection in London, both collected from small mammals in Utah in 1955, and additionaly identified by H. Hurlbutt as *Kleemannia plumosa*. These specimens are most probably a part at least of the material forming the record by Allred and Beck (1966) of *Kleemania* (sic) sp., who added also some fragmentary illustrations to this unidentified species. The original description given by Elsen and Whitaker (1985) requires a small amendments: vertex simple, not produced into bifid spur supporting j1 as stated in the original generic diagnosis; setae j1 smooth, relatively short and thick, wedge-shaped, with ventral position on vertex; epistome with bifurcate central process and spinate lateral margins; tarsi of legs I–IV with empodium but no claws).

### Sertitympanum exarmatum

Taxon classificationAnimaliaMesostigmataAmeroseiidae

Elsen & Whitaker, 1985

Sertitympanum exarmatum Elsen & Whitaker, 1985: 119.

#### Type depository.

United States National Museum, Washington, D.C., USA.

#### Type locality and habitat.

USA, Wyoming, Medicine Bow Mountains, on northern pocket gopher, *Thomomys talpoides* (Mammalia, Rodentia).

### Sertitympanum mexicanum

Taxon classificationAnimaliaMesostigmataAmeroseiidae

Villegas-Guzmán, Montiel-Parra, Vargas & Polaco, 2004

Sertitympanum mexicanum Villegas-Guzmán, Montiel-Parra, Vargas & Polaco, 2004: 30.

#### Type depository.

Laboratorio de Acarología, Departamento de Zoología, Escuela Nacional de Ciencias Biológicas, Instituto Politécnico Nacional, Ciudad de México, Mexico; Instituto de Biología, Universidad Nacional Autónoma de México, Ciudad de México, Mexico; United States National Museum, Washington, D.C., USA.

#### Type locality and habitat.

Mexico, Durango State, Santiago Papasquiaro, beside road to La Chaparra (lagoon), in nest of Mexican woodrat, *Neotoma mexicana* (Mammalia, Rodentia).

### Sertitympanum nodosum

Taxon classificationAnimaliaMesostigmataAmeroseiidae

(Sheals, 1962) comb. n.

**Plate 70.**
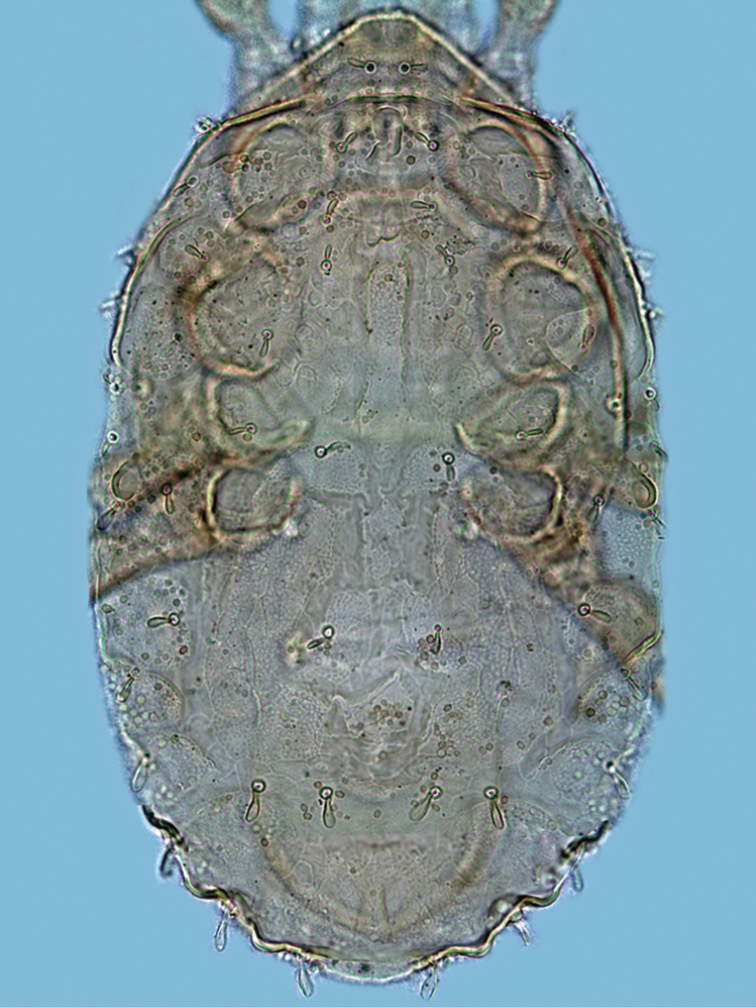
*Sertitympanum nodosum*, female (from Germany), dorsal idiosoma.

**Plate 71.**
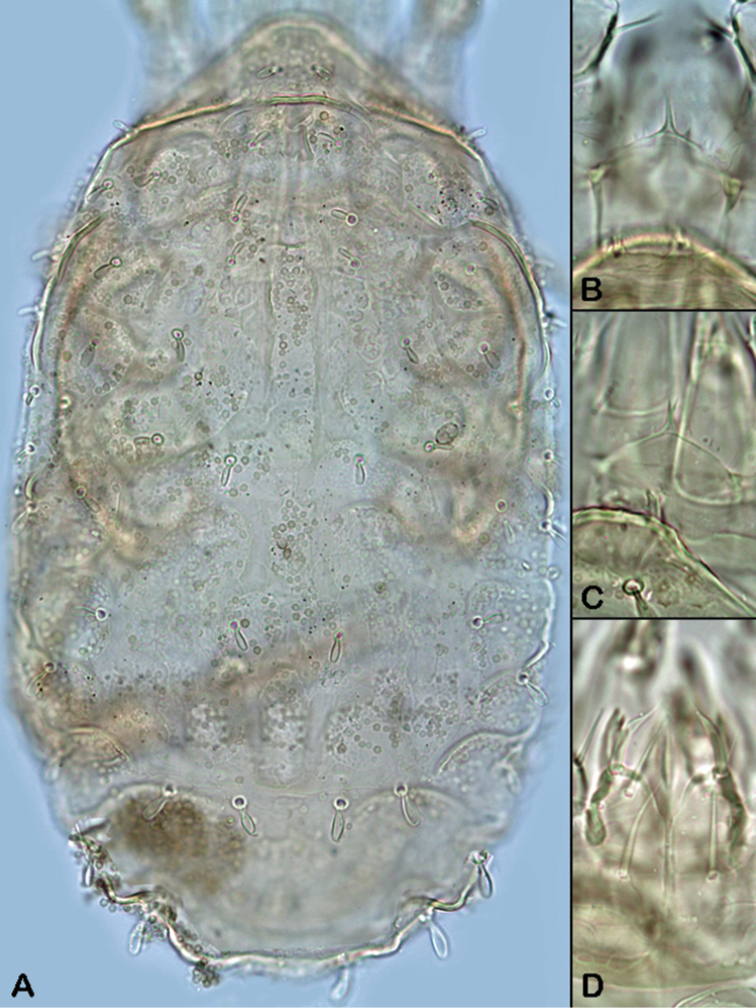
*Sertitympanum nodosum*, female (from Germany). **A** Dorsal idiosoma **B–C** Epistome, variant forms **D** Hypostome, ventral view.

**Plate 76.**
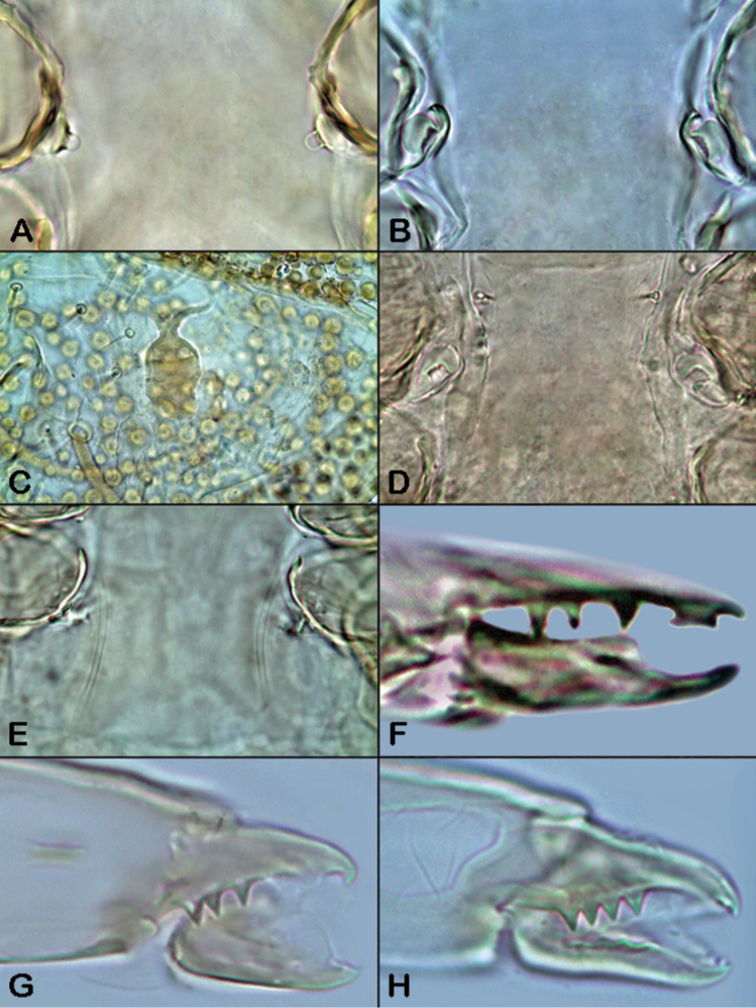
Ameroseiidae spp., female. **A–E** Structures of sperm access system. **A** *Kleemannia delicata*, sperm induction pores (solenostomes) associated with inner margin of coxae III **B** and **D** *Kleemannia plumigera*, solenostomes associated with inner margin of coxae III **C** *Epicriopsis suedus*, *sacculus foemineus* and associated *tubuli annulati* **E** *Sertitympanum nodosum*, *tubuli annulati* **F–H** Chelicera. **F** *Asperolaelaps rotundus* **G** *Ameroseius renatae* **H** *Kleemannia delicata*.

**Plate 77.**
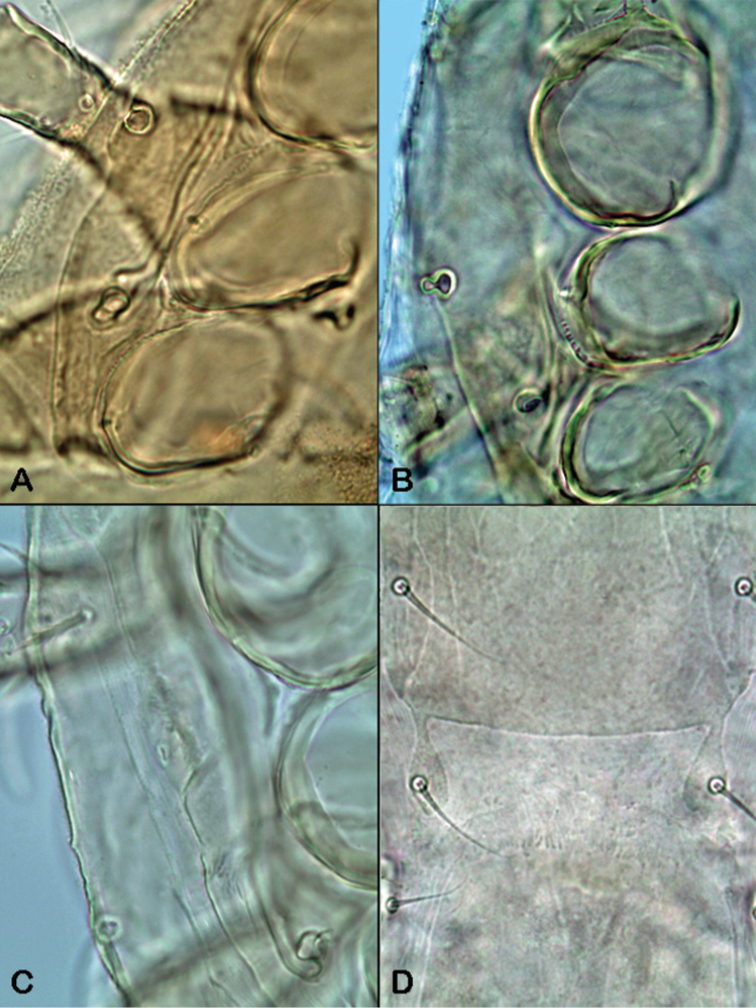
Ameroseiidae spp., female. **A–C** Poroid structure associated with peritrematal shield. **A** *Kleemannia delicata* **B** *Sertitympanum nodosum* **C** *Kleemannia plumigera* **D** Metasternal region in *Kleemannia plumigera*.

Plates 70 , 71 , 76E , 77B

Kleemannia nodosa Sheals, 1962: 99.
Kleemannia bassolsae Vargas & Polaco, 2001: 171.
Ameroseius bassolase (sic). — Kazemi and Rajaei 2013: 64; Khalili-Moghadam and Saboori 2014: 674.
Ameroseius (Kleemannia) bassolsae . — Hajizadeh et al. 2013a: 150.

#### Type depository.

Of *Kleemannia nodosa* – British Museum (Natural History), London, United Kingdom; of *Kleemannia bassolsae* – Laboratorio de Acarología, Departamento de Zoología, Escuela Nacional de Ciencias Biológicas, Instituto Politécnico Nacional, Ciudad de México, Mexico; Instituto de Biología, Universidad Nacional Autónoma de México, Ciudad de México, Mexico; Canadian National Collection of Insects, Arachnids and Nematodes, Research Branch, Agriculture & Agri-Food Canada, Ottawa, Ontario, Canada.

#### Type locality and habitat.

Of *Kleemannia nodosa* – Argentina, Nahuel Huapi National Park, habitat not stated; of *Kleemannia bassolsae* – Mexico, Durango, Laboratorio del Desierto, on Nelson‘s kangaroo rat, *Dipodomys nelsoni* (Mammalia, Rodentia).

#### Comparative material.

Argentina: 1 ♀ (BMNH: 1961.6.2029, holotype) – 39/2, det. J. G. Sheals (labelled *Kleemannia nodosa*). Germany: 5 ♀♀ (ZMH: A2/62) – 1957, Langenhorn, Müllplatz, leg. H. J. Haas, det. G. O. Evans (2 ex.) and G. Rack (3 ex.) in 1964 (labelled *Kleemannia nodosa*); 2 ♀♀ (ZMH: A41/73) – May 1973, Kassel, Neubau, leg. H. Kühne, det. G. Rack in 1973 (labelled *Ameroseius nodosus*); 6 ♀♀ (ZMH: A108/85) – 17. 9. 1985, NW Deutschland, Stade, lästig in einem Badezimmer, leg. U. Zellentin, det. G. Rack in 1985 (labelled *Ameroseius nodosus*).

#### Remarks.

This species was only briefly described by Sheals (1962), and his description needs the following amendments, according to an examination of the holotype: (1) dorsal shield sculpture with a specific pattern of straight and curved lamellar structures, less sclerotised reticulate and granulate areas, and shallow depressions in central and marginal portion; the shield scalloped posteromarginally, bearing 28 pairs of setae (there are two incompletely illustrated pairs and one redundant marginal pair in the original figure of dorsum); (2) setae j1 placed ventrally; (3) sternal shield between coxae II and III with endopodal portions well developed; (4) third pair of sternal lyrifissures on soft integument, behind the pseudo-metasternal platelets; (5) peritrematal shields with a pair of enlarged cavity-like poroid structures; (6) posterior margin of ventrianal shield widely rounded; (7) setae JV1, ZV2 and JV5 on soft integument, JV5 spatulate; (8) setae h1, fixed digit of chelicera, epistome and palptarsal apotele well discernible, typical of the genus (see generic diagnosis above); (9) coxae I–IV with rows of spicules, coxae II with a spine on anterior surface; (10) legs I–IV dorsally with lamellar and papular sclerotic structures; (11) leg chaetotaxy typical for the genus; (12) tarsi I–IV each with empodium but no claws; (13) sperm induction pores discernible, papilla-like.

I am unable to find important differences between the holotype of *Sertitympanum nodosum* from Argentina and specimens of *Sertitympanum bassolsae* from Mexico, adequately described and illustrated by Vargas and Polaco (2001). Therefore, a new synonymy is established between the two above mentioned species in this study.

I have examined a large number of *Sertitympanum* specimens from Iran (received from A. Ahadiyat, J. Hajizadeh and Sh. Kazemi). Despite certain variability of the lamellar pattern of the sculptural sclerotization (due to different pressure of the cover glass on mounted specimens), and variable shape of some sternal and ventrianal shields, I consider the specimens from Iran (excluding two mites with longer dorsal setae and identified as *Sertitympanum aegyptiacum*) as conspecific with the holotype of *Sertitympanum nodosum*. In addition, I examined some *Sertitympanum* specimens available in the Rack Collection in Hamburg. They were collected in synanthropic habitats (bathroom, new building, waste disposal area) in north-western Germany (Langenhorn, Kassel, and Stade), labelled *Kleemannia nodosa* or *Ameroseius nodosus*, and identified by G. O. Evans in 1964 or G. Rack in 1964, 1973 and 1985. Here I regard their identification as correct. The first record of *Sertitympanum* in Europe is perhaps unexpected and surprising from a biogeographic and ecological point of view, but specimens from Germany agree in detail with the specimens from South America and Asia. I expect *S. nodosum* to be cosmopolitan species distributed by human activities and associated with various synanthropic habitats, including stored grain and other food products, especially in the temperate climatic regions.

### Sertitympanum palmatum

Taxon classificationAnimaliaMesostigmataAmeroseiidae

Nasr & Abou-Awad, 1986

Sertitympanum palmatus Nasr & Abou-Awad, 1986: 78.

#### Type depository.

National Research Centre, Dokki, Cairo, Egypt.

#### Type locality and habitat.

Egypt, Sohag Region, Girga, in litter.

#### Remarks.

The only obvious difference between this species and *Sertitympanum aegyptiacum* seems to be the form of the dorsal shield setae, undulated to deeply crenelated in the former.

### Sertitympanum separationis

Taxon classificationAnimaliaMesostigmataAmeroseiidae

Elsen & Whitaker, 1985

Sertitympanum separationis Elsen & Whitaker, 1985: 119.
Sertitympanum separationis . — Elsen et al. 1992: 113.

#### Type depository.

United States National Museum, Washington, D.C., USA.

#### Type locality and habitat.

USA, Indiana, Vigo County, Terre Haute, on thirteen-lined ground squirrel, *Ictidomys tridecemlineatus* (as *Spermophilus tridecemlineatus*) (Mammalia, Rodentia).

### Sertitympanum zaheri

Taxon classificationAnimaliaMesostigmataAmeroseiidae

(El-Badry, Nasr & Hafez, 1979) comb. n.

Kleemania (sic) zaheri El-Badry, Nasr & Hafez, 1979: 8.
Kleemannia zaheri . — Zaher 1986: 405.

#### Type depository.

Not stated.

#### Type locality and habitat.

Egypt, Kafr-El-Sheikh Governorate, Sagh, in litter.

#### Remarks.

The only obvious difference between this species and its most similar congeners seems to be the shape of posterior margin of sternal shield which is widely rounded. However, I suspect this species to be a junior synonym of *Sertitympanum nodosum*. Photographed specimens stated to be *zaheri* and originated from Egypt (kindly sent by R. Abo-Shnaf) apparently belong to *S. nodosum*.

### Genus Sinoseius

Taxon classificationAnimaliaMesostigmataAmeroseiidae

Bai & Gu, 1995

Sinoseius Bai & Gu (in Bai et al., 1995: 435). Type species: Sinoseius lobatus Bai, Gu & Fang, 1995, by original designation.

#### Diagnosis (female).

Dorsal shield weakly sclerotised but coarsely ornamented, with 29 pairs of setae (z6 present). Dorsal setae including j1 similar in length and form, odd-pinnate. Sternal setae on sternal shield (st1, st2, st3), soft integument (st4) and epigynal shield (st5); opisthogastric soft integument with five pairs of setae (JV1–JV3, JV5, ZV2), all on soft integument (anal shield only with three circum-anal setae). Opisthogastric setae mostly short, smooth and needle-like; setae JV5 short or long, thickened and densely pilose to plumose; postanal seta smooth or pilose. Corniculi well sclerotised, relatively slender, with incised apex and subdistal tubercle; setae h1 slightly thickened. Cheliceral digits relatively large, fixed digit with three well developed teeth on proximal masticatory area (two proximal teeth somewhat adjacent). Epistome curved, smooth or with minute serrations. Palps relatively small, palptarsal apotele three-tined. Genu III and tibia III each with two anterolateral and one posterolateral setae, genu IV with two ventral setae, tibia IV with two anterolateral and two posterolateral setae. Tarsi I–IV each with normal empodium and claws.

#### Remarks.

Bai et al. (1995) established their originally monotypic genus *Sinoseius* on the basis of the type species *Sinoseius lobatus* collected in a mammal nest in China. The genus was characterised especially by the form of the dorsal shield setae and three pairs of setae on sternal shield. Karg (2005) and Karg and Schorlemmer (2009) synonymised *Sinoseius* with *Ameroseius* Berlese, 1904. Later, Karg as a collaborating author validated the genus by the description of a congeneric species, *Sinoseius pinnatus*, found in detritus from a straw shed in Finland (Huhta and Karg 2010). Barilo (1986) included one further new species to the genus, collected from soil and wood substrate in Central Asia (Uzbekistan, Tajikistan). The species he named as *Ameroseius fossatus* is here transferred to *Sinoseius*.

The genus *Sinoseius* is considered here to be a valid genus, based on the following combination of female characters (male is unknown): (1) sternal shield with three pairs of setae; (2) unusual chaetotaxy of some leg segments: genu III and tibia III each with two anterolateral and one posterolateral setae (as in *Ameroseius*), genu IV with two ventral setae (not found in *Ameroseius* and *Kleemannia*), tibia IV with two anterolateral and two posterolateral setae (as in *Kleemannia*); (3) dorsal shield setae pinnate; (4) flat curved epistome; (5) cheliceral digits robust, fixed digit with three large teeth; (6) all opisthogastric setae on soft integument; (7) palps relatively small, palptarsal apotele three-tined. Some of these character states occur in other genera of Ameroseiidae, but infrequently, and not in combination. For example, three pairs of setae on sternal shield can be found also in *Kleemannia miranda* sp. n., while specific pinnate setae are typical of *Ameroseius avium* and *Kleemannia bella*.

### Sinoseius fossatus

Taxon classificationAnimaliaMesostigmataAmeroseiidae

(Barilo, 1986) comb. n.

Ameroseius fossatus Barilo, 1986: 1579.

#### Type depository.

Zoological Institute, Russian Academy of Sciences, Saint Persburg, Russia; Department of Invertebrate Zoology, Faculty of Biology, Samarkand University, Uzbekistan.

#### Type locality and habitat.

Uzbekistan, Zeravshan Range (as Zeravshansky Khrebet Mountains), Samarkand, tree hollow of silver poplar (*Populus alba*), in wood detritus (paratypes: also Tajikistan).

#### Remarks.

*Sinoseius fossatus* cannot be distinguished on morphological grounds from *Sinoseius lobatus*, except for the form of JV5. These setae are considerably lengthened and similar in length and form to those on dorsal shield, as depicted in the original illustration of Barilo (1986), and also in the illustration of a *Sinoseius* specimen found in Iran (based on personal communication from Shahrooz Kazemi).

### Sinoseius lobatus

Taxon classificationAnimaliaMesostigmataAmeroseiidae

Bai, Gu & Fang, 1995

Plates 73 , 74 , 75

Sinoseius lobatus Bai, Gu & Fang, 1995: 436.
Sinoseius pinnatus Huhta & Karg, 2010: 335. **Syn. n.**
Sinoseius pinnatus . — Fenďa and Lukáš 2014: 174.

#### Type depository.

Of *Sinoseius lobatus* – Institute of Endemic Disease Control, Ningxia Autonomous Region, Yinchuan, China; of *Sinoseius pinnatus* – Zoological Museum, University of Turku, Finland; Senckenberg Museum für Naturkunde, Görlitz, Germany; Zoological Museum, University of Helsinki, Finland.

#### Type locality and habitat.

Of *Sinoseius lobatus* – China, Ningxia Autonomous Region, Haiyuan County, on long-tailed dwarf hamster, *Cricetulus longicaudatus* (Mammalia, Rodentia, Cricetidae); of *Sinoseius pinnatus* – Finland, Parainen, Sunnaberg, bottom of straw shed.

#### Comparative material.

Finland: 2 ♀♀ (ZMT: ACA.MES.FIN.3.654, holotype and paratype) – 10. 10. 1982, Parainen, Sunnaberg, bottom of straw shed, leg. P. T. Lehtinen (labelled *Sinoseius pinnatus*).

#### Published and verified material from Slovakia.

Čierna Hora Mts.: Veľký Folkmar Village, Ružín Dam; Veľký Folkmar Village, Veľká Hoľa Cave (Fenďa and Lukáš 2014, cited as *Sinoseius pinnatus*).

#### Remarks.

The first European finding of the genus *Sinoseius* was that of Huhta and Karg (2010) from Finland (bottom of straw shed and grass in garden of old farm). Fenďa and Lukáš (2014) found the species in a frozen nest of *Sitta europaea* Linnaeus, 1758 (Aves, Passeriformes), in a nest box, and in a soil sample from the dysphotic zone of a cave. I have compared the holotype and one paratype of *Sinoseius pinnatus* with the specimens reported from Slovakia by Fenďa and Lukáš (2014), and found that they are clearly conspecific. According to Huhta and Karg (2010), *Sinoseius lobatus* and *S. pinnatus* can be distinguished by the features presented as follows: in *S. lobatus*, dorsal setae „remarkably long“, j5 = j5–j6, j6 > j6–J2, tines of dorsal setae strong (depicted seven pairs of tines), anal shield distinctly wider than long (length/width = 2:3), corniculi bifid; in *S. pinnatus*, dorsal setae „moderate“, j5 = 2/3 × j5–j6, j6 < j6–J2, tines of dorsal setae very thin (depicted up to 19 instead of 9–10 pairs of tines), anal shield only a little wider than long, corniculi distally trifid. It is clear that the distinctions made in the original descriptions and differential diagnosis of *S. pinnatus* by Huhta and Karg (2010) are based on characteristics that are misinterpreted (form of setae and corniculi) or vary considerably (relative length of dorsal setae, proportion of anal shield), and I do not hesitate to propose the synonymy of both mentioned species, although no types of *S. lobatus* were examined in this study. When compared the Slovak specimens of *Sinoseius* with quite adequate original illustrations of *S. lobatus* from China, it was not possible to find reliable distinguishing characters between them.

### Unrecognizable species (*species inquirendae*)

The following species are temporarily relegated to the species „*incertae sedis*” because the descriptions do not include information about some important characters needful for their specific identification. If some of them are correctly placed in Ameroseiidae, then they cannot be included in the above classification. I dare to say that many species in this list can be excluded from Ameroseiidae after their revision (if their type specimens are available for a study).

### Actinoseius terrificans

Taxon classificationAnimaliaMesostigmataAmeroseiidae

Berlese, 1916

Epicriopsis (Actinoseius) terrificans Berlese, 1916a: 49.

#### Type depository.

Istituto Sperimentale per la Zoologia Agraria, Firenze, Italy.

#### Type locality and habitat.

Argentina, La Plata, habitat not stated.

#### Remarks.

*Actinoseius* Berlese, 1916 was originally introduced as a subgenus of the genus *Epicriopsis* Berlese, 1916, considering Epicriopsis (Actinoseius) terrificans, then described, as the type species. Castagnoli and Pegazzano (1985) did not include E. (A.) terrificans in their Catalogue of the Berlese Acaroteca. Hallan (2005) included *Actinoseius* in his compiled list of pachylaelapid genera. Original description of this taxon and of its type species is absolutely useless for species and subgenus recognition. Thus, the reason leading Hallan (2005) to include it in Pachylaelapidae is not clear. Most likely, this species could be based on a specimen mounted onto slide No. 162/9 of the Berlese Collection in Florence, and labelled *Ameroseius terrificans* (nomen nudum). That specimen and, according to the original description, the type specimen of E. (A.) terrificans were collected in La Plata, Argentina.

### Ameroseius epicrioides

Taxon classificationAnimaliaMesostigmataAmeroseiidae

Berlese, 1916

Ameroseius epicrioides Berlese, 1916a: 46.
Ameroseius epicrioides . — Castagnoli and Pegazzano 1985: 130.

#### Type depository.

Istituto Sperimentale per la Zoologia Agraria, Firenze, Italy.

#### Type locality and habitat.

Argentina, Rio Santiago, La Plata, under bark of tree.

### Ameroseius geometricus

Taxon classificationAnimaliaMesostigmataAmeroseiidae

Berlese, 1910

Ameroseius geometricus Berlese, 1910b: 254.
Ameroseius geometricus . — Berlese 1916a: 34; Castagnoli and Pegazzano 1985: 159.

#### Type depository.

Istituto Sperimentale per la Zoologia Agraria, Firenze, Italy.

#### Type locality and habitat.

Indonesia, Java, on flat-faced longhorn, *Thysia wallichii* (as *Tyckia Walliki*) (Coleoptera, Cerambycidae).

### Ameroseius hypogaeus

Taxon classificationAnimaliaMesostigmataAmeroseiidae

Berlese, 1920

Ameroseius hypogaeus Berlese, 1920: 170.
Ameroseius hypogaeus . — Castagnoli and Pegazzano 1985: 190.

#### Type depository.

Istituto Sperimentale per la Zoologia Agraria, Firenze, Italy (holotype not designated).

#### Type locality and habitat.

Italy, Sardinia, Asuni, in nest European water vole, *Arvicola amphibius* (formerly *Arvicola terrestris*).

#### Comparative material.

Italy: 1 ♀ (ISZA: 208/33, syntype) – Asuni, Sardegna, nidi di topi campagnoli, leg. Krausse.

#### Remarks.

None of the two slides in the Berlese Collection bears a type designation, but without doubt they belong to the original type series of Berlese. Unfortunately, a female on slide 208/33 is unsuitable for study and will require remounting for definitive study, whereas the female on slide 210/39 must be lost because I was unable to find any specimen or dissected structures on that slide (both slides are opaque and essentially unusable).

### Ameroseius ingens

Taxon classificationAnimaliaMesostigmataAmeroseiidae

Hull, 1918

Ameroseius ingens Hull, 1918: 64. Type locality: British Isles (England).

#### Type depository.

Not stated.

#### Type locality and habitat.

United Kingdom, England, West Allendale, in moss.

### Ameroseius serruliger

Taxon classificationAnimaliaMesostigmataAmeroseiidae

Berlese, 1916

Ameroseius serruliger Berlese, 1916b: 171.
Ameroseius serruliger . — Castagnoli and Pegazzano 1985: 376.

#### Type depository.

Istituto Sperimentale per la Zoologia Agraria, Firenze, Italy (holotype not designated).

#### Type locality and habitat.

Argentina, La Plata, habitat stated.

### Ameroseius sulcatus

Taxon classificationAnimaliaMesostigmataAmeroseiidae

Hull, 1925

Ameroseius sulcatus Hull, 1925: 207.

#### Type depository.

Not stated.

#### Type locality and habitat.

United Kingdom, England, Ambleside, habitat stated.

#### Remarks.

Hull‘s description and illustration of this species are not detailed enough to allow it to be correctly recognised and classified in a genus with any confidence. The illustrated specimen resembles a *Cheiroseius* species.

### Epicriopsis siculus

Taxon classificationAnimaliaMesostigmataAmeroseiidae

Berlese, 1916

Epicriopsis horrida var. sicula Berlese, 1916a: 48.

#### Type depository.

Istituto Sperimentale per la Zoologia Agraria, Firenze, Italy.

#### Type locality and habitat.

Italy, Sicily, Palermo, in moss.

#### Comparative material.

Italy: 2 ♀♀ (ISZA: 99/9, 99/10, syntypes) – Palermo, musco (labelled *Epicrius mollis*).

#### Remarks.

I examined two Berlese slides, each bearing one female of *Epicriopsis* collected from moss in Palermo, Sicily, and labelled *Epicrius mollis*. As stated by Castagnoli and Pegazzano (1985), they could most likely represent unlabelled and undesignated type specimens of Epicriopsis horrida var. sicula. Unfortunately, both female specimens are absolutely unsuitable for study due to the darkening of the mounting medium.

### Kleemannia cristata

Taxon classificationAnimaliaMesostigmataAmeroseiidae

(Hull, 1925)

Lasioseius cristatus Hull, 1925: 206.
Ameroseius (? Kleemania) (sic) cristatus. — Athias-Henriot 1959: 192.

#### Type depository.

Not stated.

#### Type locality and habitat.

United Kingdom, Scotland, Midlothian, Hunter’s Tryst, habitat stated.

#### Remarks.

Hull‘s description and illustration of this species are not detailed enough to allow it to be identified to the generic level with any confidence.

### Species excluded from Ameroseiidae

Below is a list of species that have been incorrectly classified in Ameroseiidae at some time, but are now placed in other families. The species are listed in alphabetical order of original species names, with current valid name and systematic placement of each species. The species transferred to Ascidae, Blattisociidae or Melicharidae were checked using recent catalogue for these families by Moraes et al. (2016) to confirm their current systematic placement.

***Ameroseius bispinosus*** Berlese, 1910b: 253. Type locality: Italy – Sicily.

= *Zerconopsis remiger* (Kramer, 1876), Ascidae — synonymy by Evans and Hyatt (1960)

***Ameroseius borealis*** Berlese, 1904: 259. Type locality: Norway.

= *Cheiroseius borealis* (Berlese, 1904), Blattisociidae — transferred by Westerboer (1963)

***Ameroseius crassipes*** Berlese, 1910c: 370. Type locality: Australia.

= a species of unspecified genus of Ologamasidae — by Halliday (1997)

**Remarks.** Berlese (1916a) transferred this species from *Ameroseius* to the subgenus Lasioseius (Leioseius). Bernhard (1963) did not consider *Ameroseius crassipes* to belong to *Leioseius*, but probably to *Ameroseius*. Halliday (1997) transferred it to Ologamasidae, based on his personal communication with Evert E. Lindquist who examined the type material of *A. crassipes* in the Berlese Collection in Florence.

***Ameroseius favosus*** Berlese, 1910b: 254. Type locality: Tasmania.

= a species of an unspecified genus of Phytoseiidae — by Halliday (1997), based on his personal communication with Evert E. Lindquist

**Remarks.** Berlese (1916a) transferred this species from *Ameroseius* to the subgenus Lasioseius (Leioseius). Bernhard (1963) did not consider *Ameroseius favosus* to belong to *Leioseius*. Halliday (1997) transferred it to Phytoseiidae, based on his personal communication with Evert E. Lindquist who examined the type material of *A. favosus* in the Berlese Collection in Florence.

***Ameroseius flagellatus*** Berlese, 1910b: 254. Type locality: South-East Asia.

= *Lasioseius flagellatus* (Berlese, 1910), Blattisociidae — transferred by Berlese (1916a)

**Remarks.** Berlese (1916a) transferred this species from *Ameroseius* to the genus *Lasioseius* s. str. Bernhard (1963) did not consider *Ameroseius flagellatus* to belong to *Lasioseius*.

***Ameroseius imitans*** Berlese, 1910c: 370. Type locality: India.

= *Lasioseius imitans* (Berlese, 1910), Blattisociidae — transferred by Westerboer (1963)

***Ameroseius italicus*** Berlese, 1905b: 234. Type locality: Italy.

= *Platyseius italicus* (Berlese, 1905), Blattisociidae — transferred by Buitendijk (1945) and Lindquist and Evans (1965)

***Ameroseius jacobsoni*** Berlese, 1910b: 258. Type locality: Indonesia – Java.

= *Lasioseius jacobsoni* (Berlese, 1910), Blattisociidae — transferred by Berlese (1916a)

***Ameroseius laelaptoides*** Berlese, 1904: 258. Type locality: Italy.

= possibly a species of the genus *Cheiroseius* Berlese, 1916, Blattisociidae — see remarks below

≠ *Cheiroseius laelaptoides* (Berlese, 1887)

**Remarks.** According to Castagnoli and Pegazzano (1985), in his unpublished manuscript “Catalogue of the Synonyms” Berlese intended to rename this species as Lasioseius (Lasioseius) hypoaspidiformis because his older species *Epicrius laelaptoides* Berlese, 1887 had been earlier transferred to the same subgenus, Lasioseius (Lasioseius) (Berlese 1916a). There are three slides and three specimens (2 ♀♀, 1 ♂) of this species in the Berlese Collection. Two of them (13/24, 13/25) are labelled „tipico”, therefore they are syntypes, and apparently belong to the original series of Berlese. Unfortunately, the male and female labelled „tipico” are unsuitable for study. Another female specimen (168/36) available in the Berlese Collection is in relatively good condition, it belongs to the genus *Cheiroseius* Berlese, 1916.

***Ameroseius minusculus*** Berlese, 1905b: 235. Type locality: Italy.

= *Leioseius minusculus* (Berlese, 1905), Ascidae — transferred by Berlese (1916a)

***Ameroseius oviforme*** Schweizer, 1949: 44. Type locality: Switzerland.

= *Iphidozercon gibbus* (Berlese, 1903), Ascidae — synonymy by Lindquist (1964)

***Ameroseius pseudocometa*** Schweizer, 1922: 42. Type locality: Switzerland.

= *Aceoseius muricatus* (C. L. Koch, 1839), Blattisociidae — synonymy by Sellnick (1941)

***Ameroseius reticulatus*** Berlese, 1905a: 171. Type locality: Indonesia – Java.

= Lasioseius (Zercoseius) reticulatus (Berlese, 1905), Blattisociidae — transferred by Berlese (1916a)

≠ *Lasioseius reticulatus* Bhattacharyya, 1968

≠ *Kleemannia reticulata* Kruger & Loots, 1980

**Remarks.** Morphological features of this species remain unknown although it could be considered to be a member of *Lasioseius* Berlese, 1916 or *Zercoseius* Berlese, 1916, based on later opinion of Berlese (1916a).

***Ameroseius tuberculiger*** Berlese, 1916a: 47. Type locality: USA, Missouri, Columbia.

= *Lasioseius tuberculiger* (Berlese, 1916), Blattisociidae — transferred by Lindquist (1964)

***Ameroseius zerconiformis*** Berlese, 1905b: 234. Type locality: Italy.

= *Zercoseius spathuliger* (Leonardi, 1899), Blattisociidae — synonymy by Berlese (1910a) and Evans, 1958

### *Nomina nuda*

Castagnoli and Pegazzano (1985) referred to seven species in the Berlese Acaroteca as members of the genus *Ameroseius* which were apparently never described by Berlese in any of his published papers, and which can be considered to be *nomina nuda*:

*Ameroseius longiscutatus* Berlese, *nomen nudum*

*Ameroseius indusiatus* Berlese, *nomen nudum*

*Ameroseius molliculus* Berlese, *nomen nudum*

*Ameroseius planus* Berlese, *nomen nudum*

*Ameroseius ribagai* Berlese, *nomen nudum*

*Ameroseius simplex* Berlese, *nomen nudum*

*Ameroseius terrificans* Berlese, *nomen nudum*

### Unavailable name

*Ameroseius ornatus* Postner, 1951: 97 (unavailable name in unpublished thesis).

≠ *Ameroseius ornatus* Womersley, 1956

## Synopsis

This catalogue of the family Ameroseiidae includes 12 genera and 138 valid species. Another nine species are temporarily relegated to the unrecognizable species (*species inquirendae*), because the descriptions do not include information about important characters required for identification, and are here regarded as of unknown systematic position. In summary, altogether 206 named species (including 37 synonyms, 15 species previously excluded from the family, and seven “nomina nuda”) are mentioned in this paper. The most diverse genera are *Ameroseius*, *Kleemannia* and *Neocypholaelaps*, which include 50 (36%), 28 (20%) and 22 (16%) species, respectively. Two genera are monotypic, *Afrocypholaelaps* and *Pseudoameroseius* gen. n.

The objective of this paper was not to present a detailed historical-taxonomic review of the family Ameroseiidae, but to analyse and revise the current state of knowledge of the family and draw attention to some unresolved problems. This review and the identification keys should provide a useful basis for identification of world genera and the species from Europe in future taxonomic research. However, the research work of the ameroseiid mites is apparently far from complete and should continue, especially with detailed study of taxa from tropical areas of the world.

## Taxonomic summary

(1) In this review, the family Ameroseiidae includes 138 valid species of the following 12 genera:

– *Afrocypholaelaps* Elsen, 1972

– *Ameroseiella* Bregetova, 1977

– *Ameroseius* Berlese, 1904

– *Asperolaelaps* Womersley, 1956

– *Brontispalaelaps* Womersley, 1956

– *Epicriopsis* Berlese, 1916

– *Hattena* Domrow, 1963

– *Kleemannia* Oudemans, 1930

– *Neocypholaelaps* Vitzthum, 1942

– *Pseudoameroseius* gen. n.

– *Sertitympanum* Elsen & Whitaker, 1985

– *Sinoseius* Bai & Gu, 1995

(2) The following new genus is proposed:

– *Pseudoameroseius* gen. n., based on *Ameroseius michaelangeli* Moraza, 2006

(3) The following five taxa are resurrected:

– *Ameroseiella* Bregetova, 1977

– *Asperolaelaps* Womersley, 1956

– *Kleemannia* Oudemans, 1930

– *Sinoseius* Bai & Gu, 1995

– Kleemannia (Primoseius) Womersley, 1956

(4) The following three new species are described and illustrated:

– *Ameroseius renatae* sp. n. (from Slovakia)

– *Kleemannia dolichochaeta* sp. n. (from Spain)

– *Kleemannia miranda* sp. n. (from the U.S.A.)

(5) A replacement name is proposed for a junior homonym:

– *Ameroseius womersleyi* Mašán, replacement name for *Ameroseius ornatus* Womersley, 1956, junior secondary homonym of *Cornubia ornata* Turk, 1943 [now an accepted synonym of *Ameroseius corbiculus* (Sowerby, 1806)].

(6) The following 27 species are newly relegated into synonymy:

– *Afrocypholaelaps analicullus* Ho, Ma, Wang & Severinghaus, 2010, a synonym of *Afrocypholaelaps africanus* (Evans, 1963)

– *Afrocypholaelaps ranomafanaensis* Haitlinger, 1987, a synonym of *Afrocypholaelaps africanus* (Evans, 1963)

– *Ameroseius apodius* Karg, 1971, a synonym of *Ameroseiella macrochelae* (Westerboer, 1963)

– *Ameroseius bregetovae* Livshits & Mitrofanov, 1975, a synonym of *Neocypholaelaps favus* Ishikawa, 1968

– *Ameroseius chinensis* Khalili-Moghadam & Saboori, 2016, a synonym of *Ameroseius guyimingi* Ma, 1997

– *Ameroseius crassisetosus* Ye & Ma, 1993, a synonym of *Ameroseius corbiculus* (Sowerby, 1806)

– *Ameroseius dubitatus* Berlese, 1918, a synonym of *Kleemannia plumosa* (Oudemans, 1902)

– *Ameroseius eumorphus* Bregetova, 1977, a synonym of *Kleemannia pseudoplumosa* (Rack, 1972)

– *Ameroseius fimetorum* Karg, 1971, a synonym of *Kleemannia tenella* (Berlese, 1916)

– *Ameroseius gilarovi* Petrova, 1986, a synonym of *Kleemannia plumigera* Oudemans, 1930

– *Ameroseius imparsetosus* Westerboer, 1963, a synonym of *Ameroseius georgei* (Turk, 1943)

– *Ameroseius lanatus* Solomon, 1969, a synonym of *Kleemannia tenella* (Berlese, 1916)

– *Ameroseius lanceosetis* Livshits & Mitrofanov, 1975, a synonym of *Kleemannia pavida* (C. L. Koch, 1839)

– *Ameroseius marginalis* Fan & Li, 1993, a synonym of *Kleemannia insignis* (Bernhard, 1963)

– *Ameroseius norvegicus* Narita, Abduch & Moraes, 2015, a synonym of *Ameroseius corbiculus* (Sowerby, 1806)

– *Ameroseius pseudofurcatus* Livshits & Mitrofanov, 1975, a synonym of *Ameroseius furcatus* Karg, 1971

– *Ameroseius qinghaiensis* Li & Yang, 2000, a synonym of *Ameroseius corbiculus* (Sowerby, 1806)

– *Ameroseius sichuanensis* Fan & Li, 1993, a synonym of *Kleemannia insignis* (Bernhard, 1963)

– *Ameroseius stramenis* Karg, 1976, a synonym of *Kleemannia delicata* (Berlese, 1918)

– *Epicriopsis baloghi* Kandil, 1978, a synonym of *Epicriopsis palustris* Karg, 1971

– *Epicriopsis langei* Livshits & Mitrofanov, 1975, a synonym of *Epicriopsis palustris* Karg, 1971

– *Epicriopsis rivus* Karg, 1971, a synonym of *Epicriopsis mirabilis* Willmann, 1956

– *Kleemannia potchefstroomensis* Kruger & Loots, 1980, a synonym of *Kleemannia pseudoplumosa* (Rack, 1972)

– Lasioseius (Lasioseius) gracilis Halbert, 1923, a synonym of *Kleemannia delicata* (Berlese, 1918)

– *Neocypholaelaps ewae* Haitlinger, 1987, a synonym of *Neocypholaelaps indicus* Evans, 1963

– *Neocypholaelaps lindquisti* Prasad, 1968, a synonym of *Afrocypholaelaps africanus* (Evans, 1963)

– *Sinoseius pinnatus* Huhta & Karg, 2010, a synonym of *Sinoseius lobatus* Bai, Gu & Fang, 1995

(7) The following 23 new combinations are proposed. Many of these changes have not been discussed explicitly in the text, but are implied by my revised definition of individual ameroseiid genera:

– *Ameroseiella macrochelae* (Westerboer, 1963), previously *Ameroseius macrochelae* Westerboer, 1963

– *Asperolaelaps sextuberculi* (Karg, 1996), previously *Ameroseius sextuberculi* Karg, 1996

– *Hattena senaria* (Allred, 1970), previously *Ameroseius senarius* Allred, 1970

– *Kleemannia bella* (Barilo, 1987), previously *Ameroseius bellus* Barilo, 1987

– *Kleemannia bisetae* (Karg, 1994), previously *Ameroseius bisetae* Karg, 1994

– *Kleemannia curvata* (Gu, Wang & Bai, 1989), previously *Ameroseius curvatus* Gu, Wang & Bai, 1989

– *Kleemannia delicata* (Berlese, 1918), previously *Ameroseius delicatus* Berlese, 1918

– *Kleemannia dipankari* (Bhattacharyya, 2004), previously *Ameroseius dipankari* Bhattacharyya, 2004

– *Kleemannia elegans* (Bernhard, 1963), previously *Ameroseius elegans* Bernhard, 1963

– *Kleemannia guyimingi* (Ma, 1997), previously *Ameroseius guyimingi* Ma, 1997

– *Kleemannia insignis* (Bernhard, 1963), previously *Ameroseius insignis* Bernhard, 1963

– *Kleemannia longisetosa* (Ye & Ma, 1993), previously *Ameroseius longisetosus* Ye & Ma, 1993

– *Kleemannia mineiro* (Narita, Bernardi & Moraes, 2013), previously *Ameroseius mineiro* Narita, Bernardi & Moraes, 2013

– *Kleemannia multus* (Gu, Wang & Bai, 1989), previously *Ameroseius multus* Gu, Wang & Bai, 1989

– *Kleemannia pennata* (Fox, 1949), previously *Ameroseius pennatus* (Fox, 1949)

– *Kleemannia plumosoides* (Gu, Wang & Bai, 1989), previously *Ameroseius plumosoides* Gu, Wang & Bai, 1989

– *Kleemannia pseudoplumosa* (Rack, 1972), previously *Ameroseius pseudoplumosus* Rack, 1972

– *Kleemannia tenella* (Berlese, 1916), previously *Ameroseius tenellus* Berlese, 1916

– *Neocypholaelaps wilsoni* (Allred, 1970), previously *Ameroseius wilsoni* Allred, 1970

– *Pseudoameroseius michaelangeli* (Moraza, 2006), previously *Ameroseius michaelangeli* Moraza, 2006

– *Sertitympanum nodosum* (Sheals, 1962), previously *Kleemannia nodosa* Sheals, 1962

– *Sertitympanum zaheri* (El-Badry, Nasr & Hafez, 1979), previously *Kleemannia zaheri* El-Badry, Nasr & Hafez, 1979

– *Sinoseius fossatus* (Barilo, 1986), previously *Ameroseius fossatus* Barilo, 1986

(8) The following species is removed from synonymy:

– *Cornubia georgei* Turk, 1943 (nom. n. pro *Epicrius canestrinii* Haller, 1881 sensu George, 1906), is not a synonym of *Ameroseius corbiculus* (Sowerby, 1806), proposed by Turk (1953).

(9) The following seven records are considered to be misidentifications:

– *Ameroseius magnisetosus* (Ishikawa, 1972) sensu Bregetova (1977), is a misidentification of *Kleemannia guyimingi* (Ma, 1997)

– *Epicriopsis horridus* (Kramer, 1876) sensu Evans & Till (1979), is a misidentification of *Epicriopsis palustris* Karg, 1971

– *Epicriopsis horridus* (Kramer, 1876) sensu Iavorschi (1995), is a misidentification of *Epicriopsis palustris* Karg, 1971

– *Kleemannia delicata* (Berlese, 1918) sensu Schweizer (1961), is a misidentification of *Kleemannia plumigera* Oudemans, 1930

– *Kleemannia plumosa* (Oudemans, 1902) sensu Zaher (1986), is a misidentification of *Kleemannia pseudoplumosa* (Rack, 1972)

– *Neocypholaelaps apicola* Delfinado-Baker & Baker, 1983 sensu Kontschán et al. (2015), is a misidentification of *Neocypholaelaps favus* Ishikawa, 1968

– *Primoseius macauleyi* (Hughes, 1948) sensu Womersley (1956), is a misidentification of *Kleemannia pseudoplumosa* (Rack, 1972)

(10) The following seven new species-groups are introduced:

– *Ameroseius magnisetosus* group

– *Ameroseius fungicola* group

– *Hattena senaria* group

– *Hattena panopla* group

– *Hattena dalyi* group

– *Hattena erosa* group

– *Hattena cometis* group

(11) The following nine species are regarded as unrecocnizable species (*species inquirendae*):

– *Actinoseius terrificans* Berlese, 1916

– *Ameroseius epicrioides* Berlese, 1916

– *Ameroseius geometricus* Berlese, 1910

– *Ameroseius hypogaeus* Berlese, 1920

– *Ameroseius ingens* Hull, 1918

– *Ameroseius serruliger* Berlese, 1916

– *Ameroseius sulcatus* Hull, 1925

– *Epicriopsis siculus* Berlese, 1916

– *Kleemannia cristata* (Hull, 1925)

(12) New keys are given for identification of 37 species belonging to eight genera found in Europe: *Ameroseiella*, *Ameroseius*, *Epicriopsis*, *Kleemannia*, *Neocypholaelaps*, *Pseudoameroseius* gen. n., *Sertitympanum* and *Sinoseius*.

(13) The following four taxa are recorded from Europe for the first time:

– *Sertitympanum* Elsen & Whitaker, 1985

– *Sertitympanum nodosum* (Sheals, 1962)

– *Kleemannia kosi* El-Badry, Nasr & Hafez, 1979

– *Kleemannia parplumosa* Nasr & Abou-Awad, 1986

(14) Altogether six genera and 27 species of Ameroseiidae have been found in Slovakia. Among them, there are nine species recorded from Slovakia for the first time.

– *Ameroseiella macrochelae* (Westerboer, 1963)

– *Ameroseius callosus* Mašán, 1998

– *Ameroseius cavernosus* Westerboer, 1963 (first record)

– *Ameroseius corbiculus* (Sowerby, 1806)

– *Ameroseius corniculus* Karg, 1971

– *Ameroseius fungicola* Mašán, 1998

– *Ameroseius furcatus* Karg, 1971

– *Ameroseius georgei* (Turk, 1943) (previously cited as *Ameroseius imparsetosus*)

– *Ameroseius lidiae* Bregetova, 1977

– *Ameroseius longitrichus* Hirschmann, 1963

– *Ameroseius renatae* sp. n. (first record)

– *Ameroseius sculptilis* Berlese, 1916

– *Ameroseius ulmi* Hirschmann, 1963 (first record)

– *Epicriopsis horridus* (Kramer, 1876)

– *Epicriopsis hungaricus* Kandil, 1978 (first record)

– *Epicriopsis mirabilis* Willmann, 1956 (previously cited as *Epicriopsis rivus*)

– *Epicriopsis palustris* Karg, 1971

– *Kleemannia delicata* (Berlese, 1918) (first record)

– *Kleemannia insignis* (Bernhard, 1963) (first record)

– *Kleemannia pavida* (C. L. Koch, 1839) (first record)

– *Kleemannia plumea* Oudemans, 1930

– *Kleemannia plumigera* Oudemans, 1930

– *Kleemannia plumosa* (Oudemans, 1902)

– *Kleemannia pseudoplumosa* (Rack, 1972) (first record)

– *Kleemannia tenella* (Berlese, 1916) (first record)

– *Neocypholaelaps favus* Ishikawa, 1968

– *Sinoseius lobatus* Bai, Gu & Fang, 1995 (previously cited as *Sinoseius pinnatus*)

## Figures and Tables

**Plate 3.**
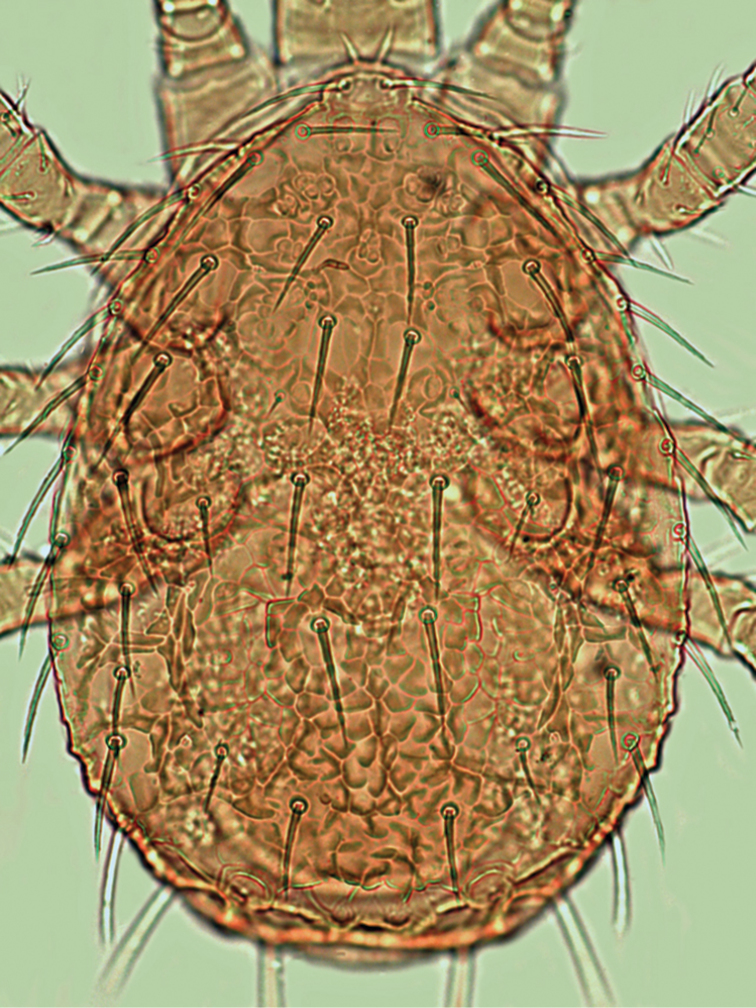
*Ameroseius callosus*, female, dorsal idiosoma.

**Plate 4.**
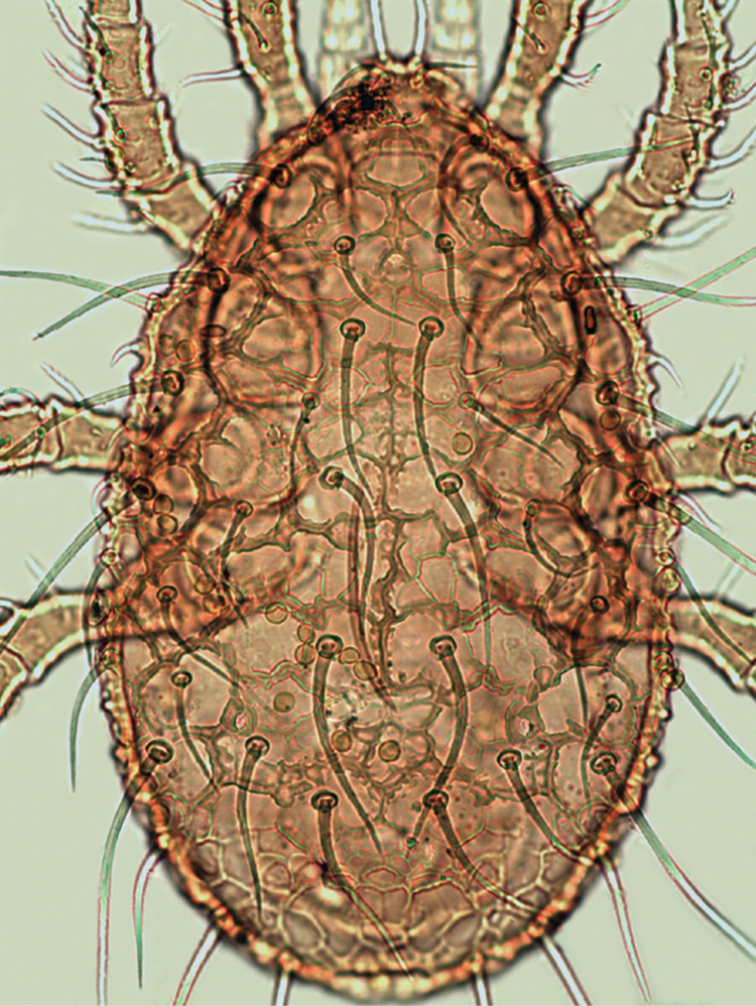
*Ameroseius cavernosus*, female, dorsal idiosoma.

**Plate 5.**
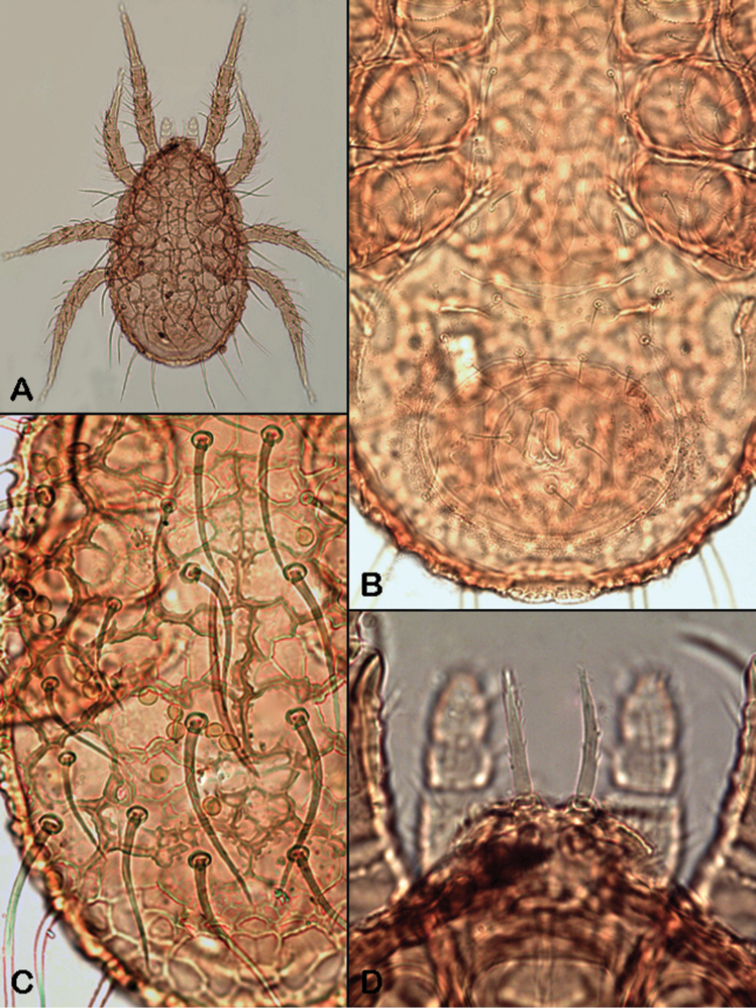
*Ameroseius cavernosus*, female. **A** Dorsal idiosoma with legs **B** Epigynal and anal shield **C** Dorsal shield sculpture, posterolateral view **D** Setae j1.

**Plate 6.**
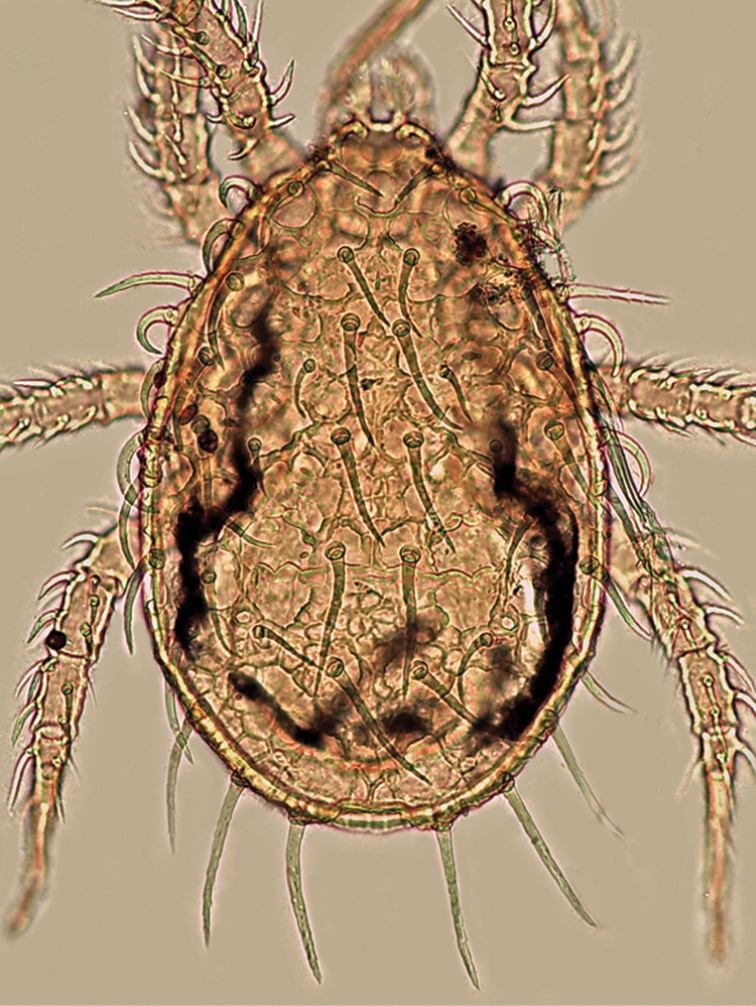
*Ameroseius corbiculus*, female, dorsal idiosoma.

**Plate 7.**
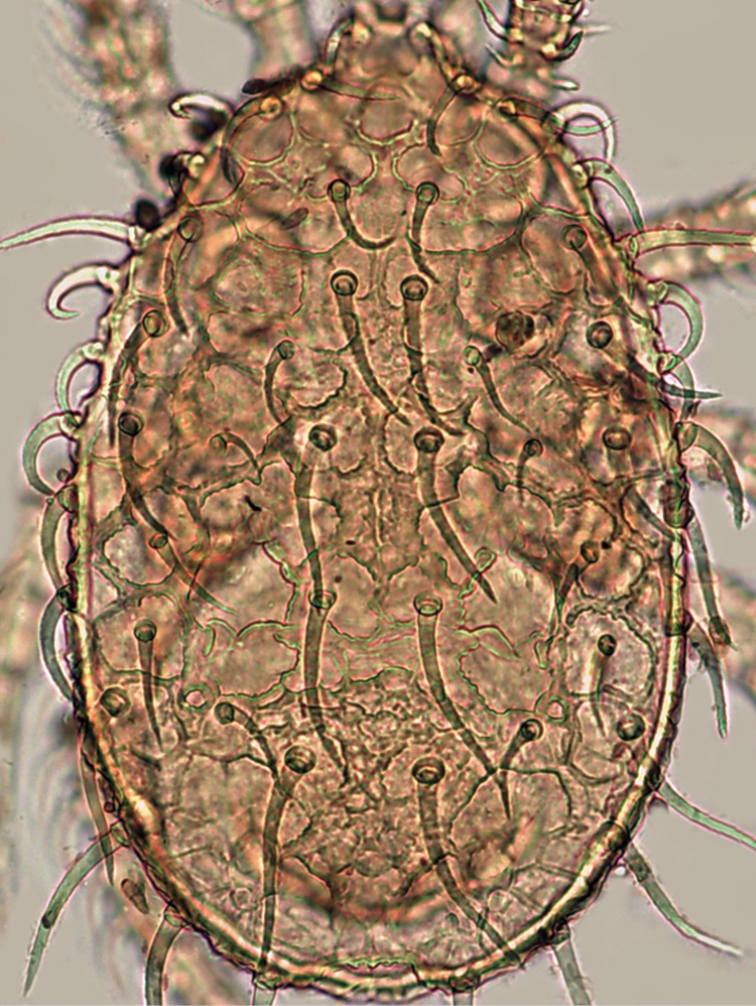
*Ameroseius corbiculus*, female, dorsal idiosoma.

**Plate 8.**
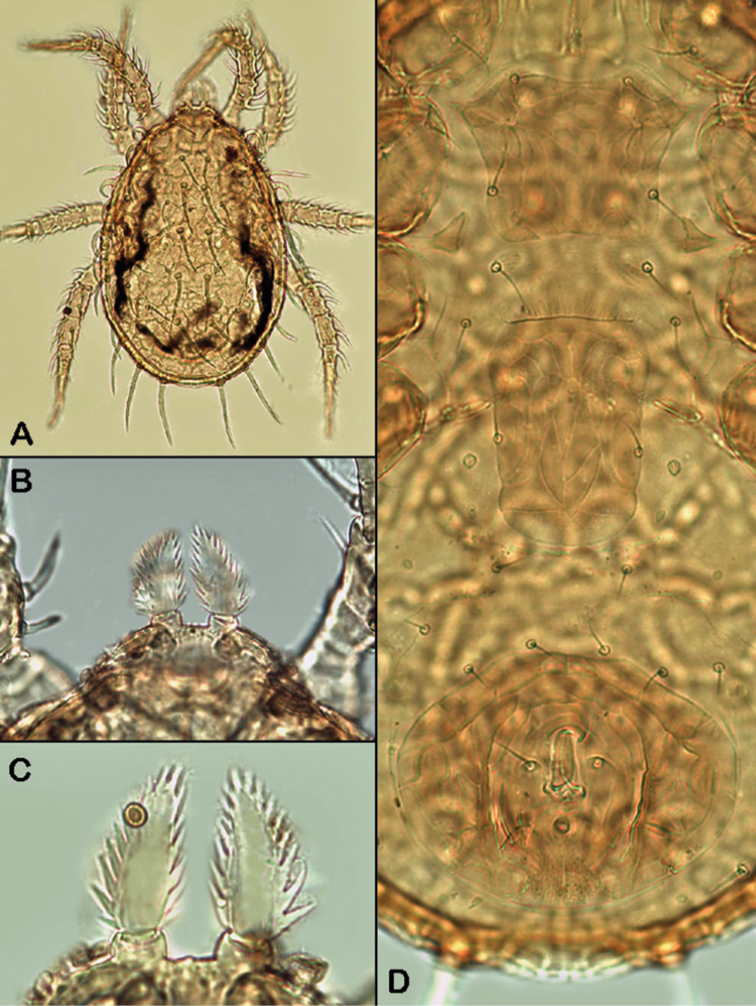
*Ameroseius corbiculus*, female. **A** Dorsal idiosoma with legs **B–C** Setae j1, variant forms **D** Ventral shields.

**Plate 9.**
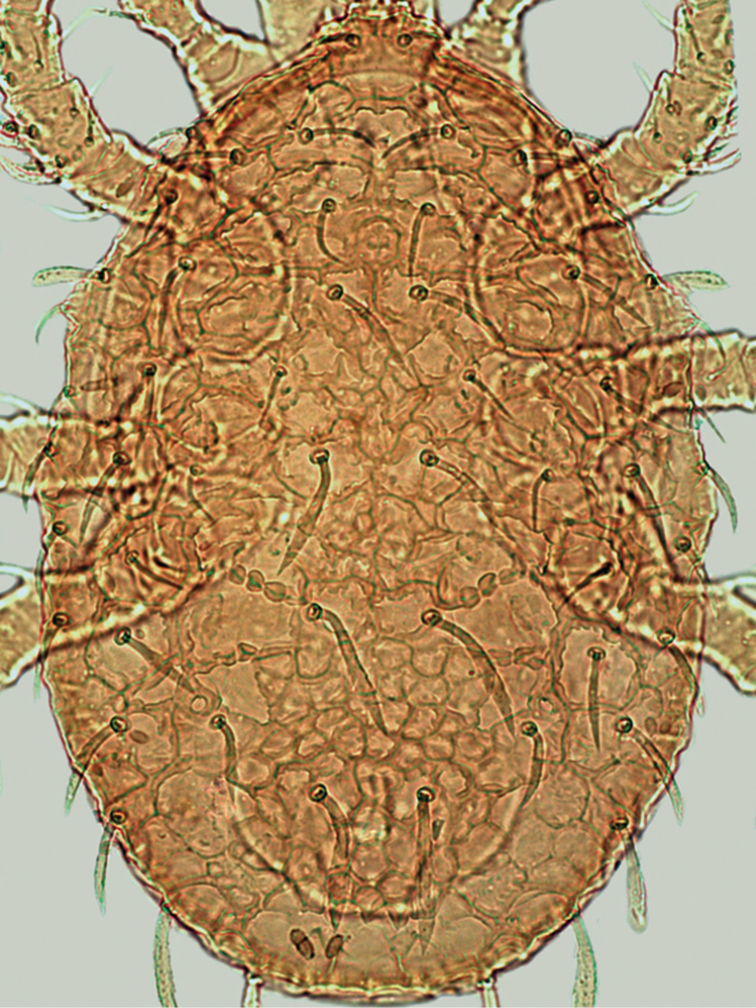
*Ameroseius corniculus*, female, dorsal idiosoma.

**Plate 10.**
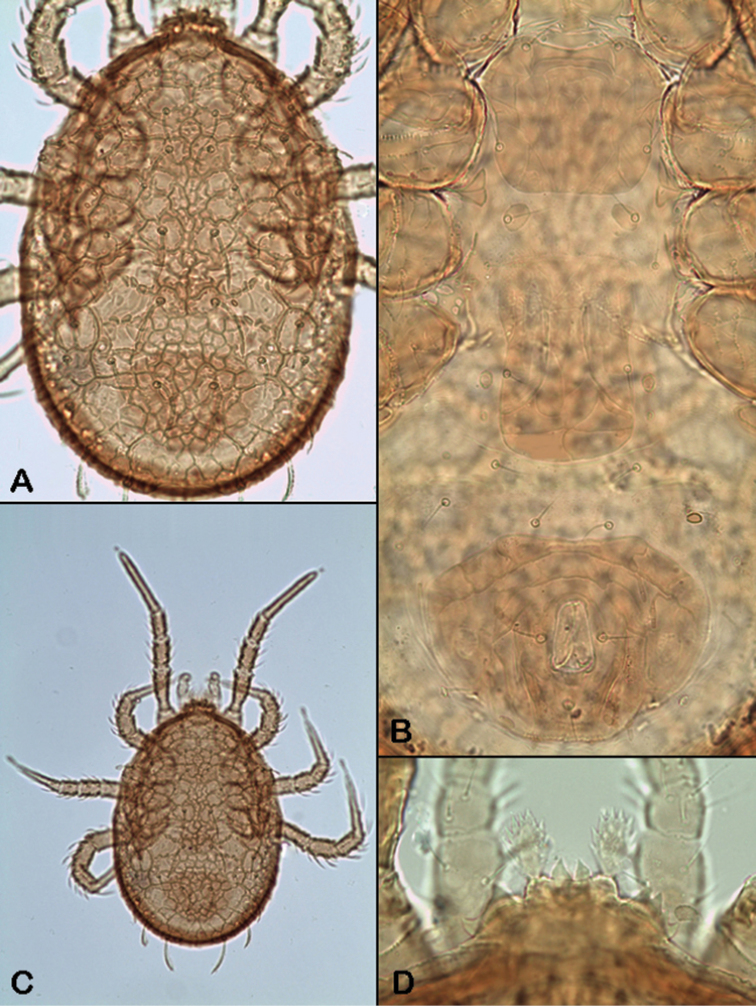
*Ameroseius corniculus*, female. **A** Dorsal idiosoma **B** Ventral shields **C** Dorsal idiosoma with legs **D** Setae j1 and dentation of vertex.

**Plate 11.**
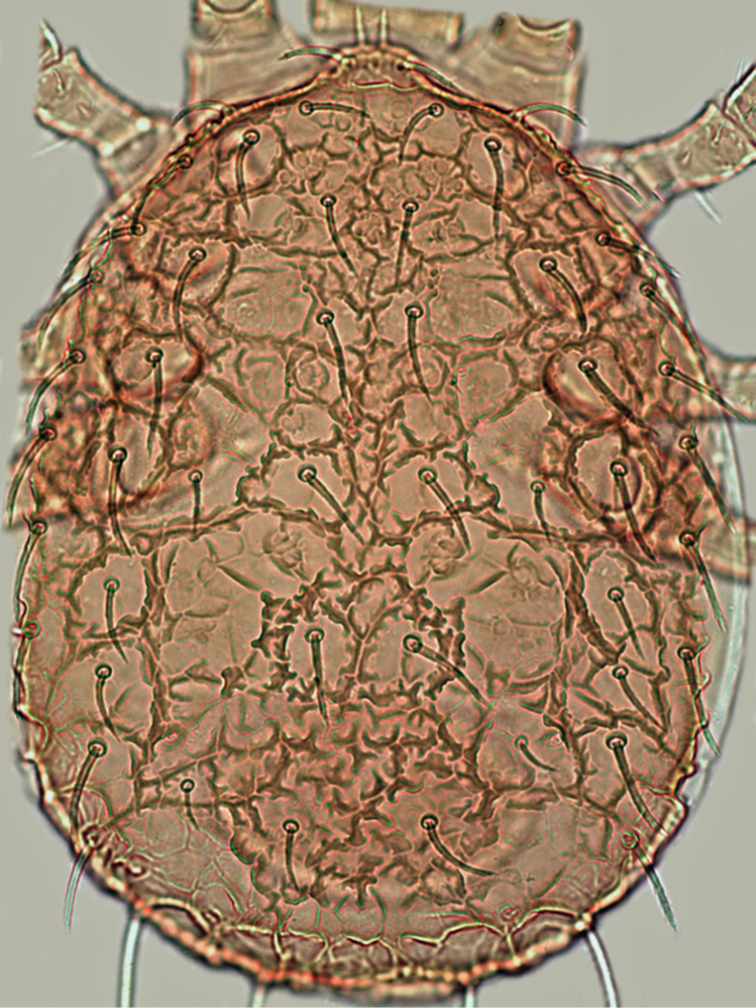
*Ameroseius fungicola*, female, dorsal idiosoma.

**Plate 12.**
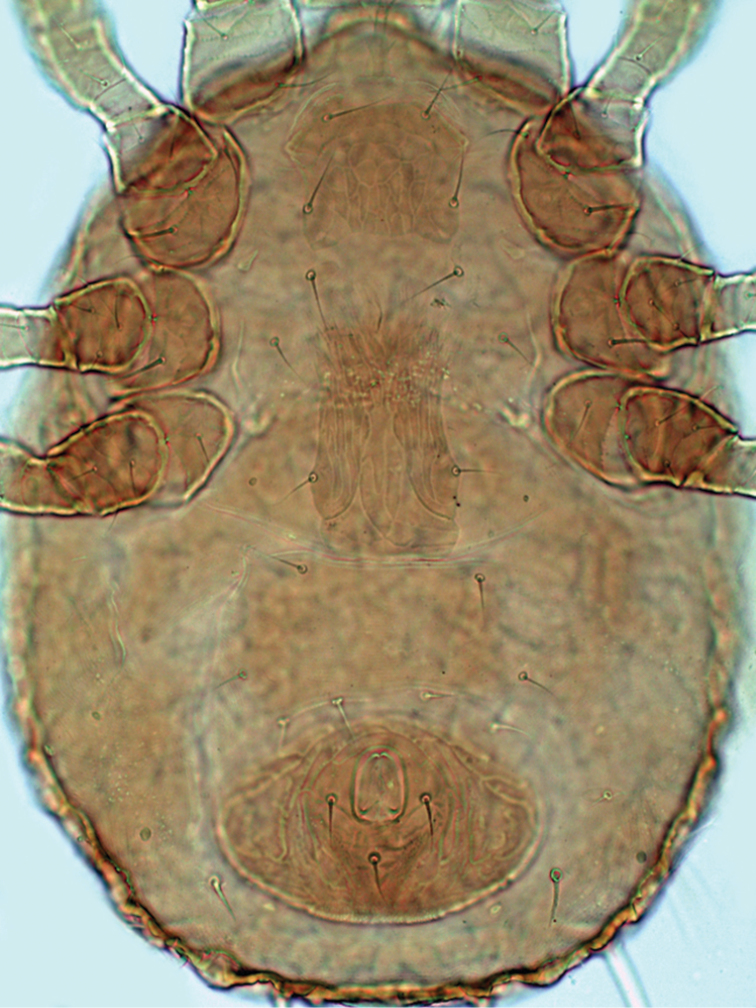
*Ameroseius fungicola*, female, ventral idiosoma.

**Plate 13.**
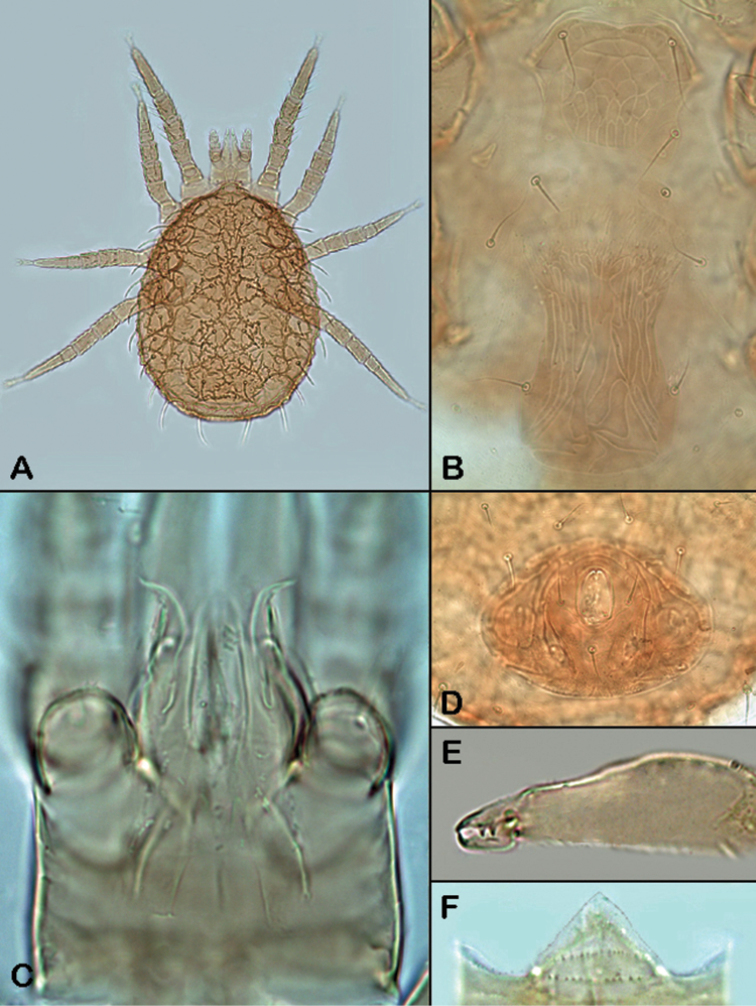
*Ameroseius fungicola*, female. **A** Dorsal idiosoma with legs **B** Sternal and epigynal shield **C** Hypostome, ventral view **D** Anal shield **E** Chelicera, lateral view **F** Epistome.

**Plate 14.**
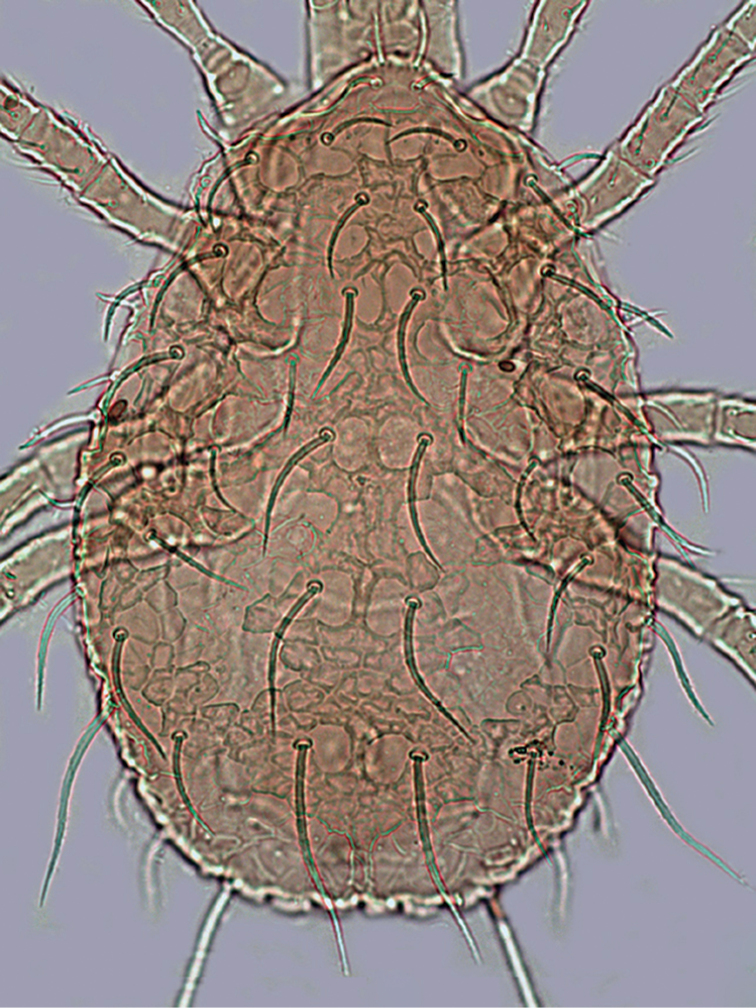
*Ameroseius furcatus*, female, dorsal idiosoma.

**Plate 15.**
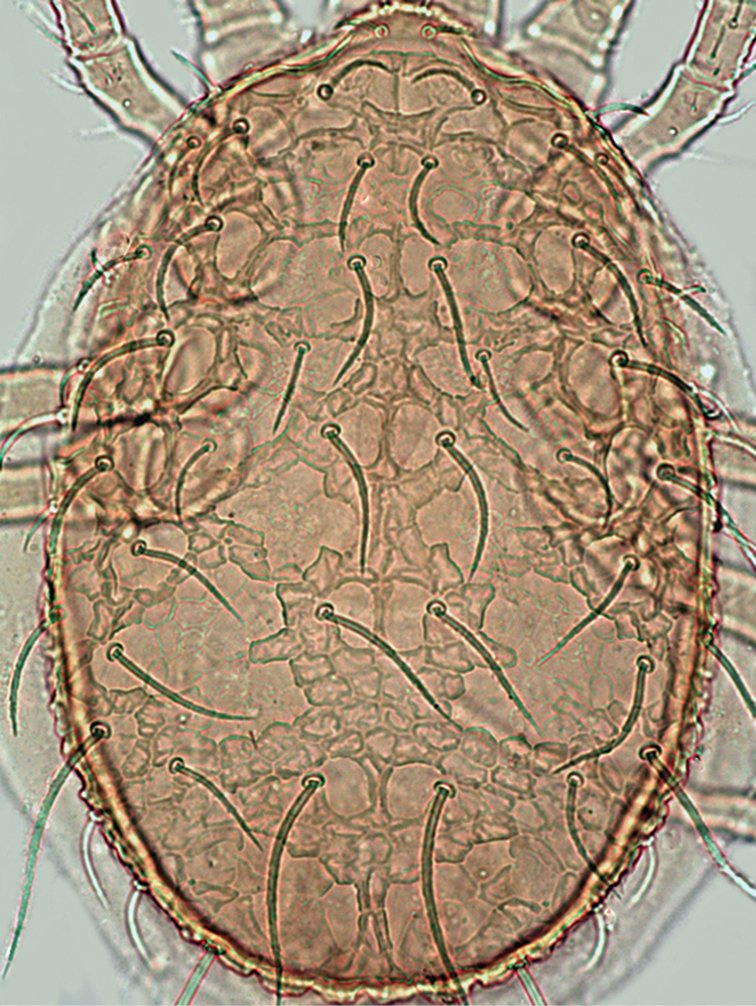
*Ameroseius furcatus*, female, dorsal idiosoma (pressed specimen).

**Plate 16.**
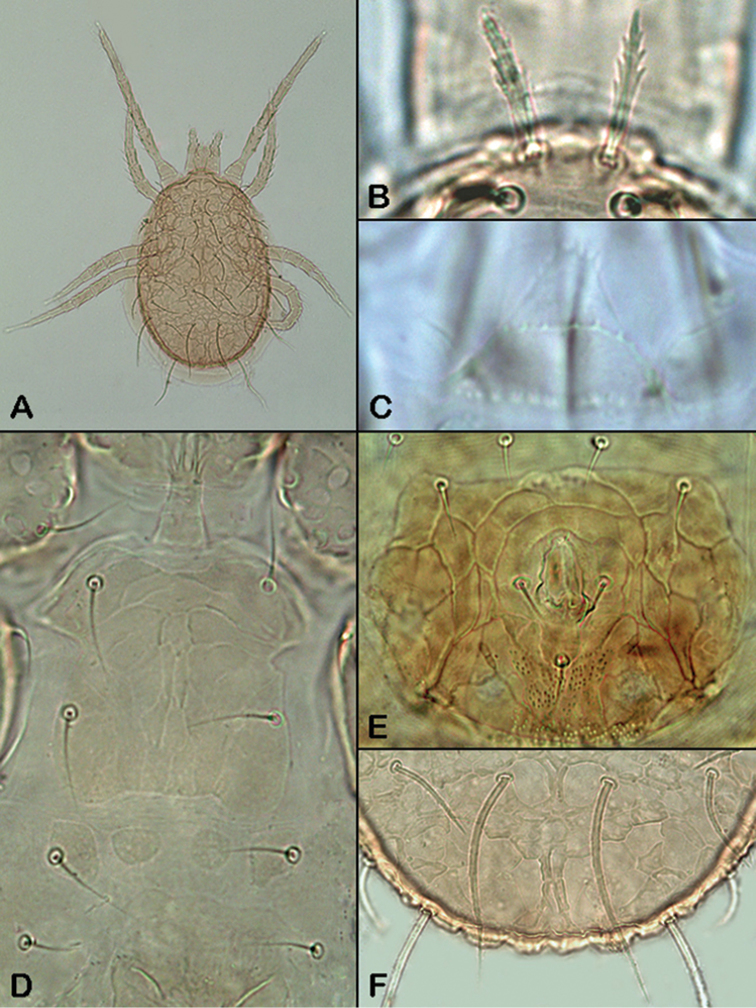
*Ameroseius furcatus*, female. **A** Dorsal idiosoma with legs **B** Setae j1 **C** Epistome **D** Sternal shield and metasternal area **E** Ventrianal shield **F** Posterior margin of dorsal shield.

**Plate 17.**
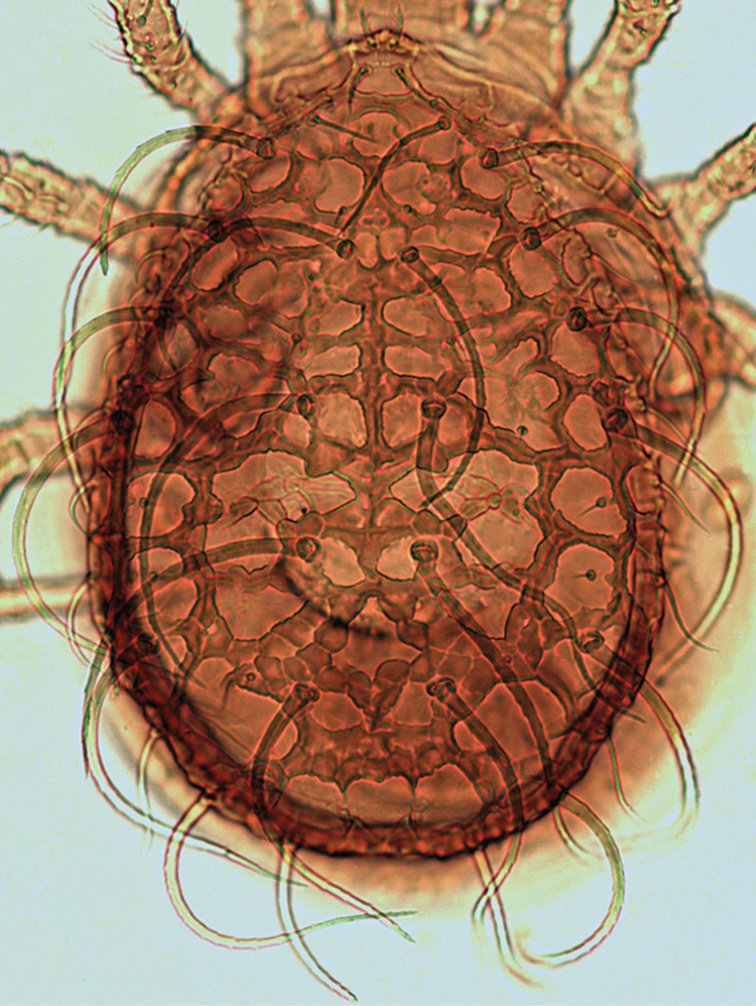
*Ameroseius georgei*, female, dorsal idiosoma (pressed specimen).

**Plate 18.**
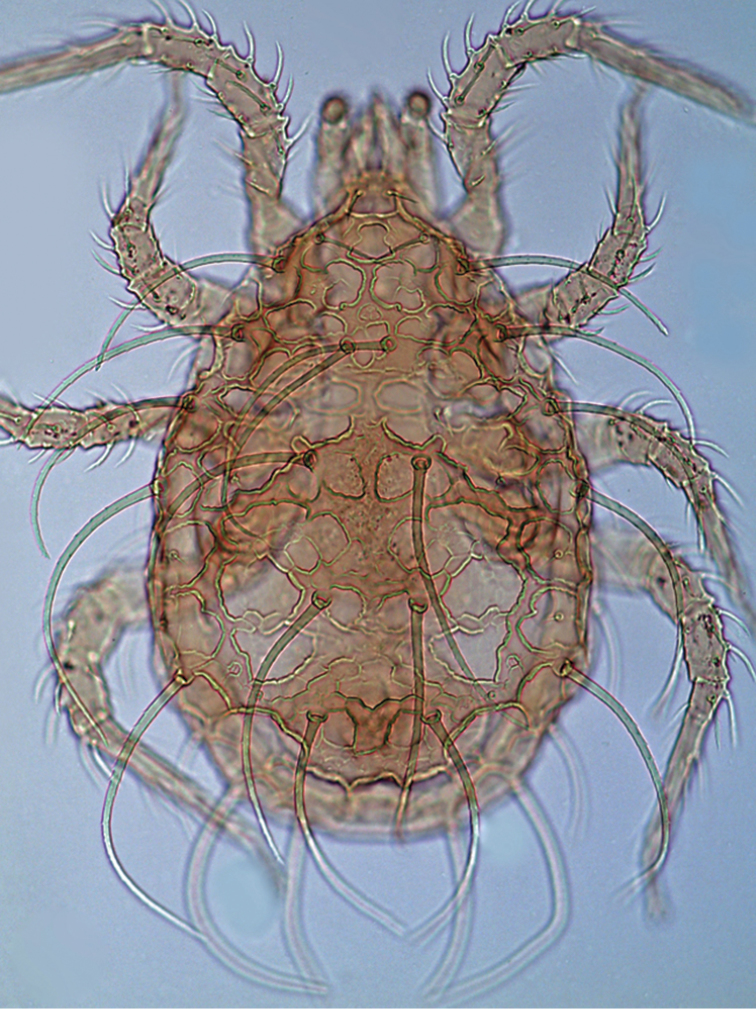
*Ameroseius georgei*, female, dorsal idiosoma.

**Plate 19.**
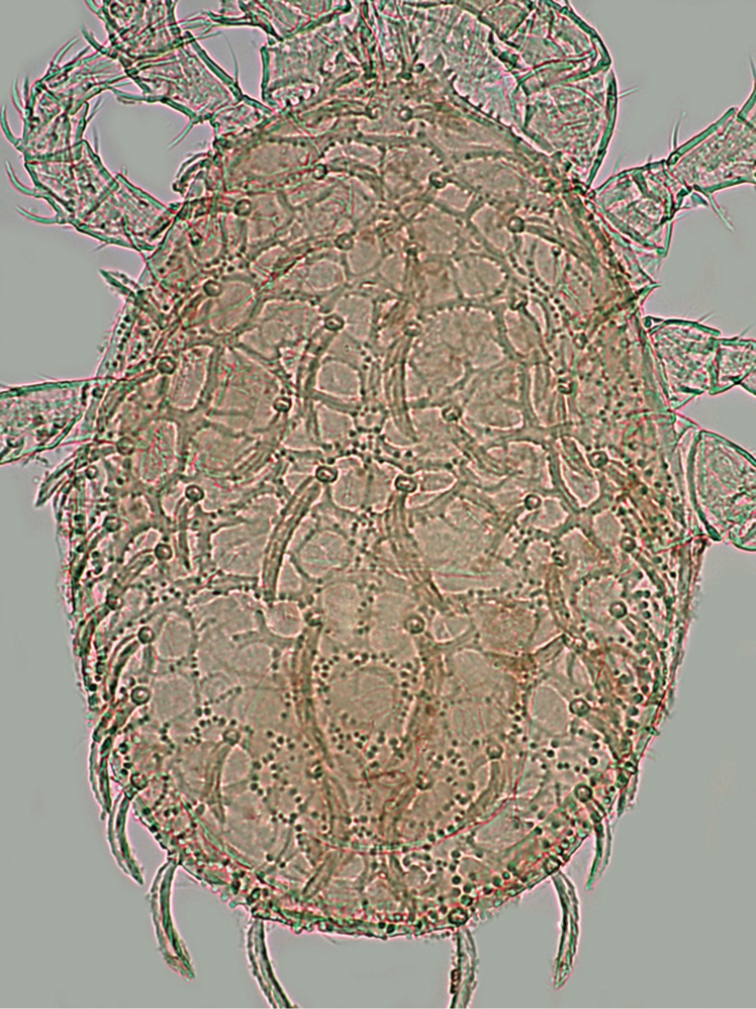
*Ameroseius lehtineni*, female (holotype, from Finland), dorsal idiosoma.

**Plate 20.**
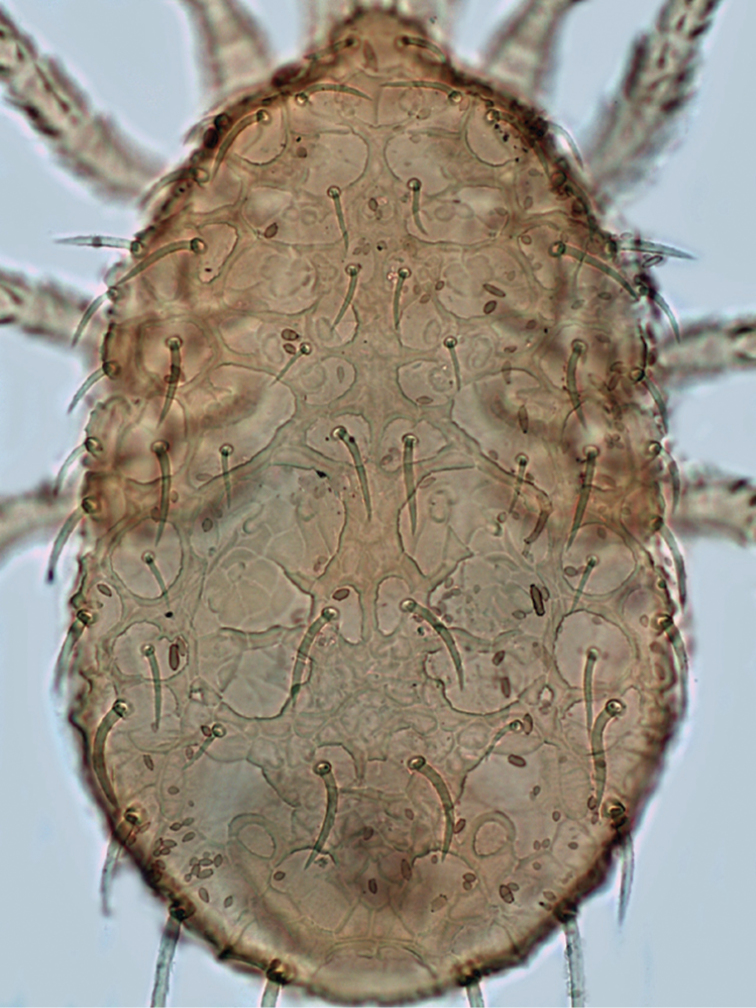
*Ameroseius lidiae*, female, dorsal idiosoma.

**Plate 21.**
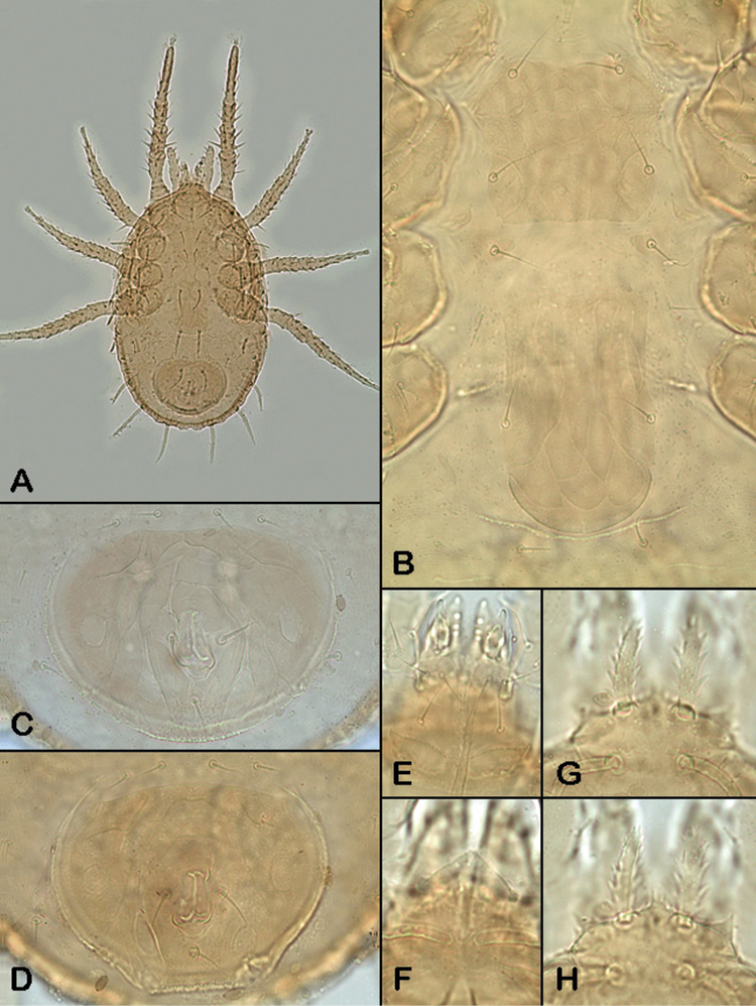
*Ameroseius lidiae*, female. **A** Dorsal idiosoma with legs **B** Sternal and epigynal shield **C–D** Anal shield, variant forms **E** Hypostome, ventral view **F** Epistome **G–H** Setae j1 and dentation of vertex.

**Plate 22.**
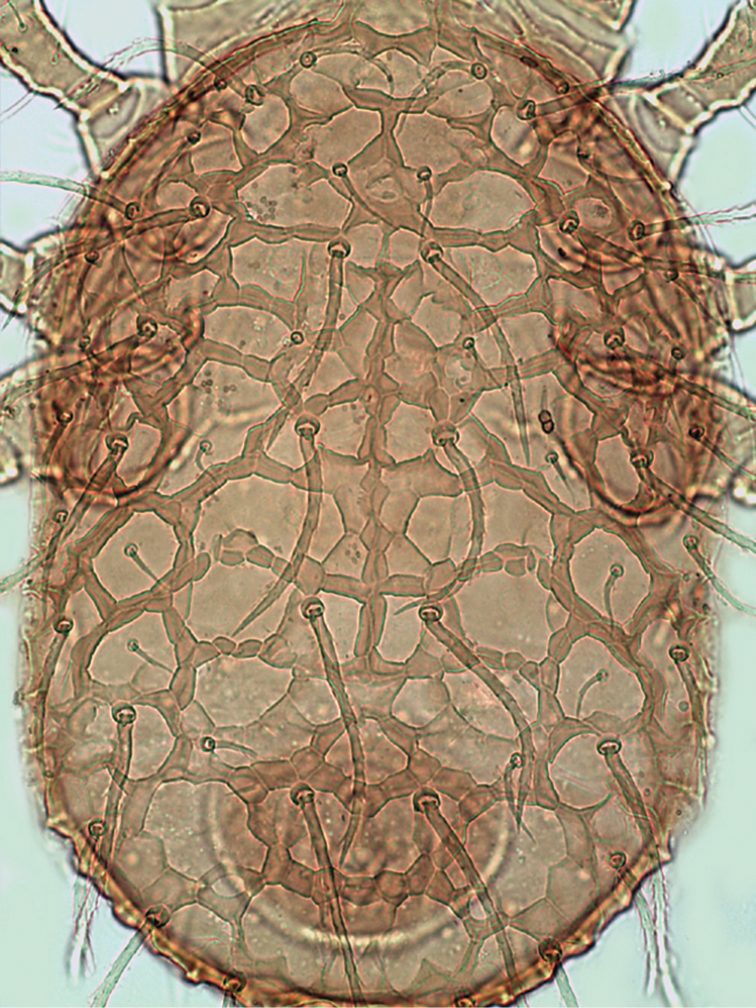
*Ameroseius longitrichus*, female, dorsal idiosoma.

**Plate 23.**
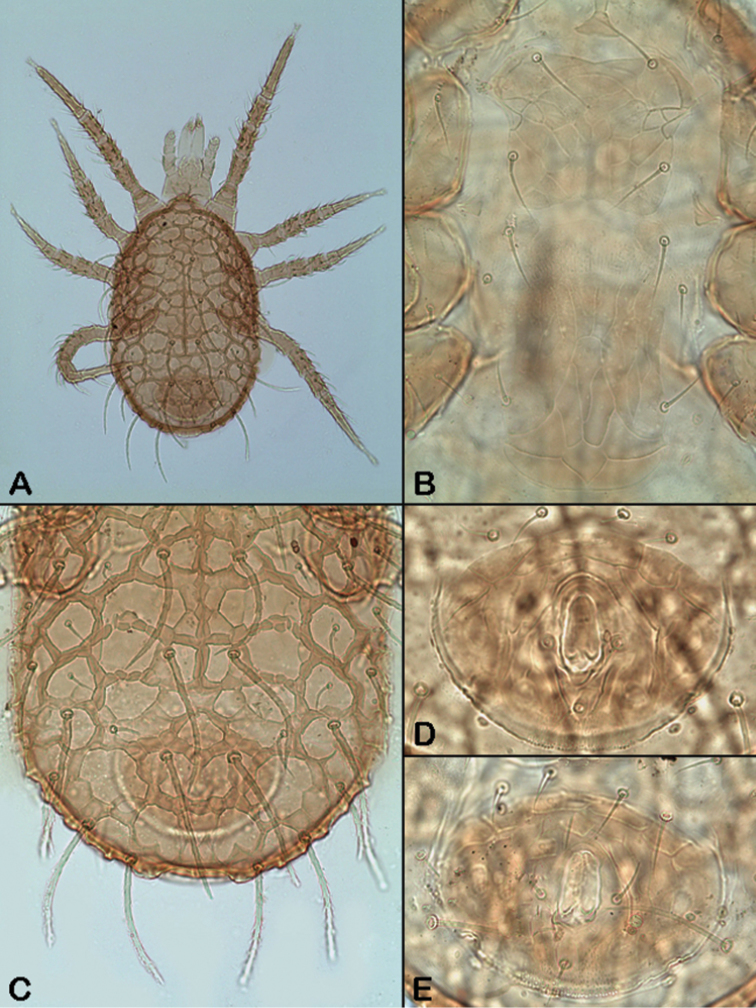
*Ameroseius longitrichus*, female. **A** Dorsal idiosoma with legs **B** Sternal and epigynal shield **C** Posterior dorsal shield **D–E** Anal shield, variant forms.

**Plate 26.**
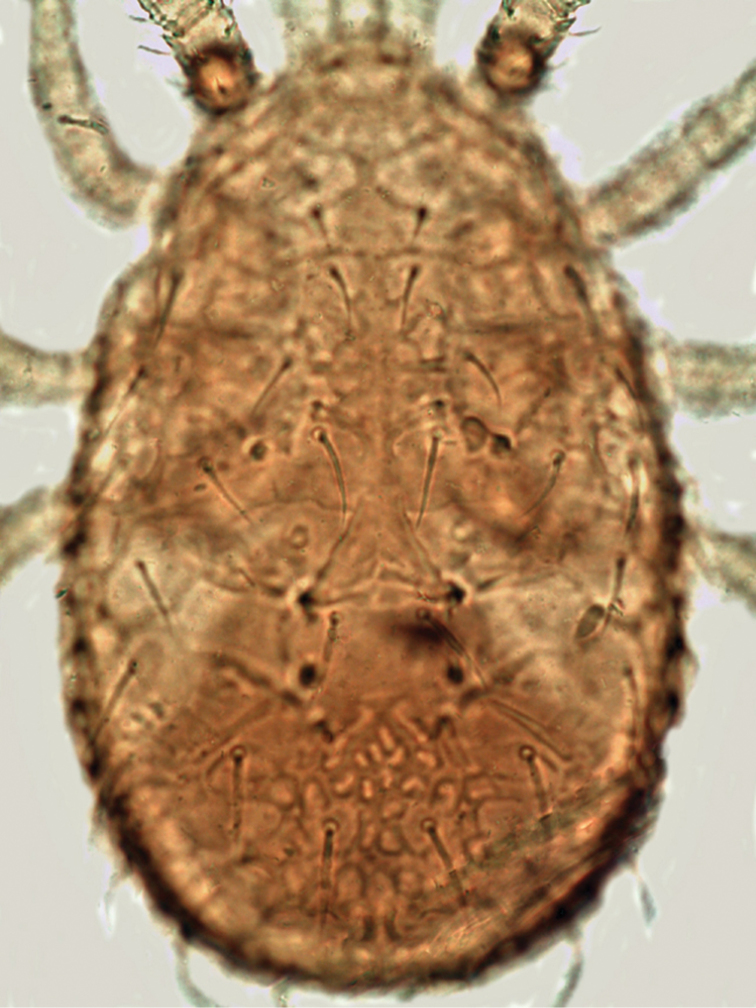
*Ameroseius sculptilis*, female, dorsal idiosoma.

**Plate 27.**
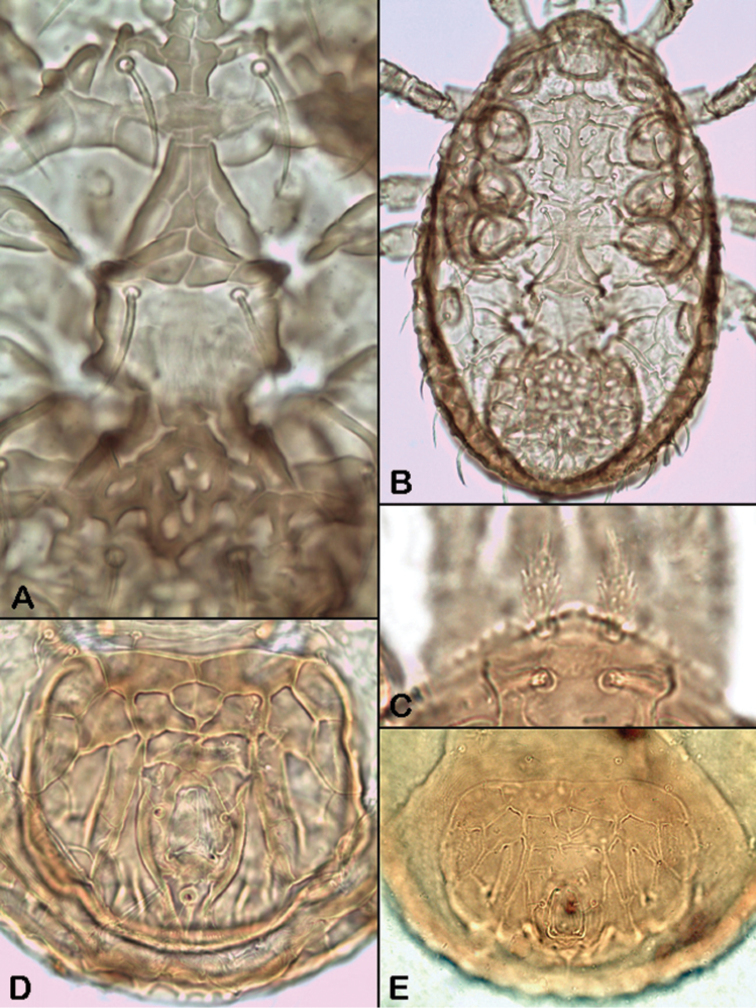
*Ameroseius sculptilis*, female (**A–D** a specimen from Germany). **A** Dorsal shield sculpture, medial area **B** Dorsal idiosoma **C** Setae j1 **D–E** Anal shield, variant forms.

**Plate 28.**
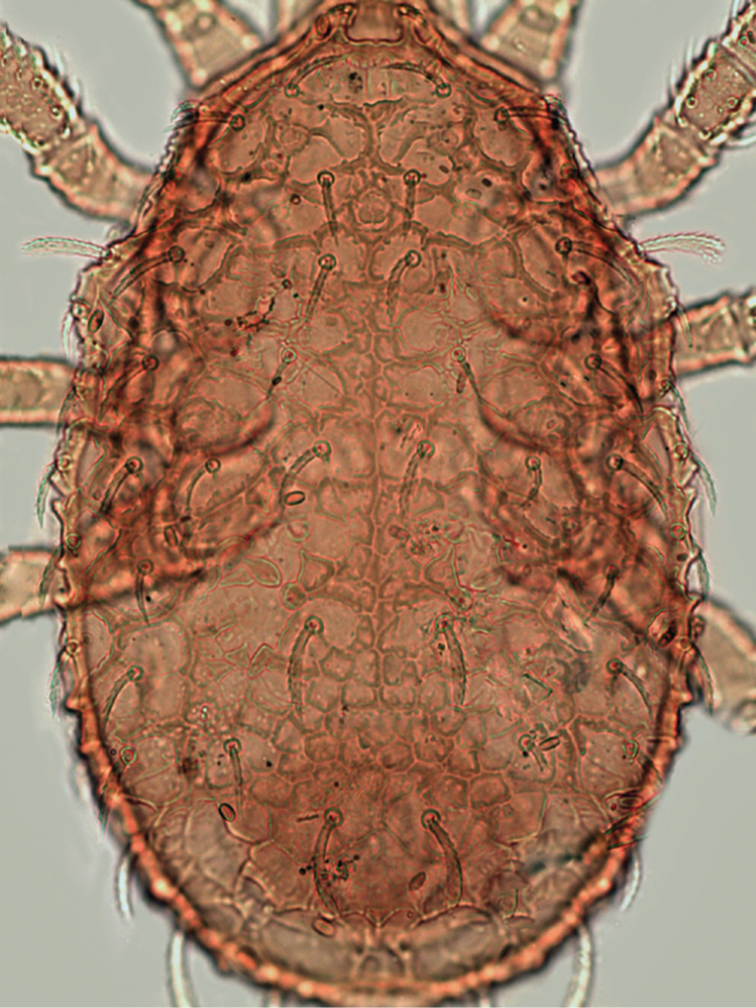
*Ameroseius ulmi*, female, dorsal idiosoma.

**Plate 29.**
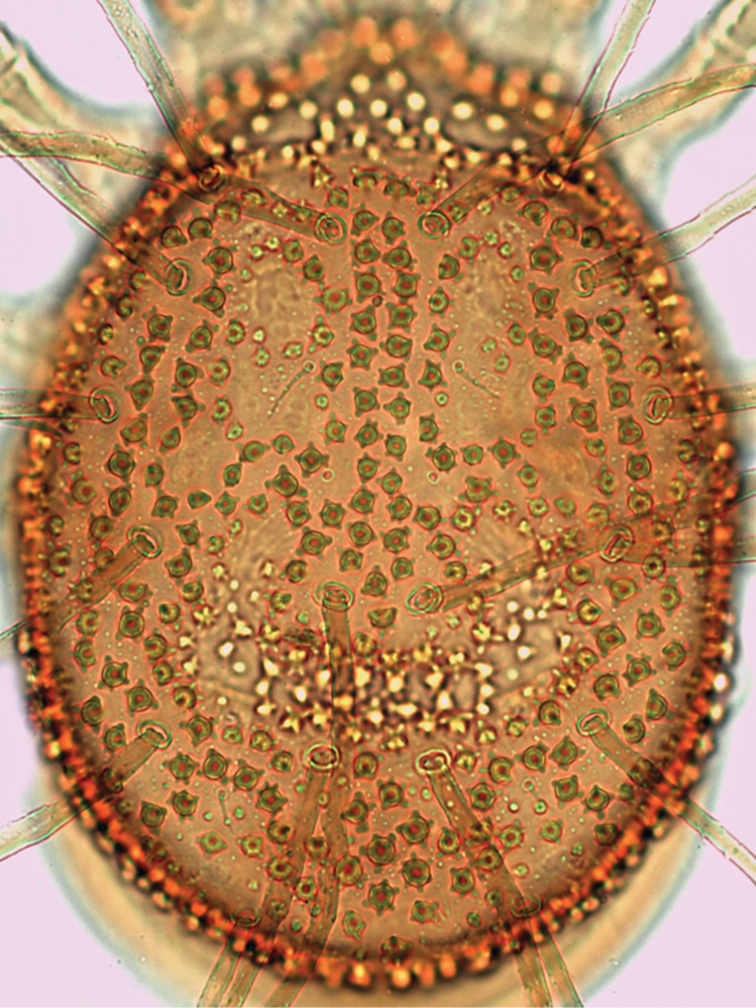
*Epicriopsis horridus*, female, dorsal idiosoma.

**Plate 30.**
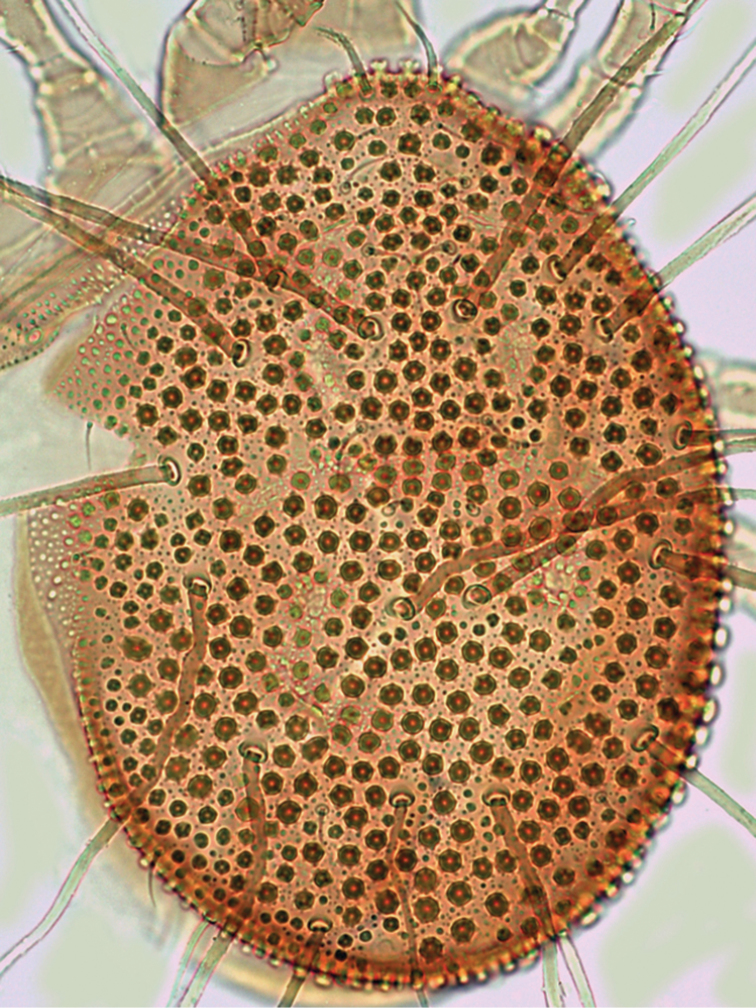
*Epicriopsis hungaricus*, female, dorsal idiosoma.

**Plate 31.**
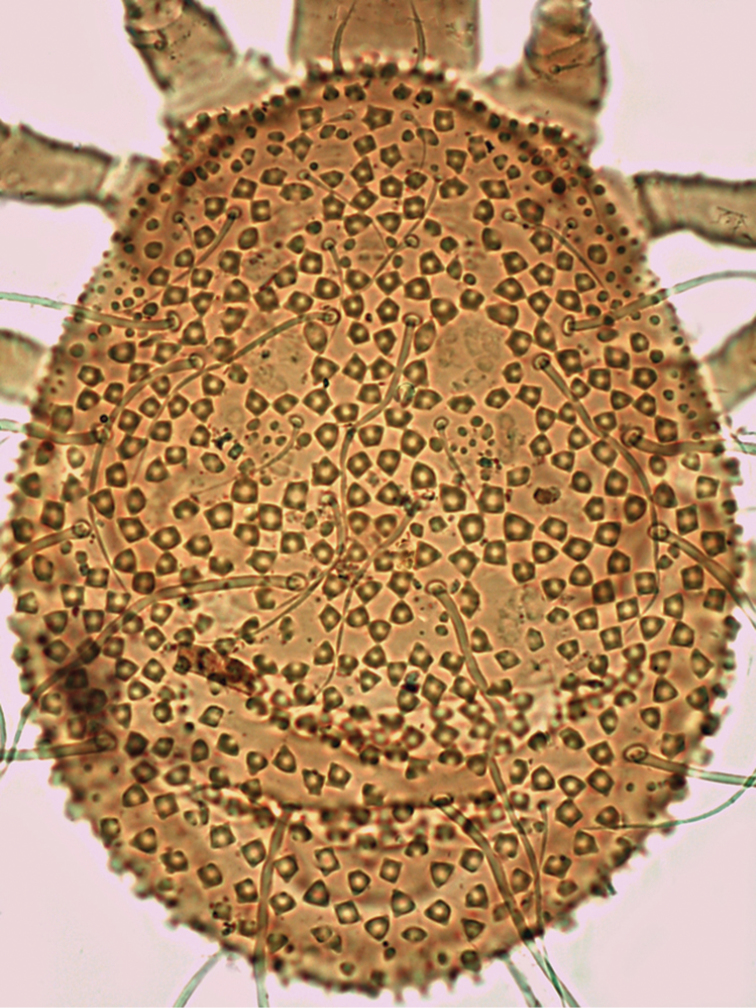
*Epicriopsis mirabilis*, female, dorsal idiosoma.

**Plate 32.**
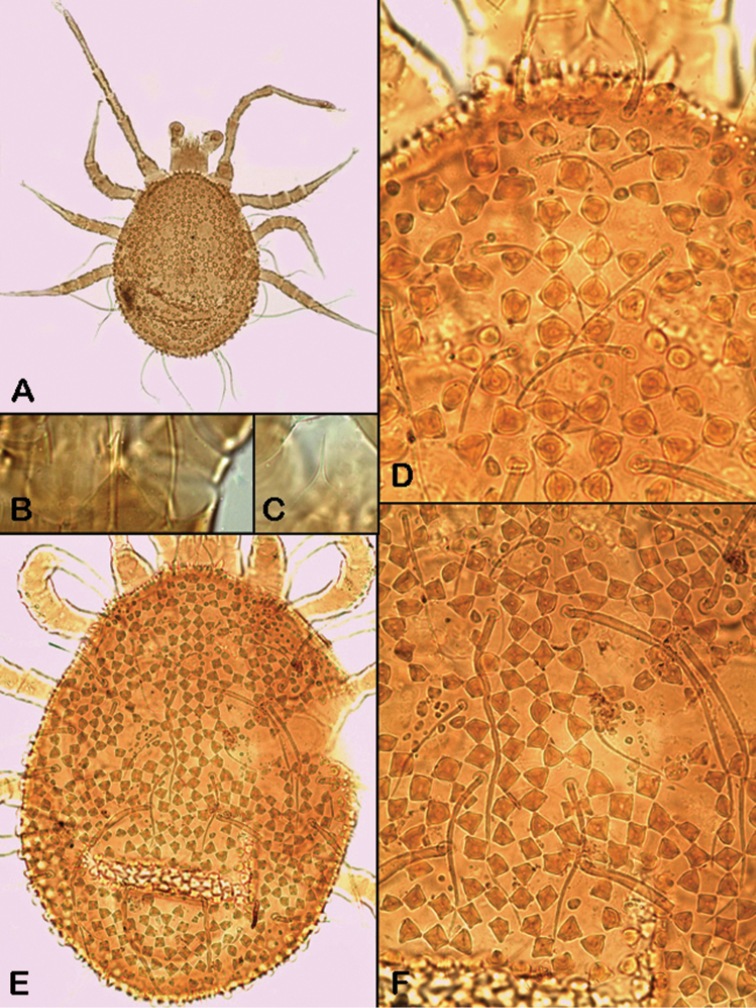
*Epicriopsis mirabilis*, female. **A** Dorsal idiosoma with legs **B–C** Epistome, variant forms **D** Dorsal shield, anterior part **E** Dorsal idiosoma **F** Dorsal shield, medial part.

**Plate 33.**
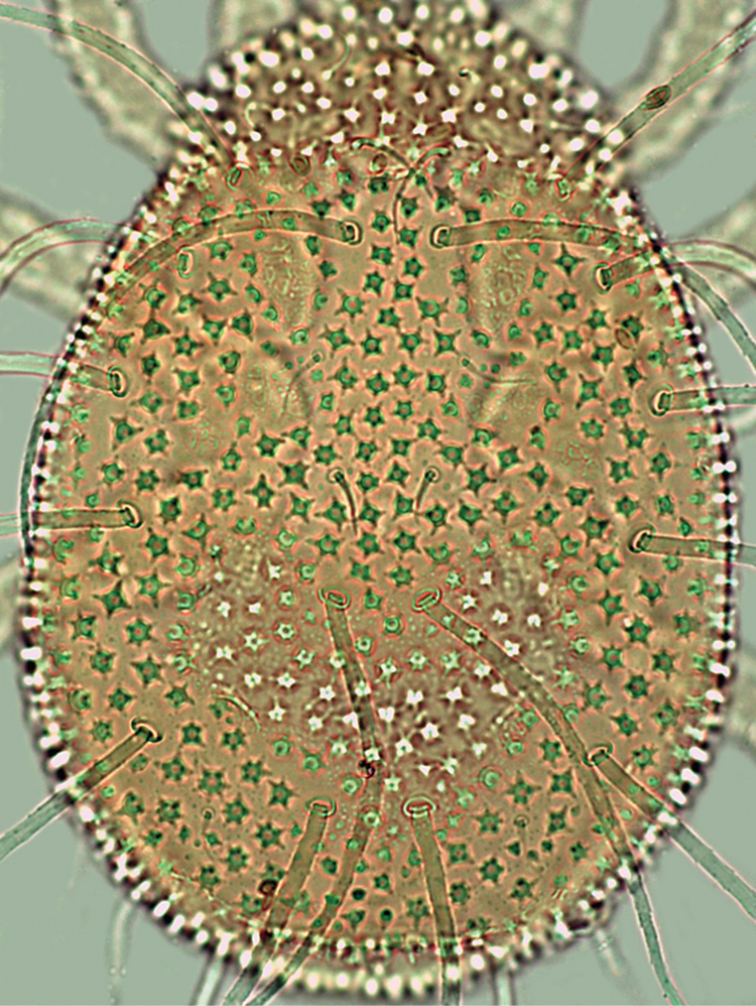
*Epicriopsis palustris*, female, dorsal idiosoma.

**Plate 34.**
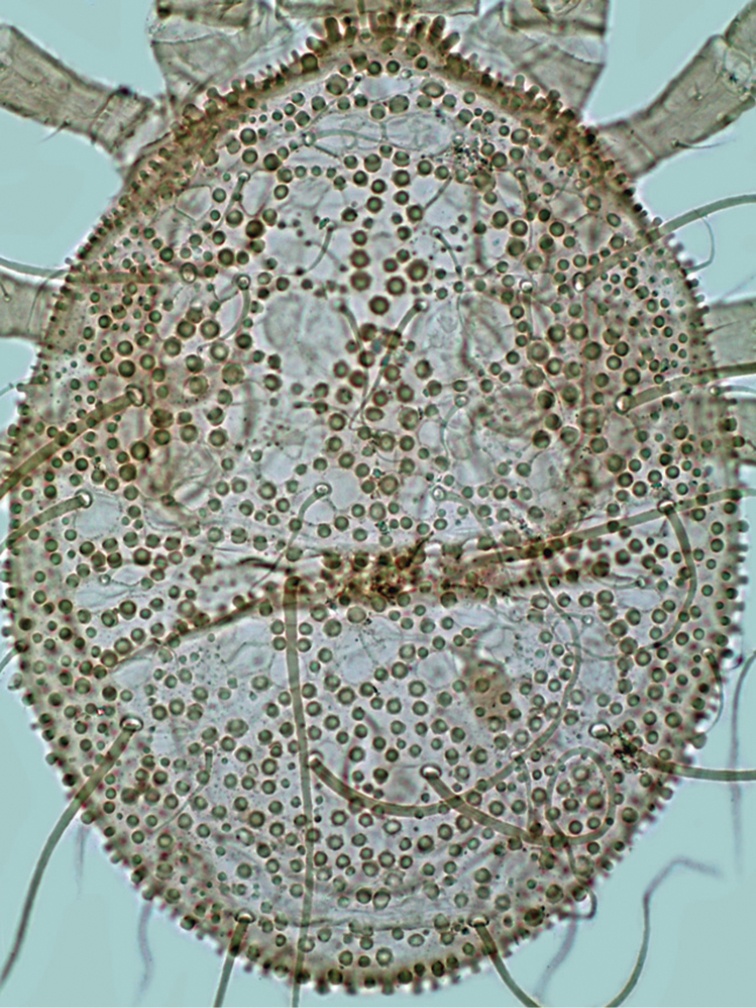
*Epicriopsis suedus*, female (holotype, from Sweden), dorsal idiosoma.

**Plate 38.**
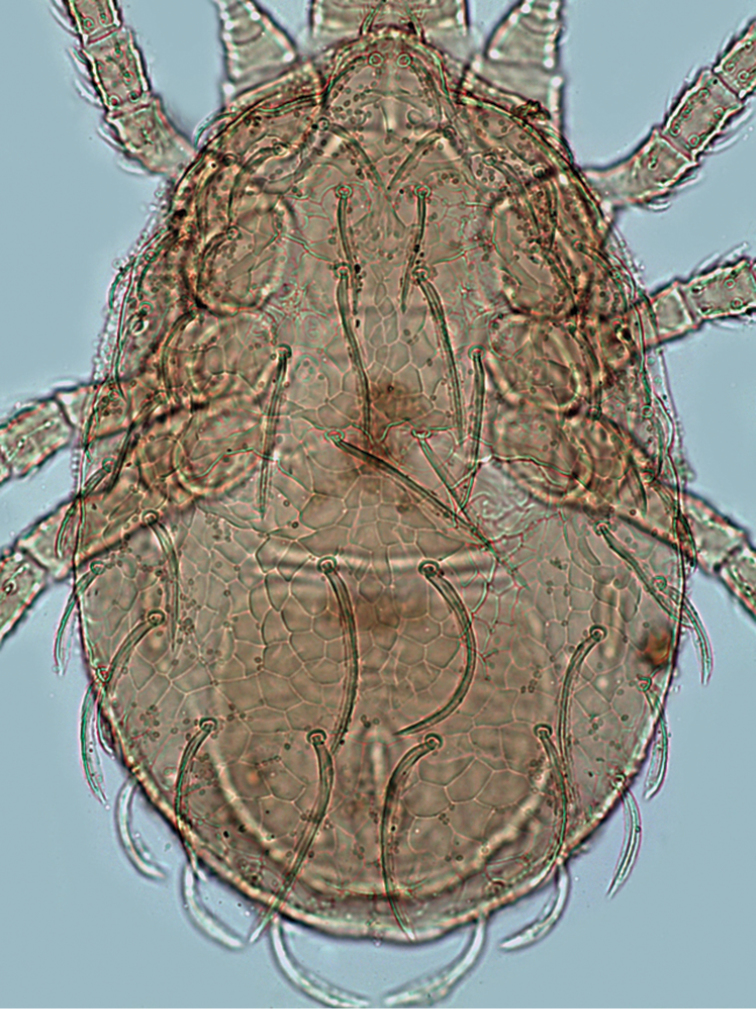
*Kleemannia dolichochaeta*, female (holotype, from Spain), dorsal idiosoma.

**Plate 39.**
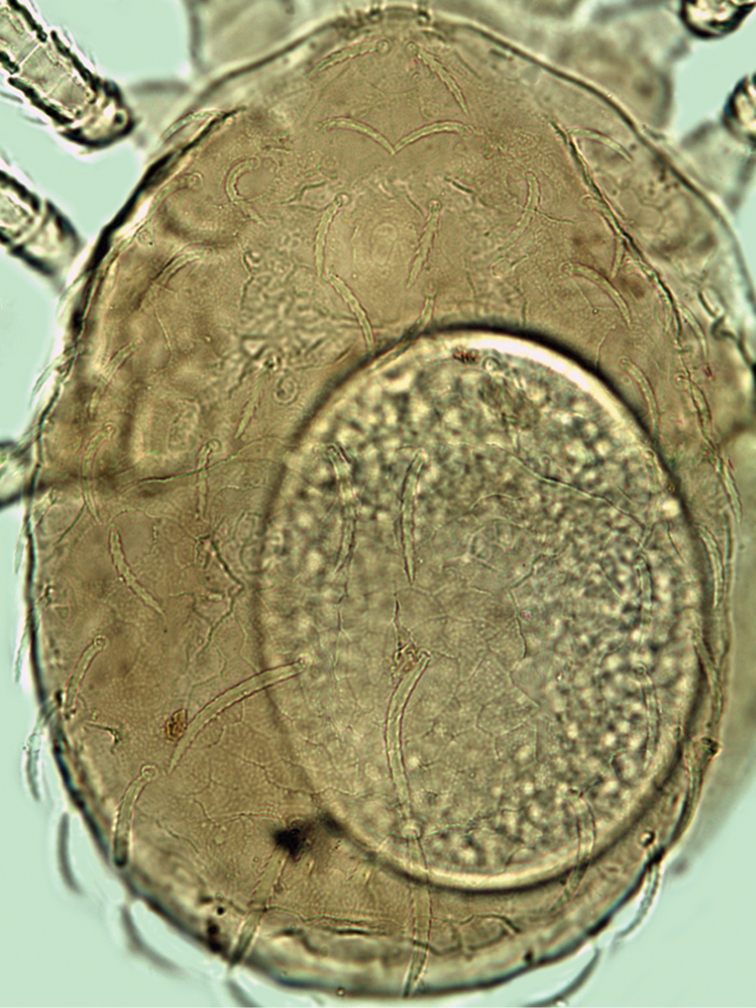
*Kleemannia insignis*, female, dorsal idiosoma.

**Plate 40.**
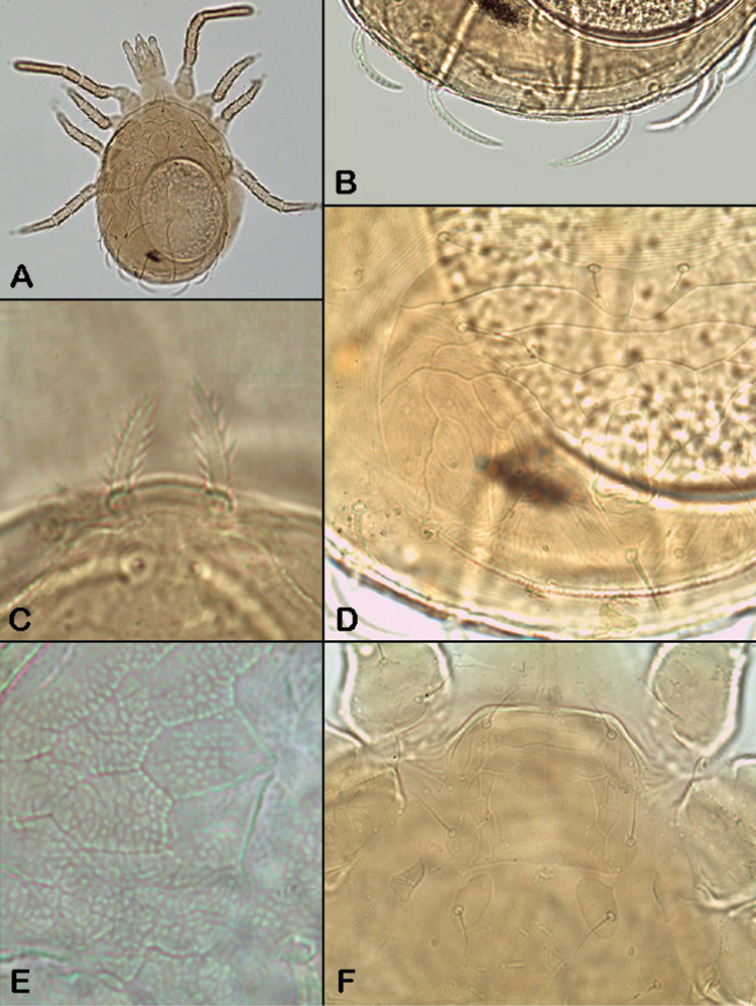
*Kleemannia insignis*, female. **A** Dorsal idiosoma with legs **B** Posterior margin of idiosoma **C** Setae j1 **D** Ventrianal shield, a part **E** Dorsal shield sculpture **F** Sternal shield and metasternal area.

**Plate 41.**
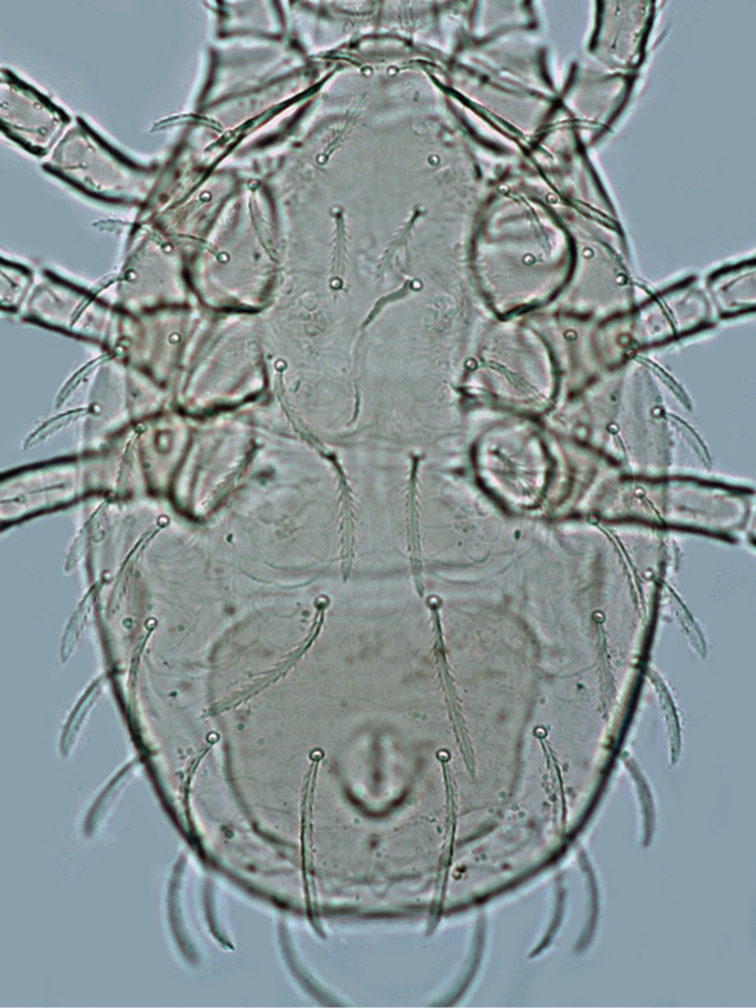
*Kleemannia kosi*, female (from Germany), dorsal idiosoma.

**Plate 42.**
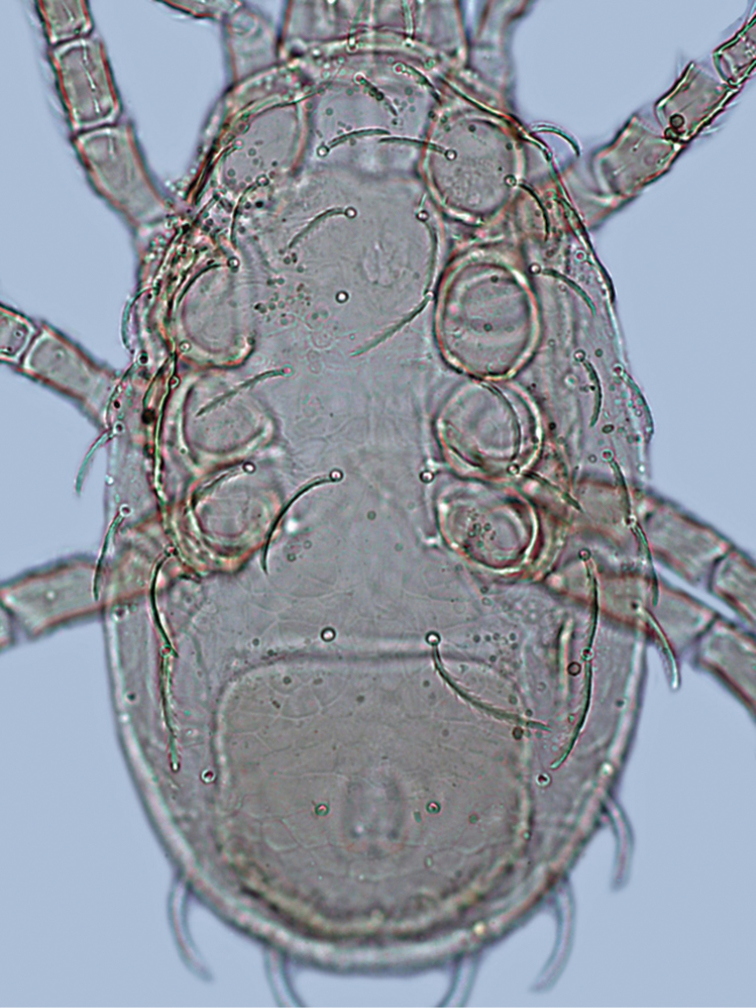
*Kleemannia kosi*, female (from Germany), dorsal idiosoma.

**Plate 43.**
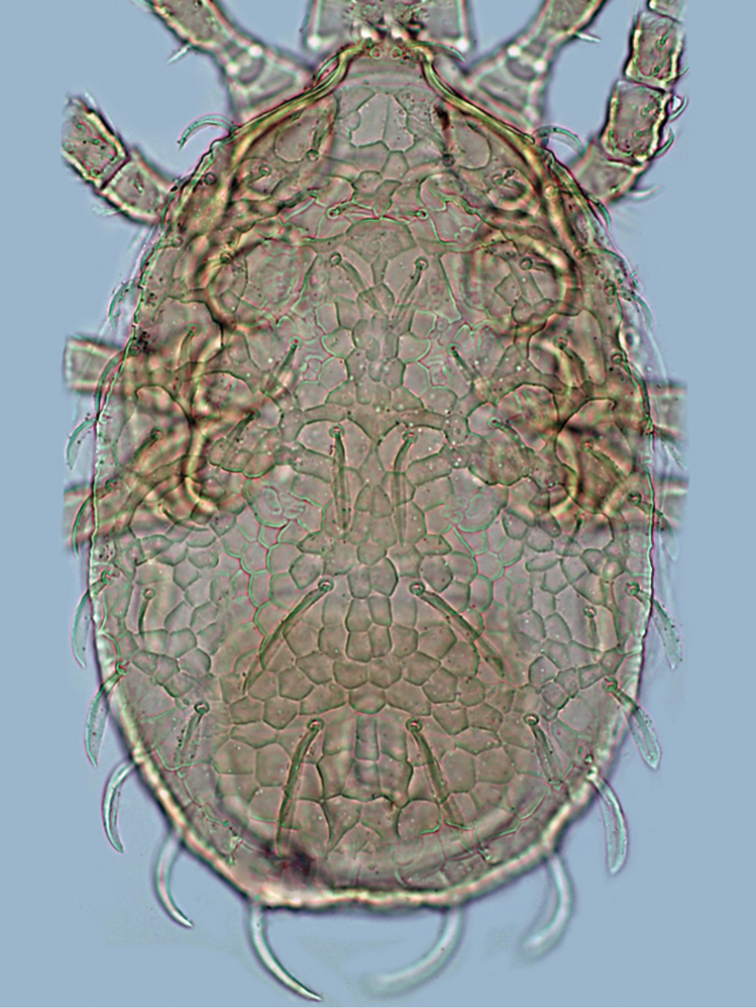
*Kleemannia nova*, female (from Iran), dorsal idiosoma.

**Plate 44.**
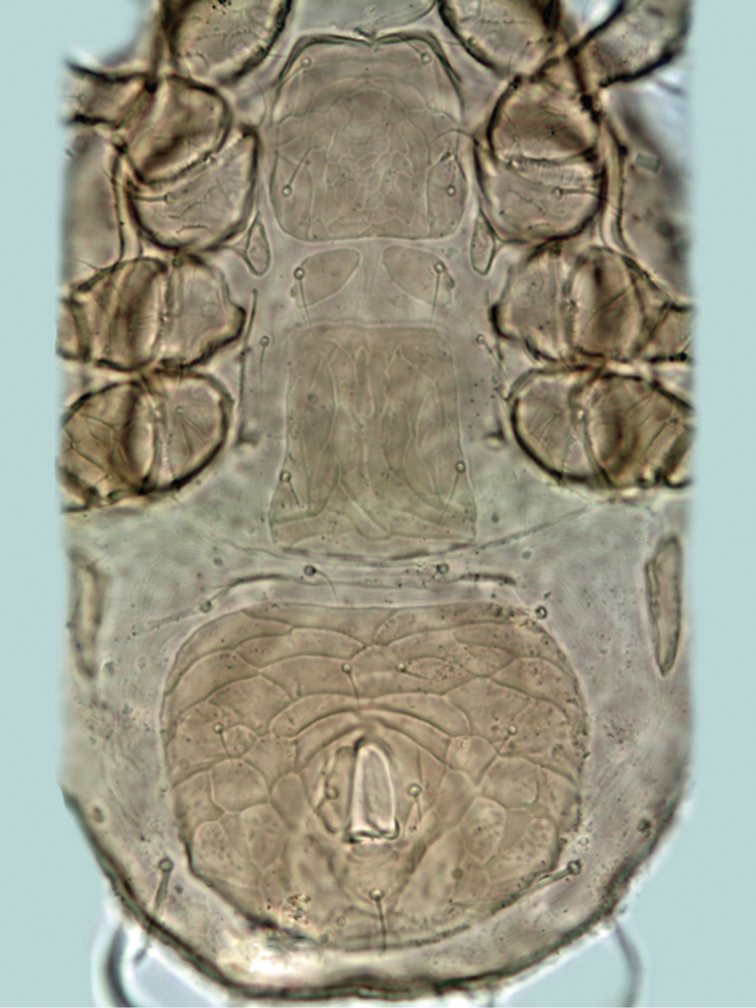
*Kleemannia nova*, female (from Iran), ventral idiosoma.

**Plate 45.**
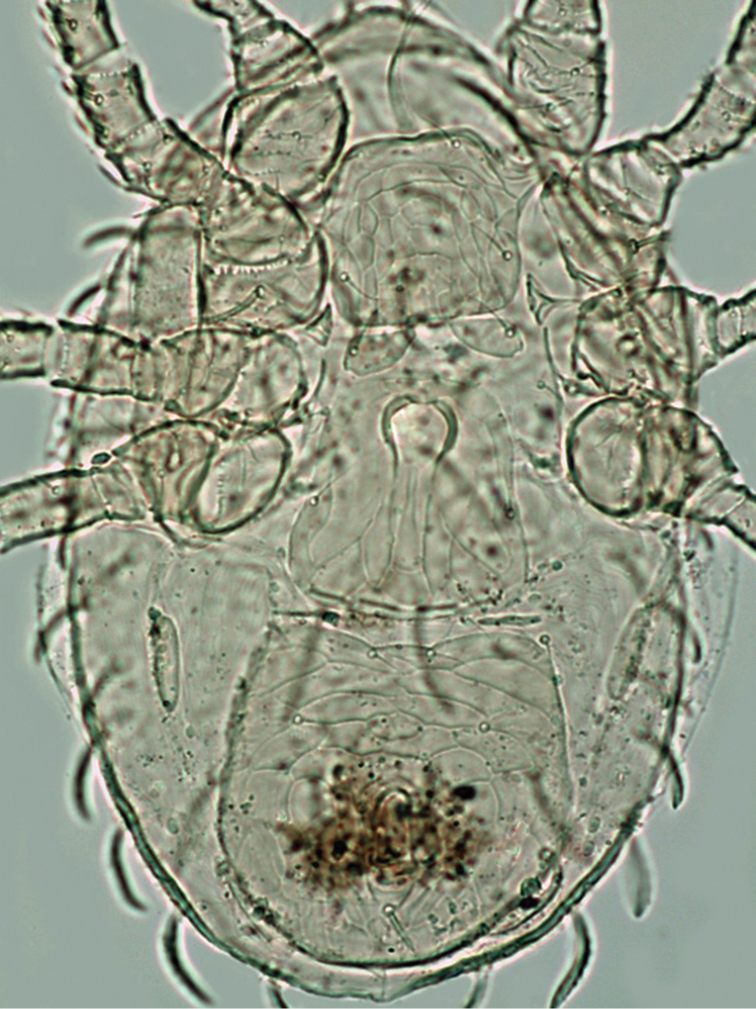
*Kleemannia parplumosa*, female (from Germany), dorsal idiosoma (pressed specimen).

**Plate 48.**
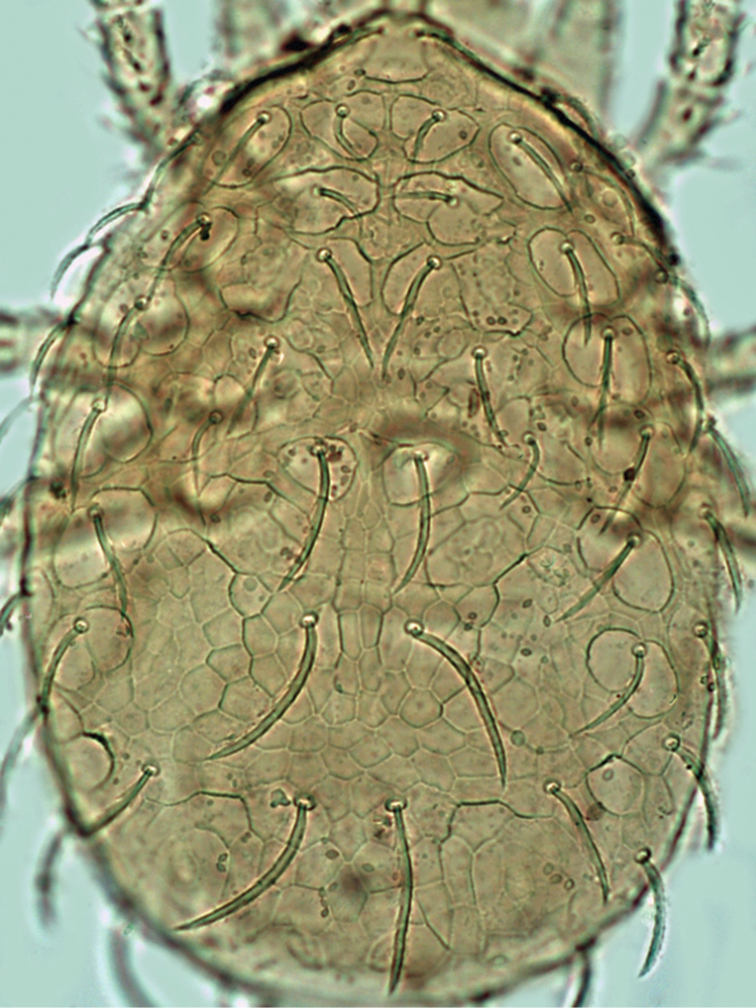
*Kleemannia plumea*, female, dorsal idiosoma.

**Plate 49.**
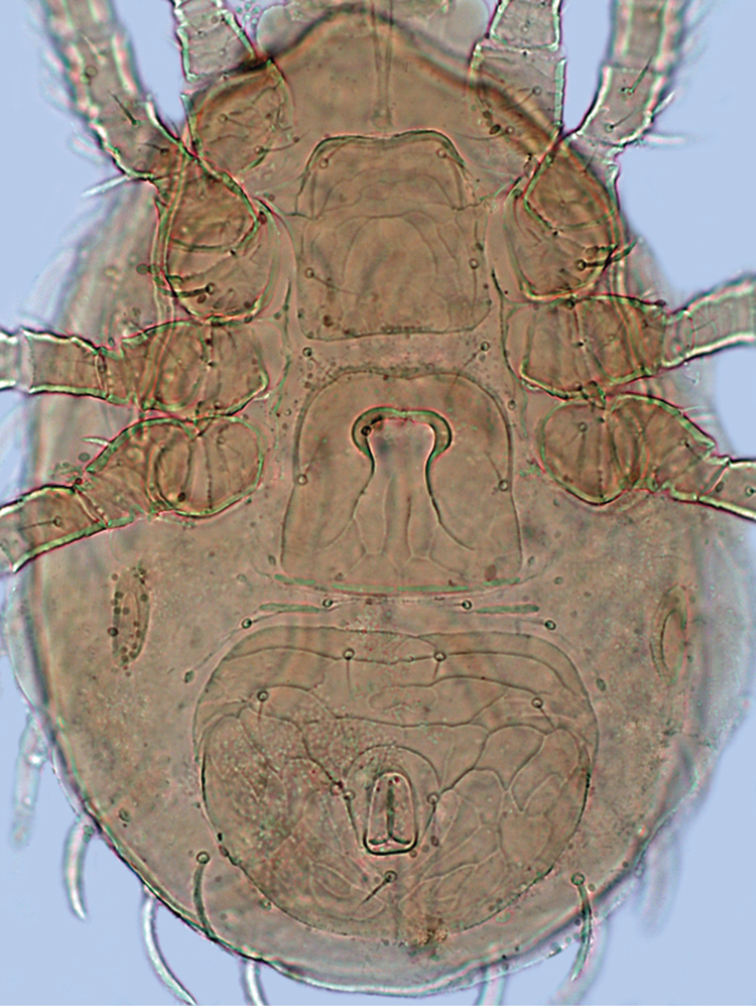
*Kleemannia plumea*, female, ventral idiosoma.

**Plate 61.**
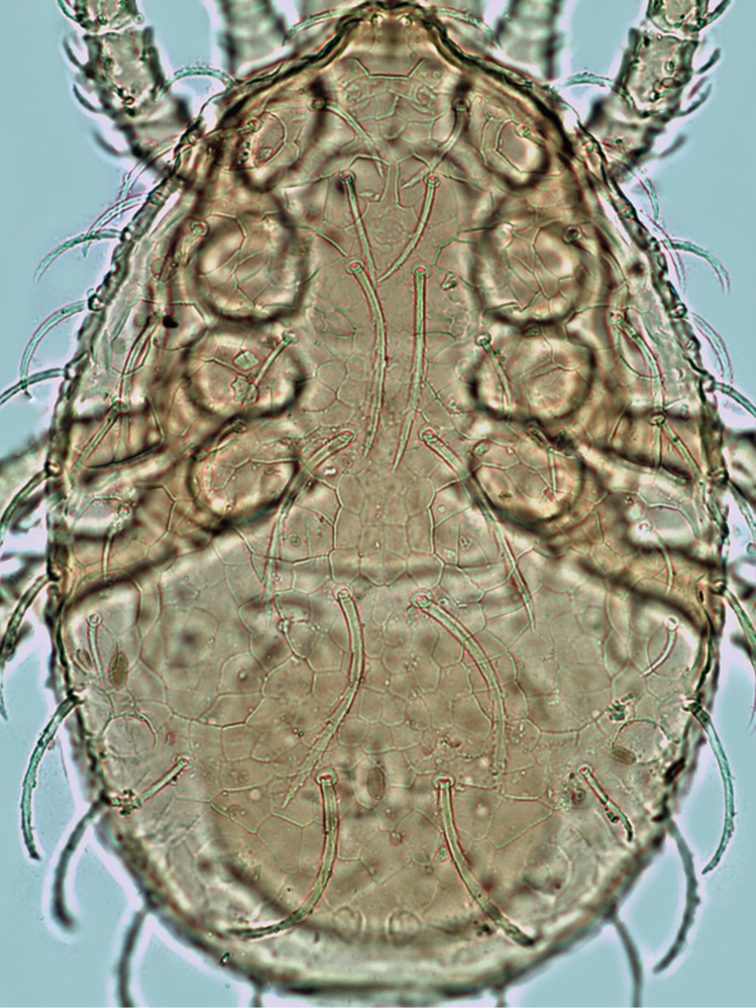
*Kleemannia tenella*, female, dorsal idiosoma.

**Plate 62.**
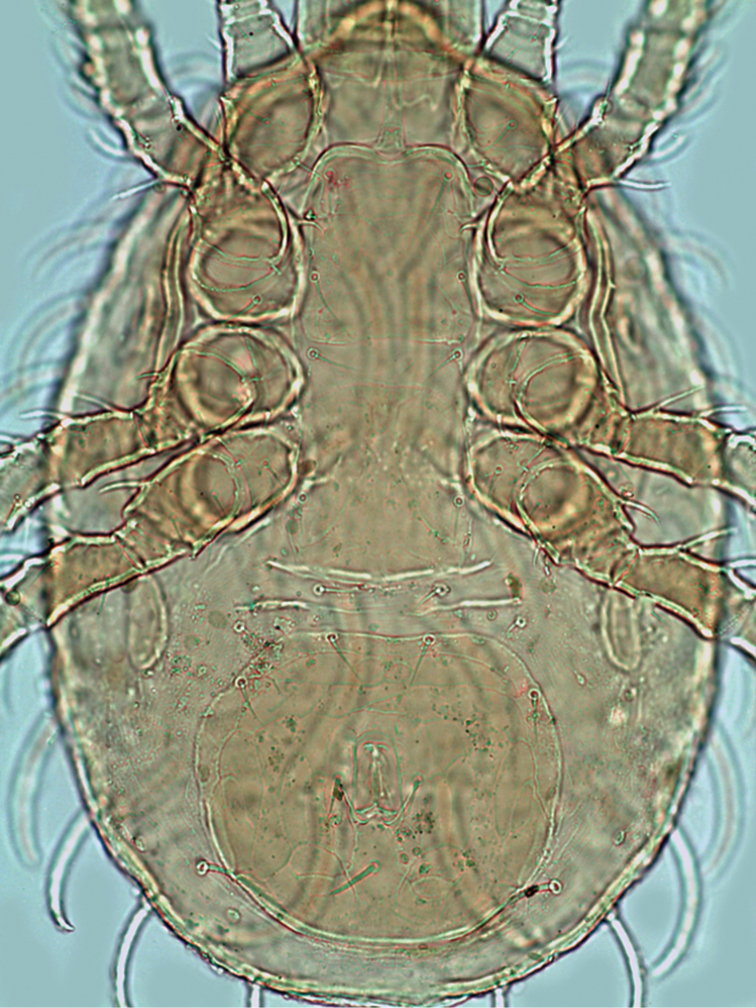
*Kleemannia tenella*, female, ventral idiosoma.

**Plate 63.**
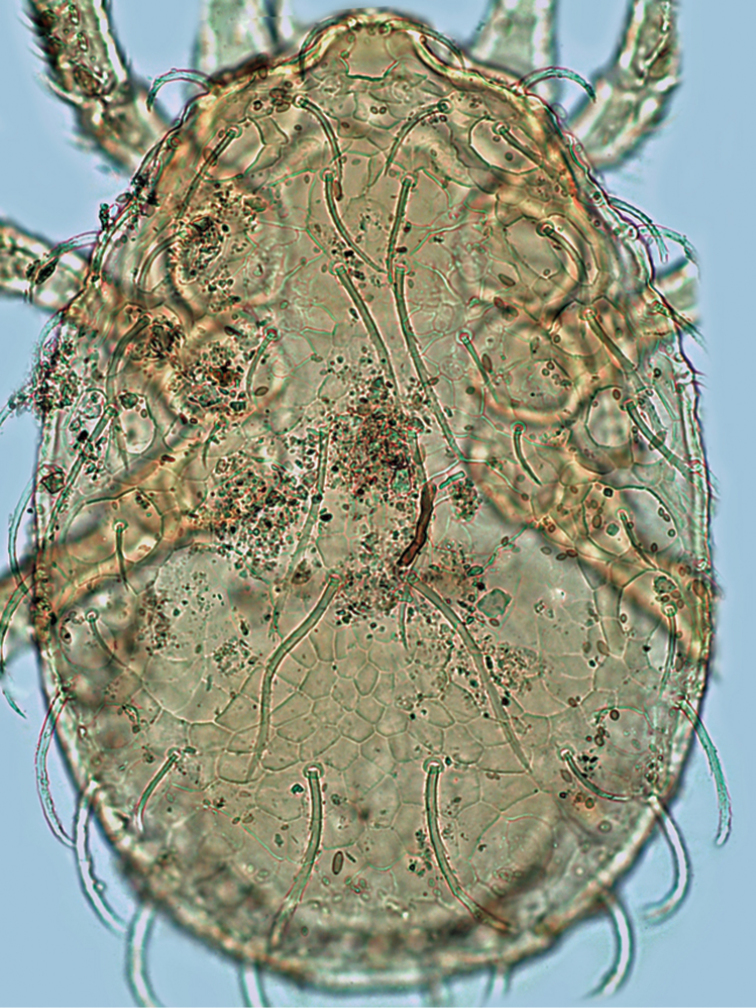
*Kleemannia tenella*, female, dorsal idiosoma.

**Plate 64.**
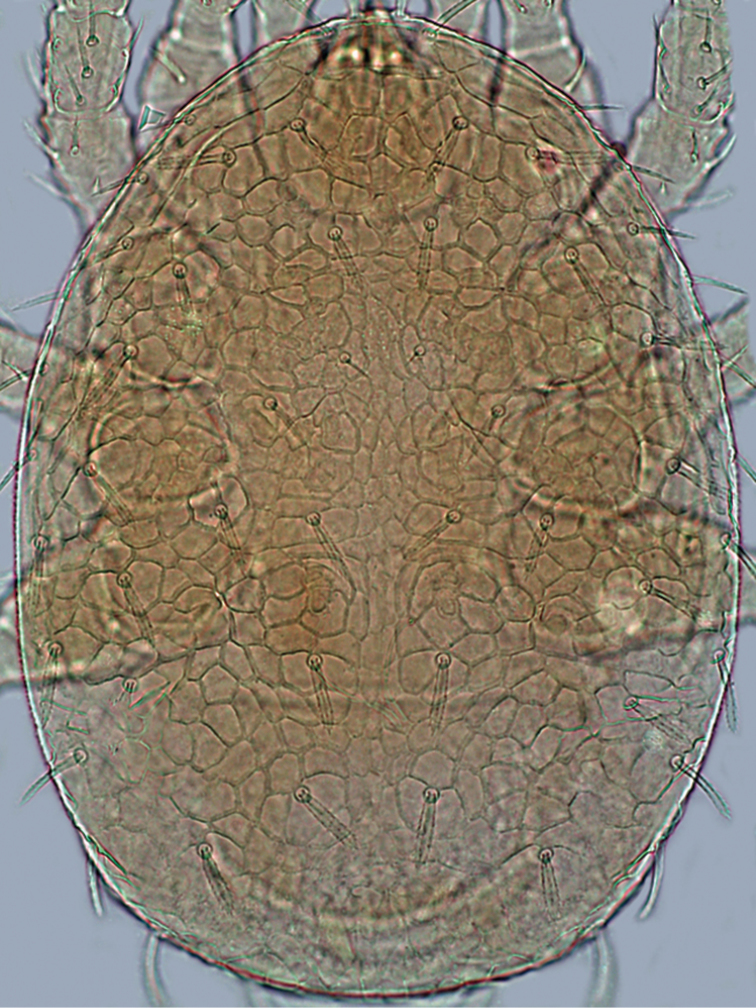
*Neocypholaelaps favus*, female, dorsal idiosoma.

**Plate 65.**
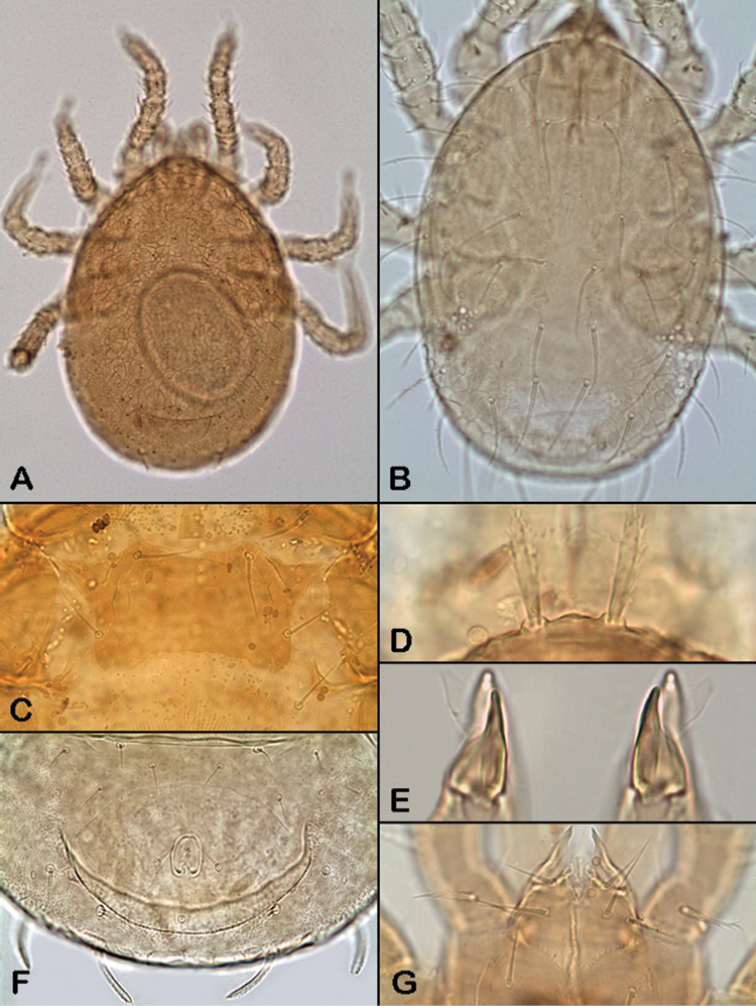
*Neocypholaelaps favus*. **A** Dorsal idiosoma with legs, female **B** Dorsal idiosoma, male **C** Sternal shield, female **D** Setae j1 and epistome, female **E** Cheliceral digits, female **F** Anal shield **G** Hypostome, ventral view.

**Plate 67.**
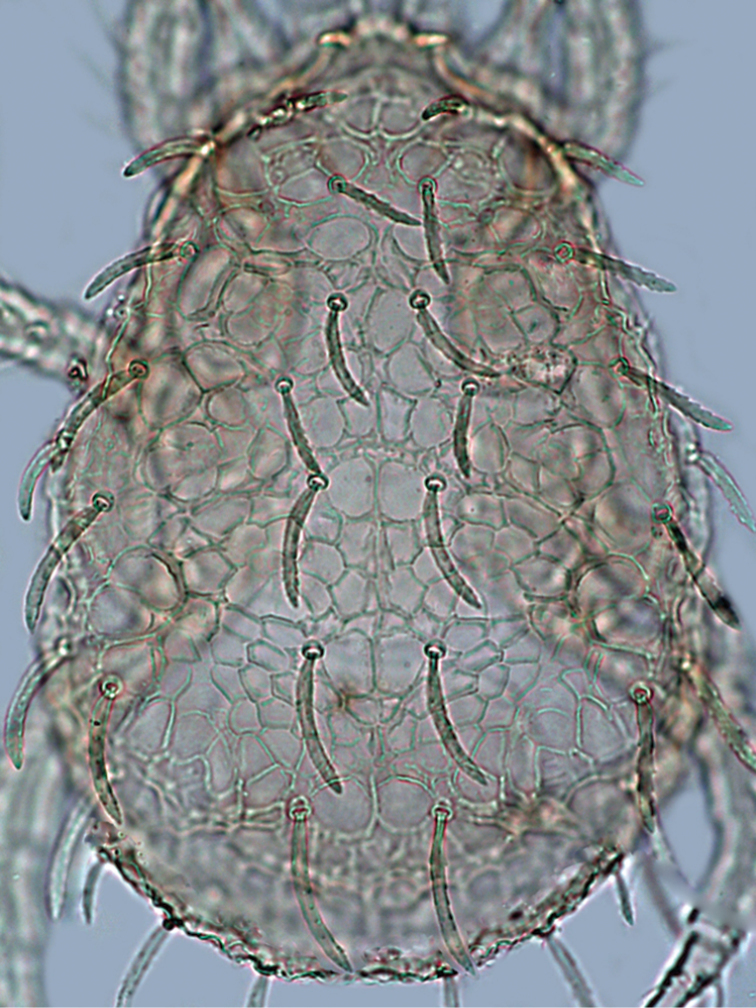
*Pseudoameroseius michaelangeli*, female (from Spain), dorsal idiosoma.

**Plate 68.**
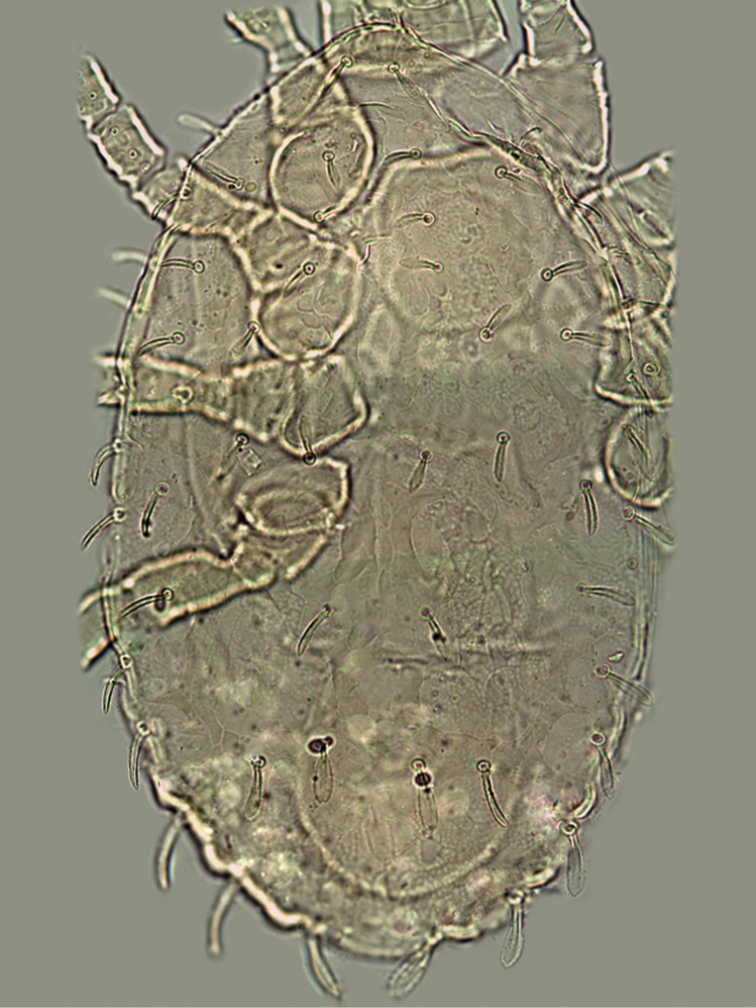
*Sertitympanum aegyptiacum*, female (from Iran), dorsal idiosoma.

**Plate 69.**
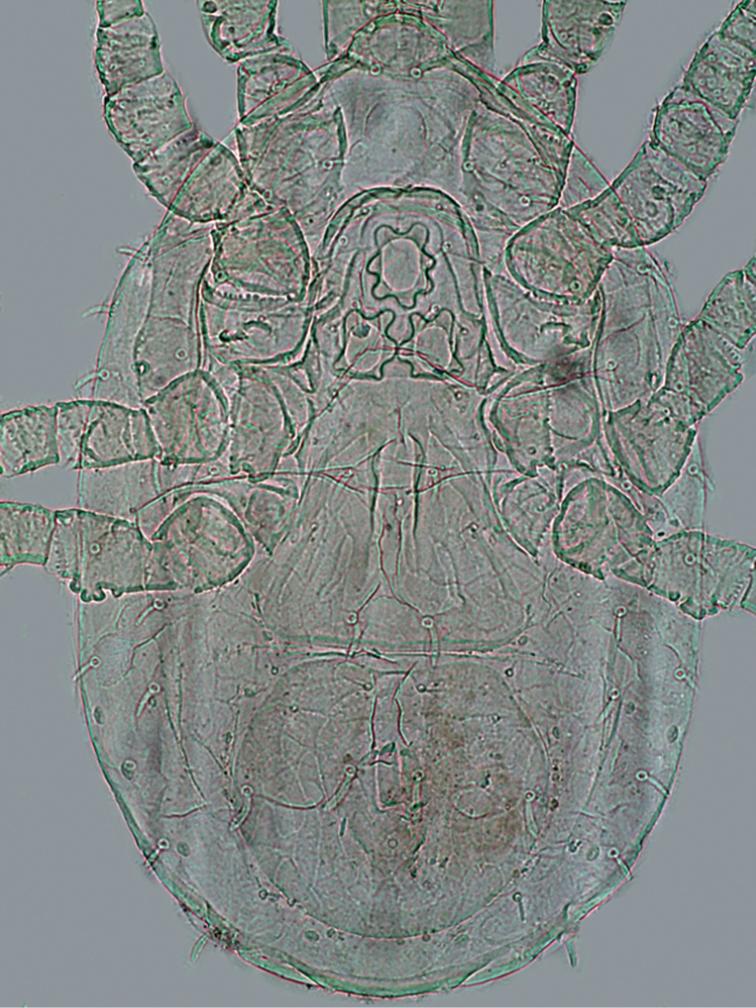
*Sertitympanum contiguum*, female (from U.S.A.), idiosoma (pressed specimen).

**Plate 72.**
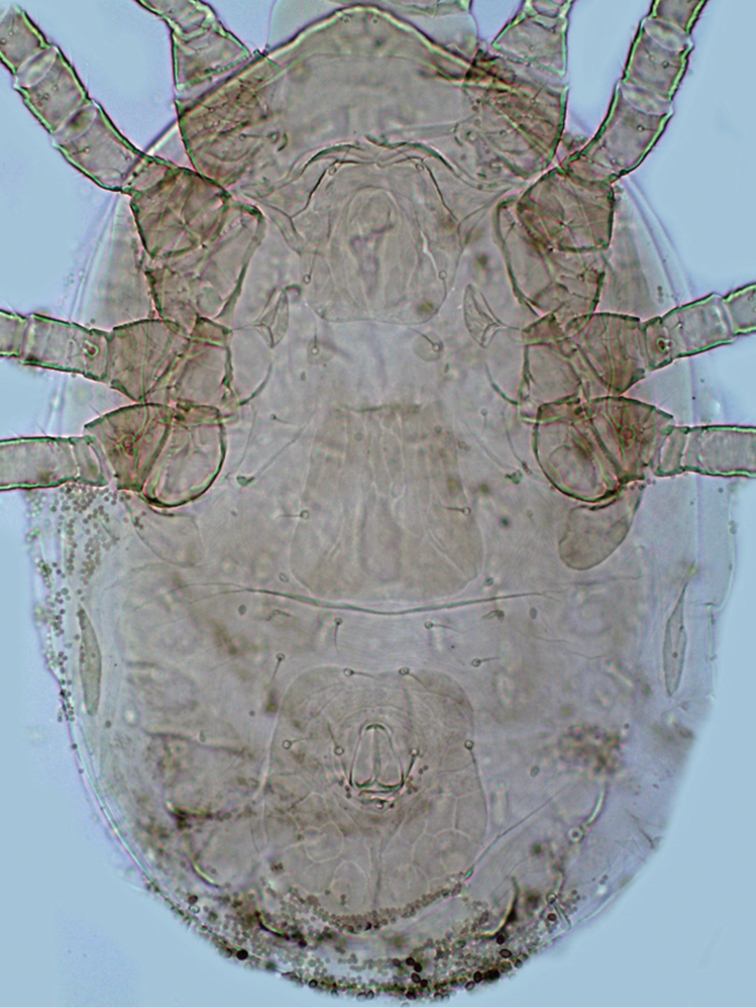
*Sertitympanum* sp., female (from Iran), ventral idiosoma (pressed specimen).

**Plate 73.**
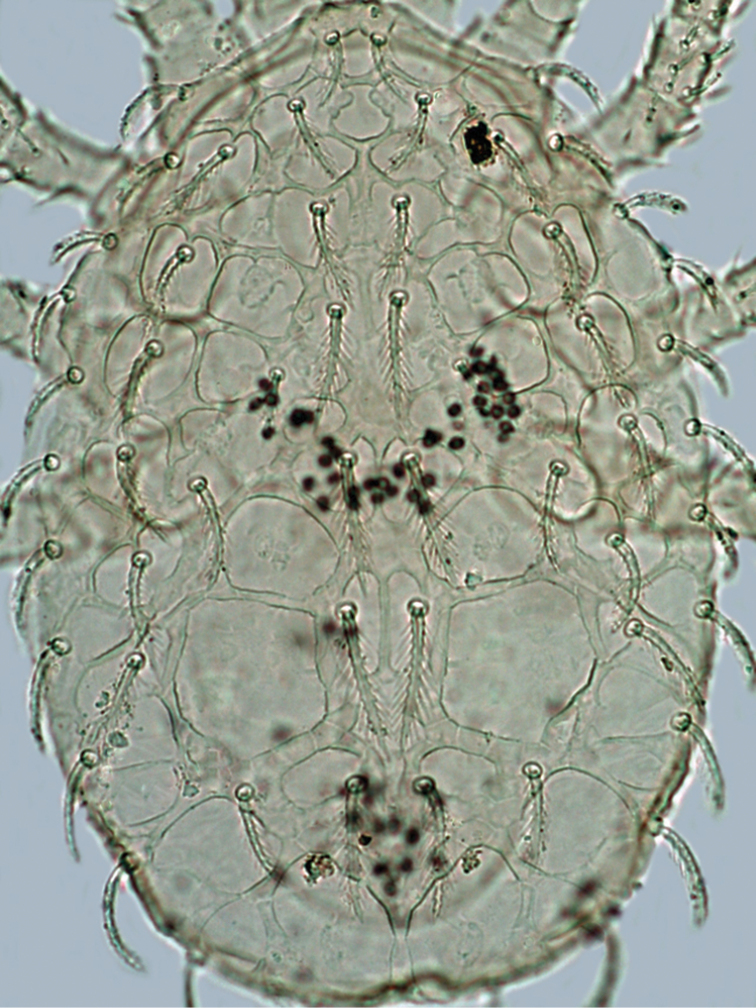
*Sinoseius lobatus*, female, dorsal idiosoma (pressed specimen).

**Plate 74.**
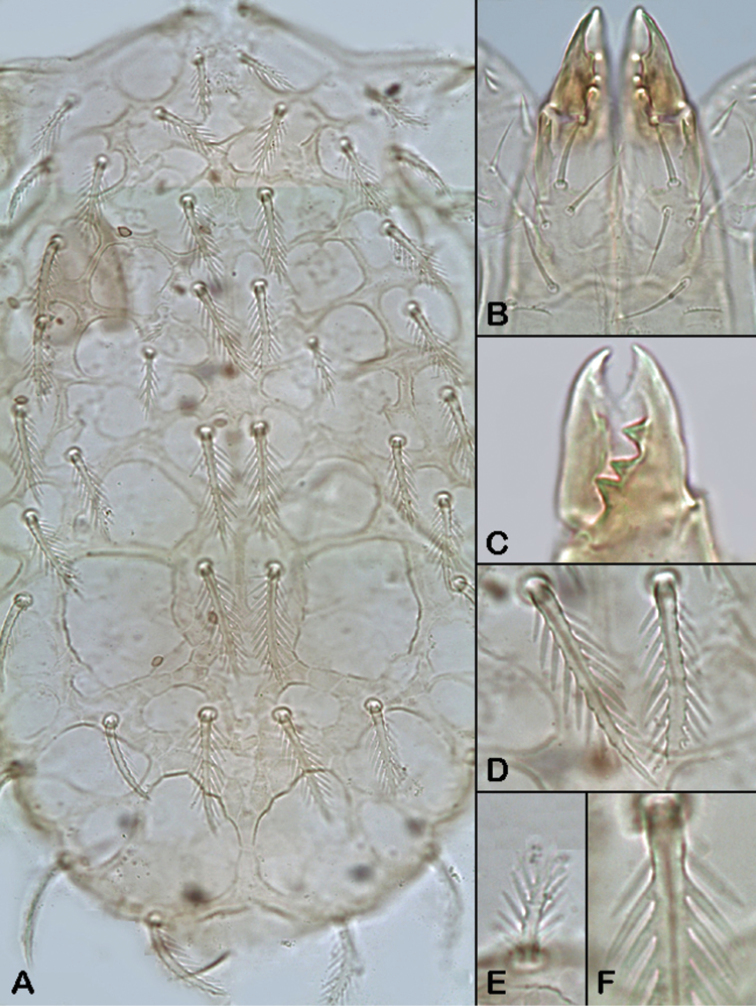
*Sinoseius lobatus*, female. **A** Dorsal idiosoma (pressed specimen) **B** Hypostome, ventral view **C** Chelicera, lateral view **D** Dorsal setae **E** Setae j1 **F** Detail of pinnate structure of dorsal seta.

**Plate 75.**
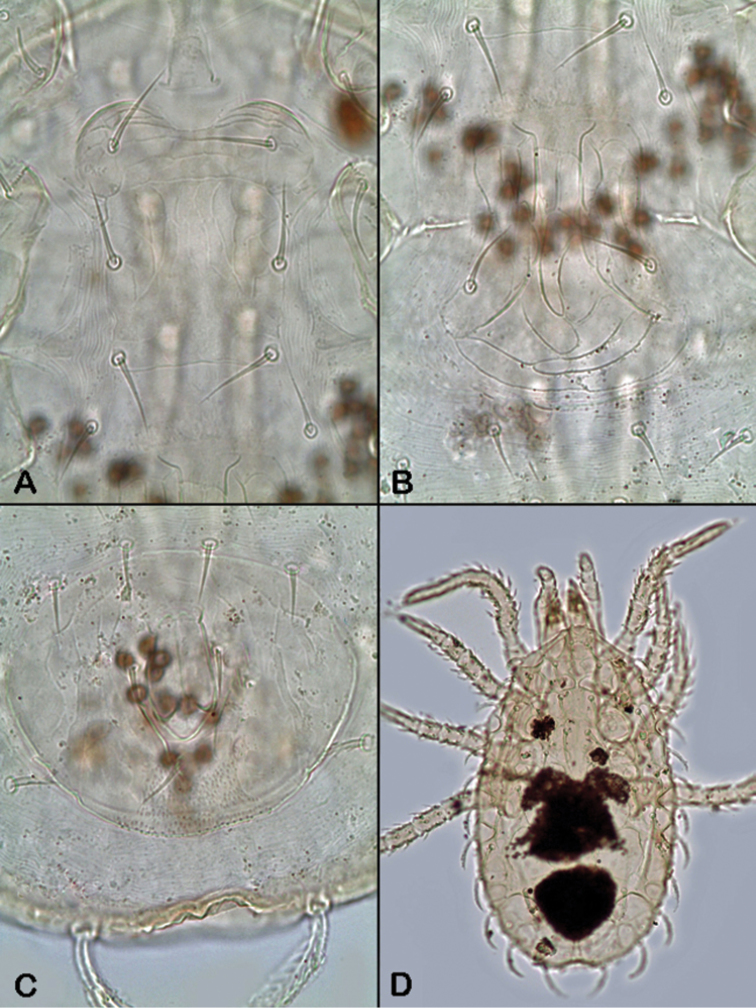
*Sinoseius lobatus*, female. **A** Sternal shield **B** Epigynal shield **C** Anal shield **D** Dorsal idiosoma with legs.

## Plates

Each plate is accompanied by a detailed legend, and mostly based on specimens from Slovakia (if not otherwise specified). For quick reference, the plate contents are summarised in the following list.

1–2. *Ameroseiella macrochelae*

3. *Ameroseius callosus*

4–5. *Ameroseius cavernosus*

6–8. *Ameroseius corbiculus*

9–10. *Ameroseius corniculus*

11–13. *Ameroseius fungicola*

14–16. *Ameroseius furcatus*

17–18. *Ameroseius georgei*

19. *Ameroseius lehtineni*

20–21. *Ameroseius lidiae*

22–23. *Ameroseius longitrichus*

24–25. *Ameroseius renatae*

26–27. *Ameroseius sculptilis*

28. *Ameroseius ulmi*

29. *Epicriopsis horridus*

30. *Epicriopsis hungaricus*

31–32. *Epicriopsis mirabilis*

33. *Epicriopsis palustris*

34. *Epicriopsis suedus*

35–36. *Kleemannia delicata*

37. *Kleemannia delicata*, *Kleemannia pavida*

38. *Kleemannia dolichochaeta*

39–40. *Kleemannia insignis*

41–42. *Kleemannia kosi*

43–44. *Kleemannia nova*

45. *Kleemannia parplumosa*

46–47. *Kleemannia pavida*

48–49. *Kleemannia plumea*

50–52. *Kleemannia plumigera*

53–56. *Kleemannia plumosa*

57–60. *Kleemannia pseudoplumosa*

61–63. *Kleemannia tenella*

64–66. *Neocypholaelaps favus*

67. *Pseudoameroseius michaelangeli*

68. *Sertitympanum aegyptiacum*

69. *Sertitympanum contiguum*

70–71. *Sertitympanum nodosum*

72. *Sertitympanum* sp.

73–75. *Sinoseius lobatus*

76. Sperm induction structures and chelicerae.

77. Peritrematal shields, and metasternal region.
